# Atroposelective Synthesis of Axially Chiral Naphthylpyrroles by a Catalytic Asymmetric 1,3-Dipolar Cycloaddition/Aromatization Sequence

**DOI:** 10.1021/acs.orglett.3c04261

**Published:** 2024-01-24

**Authors:** Ian Maclean, Enrique Gallent, Oscar Orozco, Alba Molina, Nuria Rodríguez, Javier Adrio, Juan C. Carretero

**Affiliations:** † Departamento de Química Orgánica, Facultad de Ciencias, Universidad Autónoma de Madrid, 28049 Madrid, Spain; ‡ Institute for Advanced Research in Chemical Sciences (IAdChem) and Center for Innovation in Advanced Chemistry (ORFEO-CINQA), Universidad Autónoma de Madrid, 28049 Madrid, Spain

## Abstract

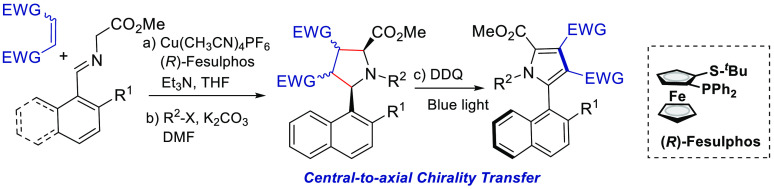

A straightforward methodology for the enantioselective preparation of axially chiral 2-naphthylpyrroles has been developed. This protocol is based on a Cu^I^/Fesulphos-catalyzed highly enantioselective 1,3-dipolar cycloaddition of an azomethine ylide followed by pyrrolidine alkylation and pyrrolidine to pyrrole oxidation. The mild conditions employed in the DDQ/blue light-mediated aromatization process facilitate an effective central-to-axial chirality transfer affording the corresponding pyrroles with high atroposelectivity.

Axially chiral molecules are key structures in organic and medicinal chemistry, present in numerous natural and biologically active compounds,^1^ and considered privileged chiral ligands for transition metal catalysis and organocatalyzed procedures.^2^ Therefore, the development of new methodologies for their efficient preparation has become a hot research topic, and many strategies have been recently described to fill significant gaps and expand the chemical space of this class of compounds. However, most contributions focus on the preparation of atropisomeric biaryls (6,6-ring systems), while the enantioselective assembly of five-membered heteroaryl atropisomers has been much less documented.^3^ This situation could be attributed to the lower conformational stability caused by the modified bond angles of the five-membered ring that increase the distance between substituents, therefore decreasing the rotation barriers.^4^

Pyrrole cores are present in a variety of natural products and are valuable building blocks for the preparation of pharmaceuticals and new materials.^5^ Consequently, strategies for the catalytic atroposelective synthesis of axially chiral arylpyrrole derivatives are highly appealing. In the past few years, several catalytic asymmetric procedures for the preparation of axially chiral pyrroles have been reported. However, most of them allow access to only pyrrole-derived atropisomers with a C–N^6^ rotational axis. The preparation of pyrroles with N–N^7^ or C–C^8^ bonds has been much less studied (Scheme 1). In fact, to the best of our knowledge, only three procedures describe the enantioselective preparation of axially chiral C2-arylpyrroles. In 2019, Tan and co-workers^9^ developed an elegant direct chirality transfer strategy by cyclization of enantioenriched atropisomeric alkenes synthesized by organocatalytic asymmetric N-alkylation reactions (Scheme 1a). More recently, Wang, Xu, Mei, and co-workers^10^ have reported a direct phosphoric acid-catalyzed asymmetric Attanasi reaction, between 1,3-dicarbonyl compounds and azoalkenes, to afford C2 naphthylpyrroles in high yields and excellent enantioselectivities (Scheme 1b). Finally, during the completion of this work, Ullah, Lu, and co-workers^11^ have described the atroposelective synthesis of CF_3_-substituted 2-aryl pyrroles by the phosphine-catalyzed cycloaddition of aldimines with allenoates and subsequent oxidation (Scheme 1c).

**Scheme 1:**
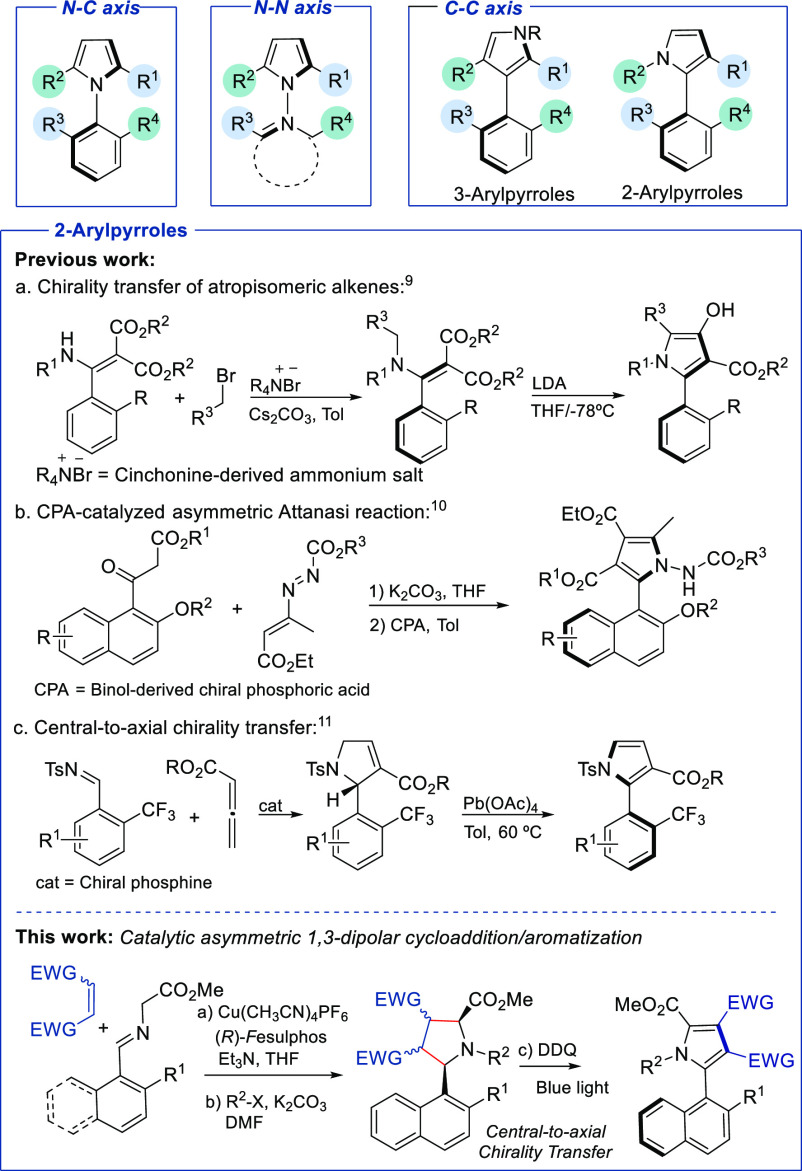
Synthesis of C–C Axially Chiral 2-Arylpyrroles

The 1,3-dipolar cycloaddition of azomethine ylides with olefins offers direct access to highly functionalized proline derivatives.^12^ Since 2002, numerous well-defined catalytic systems capable of giving rise to excellent enantioselectivities have been developed. On the contrary, several research groups have reported different protocols for the dehydrogenative oxidation from pyrrolidines to pyrroles.^13^ Thus, in connection with our previous work in 1,3-dipolar cycloaddition and pyrrole synthesis,^14^ we envisaged that a metal-catalyzed asymmetric cycloaddition of azomethine ylides and subsequent oxidation of the resulting pyrrolidine could provide an expeditious route to a new class of axially chiral C2-aryl pyrroles, via a central-to-axial chirality transfer strategy (Scheme 1).^15^ Nonetheless, to achieve this objective, two main issues had to be addressed: (a) the development of a robust asymmetric 1,3-dipolar cycloaddition leading to the preparation of highly enantioenriched pyrrolidines decorated with suitable groups around the C–C axis to avoid free rotation in the final pyrrole core and (b) the achievement of a mild pyrrolidine to pyrrole oxidation process that would allow the efficient central-to-axial chirality transfer.

We began our investigation by exploring the cycloaddition between iminoester **1a** (obtained by condensation of 2-methyl-1-naphthaldehyde and methyl glycinate) and methyl maleimide (**2**). On the basis of our previous work,^16^ a Fesulphos (**4**)/Cu^I^ complex was initially used as the catalytic system. To our delight, using Et_3_N as the base and THF as the solvent, the corresponding pyrrolidine *endo*-**3a** was obtained as the only detectable diastereoisomer in 87% yield and 83% ee (Scheme 2a). This ee could be increased to 87% by performing the reaction at 0 °C. The use of other metal complexes, bases, or solvents did not bring any further improvement.^17^ A significant decrease in conversion and asymmetric induction was observed when the catalyst loading was reduced to 5 mol %.

**Scheme 2:**
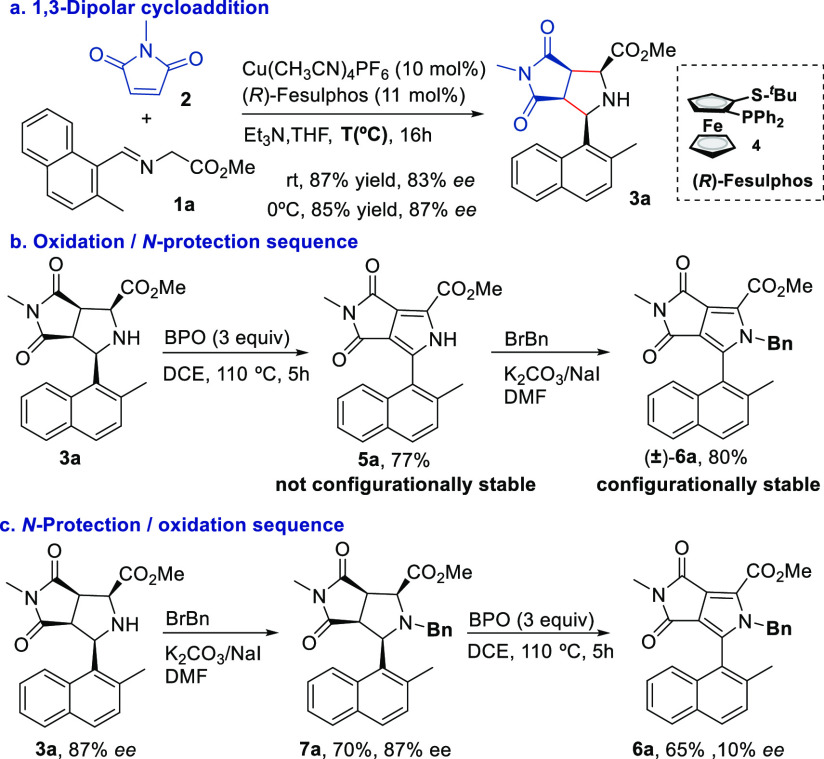
Synthesis of C–C Axially Chiral 2-Arylpyrroles by a 1,3-Dipolar Cycloaddition–Aromatization Sequence

At this point, our next purpose was to evaluate the pyrrolidine to pyrrole dehydrogenative aromatization process. We were pleased to find that racemic pyrrolidine *endo*-**3a** was easily oxidized to the corresponding pyrrole **5a** using benzoyl peroxide (BPO) in DCE at 110 °C (Scheme 2b).^13^ Unfortunately, all attempts to separate the mixture of enantiomers by HPLC with different chiral supports failed, and pyrrole **5a** prepared from enantioenriched pyrrolidine **3a** (87% ee) showed null optical rotation, suggesting that **5a** is not configurationally stable. However, the straightforward benzylation of **5a** led to *N*-benzyl pyrrole **6a**, which was easily resolved by chiral HPLC using standard conditions (CHIRALPAK IA-HPLC column) (Scheme 2b). Hence, to develop an oxidation process with central-to-axial chirality transfer, we focused on evaluating the oxidation of *N*-benzyl pyrrolidine **7a**. This reaction using BPO as the oxidant proceeded in good yield (65%) but with a severe erosion of the enantioselectivity [87% to 10% ee (Scheme 2c)].

Therefore, on the basis of literature precedent, we proceeded to evaluate other possible oxidants (Table 1).^13^ The use of 2,3-dichloro-5,6-dicyano-1,4-benzoquinone (DDQ) as an oxidant led to the formation of **6a** in high yield but with low enantioselectivity (entry 1). In an attempt to improve the enantiomeric excess, the reaction was then carried out at −15 °C (entry 2). However, at such a low temperature, starting material **7a** was recovered unaltered. This lack of reactivity was also observed when CuCl/TEMPO/O_2_- or MnO_2_-based systems were used as oxidants (entry 3 or 4, respectively). Considering that the oxidizing ability of DDQ improves significantly upon visible light excitation, we decided to perform the oxidation of **7a** with DDQ under blue light irradiation.^18^ Using this system, and under strictly anhydrous conditions,^19^ we were able to obtain **6a** in 98% isolated yield and 60% ee (entry 5). The ee could be improved to 72% by decreasing the temperature to −15 °C (entry 6). Finally, pyrrolidine **8a** with the bulkier 2,4,6-trimethylbenzyl moiety provided the best result, affording the corresponding pyrrole **9a** with 84% ee (entry 7). However, the oxidation with *N*-acyl- and *N*-tosyl-protected pyrrolidines did not occur, affording the starting material unaltered (entries 8 and 9, respectively).^20^

**Table 1:**
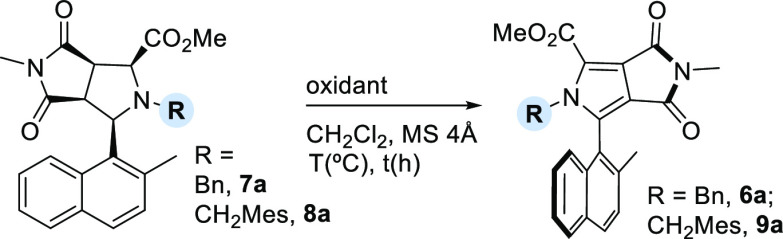
Optimization of the Aromatization Conditions

entry	oxidant	R	*T* (°C)	*t* (h)	yield (%)^a^	ee (%)^b^
1	DDQ	Bn	rt	24	98	50
2	DDQ	Bn	–15	24	nd^c^	–
3	Cu^I^/TEMPO/O_2_	Bn	80	24	5	–
4	MnO_2_	Bn	rt	24	nd^c^	–
5	DDQ/blue light	Bn	rt	1	98	60
6	DDQ/blue light	Bn	–15	1	98	72
7	DDQ/blue light	CH_2_Mes	–15	4	98	84
8	DDQ/blue light	Ac	–15	4	nd^c^	–
9	DDQ/blue light	Ts	–15	4	nd^c^	–

a Isolated yield after chromatographic purification.

b ee determined by HPLC.

c Not detected.

After establishing the optimal reaction conditions,^21^ we set out to study the structural generality of this cycloaddition dehydrogenative aromatization sequence. First, we evaluated the scope of the 1,3-dipolar cycloaddition regarding the substitution at the azomethine ylide. As shown in Scheme 3a, aromatic iminoesters with different electron-donating and electron-withdrawing groups furnished the corresponding pyrrolidines in high yields and excellent enantioselectivities (**3b**–**g**). After reaction with bromomethylmesitylene using standard conditions, 2-naphthol pyrrolidines with different O-protected groups (**3b**–**d**) were tested in the subsequent aromatization process, affording the corresponding pyrroles (**9b**–**d**, respectively) in moderate yields and high enantioselectivity [85–97% ee (Scheme 3b)]. Thus, aromatization of pyrrolidine **8e**, bearing a phenyl group at position 2 of the naphthyl moiety, led to the corresponding pyrrole derivative **9e** with moderate enantioselectivity (70% ee). Likewise, *N*-(2,4,6-trimethylbenzyl)-2-bromonaphthyl pyrrolidine **8f** was also compatible with the aromatization conditions, affording pyrrole **9f** with excellent enantioselectivity (99% ee). No enantioselectivity was observed when pyrrolidine **8g** was subjected to the aromatization conditions, confirming that the presence of a substituent at position 2 of the naphthyl moiety is essential to achieve an effective central-to-axial chirality transfer. The absolute configuration of pyrroles **9** was unequivocally established by X-ray diffraction of bromo derivative **9f** (see the Supporting Information for details; Scheme 3).^22^

**Scheme 3:**
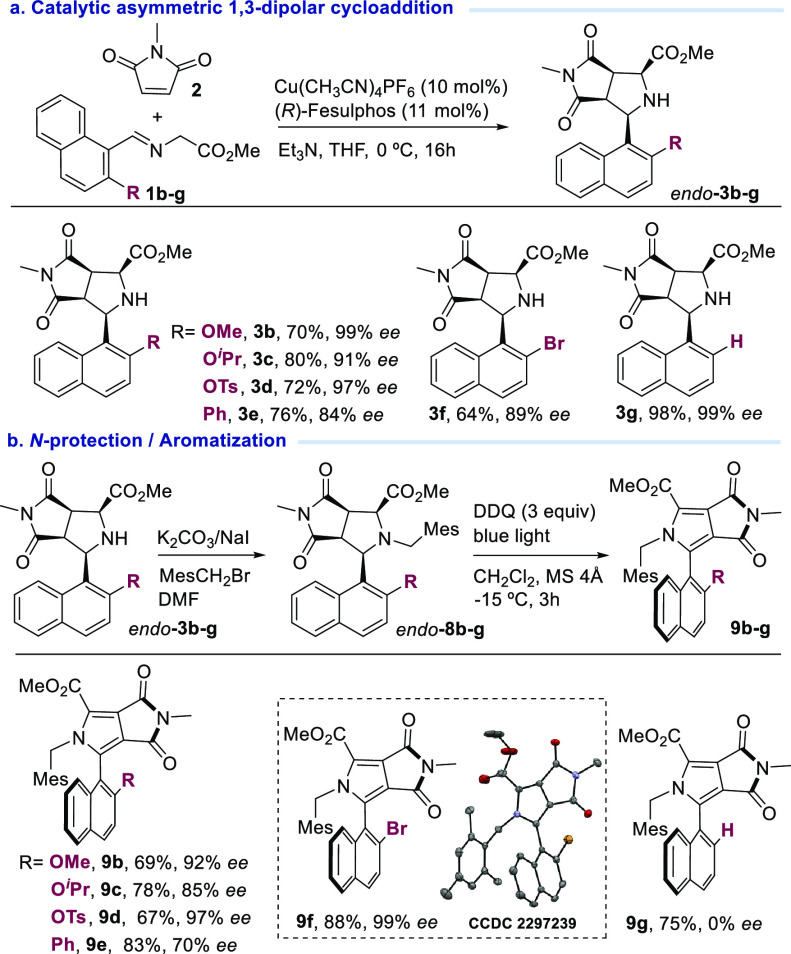
Scope of the Cycloaddition–Aromatization Sequence Using 1-Naphthyl Iminoesters, Isolated yield after chromatographic purification. ee determined by HPLC.

Next, to expand the scope of the reaction, we applied this methodology to the case of 2-naphthyl iminoesters (Scheme 4). Remarkably, under similar reaction conditions, the cycloaddition dehydrogenative aromatization sequence using iminoesters **10a**–**c** led to pyrroles **13a**–**c** respectively, with high yields (70–92%) and enantioselectivities [90–99% ee (Scheme 4)]. The presence of a halogen or alkyl substituent at position 2 of the naphthyl moiety allowed for effective central-to-axial chirality transfer. However, aryl-substituted derivative **13d** was obtained as a racemic mixture. Finally, the presence of less hindered substituents such as OMe or O^*i*^Pr in the naphthyl moiety led to not configurationally stable derivatives (**13e** and **13f**). In this series, the absolute configuration of pyrrole type **13** was established by X-ray diffraction of bromo derivative **13a** (see the Supporting Information for details; Scheme 4b).^23^

**Scheme 4:**
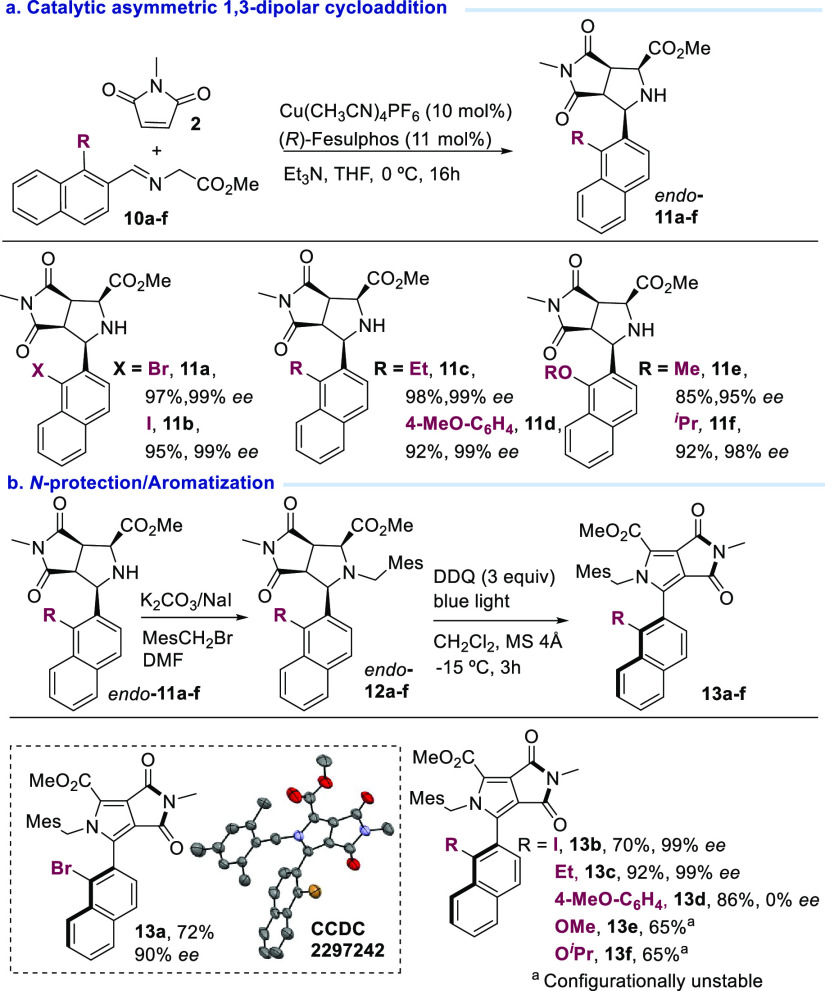
Cycloaddition–Aromatization Sequence for 2-Naphthyl Iminoesters, Isolated yield after chromatographic purification. ee determined by HPLC.

We next studied the compatibility of the procedure with other dipolarophiles (Scheme 5). *N*-Phenylmaleimide (**14a**) was an effective partner for the cycloaddition providing the corresponding pyrrolidine **15a** in 99% ee. Linear diactivated (*E*)-alkenes such as fumarate (**14b** and **14c**) or fumaronitrile (**14d**) also proved to be excellent dipolarophiles affording the corresponding pyrrolidines with almost complete *endo* diastereoselectivity and excellent enantioselectivity [adducts **15b** (88% ee), **15c** (81% ee), and **15d** (98% ee)].^24^*N*-2,4,6-Trimethylbenzyl pyrrolidine **16a** with a phenyl substituent in the maleimide moiety successfully participated in the aromatization reaction to generate pyrrole **17a** with low yield (47%) but high atroposelectivity (86% ee). However, the aromatization of pyrrolidine **16b** resulting from the cycloaddition with dimethyl fumarate and subsequent benzylation took place with good yields (71%) but lower atroposelectivity (60% ee) (**17b**). This loss of effectiveness in the central-to-axial chirality transfer could be explained by steric effects. When di-*tert*-butyl fumarate was used, a higher chirality transfer efficiency was observed [**17c** (69%, 78% ee)]. Remarkably, the central-to-axial chirality transfer is almost complete in the case of pyrrolidine **17d** owing to two cyano groups at positions 3 and 4 of the pyrrolidine ring (56% yield, 99% ee).

**Scheme 5:**
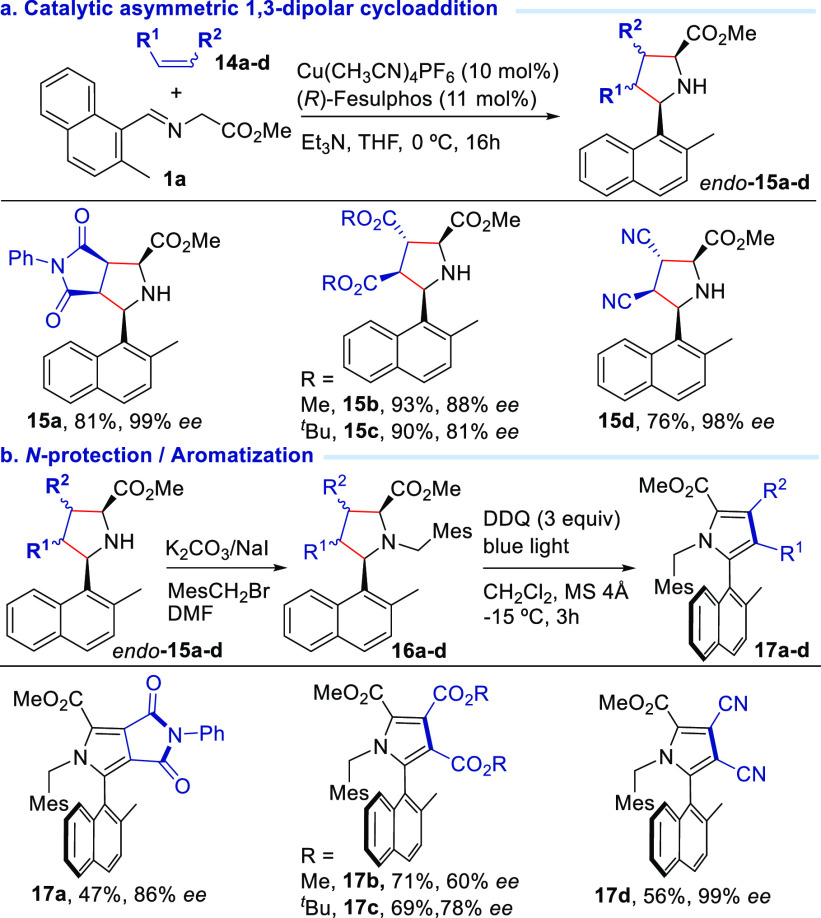
Cycloaddition Aromatization Process Using Different Dipolarophiles, Isolated yield after chromatographic purification. ee determined by HPLC.

To shed some light on stereochemical stability of naphthyl pyrrole derivatives, racemization experiments were conducted in xylene at 130 °C for 8 h (Figure 1). After 10 measurements of the enantiomeric excess had been taken, the values obtained for the energy barrier for compounds **17a** and **9c** were 33.6, and 33.8 kcal/mol, respectively, confirming the configurational stability of these atropisomers at room temperature. A lower energy barrier was obtained for **13a** (29.1 kcal/mol at 110 °C).

**Figure 1:**
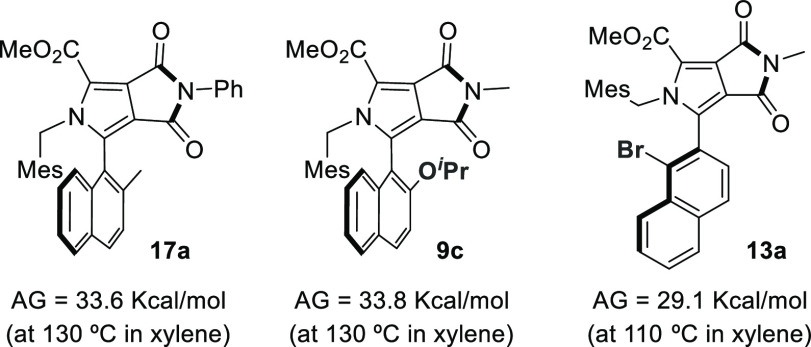
Determination of rotation barriers.

In conclusion, an innovative procedure for the preparation of C–C axially chiral naphthylpyrroles was developed using a 1,3-dipolar cycloaddition/oxidation sequence. Excellent yields and enantioselectivities were obtained in the 1,3-dipolar cycloaddition using the Cu(I)/Fesulphos complex as the catalyst system. Other key features of this approach are the proper N-alkylation of the pyrrolidine adduct and the final low-temperature DDQ/blue light-mediated pyrrolidine to pyrrole oxidation process for effective central-to-axial chirality transfer.

## Data Availability

The data underlying this study are available in the published article and its Supporting Information.

## Supporting Information Available

General experimental procedures, characterization data, and NMR spectra (PDF)
