# Supplementary material for: Atroposelective Synthesis of Axially Chiral Naphthylpyrroles by a Catalytic Asymmetric 1,3-Dipolar Cycloaddition/Aromatization Sequence
Source: Org Lett. 2024 Jan 24;26(4):922–7. doi: 10.1021/acs.orglett.3c04261 (PMC10845160; doi:10.1021/acs.orglett.3c04261)
Supplement: Supplementary file 1 — ol3c04261_si_001.pdf [file ol3c04261_si_001.pdf]

Supporting Information

Atroposelective Synthesis of Axially Chiral Naphthylpyrroles by Catalytic Asymmetric 1,3-Dipolar Cycloaddition/Aromatization Sequence

Ian Maclean,[a] Enrique Gallent,[a] Oscar Orozco,[a] Alba Molina,[a] Nuria Rodríguez, [a,b] Javier Adrio,*[a,b] and Juan C. Carretero*[a,b]

javier.adrio@uam.es, juancarlos.carretero@uam.es

[a] Departamento de Química Orgánica, Facultad de Ciencias, Universidad Autónoma de Madrid, 28049 Madrid, Spain.

[b] Institute for Advanced Research in Chemical Sciences (IAdChem) and Center for Innovation in Advanced Chemistry (ORFEO-CINQA) Universidad Autónoma de Madrid, 28049 Madrid, Spain.

1. General Methods	S2
2. Optimization of the catalytic asymmetric 1,3-dipolar cycloaddition	S3
3. Synthesis of α-iminoesters	S4
4. Cu-catalyzed asymmetric 1,3-dipolar cycloaddition of azomethine ylides	S7
5. Blue light promoted aromatization of pyrrolidines	S15
6. Synthetic method at a 1 mmol scale for the preparation of **9a**	S25
7. Evaluation of the rotational barriers of **17a**, **9c** and **13a**	S26
8. Mechanistic proposal for the DDQ-mediated oxidation	S29
9. HPLC charts	S30
10. NMR spectra collection	S54
11. References	S100

1. General Methods

All anaerobic and moisture-sensitive manipulations were carried out in anhydrous solvents and under argon pressure. Dichloromethane and tetrahydrofuran were dried over the PureSolv MD purification system. All general reagents were obtained from usual commercial sources and were used, without further purification.

All general reagents were obtained from usual commercial sources and were used, except where indicated, without further purification. *N*-Methylmaleimide (**2**) and *N*-phenylmaleimide (**14a**) were purchased from Aldrich Chemical Co. (*R*)-Fesulphos (**4**) was purchased from Aldrich Chemical Co. 2-Methyl-1-naphthaldehyde, 2-methoxy-1-naphthaldehyde, 2-bromo-1-naphthaldehyde, 1-naphthaldehyde, 1-bromo-2-naphthaldehyde, 1-methoxy-2-naphthaldehyde and glycine methyl ester hydrochloride were purchased from BLD Pharmatech Co.

Reactions were monitored by thin-layer chromatography carried out on 0.25 mm silica 230-400 mesh gel plates. Flash column chromatography was performed using 230-400 mesh ultra-pure silica gel.

NMR-spectra were obtained using chloroform-d as solvent in a 300 or 500 MHz spectrometer with proton and carbon resonances at 300/500 MHz and 75/126 MHz, respectively. ^{13}C NMR experiments are ^{1}H decoupled.

HRMS spectra were recorded on a VG *AutoSpec mass* spectrometer with electrospray ionization (ES) as the ionization source. The HPLC chromatograms of the racemic and enantiomerically enriched cycloadducts are also included.

The aldehydes needed for the preparation of α-iminoesters (**1a**-**g**, **10a**-**e**) were previously reported.[1-6] Due to their lability all the α-iminoesters once isolated were immediately used in the 1,3-dipolar cycloaddition without further purification.

2. Optimization of the catalytic asymmetric 1,3-dipolar cycloaddition

Table S1. Optimization of the catalytic asymmetric 1,3-dipolar cycloaddition

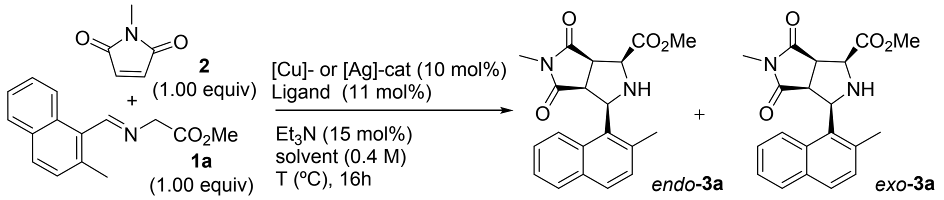

Entry	Catalyst	Ligand	Solvent	T(ºC)	**3a** (%)[a]	*exo*-**3a**/*endo*-**3a**[b]	*endo*-**3a** *ee* (%)[c]
1	$Cu(CH_3CN)_4PF_6$	(*R*)-Segphos	THF	25	75	30/70	75
2	$Cu(CH_3CN)_4PF_6$	(*R*)-DTBMSegphos	THF	25	50	90/10	50
3	$Cu(CH_3CN)_4PF_6$	(*R*)-Fesulphos	THF	25	85	20/80	85
4	$Cu(CH_3CN)_4PF_6$	(*R*)-Fesulphos	THF	25	46	20/80	46
5	$Cu(CH_3CN)_4PF_6$	(*R*)-Fesulphos	THF	25	-	-	-
6	$Cu(CH_3CN)_4PF_6$	(*R*)-Fesulphos	THF	0	85	1/99	85
7	$Cu(CH_3CN)_4PF_6$	(*R*)-Fesulphos	THF	- 50	85	1/99	85
8	$Cu(CH_3CN)_4PF_6$	(*R*)-Fesulphos	Tolueno	0	61	1/99	61
9	$Cu(CH_3CN)_4PF_6$	(*R*)-Fesulphos	DCM	25	65	1/99	65
10	AgOEt	(*R*)-Fesulphos	THF	0	61	31/69	61

[a] Isolated yield after chromatographic purification. [b]*exo*-**3a**/*endo*-**3a** determined by ^{1}H NMR. [c]*ee* determined by HPLC.

3. Synthesis of α-iminoesters

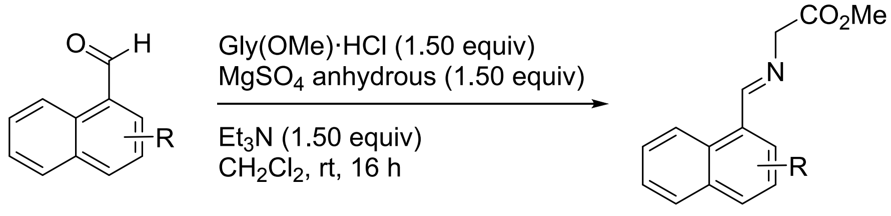

1a-g, 10a-f

General procedure. To a suspension of the corresponding aldehyde (1.20 mmol, 1.00 equiv), glycine methyl ester hydrochloride (227 mg, 1.81 mmol, 1.50 equiv) and anhydrous $MgSO_4$ (217 mg, 1.81 mmol, 1.50 equiv) in dry dichloromethane (3.00 mL), Et_3N (0.26 mL, 1.81 mmol, 1.50 equiv) was added. After stirring the mixture at room temperature for 16 h, water (10 mL) was added. The organic layer was separated, and the aqueous phase was extracted with dichloromethane (3 x 10 mL). The combined organic layers were dried over $MgSO_4$ and evaporated under reduced pressure to afford to afford the crude α-iminoesters which were used without further purification in the next reaction step.

Methyl (*E*)-2-(((2-methylnaphthalen-1-yl)methylene)amino)acetate (1a). Compound

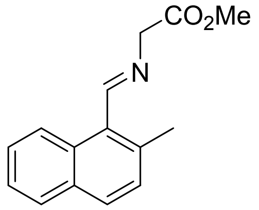

1a was prepared following the general procedure from 2-methyl-1-naphthaldehyde (204 mg, 1.20 mmol, 1.00 equiv) to afford **1a** (241 mg, 87%, yellow oil). **^{1}H NMR (300 MHz, $CDCl_3$, δ):** 8.77 (s, 1H), 8.68 (d, *J* = 8.5 Hz, 1H), 7.82 (d, *J* = 8.1 Hz, 1H), 7.76 (d, *J* = 8.4 Hz, 1H), 7.64 – 7.55 (m, 1H), 7.52 – 7.43 (m, 1H), 7.27 (d, *J* = 8.4 Hz, 1H), 4.55 (s, 2H), 3.85 (s, 3H), 2.55 (s, 3H).

Methyl (*E*)-2-(((2-methoxynaphthalen-1-yl)methylene)amino)acetate (1b).

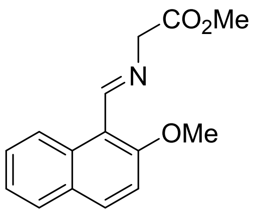

Compound **1b** was prepared following the general procedure from 2-methoxy-1-naphthaldehyde (224 mg, 1.20 mmol, 1.00 equiv) to afford **1b** (306 mg, 99%, blue oil). **^{1}H NMR (300 MHz, $CDCl_3$, δ):** 9.27 (d, *J* = 8.7 Hz, 1H), 9.03 (s, 1H), 7.91 (d, *J* = 9.1 Hz, 1H), 7.77 (d, *J* = 8.1 Hz, 1H), 7.56 (ddd, *J* = 8.6, 6.8, 1.4 Hz, 1H), 7.37 (ddd, *J* = 8.0, 6.8, 1.1 Hz, 1H), 7.26 (d, *J* = 9.1 Hz, 1H), 4.56 (s, 2H), 3.99 (s, 3H), 3.82 (s, 3H).

Methyl (*E*)-2-(((2-isopropoxynaphthalen-1-yl)methylene)amino)acetate (1c).

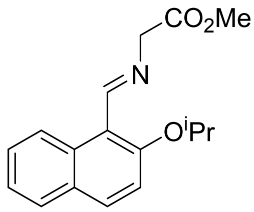

Compound **1c** was prepared following the general procedure from 2-isopropoxy-1-naphthaldehyde[1] (257 mg, 1.20 mmol, 1.00 equiv) to afford **1c** (336 mg, 98%, yellow oil). **^{1}H NMR (300 MHz, $CDCl_3$, δ):** 9.31 (d, *J* = 9.3 Hz, 1H), 9.02 (s, 1H), 7.86 (d, *J* = 9.1 Hz, 1H), 7.75 (d, *J* = 8.1 Hz, 1H), 7.61 – 7.48 (m, 1H), 7.43 – 7.31 (m, 1H), 7.23 (d, *J* = 9.0 Hz, 1H), 4.71 (hept, *J* = 5.7 Hz, 1H), 4.55 (s, 2H), 3.82 (s, 3H), 1.40 (d, *J* = 5.7 Hz, 3H), 1.38 (d, *J* = 5.7 Hz, 3H).

Methyl (*E*)-2-(((2-(tosyloxy)naphthalen-1-yl)methylene)amino)acetate (1d).

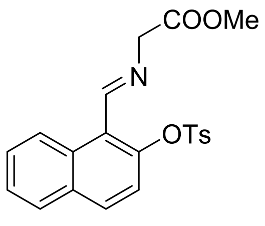

Compound **1d** was prepared following the general procedure from 1-formylnaphthalen-2-yl 4-methylbenzenesulfonate[2] (392 mg,

1.20 mmol, 1.00 equiv) to afford **1d** (467 mg, 98%, yellow oil). **^{1}H NMR (300 MHz, $CDCl_3$, δ):** 9.21 (d, *J* = 8.6 Hz, 1H), 8.54 (s, 1H), 7.83 (d, *J* = 9.0 Hz, 1H), 7.78 (d, *J* = 8.1 Hz, 1H), 7.68 (d, *J* = 8.1 Hz, 2H), 7.62 – 7.53 (m, 1H), 7.51 – 7.42 (m, 1H), 7.30 – 7.22 (m, 3H), 4.28 (s, 2H), 3.79 (s, 3H), 2.36 (s, 3H).

Methyl (*E*)-2-(((2-phenylnaphthalen-1-yl)methylene)amino)acetate (1e). Compound **1e** was prepared following the general procedure from 2-phenyl-1-naphthaldehyde[3] (279 mg, 1.20 mmol, 1.00 equiv) to afford **1e** (346 mg, 95%, yellow oil). **^{1}H NMR (300 MHz, $CDCl_3$, δ):** 9.19 (d, *J* = 8.7 Hz, 1H), 8.43 (s, 1H), 7.94 (d, *J* = 8.5 Hz, 1H), 7.89 (d, *J* = 8.2 Hz, 1H), 7.69 – 7.60 (m, 1H), 7.57 – 7.52 (m, 1H), 7.51 – 7.41 (m, 6H), 4.44 (s, 2H), 3.81 (s, 3H).

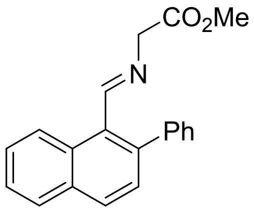

Methyl (*E*)-2-(((2-bromonaphthalen-1-yl)methylene)amino)acetate (1f). Compound **1f** was prepared following the general procedure from 2-bromo-1-naphthaldehyde (282 mg, 1.20 mmol, 1.00 equiv) to afford **1f** (342 mg, 93%, yellow oil). **^{1}H NMR (300 MHz, $CDCl_3$, δ):** 8.93 (d, *J* = 8.3 Hz, 1H), 8.74 (s, 1H), 7.76 – 7.69 (m, 1H), 7.62 – 7.43 (m, 4H), 4.57 (s, 2H), 3.81 (s, 3H).

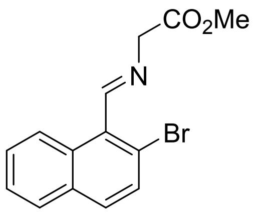

Methyl (*E*)-2-((naphthalen-1-ylmethylene)amino)acetate (1g). Compound **1g** was prepared following the general procedure from 1-naphthaldehyde (187 mg, 1.20 mmol, 1.00 equiv) to afford **1g** (237 mg, 87%, yellow oil). **^{1}H NMR (300 MHz, $CDCl_3$, δ):** 8.94 (s, 1H), 8.90 (d, *J* = 8.5 Hz, 1H), 7.94 (d, *J* = 7.5 Hz, 2H), 7.89 (d, *J* = 8.5 Hz, 1H), 7.64 – 7.48 (m, 3H), 4.54 (s, 2H), 3.81 (s, 3H).

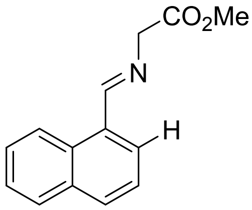

Methyl (*E*)-2-(((1-bromonaphthalen-2-yl)methylene)amino)acetate (10a). Compound **10a** was prepared following the general procedure from 1-bromo-2-naphthaldehyde (282 mg, 1.20 mmol, 1.00 equiv) to afford **10a** (334 mg, 91%, brown oil). **^{1}H NMR (300 MHz, $CDCl_3$, δ):** 8.93 (s, 1H), 8.35 (d, *J* = 7.6 Hz, 1H), 8.14 (d, *J* = 8.6 Hz, 1H), 7.88 – 7.70 (m, 2H), 7.64 – 7.45 (m, 2H), 4.52 (s, 2H), 3.80 (s, 3H).

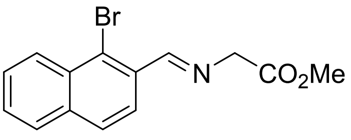

Methyl (*E*)-2-(((1-iodonaphthalen-2-yl)methylene)amino)acetate (10b). Compound **10b** was prepared following the general procedure from 1-iodo-2-naphthaldehyde (338 mg, 1.20 mmol, 1.00 equiv) to afford **10b** (381 mg, 90%, yellow oil). **^{1}H NMR (300 MHz, $CDCl_3$, δ):** 8.98 (s, 1H), 7.81 (d, *J* = 9.3 Hz, 1H), 7.71 (d, *J* = 8.1 Hz, 1H), 7.58 – 7.50 (m, 2H), 7.38 – 7.31 (m, 2H), 4.52 (s, 2H), 3.77 (s, 3H).

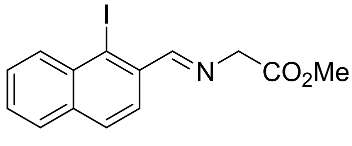

Methyl (*E*)-2-(((1-ethylnaphthalen-2-yl)methylene)amino)acetate (10c). Compound **10c** was prepared following the general procedure from 1-ethyl-2-naphthaldehyde (221 mg, 1.20 mmol, 1.00 equiv) to afford **10c** (300mg, 98%, yellow oil). **^{1}H NMR (300 MHz, $CDCl_3$, δ):** 8.79 (s, 1H), 8.15 (d, *J* = 8.7 Hz, 1H), 8.10 – 7.98 (m, 1H), 7.79 –

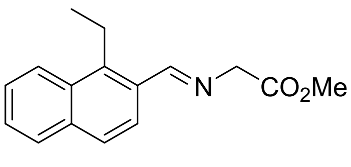

7.73 (m, 1H), 7.67 (d, *J* = 8.7 Hz, 1H), 7.52 – 7.39 (m, 2H), 4.47 (s, 2H), 3.74 (s, 3H), 3.23 (q, *J* = 7.6 Hz, 2H), 1.28 (t, *J* = 7.6 Hz, 3H).

Methyl (*E*)-2-(((1-(4-methoxyphenyl)naphthalen-2-yl)methylene)amino)acetate (10d). Compound **10d** was prepared following the general procedure from 1-(4-methoxyphenyl)-2-naphthaldehyde (315 mg, 1.20 mmol, 1.00 equiv) to afford **10d** (380mg, 95%, yellow oil). **^{1}H NMR (300 MHz, $CDCl_3$, δ):** 8.33 (d, *J* = 8.7 Hz, 1H), 8.21 (s, 1H), 7.90 (d, *J* = 8.4 Hz, 2H), 7.62 (d, *J* = 8.4 Hz, 1H), 7.58 – 7.49 (m, 1H), 7.47 – 7.37 (m, 1H), 7.28 (d, *J* = 8.7 Hz, 2H), 7.07 (d, *J* = 8.7 Hz, 2H), 4.32 (s, 2H), 3.92 (s, 3H), 3.78 (s, 3H).

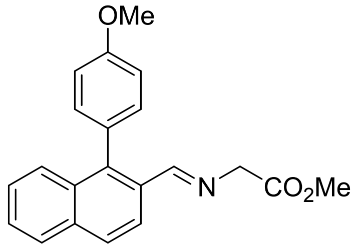

Methyl (*E*)-2-(((1-methoxynaphthalen-2-yl)methylene)amino)acetate (10e). Compound **10e** was prepared following the general procedure from 1-methoxy-2-naphthaldehyde (223 mg, 1.20 mmol, 1.00 equiv) to afford **10e** (287 mg, 93%, yellow oil). **^{1}H NMR (300 MHz, $CDCl_3$, δ):** 8.89 (s, 1H), 8.20 (d, *J* = 8.6 Hz, 2H), 7.88 – 7.82 (m, 1H), 7.66 (d, *J* = 8.7 Hz, 1H), 7.61 – 7.49 (m, 2H), 4.56 (s, 2H), 4.03 (s, 3H), 3.82 (s, 3H).

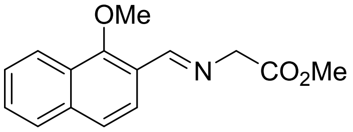

Methyl (*E*)-2-(((1-isopropoxynaphthalen-2-yl)methylene)amino)acetate (10f). Compound **10f** was prepared following the general procedure from 1-isopropoxy-2-naphthaldehyde[1] (257 mg, 1.20 mmol, 1.00 equiv) to afford **10f** (322 mg, 94%, yellow oil). **^{1}H NMR (300 MHz, $CDCl_3$, δ):** 8.81 (s, 1H), 8.17 – 8.06 (m, 2H), 7.84 – 7.76 (m, 1H), 7.58 (d, *J* = 8.6 Hz, 1H), 7.52 – 7.46 (m, 2H), 4.47 (s, 2H), 4.47 – 4.35 (m, 1H), 3.77 (s, 3H), 1.36 (d, *J* = 6.1 Hz, 6H).

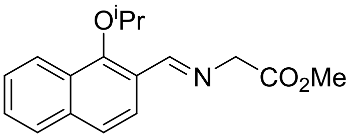

4. Cu-catalyzed asymmetric 1,3-dipolar cycloaddition of azomethine ylides

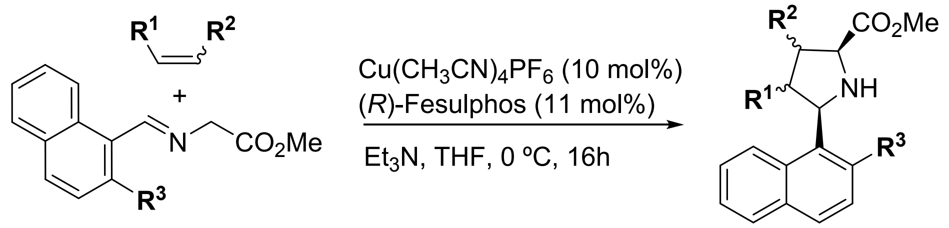

1a-g, 10a-e **3a-g, 11a-f, 15a-d**

General procedure. A sealed vial, was charged with (*R*)-Fesulphos (20 mg, 0.04 mmol, 0.11 equiv) and $Cu(CH_3CN)_4PF_6$ (14.8 mg, 0.04 mmol, 0.10 equiv) in anhydrous THF (3.50 mL) under argon atmosphere. After that, a solution of the corresponding α-iminoesters (0.40 mmol, 1.00 equiv) in THF (1.00 mL) was added. Then, the solution was cold to 0 ºC and Et_3N (8.30 µL, 0.06 mmol, 0.15 equiv) and the corresponding dipolarophile (0.40 mmol, 1.00 equiv) were successively added. After stirring 16 h at 0 ºC, the mixture was diluted with dichloromethane (2.00 mL) and filtered through a short pad of Celite®. The solution was concentrated *in vacuo* and the residue was purified by silica gel flash chromatography.

All the experimental data, global yields and characterization of the synthetized products are described below.

Methyl(1*S*,3*R*,3a*R*,6a*S*)-5-methyl-3-(2-methylnaphthalen-1-yl)-4,6-dioxooctahydropyrrolo[3,4-c]pyrrole-1-carboxylate (3a).

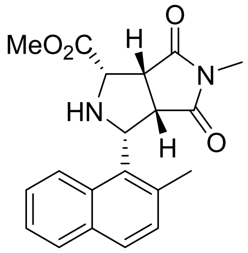

Compound **3a** was prepared following the general procedure from methyl (*E*)-2-(((2-methylnaphthalen-1-yl)methylene)amino)acetate (**1a**) (96.5 mg, 0.40 mmol, 1.00 equiv) and *N*-methylmaleimide (44.4 mg, 0.40 mmol, 1.00 equiv). The residue was purified by silica gel flash chromatography (Heptane-EtOAc 2:1) to afford **3a** (120 mg, 85%, yellow wax). **^{1}H NMR (300 MHz, $CDCl_3$, δ):** 9.01 (d, *J* = 8.7 Hz, 1H), 7.72 (dd, *J* = 7.8, 1.8 Hz, 1H), 7.65 (d, *J* = 8.3 Hz, 1H), 7.36 – 7.21 (m, 3H), 5.11 – 4.83 (m, 1H), 3.87 (s, 3H), 3.86 – 3.81 (m, 1H), 3.52 – 3.43 (m, 1H), 3.34 – 3.27 (m, 1H), 2.68 (s, 3H), 2.50 (s, 3H), 2.38 (bs, 1H). **^{13}C NMR (75 MHz, $CDCl_3$, δ):** 176.5, 174.9, 170.2, 134.4, 133.2, 133.2, 129.1, 128.8, 128.7, 128.4, 126.5, 125.7, 125.1, 124.6, 62.1, 52.2, 47.6, 46.4, 25.1, 21.3. Minor impurities of a non-identified compound remain in the analysed product. **HRMS-ESI (*m/z*):** calcd. for $C_{20}H_{21}N_2O_4$ ($M+H^+$): 353.1496; found: 353.1497.

$[\alpha]_{D\ 20}$: +29.05 (c = 0.3, $CHCl_3$), 87% *ee*.

SFC: Chiralpak IB, CO_2-MeOH 95:5 to 70-30, flow rate 2 mL/min (λ = 260 nm), t_R: 4.24 min (1*S*,3*R*,3a*S*,6a*R*)-**3a** and 6.49 min (1*R*,3*S*,3a*R*,6a*S*)-**3a**.

Methyl (1*S*,3*R*,3a*R*,6a*S*)-3-(2-methoxynaphthalen-1-yl)-5-methyl-4,6-dioxooctahydropyrrolo[3,4-c]pyrrole-1-carboxylate (3b).

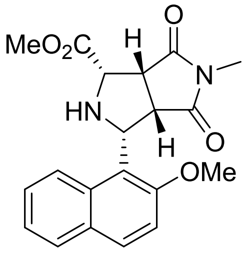

Compound **3b** was prepared following the general procedure from methyl (*E*)-2-(((2-methoxynaphthalen-1-yl)methylene)amino)acetate (**1b**) (103 mg, 0.40 mmol, 1.00 equiv) and *N*-methylmaleimide (44.4 mg, 0.40 mmol, 1.00 equiv). The

residue was purified by silica gel flash chromatography (Heptane-EtOAc 1:1) to afford **3b** (103 mg, 70%, white solid). **^{1}H NMR (300 MHz, CDCl$_3$, δ):** 8.12 (d, *J* = 8.8 Hz, 1H), 7.85 (d, *J* = 9.1 Hz, 1H), 7.80 (dd, *J* = 8.1, 1.4 Hz, 1H), 7.52 (ddd, *J* = 8.6, 6.8, 1.5 Hz, 1H), 7.37 (ddd, *J* = 8.0, 6.8, 1.1 Hz, 1H), 7.30 – 7.25 (m, 1H), 5.32 (d, *J* = 5.5 Hz, 1H), 4.07 – 3.99 (m, 1H), 3.99 – 3.97 (m, 1H), 3.95 (s, 3H), 3.85 (dd, *J* = 9.0, 5.6 Hz, 1H), 3.78 (s, 3H), 3.05 (s, 3H), 1.63 (bs, 1H). **^{13}C NMR (75 MHz, CDCl$_3$, δ):** 177.8, 176.9, 172.1, 154.8, 132.8, 130.9, 129.6, 128.9, 127.6, 124.0, 122.5, 119.3, 112.7, 63.8, 59.4, 56.0, 53.0, 52.0, 51.7, 25.3. **HRMS-ESI (*m/z*):** calcd. for $C_{20}H_{21}N_2O_5$ (M+H$^+$): 369.1445; found: 369.1451.

[α]$_{D\ 20}$: +9.33 (c = 0.15, CH_2Cl_2), 99% *ee*.

HPLC: Chiralpak IG, hexane/^{i}PrOH 87/13 in 30 min, flow rate 1 mL/min (λ = 230 nm) t_R: 6.52 min (1*S*,3*R*,3a*S*,6a*R*)-**3b** and 8.05 min (1*R*,3*S*,3a*R*,6a*S*)-**3b**.

Methyl (1*S*,3*R*,3a*R*,6a*S*)-3-(2-isopropoxynaphthalen-1-yl)-5-methyl-4,6-dioxooctahydropyrrolo[3,4-c]pyrrole-1-carboxylate (3c).

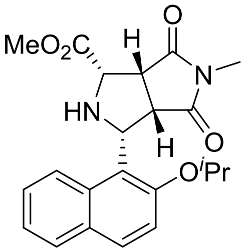

Compound **3c** was prepared following the general procedure from methyl (*E*)-2-(((2-isopropoxynaphthalen-1-yl)methylene)amino)acetate (**1c**) (114 mg, 0.40 mmol, 1.00 equiv) and *N*-methylmaleimide (44.4 mg, 0.40 mmol, 1.00 equiv). The residue was purified by silica gel flash chromatography (Heptane-EtOAc 1:1) to afford **3c** (127 mg, 80%, white solid). **^{1}H NMR (300 MHz, CDCl$_3$, δ):** 7.90 (d, *J* = 8.6 Hz, 1H), 7.81 (d, *J* = 9.3 Hz, 2H), 7.55 – 7.46 (m, 1H), 7.40 – 7.32 (m, 1H), 7.26 – 7.22 (m, 1H), 5.31 – 5.17 (m, 1H), 5.01 – 4.89 (m, 1H), 4.81 (hept, *J* = 6.6 Hz, 1H), 4.20 – 4.06 (m, 1H), 3.89 (s, 3H), 3.69 – 3.61 (m, 1H), 3.57 – 3.46 (m, 1H), 2.90 (s, 3H), 1.41 (d, *J* = 6.0 Hz, 3H), 1.36 (d, *J* = 5.9 Hz, 3H). **^{13}C NMR (75 MHz, CDCl$_3$, δ):** 176.2, 174.9, 170.6, 154.4, 133.0, 130.3, 129.2, 129.1, 127.3, 123.7, 121.5, 115.2, 114.8, 70.5, 63.6, 61.0, 52.5, 51.2, 50.4, 25.3, 22.2, 21.6. **HRMS-ESI (*m/z*):** calcd. for $C_{22}H_{25}N_2O_5$ (M+H$^+$): 397.1758; found: 397.1757.

[α]$_{D\ 20}$: +38.1 (c = 0.30, CH_2Cl_2), 91% *ee*.

SFC: Chiralpak IB, CO_2-MeOH 95:5 to 70-30, flow rate 2 mL/min (λ = 230 nm), t_R: 4.28 min (1*R*,3*S*,3a*R*,6a*S*)-**3c** and 5.27 min (1*S*,3*R*,3a*S*,6a*R*)-**3c**.

Methyl(1*S*,3*R*,3a*R*,6a*S*)-5-methyl-4,6-dioxo-3-(2-(tosyloxy)naphthalen-1-yl)octahydropyrrolo[3,4-c]pyrrole-1-carboxylate (3d).

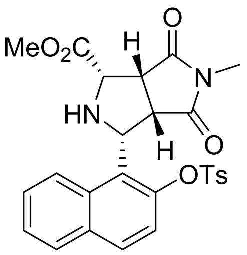

Compound **3d** was prepared following the general procedure from methyl (*E*)-2-(((2-(tosyloxy)naphthalen-1-yl)methylene)amino)acetate (**1d**) (159 mg, 0.40 mmol, 1.00 equiv) and *N*-methylmaleimide (44.4 mg, 0.40 mmol, 1.00 equiv). The residue was purified by silica gel flash chromatography (Heptane-EtOAc 1:1) to afford **3d** (146 mg, 72%, white solid). **^{1}H NMR (300 MHz, CDCl$_3$, δ):** 8.98 (dd, *J* = 8.6, 1.1 Hz, 1H), 7.81 – 7.73 (m, 3H), 7.69 (d, *J* = 8.9 Hz, 1H), 7.43 (ddd, *J* = 8.1, 6.8, 1.3 Hz, 1H), 7.39 – 7.31 (m, 3H), 6.95 (d, *J* = 8.9 Hz, 1H), 5.18 (d, *J* = 9.0 Hz, 1H), 3.99 – 3.94 (m, 1H), 3.92 (s, 3H), 3.64 – 3.57 (m, 1H), 3.55 – 3.48 (m, 1H), 2.72 (s, 3H), 2.46 (s, 3H), 2.06 (bs, 1H). **^{13}C NMR (75 MHz, CDCl$_3$, δ):** 176.4, 174.9, 170.1, 146.5, 146.1, 133.8, 132.7, 130.8,

130.1, 128.0, 129.86, 126.4, 126.2, 124.2, 120.6, 62.2, 58.6, 52.4, 47.4, 47.4, 31.1, 25.3, 21.9. **HRMS-ESI (*m/z*):** calcd. for $C_{26}H_{25}N_2O_7S$ (M+H$^+$): 509.1377; found: 509.1376.

[α]$_{D\ 20}$: +44.8 (c = 0.50, CH_2Cl_2), 97% *ee*.

SFC: Chiralpak IA, CO_2-MeOH 95:5 to 60-40, flow rate 2 mL/min (λ = 230 nm), t_R: 6.48 min (1*R*,3*S*,3a*R*,6a*S*)-**3d** and 7.63 min (1*S*,3*R*,3a*S*,6a*R*)-**3d**.

Methyl (1*S*,3*R*,3a*S*,6a*R*)-5-methyl-4,6-dioxo-3-(2-phenylnaphthalen-1-yl)octahydropyrrolo[3,4-c]pyrrole-1-carboxylate (3e).

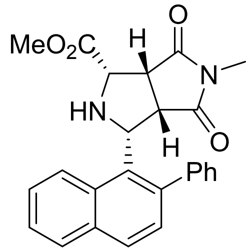

Compound **3e** was prepared following the general procedure from methyl (*E*)-2-(((2-phenylnaphthalen-1-yl)methylene)amino)acetate (**1e**) (121 mg, 0.40 mmol, 1.00 equiv) and *N*-methylmaleimide (44.4 mg, 0.40 mmol, 1.00 equiv). The residue was purified by silica gel flash chromatography (Heptane-EtOAc 2:1) to afford **3e** (126 mg, 76%, white solid). **^{1}H NMR (300 MHz, $CDCl_3$, δ):** 9.03 (d, *J* = 8.6 Hz, 1H), 7.82 (d, *J* = 7.8 Hz, 1H), 7.77 (d, *J* = 8.3 Hz, 1H), 7.47 – 7.28 (m, 8H), 4.75 (d, *J* = 8.7 Hz, 1H), 3.85 (s, 3H), 3.74 (d, *J* = 5.5 Hz, 1H), 3.51 – 3.31 (m, 1H), 3.23 – 3.07 (m, 1H), 2.74 (s, 3H), 2.46 (bs, 1H). **^{13}C NMR (75 MHz, $CDCl_3$, δ):** 176.5, 175.2, 170.2, 142.8, 141.4, 133.8, 132.95, 129.0, 128.6, 128.3, 127.8, 127.5, 126.2, 125.5, 125.4, 77.4, 63.0, 61.7, 52.2, 48.8, 47.7, 25.2. **HRMS-ESI (*m/z*):** calcd. for $C_{25}H_{23}N_2O_4$ (M+H$^+$): 415.1652; found: 415.1651.

[α]$_{D\ 20}$: +31.3 (c = 0.3, CH_2Cl_2), 84% *ee*.

HPLC: Chiralpak IA, hexane/^{i}PrOH 90/10 in 30 min, flow rate 1 mL/min (λ = 230 nm) t_R: 14.88 min (1*S*,3*R*,3a*S*,6a*R*)-**3e** and 18.58 min (1*R*,3*S*,3a*R*,6a*S*)-**3e**.

Methyl (1*S*,3*R*,3a*S*,6a*R*)-3-(2-bromonaphthalen-1-yl)-5-methyl-4,6-dioxooctahydropyrrolo[3,4-c]pyrrole-1-carboxylate (3f). Compound **3f** was prepared

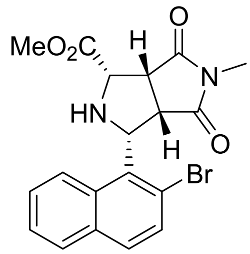

following the general procedure from methyl (*E*)-2-(((2-bromonaphthalen-1-yl)methylene)amino)acetate (**1f**) (122 mg, 0.40 mmol, 1.00 equiv) and *N*-methylmaleimide (44.4 mg, 0.40 mmol, 1.00 equiv). The residue was purified by silica gel flash chromatography (Heptane-EtOAc 2:1) to afford **3f** (107 mg, 64%, white solid). **^{1}H NMR (300 MHz, $CDCl_3$, δ):** 9.16 (d, *J* = 8.7 Hz, 1H), 7.74 (d, *J* = 8.1 Hz, 1H), 7.64 – 7.55 (m, 2H), 7.47 – 7.35 (m, 1H), 7.39 – 7.28 (m, 1H), 5.41 – 5.32 (m, 1H), 4.01 – 3.93 (m, 1H), 3.88 (s, 3H), 3.90 – 3.82 (m, 1H), 3.62 – 3.54 (m, 1H), 2.67 (s, 3H), 2.49 (bs, 1H). **^{13}C NMR (75 MHz, $CDCl_3$, δ):** 176.4, 174.7, 170.2, 134.0, 133.4, 130.4, 130.1, 129.8, 128.9, 126.0, 126.0, 125.2, 123.8, 65.7, 62.0, 52.3, 47.2, 46.1, 25.2. **HRMS-ESI (*m/z*):** calcd. for $C_{19}H_{18}BrN_2O_4$ (M+H$^+$): 417.0444; found: 417.0440.

[α]$_{D\ 20}$: +23.0 (c = 0.15, CH_2Cl_2), 89% *ee*.

HPLC: Chiralpak IC, hexane/^{i}PrOH 60/40 in 30 min, flow rate 1 mL/min (λ = 230 nm) t_R: 10.16 min (1*S*,3*R*,3a*S*,6a*R*)-**3f** and 18.46 min (1*R*,3*S*,3a*R*,6a*S*)-**3f**.

Methyl (1*S*,3*R*,3a*S*,6a*R*)-5-methyl-3-(naphthalen-1-yl)-4,6-dioxooctahydropyrrolo[3,4-c]pyrrole-1-carboxylate (3g).

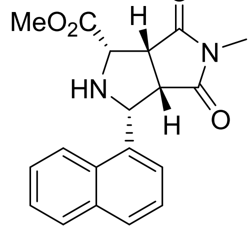

Compound **3g** was prepared following the general procedure from methyl (*E*)-2-((naphthalen-1-ylmethylene)amino)acetate (**1g**) (91.0 mg, 0.40 mmol, 1.00 equiv) and *N*-methylmaleimide

(44.4 mg, 0.40 mmol, 1.00 equiv). The residue was purified by silica gel flash chromatography (Heptane-EtOAc 2:1) to afford **3g** (133 mg, 98%, white solid). **^{1}H NMR (300 MHz, CDCl$_3$, δ):** 7.93 (d, J = 8.1 Hz, 1H), 7.88 (d, J = 8.8 Hz, 1H), 7.79 (d, J = 8.1 Hz, 1H), 7.66 (d, J = 6.8 Hz, 1H), 7.59 – 7.46 (m, 2H), 7.44 – 7.37 (m, 1H), 5.00 (d, J = 7.9 Hz, 1H), 4.01 (d, J = 6.1 Hz, 1H), 3.89 (s, 3H), 3.59 (d, J = 8.0 Hz, 1H), 3.53 (d, J = 6.7 Hz, 1H), 2.75 (s, 3H), 2.29 (bs, 1H). **^{13}C NMR (75 MHz, CDCl$_3$, δ):** 176.0, 174.2, 170.3, 133.4, 133.1, 131.2, 129.2, 128.5, 126.5, 125.7, 125.5, 123.3, 122.4, 59.8, 52.4, 48.1, 25.0. **HRMS-ESI (*m/z*):** calcd. for $C_{19}H_{19}N_2O_4$ (M+H$^+$): 339.1339; found: 339.1336.

$[\alpha]_{D\,20}$: +37.5 (c = 0.15, CH_2Cl_2), 99% *ee*.

HPLC: Chiralpak IC, hexane/^{i}PrOH 20/80 in 15 min, flow rate 1 mL/min (λ = 280 nm) t_R: 8.34 min (1*S*,3*R*,3a*S*,6a*R*)-**3g** and 12.86 min (1*R*,3*S*,3a*R*,6a*S*)-**3g**.

Methyl(1*S*,3*R*,3a*R*,6a*S*)-3-(1-bromonaphthalen-2-yl)-5-methyl-4,6-dioxooctahydropyrrolo[3,4-c]pyrrole-1-carboxylate (11a).

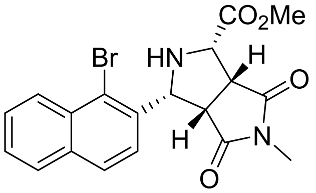

Compound **11a** was prepared following the general procedure from methyl methyl (*E*)-2-(((1-bromonaphthalen-2-yl)methylene)amino)acetate (**10a**) (122 mg, 0.40 mmol, 1.00 equiv) and *N*-methylmaleimide (44.4 mg, 0.40 mmol, 1.00 equiv). The residue was purified by silica gel flash chromatography (Heptane-EtOAc 2:1) to afford **11a** (159 mg, 97%, white solid). **^{1}H NMR (300 MHz, CDCl$_3$, δ):** δ 8.33 (d, J = 8.1 Hz, 1H), 7.80 (dd, J = 8.0, 1.4 Hz, 1H), 7.76 (d, J = 8.6 Hz, 1H), 7.62 (d, J = 8.5 Hz, 1H), 7.59 – 7.48 (m, 2H), 5.00 (d, J = 8.5 Hz, 1H), 4.08 (d, J = 6.8 Hz, 1H), 3.90 (s, 3H), 3.85 – 3.74 (m, 1H), 3.63 – 3.54 (m, 1H), 2.82 (s, 3H), 2.40 (bs, 1H). **^{13}C NMR (75 MHz, CDCl$_3$, δ):** 176.1, 174.5, 170.2, 134.6, 134.4, 132.2, 128.4, 128.0, 127.7, 127.4, 126.8, 124.7, 124.0, 63.6, 61.4, 52.4, 47.5, 46.5, 25.1. **HRMS-ESI (*m/z*):** calcd. for $C_{19}H_{18}BrN_2O_4$ (M+H$^+$): 418.0444; found: 418.0451.

$[\alpha]_{D\,20}$: +21.4 (c = 0.10, CH_2Cl_2), 99% *ee*.

SFC: Chiralpak IB, CO_2-MeOH 95:5 to 70-30, flow rate 2 mL/min (λ = 230 nm), t_R: 5.52 min (1*S*,3*R*,3a*S*,6a*R*)-**11a** and 5.65 min (1*R*,3*S*,3a*R*,6a*S*)-**11a**.

Methyl(1*S*,3*R*,3a*R*,6a*S*)-3-(1-iodonaphthalen-2-yl)-5-methyl-4,6-dioxooctahydropyrrolo[3,4-c]pyrrole-1-carboxylate (11b).

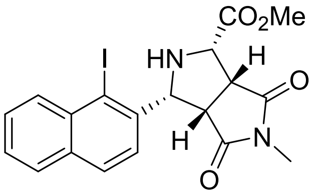

Compound **11b** was prepared following the general procedure from methyl (*E*)-2-(((1-iodonaphthalen-2-yl)methylene)amino)acetate (**10b**) (141 mg, 0.40 mmol, 1.00 equiv) and *N*-methylmaleimide (44.4 mg, 0.40 mmol, 1.00 equiv). The residue was purified by silica gel flash chromatography (Heptane-EtOAc 2:1) to afford **11b** (176 mg, 95%, yellow solid). **^{1}H NMR (300 MHz, CDCl$_3$, δ):** δ 8.23 (d, J = 8.4 Hz, 1H), 7.74 – 7.70 (m, 2H), 7.56 – 7.51 (m, 2H), 7.50 – 7.45 (m, 1H), 4.84 (d, J = 8.7 Hz, 1H), 3.97 (d, J = 6.9 Hz, 1H), 3.87 (s, 3H), 3.82 – 3.75 (m, 1H), 3.55 – 3.44 (m, 1H), 2.77 (s, 3H), 2.34 (bs, 1H). **^{13}C NMR (75 MHz, CDCl$_3$, δ):** 176.0, 174.3, 170.2, 138.8, 134.6, 134.2, 132.7, 128.9, 128.6, 127.9, 126.8, 125.2, 105.6, 68.7, 61.1, 52.3, 47.3, 46.2, 25.0. **HRMS-ESI (*m/z*):** calcd. for $C_{19}H_{18}IN_2O_4$ (M+H$^+$): 465.0306; found: 465.0305.

[α]$_{D\ 20}$: +123.2 (c = 0.10, CH_2Cl_2), 99% *ee*.

HPLC: Chiralpak IC, hexane/^{i}PrOH 20/80 in 30 min (λ = 254 nm), t_R: 11.57 min (1*S*,3*R*,3a*S*,6a*R*)-**11b** and 18.73 min (1*R*,3*S*,3a*R*,6a*S*)-**11b**.

Methyl (1*S*,3*R*,3a*S*,6a*R*)-3-(1-ethylnaphthalen-2-yl)-5-methyl-4,6-dioxooctahydropyrrolo[3,4-c]pyrrole-1-carboxylate (11c).

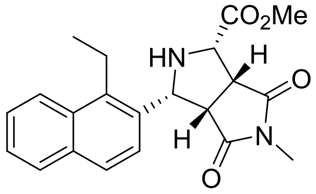

Compound **11c** was prepared following the general procedure from methyl (*E*)-2-(((1-ethylnaphthalen-2-yl)methylene)amino)acetate (**10c**) (102 mg, 0.40 mmol, 1.00 equiv) and *N*-methylmaleimide (44.4 mg, 0.40 mmol, 1.00 equiv). The residue was purified by silica gel flash chromatography (Heptane-EtOAc 2:1) to afford **11c** (144 mg, 98%, white solid). **^{1}H NMR (300 MHz, $CDCl_3$, δ):** δ 8.08 (d, *J* = 8.5 Hz, 1H), 7.80 (dd, *J* = 8.1, 1.5 Hz, 1H), 7.66 (d, *J* = 8.7 Hz, 1H), 7.57 (d, *J* = 8.7 Hz, 1H), 7.51 (ddd, *J* = 8.5, 6.8, 1.5 Hz, 1H), 7.45 (ddd, *J* = 8.0, 6.7, 1.2 Hz, 1H), 4.83 (dd, *J* = 8.7, 4.5 Hz, 1H), 4.05 (dd, *J* = 6.7, 4.2 Hz, 1H), 3.90 (s, 3H), 3.63 – 3.53 (m, 1H), 3.51 – 3.43 (m, 1H), 3.31 – 3.18 (m, 1H), 3.17 – 3.03 (m, 1H), 2.84 (s, 3H), 2.36 (bs, 1H), 1.38 (t, *J* = 7.6 Hz, 3H). **^{13}C NMR (75 MHz, $CDCl_3$, δ):** 176.2, 174.6, 170.3, 137.7, 133.6, 131.7, 131.1, 129.0, 126.7, 126.2, 125.6, 124.2, 123.8, 61.6, 60.4, 52.4, 48.3, 48.2, 25.1, 21.6, 15.5. **HRMS-ESI (*m/z*):** calcd. for $C_{21}H_{23}N_2O_4$ ($M+H^+$): 367.1652; found: 367.1652.

[α]$_{D\ 20}$: -75.4 (c = 0.10, CH_2Cl_2), 99% *ee*.

HPLC: Chiralpak IC, hexane/^{i}PrOH 20/80 in 30 min (λ = 230 nm), t_R: 8.81 min (1*S*,3*R*,3a*S*,6a*R*)-**11c** and 15.00 min (1*R*,3*S*,3a*R*,6a*S*)-**11c**.

Methyl (1*S*,3*R*,3a*S*,6a*R*)-3-(1-(4-methoxyphenyl)naphthalen-2-yl)-5-methyl-4,6-dioxooctahydropyrrolo[3,4-c]pyrrole-1-carboxylate (11d).

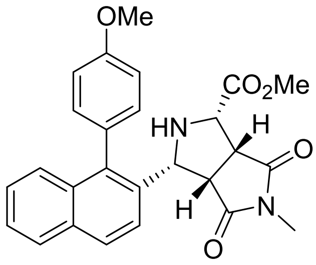

Compound **11d** was prepared following the general procedure from methyl methyl (*E*)-2-(((1-(4-methoxyphenyl)naphthalen-2-yl)methylene)amino)acetate (**10d**) (133 mg, 0.40 mmol, 1.00 equiv) and *N*-methylmaleimide (44.4 mg, 0.40 mmol, 1.00 equiv). The residue was purified by silica gel flash chromatography (Heptane-EtOAc 2:1) to afford **11d** (164 mg, 92%, white solid). **^{1}H NMR (300 MHz, $CDCl_3$, δ):** δ 7.87 – 7.77 (m, 2H), 7.63 (d, *J* = 8.7 Hz, 1H), 7.55 (d, *J* = 7.3 Hz, 1H), 7.46 – 7.27 (m, 3H), 7.21 (d, *J* = 7.3 Hz, 1H), 7.04 (d, *J* = 8.8 Hz, 2H), 4.30 (d, *J* = 8.7 Hz, 1H), 3.90 (s, 3H), 3.85 (s, 3H), 3.80 (dd, *J* = 6.6, 1.7 Hz, 1H), 3.37 – 3.27 (m, 1H), 3.09 – 2.95 (m, 1H), 2.85 (s, 3H), 2.58 (bs, 1H). **^{13}C NMR (126 MHz, $CDCl_3$, δ):** 176.2, 175.1, 170.3, 159.2, 138.5, 133.2, 133.1, 132.5, 131.9, 130.9, 130.5, 128.0, 127.8, 126.7, 126.0, 125.8, 123.2, 114.0, 113.8, 61.3, 55.4, 52.3, 48.7, 48.1, 25.0.

[α]$_{D\ 20}$: -93.4 (c = 0.10, CH_2Cl_2), 99% *ee*.

HPLC: Chiralpak IA, hexane/^{i}PrOH 80/20 in 30 min (λ = 230 nm), t_R: 14.89 min (1*S*,3*R*,3a*S*,6a*R*)-**11d** and 19.68 min (1*R*,3*S*,3a*R*,6a*S*)-**11d**.

Methyl (1*S*,3*R*,3a*S*,6a*R*)-3-(1-methoxynaphthalen-2-yl)-5-methyl-4,6-dioxooctahydropyrrolo[3,4-c]pyrrole-1-carboxylate (11e).

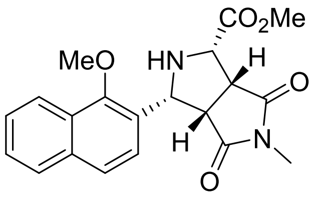

Compound **11e** was prepared following the general procedure

from methyl (*E*)-2-(((1-methoxynaphthalen-2-yl)methylene)amino)acetate (**10e**) (103 mg, 0.40 mmol, 1.00 equiv) and *N*-methylmaleimide (44.4 mg, 0.40 mmol, 1.00 equiv). The residue was purified by silica gel flash chromatography (Heptane-EtOAc 1:1) to afford **11e** (125 mg, 85%, white solid). **^{1}H NMR (300 MHz, $CDCl_3$, δ):** 8.05 (d, *J* = 7.8 Hz, 1H), 7.79 (d, *J* = 7.4 Hz, 1H), 7.58 – 7.37 (m, 4H), 4.81 (d, *J* = 5.6 Hz, 1H), 4.00 (s, 3H), 3.88 (d, *J* = 4.8 Hz, 1H), 3.83 (s, 3H), 3.40 (d, *J* = 6.9 Hz, 2H), 2.76 (s, 3H), 2.34 (bs, 1H). **^{13}C NMR (75 MHz, $CDCl_3$, δ):** 176.0, 174.8, 170.2, 153.7, 134.5, 128.2, 127.2, 126.0, 125.9, 125.4, 124.1, 124.0, 122.0, 62.5, 61.4, 57.8, 52.0, 48.2, 48.0, 24.7. **HRMS-ESI (*m/z*):** calcd. for $C_{20}H_{21}N_2O_5$ (M+H$^+$): 369.1445; found: 369.1450.

$[\alpha]_{D\ 20}$: +235.1 (c = 1.00, $CHCl_3$), 95% *ee*.

SFC: Chiralpak IB, CO_2-MeOH 95:5 to 60-40, flow rate 2 mL/min (λ = 265 nm), t_R: 4.79 min (1*R*,3*S*,3a*R*,6a*S*)-**11e** and 5.59 min (1*S*,3*R*,3a*S*,6a*R*)-**11e**.

Methyl (1*R*,3*S*,3a*R*,6a*S*)-3-(1-isopropoxynaphthalen-2-yl)-5-methyl-4,6-dioxooctahydropyrrolo[3,4-c]pyrrole-1-carboxylate (11f).

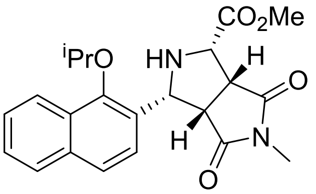

Compound **11f** was prepared following the general procedure from methyl methyl (*E*)-2-(((1-isopropoxynaphthalen-2-yl)methylene)amino)acetate (**10f**) (114 mg, 0.40 mmol, 1.00 equiv) and *N*-methylmaleimide (44.4 mg, 0.40 mmol, 1.00 equiv). The residue was purified by silica gel flash chromatography (Heptane-EtOAc 1:1) to afford **11f** (146 mg, 92%, white solid). **^{1}H NMR (300 MHz, $CDCl_3$, δ):** 8.12 – 8.05 (m, 1H), 7.83 – 7.77 (m, 1H), 7.55 (d, *J* = 8.6 Hz, 1H), 7.51 – 7.41 (m, 2H), 7.35 (d, *J* = 8.6 Hz, 1H), 5.05 (d, *J* = 8.4 Hz, 1H), 4.56 (hept, *J* = 5.9 Hz, 1H), 4.09 (d, *J* = 6.6 Hz, 1H), 3.89 (s, 3H), 3.64 – 3.58 (m, 1H), 3.57 – 3.50 (m, 1H), 2.91 (s, 3H), 2.35 (bs, 1H), 1.50 (d, *J* = 6.1 Hz, 3H), 1.33 (d, *J* = 6.1 Hz, 3H). **^{13}C NMR (75 MHz, $CDCl_3$, δ):** 176.2, 175.0, 170.3, 151.8, 135.0, 134.9, 128.5, 128.3, 126.2, 125.8, 125.7, 124.6, 123.8, 123.1, 61.8, 58.2, 52.4, 48.6, 48.5, 25.1, 23.3, 22.5. **HRMS-ESI (*m/z*):** calcd. for $C_{22}H_{25}N_2O_5$ (M+H$^+$): 397.1758; found: 397.1762.

$[\alpha]_{D\ 20}$: +28.9 (c = 0.20, CH_2Cl_2), 98% *ee*.

HPLC: Chiralpak IC, hexane/^{i}PrOH 50/50 in 30 min (λ = 230 nm) t_R: 6.68 min (1*R*,3*S*,3a*R*,6a*S*)-**11f** and 10.93 min (1*S*,3*R*,3a*S*,6a*R*)-**11f**.

Methyl (1*S*,3*R*,3a*S*,6a*R*)-3-(2-methylnaphthalen-1-yl)-4,6-dioxo-5-phenyloctahydropyrrolo[3,4-c]pyrrole-1-carboxylate (15a).

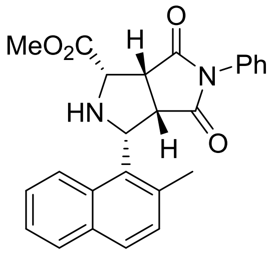

Compound **15a** was prepared following the general procedure from methyl (*E*)-2-(((2-methylnaphthalen-1-yl)methylene)amino)acetate (**1a**) (96.5 mg, 0.40 mmol, 1.00 equiv) and *N*-phenylmaleimide (69.3 mg, 0.40 mmol, 1.00 equiv). The residue was purified by silica gel flash chromatography (Heptane-EtOAc 2:1) to afford **15a** (134 mg, 81%, white solid). **^{1}H NMR (300 MHz, $CDCl_3$, δ):** 9.24 (d, *J* = 8.6 Hz, 1H), 7.63 (dd, *J* = 7.9, 1.7 Hz, 1H), 7.55 (d, *J* = 8.3 Hz, 1H), 7.27 – 7.18 (m, 3H), 7.15 – 7.10 (m, 2H), 7.09 – 7.05 (m, 1H), 6.90 (d, *J* = 7.3 Hz, 2H), 4.87 (d, *J* = 9.0 Hz, 1H), 3.77 (s, 3H), 3.75 – 3.73 (m, 1H), 3.55 – 3.51 (m, 1H), 3.38 – 3.32 (m, 1H), 2.38 (s, 3H), 2.27 (bs, 1H). **^{13}C NMR (75 MHz, $CDCl_3$, δ):** 175.6, 173.5, 170.2, 134.6, 133.3, 133.2, 131.8, 129.2, 129.1, 128.8, 128.8, 128.2, 126.5, 126.0, 125.9, 125.7, 124.7, 62.4, 62.3, 52.3, 47.5, 46.4, 21.2. Minor impurities of a non-identified compound remain in the

analysed product. **HRMS-ESI (*m/z*):** calcd. for $C_{25}H_{23}N_2O_4$ ($M+H^+$): 415.1652; found: 415.1654.

[α]$_{D\ 20}$: +22.9 (c = 0.05, CH_2Cl_2), 99% *ee*.

HPLC: Chiralpak IC, hexane/^{i}PrOH 20/80 in 30 min (λ = 280 nm), t_R: 8.22 min (1*S*,3*R*,3a*S*,6a*R*)-**15a** and 11.99 min (1*R*,3*S*,3a*R*,6a*S*)-**15a**.

Trimethyl (2*S*,3*S*,4*S*,5*R*)-5-(2-methylnaphthalen-1-yl)pyrrolidine-2,3,4-tricarboxylate (15b). Compound **15b** was prepared following the general procedure from methyl (*E*)-2-(((2-methylnaphthalen-1-yl)methylene)amino)acetate (**1a**) (96.5 mg, 0.40 mmol, 1.00 equiv) and dimethyl fumarate (57.7 mg, 0.40 mmol, 1.00 equiv). The residue was purified by silica gel flash chromatography (Heptane-EtOAc 2:1) to afford **15b** (143 mg, 93%, white solid). **^{1}H NMR (300 MHz, CDCl$_3$, δ):** 8.61 (d, *J* = 8.5 Hz, 1H), 7.79 (d, *J* = 8.0 Hz, 1H), 7.67 (d, *J* = 8.4 Hz, 1H), 7.54 – 7.45 (m, 1H), 7.44 – 7.36 (m, 1H), 7.24 (d, *J* = 8.4 Hz, 1H), 5.14 (d, *J* = 9.8 Hz, 1H), 4.32 (d, *J* = 8.9 Hz, 1H), 4.03 (dd, *J* = 9.7, 7.9 Hz, 1H), 3.97 – 3.83 (m, 1H), 3.75 (s, 3H), 3.74 (s, 3H), 3.43 (s, 3H), 3.03 (bs, 1H), 2.54 (s, 3H). **^{13}C NMR (75 MHz, CDCl$_3$, δ):** 173.1, 172.4, 171.4, 135.5, 133.2, 131.4, 129.7, 129.5, 129.0, 128.7, 125.9, 124.6, 124.3, 62.4, 61.9, 52.4, 52.2, 52.1, 51.4, 51.0, 21.1. **HRMS-ESI (*m/z*):** calcd. for $C_{21}H_{24}NO_6$ ($M+H^+$):386.3221; found:386.3223.

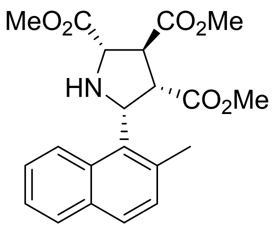

[α]$_{D\ 20}$: +36.2 (c = 0.40, CH_2Cl_2), 88% *ee*.

SFC: Chiralpak IB, CO_2-MeOH 80:20, flow rate 1 mL/min (λ = 265 nm), t_R: 11.62 min (2*R*,3*R*,4*R*,5*S*)-**15b** and 14.78 min (2*S*,3*S*,4*S*,5*R*)-**15b.**

3,4-Di-*tert*-butyl 2-methyl (2*S*,3*S*,4*S*,5*R*)-5-(2-methylnaphthalen-1-yl)pyrrolidine-2,3,4-tricarboxylate (15c). Compound **15c** was prepared following the general procedure from methyl (*E*)-2-(((2-methylnaphthalen-1-yl)methylene)amino)acetate (**1a**) (96.5 mg, 0.40 mmol, 1.00 equiv) and di-tert-butyl fumarate (91.3 mg, 0.40 mmol, 1.00 equiv). The residue was purified by silica gel flash chromatography (Heptane-EtOAc 10:1) to afford **15b** (169 mg, 90%, white solid). **^{1}H NMR (300 MHz, CDCl$_3$, δ):** 8.58 (d, *J* = 8.7 Hz, 1H), 7.81 (dd, *J* = 8.2, 1.5 Hz, 1H), 7.68 (d, *J* = 8.4 Hz, 1H), 7.48 (ddd, *J* = 8.5, 6.8, 1.5 Hz, 1H), 7.41 (ddd, *J* = 7.9, 6.7, 1.1 Hz, 1H), 7.26 (d, *J* = 8.4 Hz, 1H), 5.13 (d, *J* = 10.1 Hz, 1H), 4.28 (d, *J* = 9.1 Hz, 1H), 3.83 (dd, *J* = 10.1, 7.9 Hz, 1H), 3.80 (s, 3H), 3.70 (dd, *J* = 9.1, 7.8 Hz, 1H), 3.02 (bs, 1H), 2.61 (s, 3H), 1.50 (s, 9H), 1.20 (s, 9H). **^{13}C NMR (75 MHz, CDCl$_3$, δ):** 171.8, 171.7, 171.4, 135.6, 133.3, 131.7, 130.2, 129.6, 129.0, 128.6, 126.0, 124.6, 124.6, 81.7, 81.1, 62.2, 61.8, 53.3, 52.5, 52.1, 28.0, 27.8, 21.5. **HRMS-ESI (*m/z*):** calcd. for $C_{27}H_{36}NO_6$ ($M+H^+$): 470.2537; found: 470.2535.

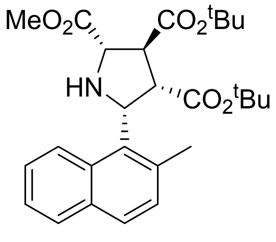

[α]$_{D\ 20}$: -33.8 (c = 0.40, CH_2Cl_2), 81% *ee*.

HPLC: Chiralpak IC, hexane/^{i}PrOH 80/20 in 50 min (λ = 280 nm), t_R: 23.80 min (2*S*,3*S*,4*S*,5*R*)-**15b** and 27.20 min (2*S*,3*S*,4*S*,5*R*)-**15b.**

Methyl (2*S*,3*S*,4*S*,5*R*)-3,4-dicyano-5-(2-methylnaphthalen-1-yl)pyrrolidine-2-carboxylate (15d). Compound **15d** was prepared following the general procedure from methyl (*E*)-2-(((2-methylnaphthalen-1-

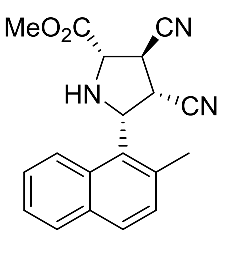

yl)methylene)amino)acetate (**1a**) (96.5 mg, 0.40 mmol, 1.00 equiv) and fumaronitrile (31.2 mg, 0.40 mmol, 1.00 equiv). The residue was purified by silica gel flash chromatography (Heptane-EtOAc 2:1) to afford **15d** (97.1 mg, 76%, white solid). **^{1}H NMR (300 MHz, $CDCl_3$, δ):** 8.53 (d, J = 8.6 Hz, 1H), 7.87 (d, J = 7.7 Hz, 1H), 7.78 (d, J = 8.4 Hz, 1H), 7.62 – 7.55 (m, 1H), 7.53 – 7.42 (m, 1H), 7.33 (d, J = 8.4 Hz, 1H), 5.23 (d, J = 8.9 Hz, 1H), 4.35 (d, J = 6.8 Hz, 1H), 3.94 (s, 3H), 3.91 – 3.88 (m, 1H), 3.86 – 3.80 (m, 1H), 2.84 (bs, 1H), 2.68 (s, 3H). **^{13}C NMR (75 MHz, $CDCl_3$, δ):** 168.8, 136.6, 133.6, 131.0, 130.3, 129.8, 129.7, 127.2, 126.0, 125.4, 123.3, 117.8, 117.2, 63.23, 62.6, 53.4, 37.5, 37.0, 21.6. **HRMS-ESI (*m/z*):** calcd. for $C_{19}H_{18}N_3O_2$ (M+H$^+$): 320.1394; found: 320.1390.

$[\alpha]_{D\ 20}$: +22.9 (c = 0.05, CH_2Cl_2), 98% *ee*.

HPLC: Chiralpak IC, Hexane/^{i}PrOH 75/25 in 40 min, flow rate 0.9 mL/min (λ = 230 nm) t_R: 20.88 min (2*S*,3*S*,4*S*,5*R*)-**15d** and 12.63 min (2*R*,3*R*,4*R*,5*S*)-**15d**.

5. Blue light promoted aromatization of pyrrolidines

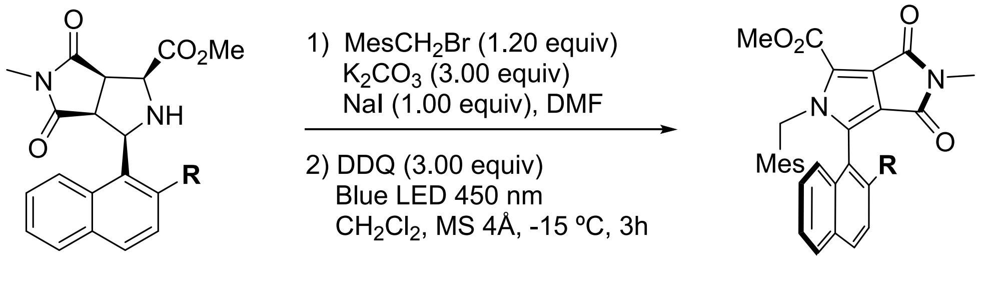

3a-g, 11a-e, 15a-d **6a, 9a-g, 13a-f, 17a-d**

Step 1. *N*-alkylation of pyrrolidines with 2-bromomethyl-1,3,5-trimethylbenzene. A solution of the corresponding pyrrolidine (0.37 mmol, 1.00 equiv), 2-(bromomethyl)-1,2,5-trimethylbenzene (94.3 mg, 0.44 mmol, 1.20 equiv), sodium carbonate (153 mg, 1.11 mmol, 3.00 equiv) and sodium iodine (55.3 mg, 0.37 mmol, 1.00 equiv) in dimethylformamide (3.80 mL) were stirred for 16 h at room temperature. The solution was dissolved with *tert*-butyl methyl ether (10.0 mL), washed with brine, and the volatiles were removed in vacuo affording the corresponding *N*-alkylated pyrrolidine which was used in next step without further purification.

Step 2. Blue light promoted aromatization of pyrrolidines. A solution of the corresponding *N*-alkylated pyrrolidine (0.21 mmol, 1.00 equiv) and 2,3-dichloro-5,6-dicyano-1,4-benzoquinone (145 mg, 0.64 mmol, 3.00 equiv) in dry dichloromethane (6.00 mL) were stirred at -15 ºC under blue light for 3 h. The solution was dissolved with dichloromethane (10.0 mL), washed with $NaHCO_3$(sat) and concentrated under reduced pressure. The residue was purified by silica gel flash chromatography to afford the corresponding pyrrole.

(*R*)-Methyl 5-methyl-3-(2-methylnaphthalen-1-yl)-4,6-dioxo-2-(2,4,6-trimethylbenzyl)-2,4,5,6-tetrahydropyrrolo[3,4-c]pyrrole-1-carboxylate (9a). Compound **9a** was prepared following the

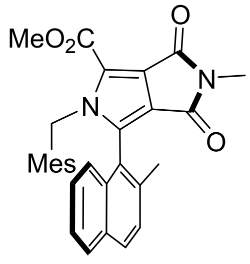

general procedure from methyl methyl(1*S*,3*R*,3a*R*,6a*S*)-5-methyl-3-(2-methylnaphthalen-1-yl)-4,6-dioxooctahydropyrrolo[3,4-c]pyrrole-1-carboxylate (**3a**) (102 mg, 0.21 mmol, 1.00 equiv). The residue was purified by silica gel flash chromatography (Heptane-EtOAc 6:1) to afford **9a** (98.9 mg, 98%, yellow solid). **^{1}H NMR (300 MHz, CDCl$_3$, δ):** 7.81 – 7.75 (m, 1H), 7.72 (d, *J* = 8.4 Hz, 1H), 7.43 – 7.31 (m, 2H), 7.14 – 7.06 (m, 2H), 6.36 (s, 2H), 6.09 (d, *J* = 15.8 Hz, 1H), 5.17 (d, *J* = 15.8 Hz, 1H), 4.05 (s, 3H), 3.04 (s, 3H), 2.06 (s, 3H), 1.89 (s, 3H), 1.43 (s, 6H). **^{13}C NMR (75 MHz, CDCl$_3$, δ):** 163.5, 163.3, 161.0, 137.5, 136.9, 136.7, 135.2, 132.3, 131.9, 129.8, 129.8, 129.3, 128.5, 128.4, 127.3, 125.3, 124.9, 124.0, 123.7, 121.2, 120.8, 52.7, 45.9, 24.3, 20.7, 20.0, 19.3. **HRMS-ESI (*m/z*):** calcd. for $C_{30}H_{29}N_2O_4$ (M+H$^+$): 481.2122; found: 481.2124.

[α]$_{D\ 20}$: +29.9 (c = 0.30, CHCl$_3$), 84% *ee*.

HPLC: Chiralpak IA, hexane/^{i}PrOH 97/3 in 60 min, flow rate 0.20 mL/min (λ = 230 nm) t$_R$: 38.40 min (*R*)-**9a** and 41.63 min (*S*)-**9a**.

(*S*)-Methyl-3-(2-methoxynaphthalen-1-yl)-5-methyl-4,6-dioxo-2-(2,4,6-trimethylbenzyl)-2,4,5,6-tetrahydropyrrolo[3,4-c]pyrrole-1-carboxylate (9b). Compound **9b** was prepared following the general procedure from methyl (1*S*,3*R*,3a*R*,6a*S*)-3-(2-methoxynaphthalen-1-yl)-5-methyl-4,6-dioxooctahydropyrrolo[3,4-c]pyrrole-1-carboxylate (**3b**) (105 mg, 0.21 mmol, 1.00 equiv). The residue was purified by silica gel flash chromatography (Heptane-EtOAc 5:1) to afford **9b** (72.0 mg, 69%, yellow solid). **^{1}H NMR (300 MHz, CDCl$_3$, δ):** 7.79 (d, *J* = 9.1 Hz, 1H), 7.72 (d, *J* = 7.5 Hz, 1H), 7.34 – 7.27 (m, 2H), 7.16 (d, *J* = 8.1 Hz, 1H), 6.96 (d, *J* = 9.1 Hz, 1H), 6.30 (s, 2H), 5.89 (d, *J* = 15.8 Hz, 1H), 5.38 (d, *J* = 15.8 Hz, 1H), 4.01 (s, 3H), 3.61 (s, 3H), 3.03 (s, 3H), 2.00 (s, 3H), 1.54 (s, 6H). **^{13}C NMR (75 MHz, CDCl$_3$, δ):** 163.6, 163.5, 161.0, 155.5, 136.5, 136.2, 132.9, 132.5, 131.8, 129.6, 128.9, 128.6, 128.3, 127.6, 125.1, 123.8, 123.5, 121.4, 120.5, 112.1, 110.8, 55.9, 52.5, 46.1, 24.2, 20.7, 19.4. **HRMS-ESI (*m/z*):** calcd. for $C_{30}H_{29}N_2O_5$ (M+H$^+$): 497.2053; found: 497.2061.

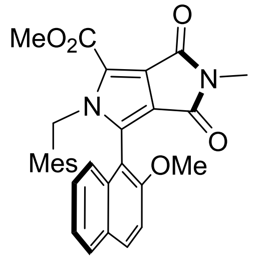

[α]$_{D\ 20}$: +19.4 (c = 0.30, CHCl$_3$), 92% *ee*.

SFC: Chiralpak IB, CO_2-MeOH 95:5 to 70-30, flow rate 2 mL/min (λ = 230 nm), t$_R$: 3.08 min (*R*)-**9b** and 4.45 min (*S*)-**9b**.

(*S*)-Methyl-3-(2-isopropoxynaphthalen-1-yl)-5-methyl-4,6-dioxo-2-(2,4,6-trimethylbenzyl)-2,4,5,6-tetrahydropyrrolo[3,4-c]pyrrole-1-carboxylate (9c). Compound **9c** was prepared following the general procedure from methyl (1*S*,3*R*,3a*R*,6a*S*)-3-(2-isopropoxynaphthalen-1-yl)-5-methyl-4,6-dioxooctahydropyrrolo[3,4-c]pyrrole-1-carboxylate (**3c**) (111 mg, 0.21 mmol, 1.00 equiv). The residue was purified by silica gel flash chromatography (Heptane-EtOAc 5:1) to afford **9c** (85.9 mg, 78%, yellow solid). **^{1}H NMR (300 MHz, CDCl$_3$, δ):** 7.76 (d, *J* = 9.2 Hz, 1H), 7.72 – 7.67 (m, 1H), 7.34 – 7.27 (m, 2H), 7.22 – 7.16 (m, 1H), 7.03 (d, *J* = 9.1 Hz, 1H), 6.27 (s, 2H), 5.68 (d, *J* = 15.9 Hz, 1H), 5.60 (d, *J* = 15.7 Hz, 1H), 4.51 – 4.37 (m, 1H), 3.98 (s, 3H), 3.03 (s, 3H), 1.95 (s, 3H), 1.64 (s, 6H), 1.27 (d, *J* = 6.1 Hz, 3H), 1.10 (d, *J* = 6.0 Hz, 3H). **^{13}C NMR (75 MHz, CDCl$_3$, δ):** 163.7, 163.5, 160.9, 154.2, 136.4,

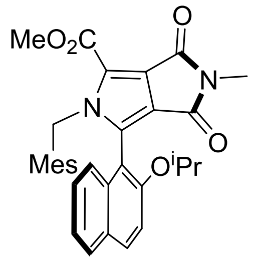

136.2, 133.0, 132.9, 131.7, 129.8, 129.1, 128.8, 128.2, 127.3, 124.9, 123.9, 123.7, 120.8, 120.5, 115.3, 112.7, 71.8, 52.5, 46.3, 24.2, 22.7, 22.2, 20.6, 19.6. **HRMS-ESI (*m/z*):** calcd. for $C_{32}H_{33}N_2O_5$ (M+H$^+$): 525.2384; found: 525.2402.

[α]$_{D\,20}$: -5.67 (c = 0.15, CH_2Cl_2), 85% *ee*.

HPLC: Chiralpak IG, hexane/^{i}PrOH 95/5 in 25 min, flow rate 1 mL/min (λ = 250 nm) t_R: 9.85 min (*S*)-**9c** and 17.95 min (*R*)-**9c**.

(*S*)-Methyl-5-methyl-4,6-dioxo-3-(2-(tosyloxy)naphthalen-1-yl)-2-(2,4,6-trimethylbenzyl)-2,4,5,6-tetrahydropyrrolo[3,4-c]pyrrole-1-carboxylate (9d). Compound **9d** was prepared following the general procedure from methyl(1*S*,3*R*,3a*R*,6a*S*)-5-methyl-4,6-dioxo-3-(2-(tosyloxy)naphthalen-1-yl)octahydropyrrolo[3,4-c]pyrrole-1-carboxylate (**3d**) (135 mg, 0.21 mmol, 1.00 equiv). The residue was purified by silica gel flash chromatography (Heptane-EtOAc 5:1) to afford **9d** (89.6 mg, 67%, yellow solid). **^{1}H NMR (300 MHz, $CDCl_3$, δ):** 7.86 (d, *J* = 9.1 Hz, 1H), 7.71 (d, *J* = 8.1 Hz, 1H), 7.55 (d, *J* = 9.0 Hz, 1H), 7.45 (d, *J* = 8.3 Hz, 2H), 7.38 (ddd, *J* = 8.1, 6.3, 1.7 Hz, 1H), 7.25 – 7.17 (m, 2H), 7.08 (d, *J* = 8.1 Hz, 2H), 6.17 (d, *J* = 15.9 Hz, 1H), 6.12 (s, 2H), 5.23 (d, *J* = 15.8 Hz, 1H), 3.99 (s, 3H), 2.98 (s, 3H), 2.34 (s, 3H), 1.76 (s, 9H). **^{13}C NMR (75 MHz, $CDCl_3$, δ):** 162.8, 162.3, 160.4, 145.7, 145.6, 136.6, 136.3, 133.1, 132.0, 131.8, 131.3, 129.7, 129.5, 129.2, 128.8, 128.1, 127.9, 127.4, 126.5, 125.1, 124.1, 121.8, 121.3, 118.3, 52.7, 46.6, 24.2, 21.7, 20.3, 19.8. **HRMS-ESI (*m/z*):** calcd. for $C_{36}H_{33}N_2O_7S$ (M+H$^+$): 637.2003; found: 637.1993.

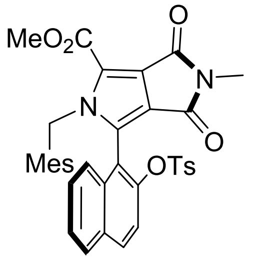

[α]$_{D\,20}$: -24.6 (c = 0.06, CH_2Cl_2), 97% *ee*.

HPLC: Chiralpak IA, hexane/^{i}PrOH 95/5 in 35 min, flow rate 1 mL/min (λ = 230 nm) t_R: 12.06 min (*S*)-**9d** and 20.42 min (*R*)-**9d**.

(*S*)-Methyl 5-methyl-4,6-dioxo-3-(2-phenylnaphthalen-1-yl)-2-(2,4,6-trimethylbenzyl)-2,4,5,6-tetrahydropyrrolo[3,4-c]pyrrole-1-carboxylate (9e). Compound **9e** was prepared following the general procedure from methyl (1*S*,3*R*,3a*S*,6a*R*)-5-methyl-4,6-dioxo-3-(2-phenylnaphthalen-1-yl)octahydropyrrolo[3,4-c]pyrrole-1-carboxylate (**3e**) (115 mg, 0.21 mmol, 1.00 equiv). The residue was purified by silica gel flash chromatography (Heptane-EtOAc 6:1) to afford **9e** (94.6 mg, 83%, yellow solid). **^{1}H NMR (300 MHz, $CDCl_3$, δ):** 7.99 (d, *J* = 8.5 Hz, 1H), 7.83 (d, *J* = 8.1 Hz, 1H), 7.51 (d, *J* = 8.5 Hz, 1H), 7.44 (ddd, *J* = 8.1, 6.2, 1.8 Hz, 1H), 7.34 – 7.27 (m, 5H), 7.16 – 7.11 (m, 2H), 6.33 (s, 2H), 5.20 (d, *J* = 15.7 Hz, 1H), 4.94 (d, *J* = 15.8 Hz, 1H), 3.81 (s, 3H), 3.08 (s, 3H), 2.00 (s, 3H), 1.57 (s, 6H). **^{13}C NMR (75 MHz, $CDCl_3$, δ):** 163.7, 163.4, 160.3, 141.3, 140.4, 136.9, 136.8, 134.3, 132.7, 132.3, 130.9, 129.5, 129.3, 129.2, 128.4, 128.2, 128.0, 127.7, 127.4, 126.1, 125.1, 124.1, 123.3, 122.6, 120.9, 52.4, 45.9, 24.4, 20.7, 19.7. **HRMS-ESI (*m/z*):** calcd. for $C_{35}H_{31}N_2O_4$ (M+H$^+$): 543.2278; found: 543.2294.

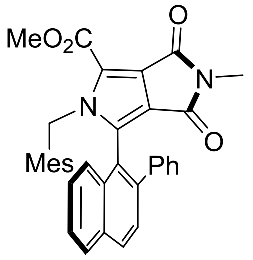

[α]$_{D\,20}$: +3.57 (c = 0.03, CH_2Cl_2), 70% *ee.*

HPLC: Chiralpak IG, hexane/^{i}PrOH 95/5 in 25 min, flow rate 1 mL/min (λ = 250 nm) t_R: 15.64 min (*R*)-**9e** and 19.62 min (*S*)-**9e**.

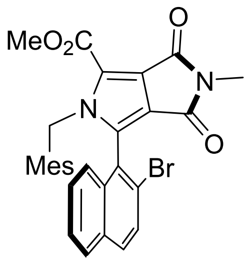

(*S*)-Methyl 3-(2-bromonaphthalen-1-yl)-5-methyl-4,6-dioxo-2-(2,4,6-trimethylbenzyl)-2,4,5,6-tetrahydropyrrolo[3,4-c]pyrrole-1-carboxylate (9f). Compound **9f** was prepared following the general procedure from methyl (1*S*,3*R*,3a*S*,6a*R*)-3-(2-bromonaphthalen-1-yl)-5-methyl-4,6-dioxooctahydropyrrolo[3,4-c]pyrrole-1-carboxylate (**3f**) (115 mg, 0.21 mmol, 1.00 equiv). The residue was purified by silica gel flash chromatography (Heptane-EtOAc 7:1) to afford **9f** (101 mg, 88%, yellow solid). **^{1}H NMR (300 MHz, $CDCl_3$, δ):** 7.69 (d, *J* = 5.3 Hz, 1H), 7.66 (d, *J* = 6.2 Hz, 1H), 7.50 (d, *J* = 8.8 Hz, 1H), 7.42 – 7.34 (m, 1H), 7.25 – 7.18 (m, 1H), 7.08 (d, *J* = 8.3 Hz, 1H), 6.17 (s, 2H), 6.02 (d, *J* = 15.8 Hz, 1H), 5.55 (d, *J* = 15.8 Hz, 1H), 4.06 (s, 3H), 3.04 (s, 3H), 1.86 (s, 3H), 1.67 (s, 6H). **^{13}C NMR (75 MHz, $CDCl_3$, δ):** 163.2, 163.1, 160.9, 136.7, 133.7, 133.0, 132.0, 131.4, 129.5, 129.4, 129.3, 128.1, 127.7, 127.1, 126.3, 124.9, 124.8, 123.6, 121.5, 120.7, 52.7, 46.1, 24.3, 20.6, 19.7. **HRMS-ESI (*m/z*):** calcd. for $C_{29}H_{26}BrN_2O_4$ (M+H$^+$): 545.1070; found: 545.1056.

$[\alpha]_{D\ 20}$: +32.8 (c = 0.03, CH_2Cl_2), 99% *ee.*

HPLC: Chiralpak IG, hexane/^{i}PrOH 95/5 in 25 min, flow rate 1 mL/min (λ = 250 nm) t_R: 15.64 min (*R*)-**9f** and 19.62 min (*S*)-**9f**.

The structure of this compound was confirmed by X-ray diffraction.

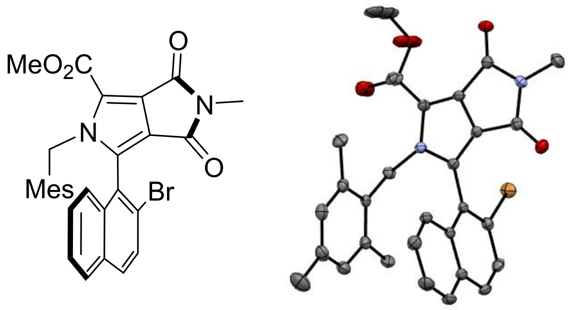

CCDC 2297239

ORTEP view of substrate **(*S*)-9f**. Hydrogen atom have been removed for simplicity.

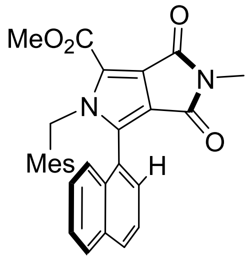

(*rac*)-Methyl 5-methyl-3-(naphthalen-1-yl)-4,6-dioxo-2-(2,4,6-trimethylbenzyl)-2,4,5,6-tetrahydropyrrolo[3,4-c]pyrrole-1-carboxylate (9g). Compound **9g** was prepared following the general procedure from methyl (1*S*,3*R*,3a*S*,6a*R*)-5-methyl-3-(naphthalen-1-yl)-4,6-dioxooctahydropyrrolo[3,4-c]pyrrole-1-carboxylate (**3g**) (98.8 mg, 0.21 mmol, 1.00 equiv). The residue was purified by silica gel flash chromatography (Heptane-EtOAc 7:1) to afford **9g** (73.5 mg, 75%, white solid). **^{1}H NMR (500 MHz, $CDCl_3$, δ):** 7.87 – 7.81 (m, 2H), 7.51 – 7.39 (m, 3H), 7.29 – 7.27 (m, 1H), 7.11 (d, *J* = 6.3 Hz, 1H), 6.37 (s, 2H), 5.85 (d, *J* = 15.7 Hz, 1H), 5.22 (d, *J* = 15.8 Hz, 1H), 3.95 (s, 3H), 3.04 (s, 3H), 2.03 (s, 3H), 1.58 (s, 6H). **^{13}C NMR (126 MHz, $CDCl_3$, δ):** 163.7, 163.2, 160.7, 137.0, 136.4, 136.0, 133.5, 131.5, 130.2, 129.8, 129.3, 128.7, 128.6, 127.2, 126.3, 125.9, 125.1, 124.8, 124.6, 120.9, 120.6, 52.6, 46.7, 29.8, 24.3, 20.7, 19.4. **HRMS-ESI (*m/z*):** calcd. for $C_{29}H_{27}N_2O_4$ (M+H$^+$): 467.1938; found: 467.1960.

$[\alpha]_{D\ 20}$: 0.0 (c = 0.03, CH_2Cl_2), 0% *ee.*

HPLC: Chiralpak IB, hexane/^{i}PrOH 80/20 in 30 min, flow rate 1 mL/min (λ = 230 nm) t_R: 8.90 min (1*R*)-**9g** and 17.50 min (*S*)-**9g**.

(*R*)-Methyl 3-(1-bromonaphthalen-2-yl)-5-methyl-4,6-dioxo-2-(2,4,6-trimethylbenzyl)-2,4,5,6-tetrahydropyrrolo[3,4-c]pyrrole-1-carboxylate (13a). Compound **13a** was prepared following the general procedure from methyl(1*S*,3*R*,3a*R*,6a*S*)-3-(1-bromonaphthalen-2-yl)-5-methyl-4,6-dioxooctahydropyrrolo[3,4-c]pyrrole-1-carboxylate (**11a**) (115 mg, 0.21 mmol, 1.00 equiv). The residue was purified by silica gel flash chromatography (Heptane-EtOAc 7:1) to afford **13a** (82.5 mg, 72%, white solid).

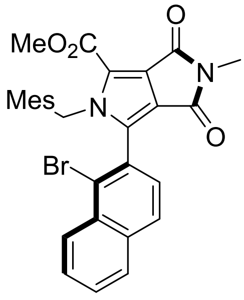

^{1}H NMR (300 MHz, CDCl$_3$, δ): 8.28 (d, *J* = 8.2 Hz, 1H), 7.78 (d, *J* = 8.1 Hz, 1H), 7.70 – 7.54 (m, 2H), 7.44 (d, *J* = 8.4 Hz, 1H), 6.67 (d, *J* = 8.4 Hz, 1H), 6.40 (s, 2H), 6.28 (d, *J* = 15.8 Hz, 1H), 5.24 (d, *J* = 15.8 Hz, 1H), 3.98 (s, 3H), 3.05 (s, 3H), 2.10 (s, 3H), 1.74 (s, 6H). **^{13}C NMR (75 MHz, CDCl$_3$, δ):** 163.4, 163.2, 160.7, 137.3, 136.9, 136.2, 134.6, 131.8, 130.1, 129.3, 128.4, 128.3, 128.1, 127.9, 127.6, 127.5, 126.9, 125.0, 124.6, 120.2, 120.2, 52.6, 46.4, 24.3, 20.8, 19.6. **HRMS-ESI (*m/z*):** calcd. for $C_{29}H_{26}BrN_2O_4$ (M+H$^+$): 545.1070; found: 545.1056.

$[\alpha]_{D\ 20}$: +52.6 (c = 0.3, CH_2Cl_2), 90% *ee*.

HPLC: Chiralpak IB, hexane/iPrOH 80/20 in 30 min, flow rate 1 mL/min (λ = 254 nm), t_R: 7.44 min (*R*)-**13a** and 14.43 min (*S*)-**13a**.

The structure of this compound was confirmed by X-ray diffraction.

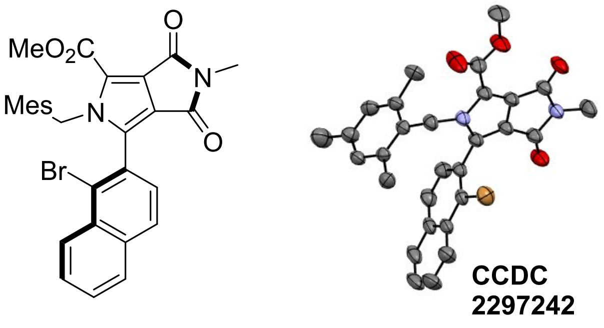

ORTEP view of substrate **13a**. Hydrogen atom have been removed for simplicity.

(*R*)-Methyl 3-(1-iodonaphthalen-2-yl)-5-methyl-4,6-dioxo-2-(2,4,6-trimethylbenzyl)-2,4,5,6-tetrahydropyrrolo[3,4-c]pyrrole-1-carboxylate (13b). Compound **13b** was prepared following the general procedure from methyl(1*S*,3*R*,3a*R*,6a*S*)-3-(1-idodonaphthalen-2-yl)-5-methyl-4,6-dioxooctahydropyrrolo[3,4-c]pyrrole-1-carboxylate (**11b**) (97.5 mg, 0.21 mmol, 1.00 equiv). The residue was purified by silica gel flash chromatography (Heptane-EtOAc 7:1) to afford **13b** (87.1 mg, 70%, yellow solid). **^{1}H NMR (300 MHz, CDCl$_3$, δ):** 8.17 (d, *J* = 8.4 Hz, 1H), 7.73 – 7.69 (m, 1H), 7.64 – 7.53 (m, 2H), 7.37 (d, *J* = 8.4 Hz, 1H), 6.50 (d, *J* = 8.4 Hz, 1H), 6.40 (s, 2H), 6.39 (d, *J* = 9.5 Hz, 1H), 5.15 (d, *J* = 15.9 Hz, 1H), 4.02 (s, 3H), 3.05 (s, 3H), 2.13 (s, 3H), 1.70 (s, 6H). **^{13}C NMR (75 MHz, CDCl$_3$, δ):** 163.3, 163.2, 160.8, 139.3, 137.4, 137.0, 134.3, 133.8, 133.8, 132.8, 130.1, 129.4, 128.5, 128.3, 127.9, 126.5, 124.7, 120.1, 119.8, 106.0, 52.6,

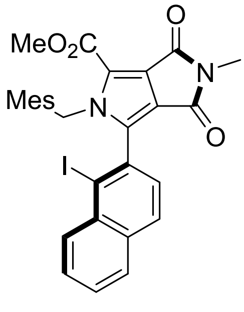

46.4, 24.3, 20.8, 19.7. **HRMS-ESI (*m/z*):** calcd. for $C_{29}H_{26}IN_2O_4$ (M+H$^+$): 593.0932; found: 593.0924.

[α]$_{D\ 20}$: +112.4 (c = 0.3, CH_2Cl_2), 99% *ee*.

HPLC: Chiralpak IB, hexane/iPrOH 90/10 in 30 min, flow rate 1 mL/min (λ = 254 nm), t_R: 13.27 min (*R*)-**13b** and 18.73 min (*S*)-**13b**.

(*R*)-Methyl 3-(1-ethylnaphthalen-2-yl)-5-methyl-4,6-dioxo-2-(2,4,6-trimethylbenzyl)-2,4,5,6-tetrahydropyrrolo[3,4-c]pyrrole-1-carboxylate (13c).

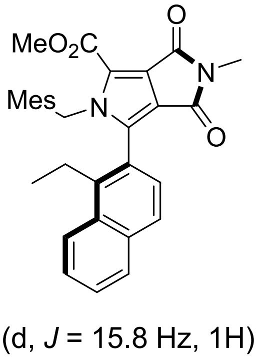

Compound **13c** was prepared following the general procedure from methyl(1*S*,3*R*,3a*R*,6a*S*)-3-(1-ethylnaphthalen-2-yl)-5-methyl-4,6-dioxooctahydropyrrolo[3,4-c]pyrrole-1-carboxylate (**11c**) (76.9 mg, 0.21 mmol, 1.00 equiv). The residue was purified by silica gel flash chromatography (Heptane-EtOAc 8:1) to afford **13c** (95.6 mg, 92%, white solid). **^{1}H NMR (300 MHz, CDCl$_3$, δ):** 7.99 – 7.94 (m, 1H), 7.82 – 7.78 (m, 1H), 7.55 – 7.51 (m, 2H), 7.42 (d, *J* = 8.4 Hz, 1H), 6.74 (d, *J* = 8.4 Hz, 1H), 6.43 (s, 2H), 5.88 (d, *J* = 15.7 Hz, 1H), 5.35 (d, *J* = 15.8 Hz, 1H), 3.97 (s, 3H), 3.04 (s, 3H), 2.98 – 2.88 (m, 1H), 2.45 – 2.35 (m, 1H), 2.13 (s, 3H), 1.67 (s, 6H), 1.10 (t, *J* = 7.5 Hz, 3H). **^{13}C NMR (75 MHz, CDCl$_3$, δ):** 163.7, 163.3, 160.8, 141.1, 137.4, 137.3, 137.0, 134.4, 131.2, 129.7, 129.4, 128.9, 126.8, 126.7, 126.5, 126.4, 124.7, 124.6, 124.4, 120.5, 120.2, 52.6, 46.0, 24.3, 23.7, 20.8, 19.6, 15.2. **HRMS-ESI (*m/z*):** calcd. for $C_{31}H_{31}N_2O_4$ (M+H$^+$): 495.2292; found: 495.2278.

[α]$_{D\ 20}$: -39.3 (c = 0.3, CH_2Cl_2), 99% *ee*.

HPLC: Chiralpak IC, hexane/iPrOH 20/80 in 30 min, flow rate 1 mL/min (λ = 254 nm), t_R: 10.90 min (*R*)-**13c** and 14.26 min (*S*)-**13c**.

(±)-Methyl 3-(1-(4-methoxyphenyl)naphthalen-2-yl)-5-methyl-4,6-dioxo-2-(2,4,6-trimethylbenzyl)-2,4,5,6-tetrahydropyrrolo[3,4-c]pyrrole-1-carboxylate (13d).

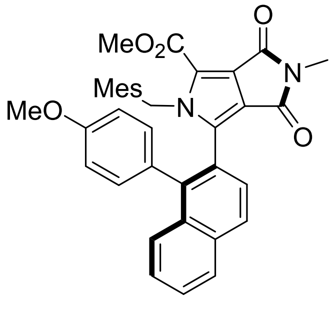

Compound **13d** was prepared following the general procedure from methyl (1*S*,3*R*,3a*S*,6a*R*)-3-(1-(4-methoxyphenyl)naphthalen-2-yl)-5-methyl-4,6-dioxooctahydropyrrolo[3,4-c]pyrrole-1-carboxylate (**11d**) (93.3 mg, 0.21 mmol, 1.00 equiv). The residue was purified by silica gel flash chromatography (Heptane-EtOAc 8:1) to afford **13d** (103 mg, 86%, white solid). **^{1}H NMR (300 MHz, CDCl$_3$, δ):** 7.89 (d, *J* = 8.2 Hz, 1H), 7.76 (d, *J* = 8.5 Hz, 1H), 7.72 (d, *J* = 8.6 Hz, 1H), 7.55 (ddd, *J* = 8.1, 6.7, 1.3 Hz, 1H), 7.44 (ddd, *J* = 8.3, 6.8, 1.4 Hz, 1H), 7.30 (dd, *J* = 8.4, 2.3 Hz, 1H), 7.12 (d, *J* = 8.5 Hz, 1H), 7.06 – 6.97 (m, 2H), 6.82 (dd, *J* = 8.5, 2.7 Hz, 1H), 6.60 (s, 2H), 5.24 (d, *J* = 15.7 Hz, 1H), 4.78 (d, *J* = 15.6 Hz, 1H), 3.86 (s, 3H), 3.70 (s, 3H), 3.04 (s, 3H), 2.18 (s, 3H), 1.74 (s, 6H). **^{13}C NMR (75 MHz, CDCl$_3$, δ):** 163.9, 163.3, 159.9, 159.4, 141.0, 137.6, 137.2, 136.9, 134.4, 132.5, 132.0, 131.3, 130.1, 129.7, 129.6, 128.3, 127.8, 127.6, 127.2, 127.0, 126.8, 125.1, 124.4, 120.1, 120.0, 114.0, 113.7, 55.5, 52.2, 46.6, 24.2, 20.8, 19.8.

[α]$_{D\ 20}$: 0.0 (c = 0.3, CH_2Cl_2), 0% *ee*.

HPLC: Chiralpak IC, hexane/iPrOH 50/50 in 15 min, flow rate 1 mL/min (λ = 254 nm), t_R: 7.80 min (*R*)-**13d** and 12.01 min (*S*)-**13d**.

Methyl 3-(1-methoxynaphthalen-2-yl)-5-methyl-4,6-dioxo-2-(2,4,6-trimethylbenzyl)-2,4,5,6-tetrahydropyrrolo[3,4-c]pyrrole-1-carboxylate (13e).

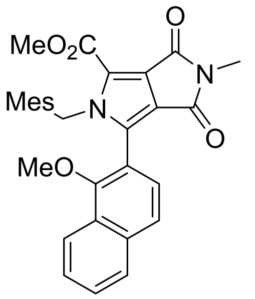

Compound **13e** was prepared following the general procedure from methyl (1*S*,3*R*,3a*S*,6a*R*)-3-(1-methoxynaphthalen-2-yl)-5-methyl-4,6-dioxooctahydropyrrolo[3,4-c]pyrrole-1-carboxylate (**11e**) (105 mg, 0.21 mmol, 1.00 equiv). The residue was purified by silica gel flash chromatography (Heptane-EtOAc 6:1) to afford **13e** (67.8 mg, 65%, white solid). **^{1}H NMR (300 MHz, $CDCl_3$, δ):** 8.24 – 8.13 (m, 1H), 7.87 – 7.77 (m, 1H), 7.60 – 7.51 (m, 2H), 7.45 (d, *J* = 8.5 Hz, 1H), 7.06 (d, *J* = 8.5 Hz, 1H), 6.44 (s, 2H), 6.06 (d, *J* = 15.8 Hz, 1H), 5.16 (d, *J* = 15.8 Hz, 1H), 3.85 (s, 3H), 3.68 (s, 3H), 3.06 (s, 3H), 2.08 (s, 3H), 1.82 (s, 6H). **^{13}C NMR (75 MHz, $CDCl_3$, δ):** 163.9, 163.2, 160.4, 154.7, 136.9, 136.8, 135.8, 134.9, 130.2, 129.2, 128.2, 127.8, 127.6, 126.6, 124.7, 124.4, 122.6, 121.1, 119.1, 117.6, 62.1, 52.5, 47.0, 24.3, 20.7, 19.7. **HRMS-ESI (*m/z*):** calcd. for $C_{30}H_{29}N_2O_5$ (M+H$^+$): 497.2071; found: 497.2085.

Methyl 3-(1-isopropoxynaphthalen-2-yl)-5-methyl-4,6-dioxo-2-(2,4,6-trimethylbenzyl)-2,4,5,6-tetrahydropyrrolo[3,4-c]pyrrole-1-carboxylate (13f).

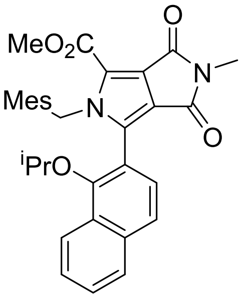

Compound **13f** was prepared following the general procedure from methyl (1*S*,3*R*,3a*S*,6a*R*)-3-(1-isopropoxynaphthalen-2-yl)-5-methyl-4,6-dioxooctahydropyrrolo[3,4-c]pyrrole-1-carboxylate (**11f**) (111 mg, 0.21 mmol, 1.00 equiv). The residue was purified by silica gel flash chromatography (Heptane-EtOAc 6:1) to afford **13f** (71.6 mg, 65%, white solid). **^{1}H NMR (300 MHz, $CDCl_3$, δ):** 8.26 – 8.21 (m, 1H), 7.84 – 7.78 (m, 1H), 7.57 – 7.50 (m, 3H), 7.26 – 7.22 (m, 1H), 6.46 (s, 2H), 6.01 (d, *J* = 15.9 Hz, 1H), 5.10 (d, *J* = 15.9 Hz, 1H), 4.05 (hept, *J* = 6.1 Hz, 1H), 3.79 (s, 3H), 3.07 (s, 3H), 2.08 (s, 3H), 1.85 (s, 6H), 1.22 (d, *J* = 6.1 Hz, 3H), 1.10 (d, *J* = 6.1 Hz, 3H). **^{13}C NMR (75 MHz, $CDCl_3$, δ):** 163.9, 163.3, 160.2, 152.7, 136.8, 136.8, 136.3, 135.8, 130.2, 129.3, 129.2, 128.0, 127.8, 127.5, 126.3, 124.6, 124.2, 123.5, 121.2, 118.6, 118.1, 77.9, 52.4, 47.4, 24.3, 22.9, 22.7, 20.7, 19.7. **HRMS-ESI (*m/z*):** calcd. for $C_{32}H_{33}N_2O_5$ (M+H$^+$): 525.2384; found: 525.2400.

(*R*)-Methyl 3-(2-methylnaphthalen-1-yl)-4,6-dioxo-5-phenyl-2-(2,4,6-trimethylbenzyl)-2,4,5,6-tetrahydropyrrolo[3,4-c]pyrrole-1-carboxylate (17a).

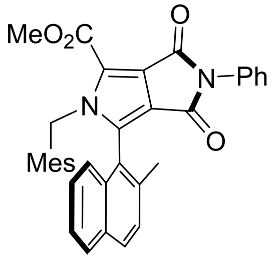

Compound **17a** was prepared following the general procedure from methyl (1*S*,3*R*,3a*S*,6a*R*)-3-(2-methylnaphthalen-1-yl)-4,6-dioxo-5-phenyloctahydropyrrolo[3,4-c]pyrrole-1-carboxylate (**15a**) (115 mg, 0.21 mmol, 1.00 equiv). The residue was purified by silica gel flash chromatography (Heptane-EtOAc 4:1) to afford **17a** (53.6 mg, 47%, yellow solid). **^{1}H NMR (300 MHz, $CDCl_3$, δ):** ^{1}H NMR (500 MHz, Chloroform-*d*) δ 7.80 (dd, *J* = 7.7, 1.4 Hz, 1H), 7.73 (d, *J* = 8.4 Hz, 1H), 7.45 – 7.34 (m, 6H), 7.33 – 7.29 (m, 1H), 7.15 (d, *J* = 8.4 Hz, 1H), 7.10 (d, *J* = 8.5 Hz, 1H), 6.39 (s, 2H), 6.18 (d, *J* = 15.8 Hz, 1H), 5.20 (d, *J* = 15.8 Hz, 1H), 4.06 (s, 3H), 2.07 (s, 3H), 1.93 (s, 3H), 1.46 (s, 6H). **^{13}C NMR (75 MHz, $CDCl_3$, δ):** 162.3, 162.1, 161.0, 137.6, 137.0, 136.8, 135.9, 132.7, 132.3, 132.0, 129.9, 129.7, 129.3, 129.2, 128.9, 128.5, 128.4, 128.4, 127.7, 127.4, 127.0, 125.4, 124.3, 124.0, 123.6, 121.3, 120.8, 52.7, 46.1, 20.7, 20.0, 19.3. **HRMS-ESI (*m/z*):** calcd. for $C_{35}H_{31}N_2O_4$ (M+H$^+$): 543.2278; found: 543.2286.

$[\alpha]_{D\ 20}$: +7.59 (c = 0.04, CH_2Cl_2), 86% *ee*.

HPLC: Chiralpak IA, hexane/^{i}PrOH 90/10 in 15 min, flow rate 1 mL/min (λ = 254 nm) t_R: 4.22 min (*R*)-**17a** and 4.96 min (*S*)-**17a**.

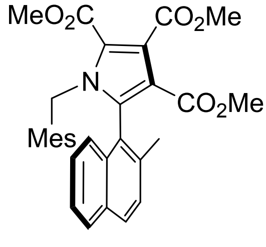

(*R*)-Trimethyl 5-(2-methylnaphthalen-1-yl)-1-(2,4,6-trimethylbenzyl)-1H-pyrrole-2,3,4-tricarboxylate (17b). Compound **17b** was prepared following the general procedure from trimethyl (2*S*,3*S*,4*S*,5*R*)-5-(2-methylnaphthalen-1-yl)pyrrolidine-2,3,4-tricarboxylate (**15b**) (109 mg, 0.21 mmol, 1.00 equiv). The residue was purified by silica gel flash chromatography (Heptane-EtOAc 4:1) to afford **17b** (76.6 mg, 71%, yellow solid). **^{1}H NMR (300 MHz, $CDCl_3$, δ):** 7.73 (d, *J* = 8.1 Hz, 1H), 7.70 (d, *J* = 8.4 Hz, 1H), 7.37 – 7.32 (m, 1H), 7.28 – 7.24 (m, 1H), 7.08 (d, *J* = 8.4 Hz, 1H), 6.95 (d, *J* = 8.4 Hz, 1H), 6.36 (s, 2H), 5.96 (d, *J* = 15.7 Hz, 1H), 5.17 (d, *J* = 15.7 Hz, 1H), 3.98 (s, 3H), 3.91 (s, 3H), 3.40 (s, 3H), 2.05 (s, 3H), 1.78 (s, 3H), 1.46 (s, 6H). **^{13}C NMR (75 MHz, $CDCl_3$, δ):** 166.9, 162.8, 161.2, 141.8, 137.7, 137.0, 136.7, 132.7, 131.7, 129.9, 129.2, 129.2, 128.1, 128.1, 126.9, 126.0, 125.8, 125.0, 124.3, 122.0, 113.7, 52.8, 52.4, 51.5, 45.2, 20.7, 20.0, 19.4. **HRMS-ESI (*m/z*):** calcd. for $C_{31}H_{32}NO_6$ (M+H$^+$): 514.2224; found: 514.2238.

$[\alpha]_{D\ 20}$: -14.6 (c = 0.30, CH_2Cl_2), 60% *ee*.

SFC: Chiralpak IB, CO_2-MeOH 95:5 to 70-30, flow rate 2 mL/min (λ = 280 nm), t_R: 1.94 min (*R*)-**17b** and 2.22 min (*S*)-**17b**.

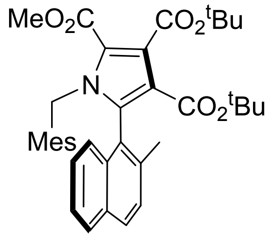

(*R*)-3,4-Di-tert-butyl 2-methyl 5-(2-methylnaphthalen-1-yl)-1-(2,4,6-trimethylbenzyl)-1H-pyrrole-2,3,4-tricarboxylate (17c). Compound **17c** was prepared following the general procedure from 3,4-Di-tert-butyl 2-methyl (2*S*,3*S*,4*S*,5*R*)-5-(2-methylnaphthalen-1-yl)pyrrolidine-2,3,4-tricarboxylate (**15c**) (98.6 mg, 0.21 mmol, 1.00 equiv). The residue was purified by silica gel flash chromatography (Heptane-EtOAc 7:1) to afford **17c** (86.6 mg, 69%, yellow solid). **^{1}H NMR (300 MHz, $CDCl_3$, δ):** 8.06 (d, *J* = 8.7 Hz, 1H), 7.85 – 7.79 (m, 1H), 7.66 (d, *J* = 8.3 Hz, 1H), 7.59 (ddd, *J* = 8.5, 6.8, 1.5 Hz, 1H), 7.51 – 7.43 (m, 1H), 7.07 (d, *J* = 8.3 Hz, 1H), 6.71 (s, 2H), 5.30 (d, *J* = 9.8 Hz, 1H), 4.07 (d, *J* = 14.6 Hz, 1H), 3.94 (s, 3H), 2.23 (s, 3H), 1.58 (s, 3H), 1.53 (s, 3H), 1.46 (s, 9H), 1.45 (s, 9H). **^{13}C NMR (75 MHz, $CDCl_3$, δ):** 172.8, 164.2, 163.7, 152.8, 138.8, 137.7, 136.2, 133.3, 131.9, 131.3, 129.2, 129.1, 129.0, 128.9, 127.1, 127.0, 124.9, 123.7, 98.2, 80.9, 64.8, 54.9, 52.9, 44.4, 28.5, 28.0, 27.9, 20.8, 19.4, 19.3. **HRMS-ESI (*m/z*):** calcd. for $C_{37}H_{44}NO_6$ (M+H$^+$): 597.3240; found: 597.3241.

$[\alpha]_{D\ 20}$: -219.5 (c = 0.30, CH_2Cl_2), 78% *ee*.

HPLC: Chiralpak OD, hexane/iPrOH 95/5 in 40 min, flow rate 0.50 mL/min (λ = 230 nm), t_R: 12.98 min (*S*)-**17c** and 19.19 min (*R*)-**17c**.

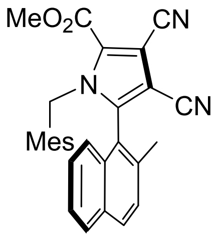

(*R*)-Methyl 3,4-dicyano-5-(2-methylnaphthalen-1-yl)-1-(2,4,6-trimethylbenzyl)-1H-pyrrole-2-carboxylate (17c). Compound **17c** was prepared following the general procedure from methyl (2*S*,3*S*,4*S*,5*R*)-3,4-dicyano-5-(2-methylnaphthalen-1-yl)pyrrolidine-2-carboxylate (**15c**) (94.8 mg, 0.21 mmol, 1.00 equiv). The residue was purified by silica gel flash chromatography (Heptane-EtOAc 4:1) to afford **17c** (52.6 mg, 56%, yellow solid). **^{1}H NMR (300 MHz, $CDCl_3$, δ):** 7.84 – 7.74 (m, 2H), 7.46

– 7.40 (m, 1H), 7.36 (ddd, *J* = 8.2, 7.0, 1.4 Hz, 1H), 7.12 (d, *J* = 8.5 Hz, 1H), 6.87 (d, *J* = 8.3 Hz, 1H), 6.37 (s, 2H), 6.09 (d, *J* = 15.7 Hz, 1H), 5.25 (d, *J* = 15.7 Hz, 1H), 4.08 (s, 3H), 2.05 (s, 3H), 1.85 (s, 3H), 1.41 (s, 6H). **^{13}C NMR (75 MHz, $CDCl_3$, δ):** 159.5, 145.6, 137.9, 137.6, 136.9, 132.3, 131.9, 130.8, 129.6, 129.5, 128.6, 128.5, 128.3, 127.8, 125.8, 123.4, 122.3, 112.4, 111.9, 102.7, 100.5, 53.1, 46.7, 20.7, 19.8, 19.2. **HRMS-ESI (*m/z*):** calcd. for $C_{29}H_{26}N_3O_2$ (M+H$^+$): 448.2020; found: 448.2034.

$[\alpha]_{D\ 20}$: -1.23 (c = 0.04, CH_2Cl_2), 99% *ee*.

HPLC: Chiralpak IA, hexane/^{i}PrOH 90/10 in 15 min, flow rate 1 mL/min (λ = 254 nm) t_R: 3.80 min (*S*)-**17c** and 4.23 min (*R*)-**17c**.

(*R*)-Methyl 2-benzyl-5-methyl-3-(2-methylnaphthalen-1-yl)-4,6-dioxo-2,4,5,6-tetrahydropyrrolo[3,4-c]pyrrole-1-carboxylate (6a). Compound **6a** was prepared following the general procedure from methyl(1*S*,3*R*,3a*R*,6a*S*)-5-methyl-3-(2-methylnaphthalen-1-yl)-4,6-dioxooctahydropyrrolo[3,4-c]pyrrole-1-carboxylate (**3a**) (102 mg, 0.21 mmol, 1.00 equiv). The residue was purified by silica gel flash chromatography (Heptane-EtOAc 6:1) to afford **6a** (90.2 mg, 98%, yellow solid). **^{1}H NMR (500 MHz, $CDCl_3$, δ):** 7.90 – 7.85 (m, 2H), 7.45 (ddd, *J* = 8.1, 6.8, 1.3 Hz, 1H), 7.40 (ddd, *J* = 8.2, 6.8, 1.4 Hz, 1H), 7.31 (d, *J* = 8.5 Hz, 1H), 7.22 (d, *J* = 8.6 Hz, 1H), 7.14 – 7.10 (m, 1H), 7.08 – 7.04 (m, 2H), 5.78 (d, *J* = 15.1 Hz, 1H), 4.82 (d, *J* = 15.1 Hz, 1H), 4.02 (s, 3H), 3.06 (s, 3H), 1.95 (s, 3H). **^{13}C NMR (126 MHz, $CDCl_3$, δ):** 163.6, 163.2, 160.4, 138.2, 136.3, 134.5, 132.5, 132.0, 130.6, 128.7, 128.6, 128.4, 127.8, 127.6, 127.3, 125.8, 125.7, 124.1, 123.2, 121.4, 119.2, 52.6, 50.0, 24.3, 20.1. **HRMS-ESI (*m/z*):** calcd. for $C_{27}H_{23}N_2O_4$ (M+H$^+$): 439.1655; found: 439.1652.

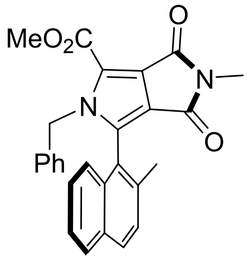

Methyl 5-methyl-3-(2-methylnaphthalen-1-yl)-4,6-dioxo-2,4,5,6-tetrahydropyrrolo[3,4-c]pyrrole-1-carboxylate (5a). Compound **5a** was prepared following the general procedure from methyl(1*S*,3*R*,3a*R*,6a*S*)-5-methyl-3-(2-methylnaphthalen-1-yl)-4,6-dioxooctahydropyrrolo[3,4-c]pyrrole-1-carboxylate (**3a**) (102 mg, 0.21 mmol, 1.00 equiv) and adding water (3.00 equiv). The residue was purified by silica gel flash chromatography (Heptane-EtOAc 3:1) to afford **6a** (57.1 mg, 78%, white solid). **^{1}H NMR (500 MHz, $CDCl_3$, δ):** 9.97 (bs, 1H), 7.91 – 7.83 (m, 2H), 7.51 – 7.40 (m, 4H), 3.81 (s, 3H), 3.07 (s, 3H), 2.41 (s, 3H). **^{13}C NMR (126 MHz, $CDCl_3$, δ):** 163.7, 162.9, 160.1, 137.1, 132.7, 132.1, 130.8, 130.4, 128.7, 128.5, 127.4, 125.8, 124.5, 124.0, 123.7, 122.4, 118.5, 52.9, 24.4, 20.6. **HRMS-ESI (*m/z*):** calcd. for $C_{20}H_{17}N_2O_4$ (M+H$^+$): 349.1183; found: 349.1183.

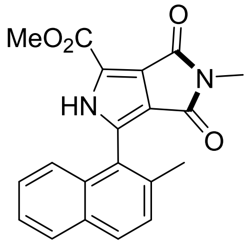

Methyl (1*S*,3*R*,3a*S*,6a*R*)-2-acetyl-5-methyl-3-(2-methylnaphthalen-1-yl)-4,6-dioxooctahydropyrrolo[3,4-c]pyrrole-1-carboxylate (8b). To a stirred solution of methyl(1*S*,3*R*,3a*R*,6a*S*)-5-methyl-3-(2-methylnaphthalen-1-yl)-4,6-dioxooctahydropyrrolo[3,4-c]pyrrole-1-carboxylate **3a** (26.0 mg, 0.07 mmol, 1.00 equiv) and Et_3N (9.95 µL, 0.14 mmol, 2.0 0 equiv) in dichloromethane (3.00 mL was added at 0° C acetyl chloride (5.84 µL, 0.09 mmol, 1.20 equiv). The mixture was stirred at room temperature for 16 h, evaporated,

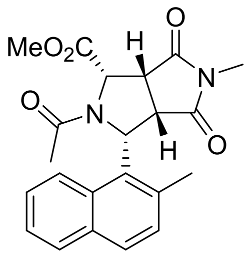

dissolved in water and extracted with dichloromethane (2x10.0 mL). The combined organic layers were washed with water (10.0 mL), brine (10.0 mL), dried over sodium sulphate anhydrous and evaporated. The residue was purified by silica gel flash chromatography (Heptane-EtOAc 6:1) to afford **8b** (30.7 mg, 82%, white solid). **^{1}H NMR (300 MHz, $CDCl_3$, δ):** 8.84 (d, *J* = 7.8 Hz, 1H), 7.80 (d, *J* = 7.4 Hz, 1H), 7.77 (d, *J* = 8.3 Hz, 1H), 7.47 – 7.38 (m, 2H), 7.35 (d, *J* = 8.4 Hz, 1H), 5.98 (d, *J* = 10.9 Hz, 1H), 4.90 (d, *J* = 9.7 Hz, 1H), 3.96 (s, 3H), 3.93 – 3.87 (m, 1H), 3.87 – 3.78 (m, 1H), 2.66 (s, 3H), 2.39 (s, 3H), 1.56 (s, 3H). **^{13}C NMR (75 MHz, $CDCl_3$, δ):** 174.6, 173.3, 173.2, 169.0, 134.5, 133.3, 131.4, 129.8, 129.5, 129.2, 128.4, 126.3, 125.3, 124.3, 62.9, 61.3, 52.9, 48.4, 44.9, 24.9, 22.5, 21.7. **HRMS-ESI (*m/z*):** calcd. for $C_{22}H_{23}N_2O_5$ (M+H$^+$): 395.1601; found: 395.1600.

Methyl (1*S*,3*R*,3a*S*,6a*R*)-5-methyl-3-(2-methylnaphthalen-1-yl)-4,6-dioxo-2-tosyloctahydropyrrolo[3,4-c]pyrrole-1-carboxylate (8c). To a stirred solution of methyl(1*S*,3*R*,3a*R*,6a*S*)-5-methyl-3-(2-methylnaphthalen-1-yl)-4,6-dioxooctahydropyrrolo[3,4-c]pyrrole-1-carboxylate **3a** (26.0 mg, 0.07 mmol, 1.00 equiv) and Et_3N (20.0 µL, 0.11 mmol, 1.50 equiv) in dichloromethane (3.00 mL was added at 0° C toluene sulfony-4-sulfonyl chloride (16.9 mg, 0.09 mmol, 1.20 equiv). The mixture was stirred at room temperature for 16 h, evaporated, dissolved in water and extracted with dichloromethane (2x10.0 mL). The combined organic layers were washed with water (10.0 mL), brine (10.0 mL), dried over sodium sulphate anhydrous and evaporated. The residue was purified by silica gel flash chromatography (Heptane-EtOAc 5:1) to afford **8c** (30.7 mg, 82%, white solid). **^{1}H NMR (300 MHz, $CDCl_3$, δ):** 8.07 (d, *J* = 8.7 Hz, 1H), 7.52 (d, *J* = 8.3 Hz, 1H), 7.45 (d, *J* = 8.2 Hz, 1H), 7.22 (d, *J* = 8.4 Hz, 1H), 7.15 – 7.09 (m, 1H), 7.04 – 7.00 (m, 1H), 6.98 (d, *J* = 8.0 Hz, 2H), 6.36 (d, *J* = 7.9 Hz, 2H), 6.00 (d, *J* = 11.1 Hz, 1H), 5.09 (d, *J* = 9.5 Hz, 1H), 4.03 (s, 3H), 3.92 – 3.85 (m, 1H), 3.85 – 3.79 (m, 1H), 2.70 (s, 3H), 2.57 (s, 3H), 1.96 (s, 3H). **^{13}C NMR (75 MHz, $CDCl_3$, δ):** 174.7, 173.5, 169.6, 143.3, 136.1, 133.5, 132.7, 132.0, 129.6, 129.0, 128.4, 128.0, 127.8, 125.3, 125.2, 124.4, 124.2, 62.9, 61.8, 53.1, 47.5, 45.5, 25.3, 21.4, 21.2. **HRMS-ESI (*m/z*):** calcd. for $C_{27}H_{27}N_2O_6S$ (M+H$^+$): 507.1584; found: 507.1578.

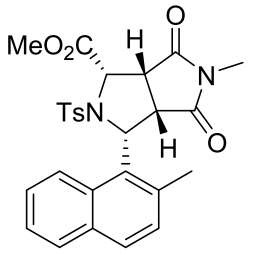

6. Synthetic method at a 1 mmol scale for the preparation of 9a

I. Cu-catalyzed asymmetric 1,3-dipolar cycloaddition

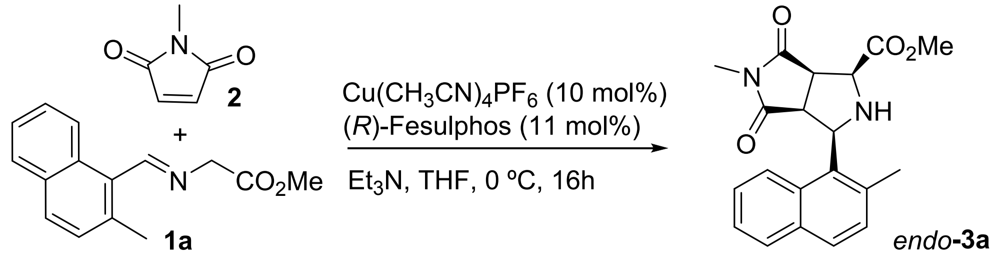

A sealed vial was charged with (*R*)-Fesulphos (45.8 mg, 0.10 mmol, 0.11 equiv) and $Cu(CH_3CN)_4PF_6$ (37.3 mg, 0.10 mmol, 0.10 equiv) in anhydrous THF (3.50 mL) under argon atmosphere. After that, a solution of (*E*)-2-(((2-methylnaphthalen-1-yl)methylene)amino)acetate (**1a**) (241 mg, 1.00 mmol, 1.00 equiv) in THF (2.00 mL) was added. Then, the solution was cold to 0 ºC and Et_3N (20.9 µL, 0.15 mmol, 0.15 equiv) and *N*-methylmaleimide (111 mg, 1.00 mmol, 1.00 equiv) were successively added. After stirring 16 h at 0 ºC, the mixture was diluted with dichloromethane (4.00 mL) and filtered through a short pad of Celite®. The solution was concentrated *in vacuo* and the residue was purified by silica gel flash chromatography (Heptane-EtOAc 2:1) to afford **3a** (293 mg, 83%, yellow wax) [87% *ee;* $[\alpha]_{D\,20}$: +29.05 (c = 0.3, $CHCl_3$)].

II. Blue light promoted aromatization of pyrrolidine 3a

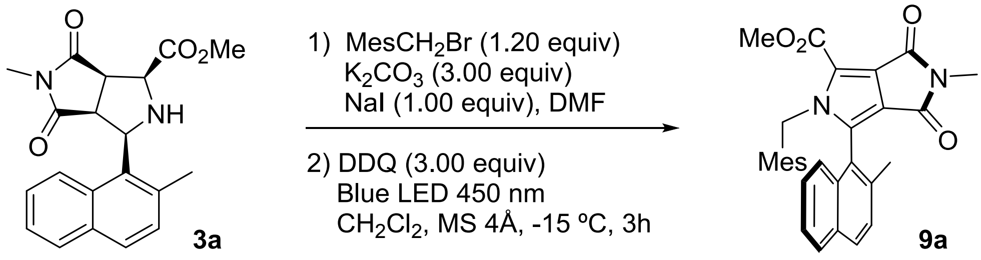

Step 1. *N*-alkylation of pyrrolidines with 2-bromomethyl-1,3,5-trimethylbenzene. A solution of pyrrolidine **3a** (293 mg, 0.83 mmol, 1.00 equiv), 2-(bromomethyl)-1,2,5-trimethylbenzene (213 mg, 1.00 mmol, 1.20 equiv), sodium carbonate (344 mg, 2.49 mmol, 3.00 equiv) and sodium iodine (124 mg, 0.83 mmol, 1.00 equiv) in dimethylformamide (8.50 mL) were stirred for 16 h at room temperature. The solution was dissolved with *tert*-butyl methyl ether (25.0 mL), washed with brine, and the volatiles were removed in vacuo affording the corresponding *N*-alkylated pyrrolidine **8a** which was used in next step without further purification.

Step 2. Blue light promoted aromatization. Four reactions were carried out in parallel following this general prpocedure:

A solution of the *N*-alkylated pyrrolidine **8a** (102 mg, 0.21 mmol, 1.00 equiv) and 2,3-dichloro-5,6-dicyano-1,4-benzoquinone (143 mg, 0.64 mmol, 3.00 equiv) in dry dichloromethane (6.00 mL) were stirred at -15 ºC under blue light for 3 h. The solution was dissolved with dichloromethane (10.0 mL), washed with $NaHCO_3$(sat) and concentrated under reduced pressure.

Then, the four residues were combined and purified by silica gel flash chromatography to afford the corresponding pyrrole in **9a** (363 mg, 90%, yellow wax) [84% *ee*; $[\alpha]_{D\ 20}$: +29.9 (c = 0.30, $CHCl_3$)].

7. Evaluation of the rotational barriers of 17a, 9c and 13a

The racemization experiments. A solution of the corresponding *N*-alkylated pyrrole (0.05 mmol, 1.00 equiv) in xylene (3.00 mL) were stirred at 110-130 ºC for 8 h. Aliquots of 0.10 mL were taken from time to time and the solution was concentrated under reduced pressure. The residue was analysed by HPLC to determine the enantiomeric excess.

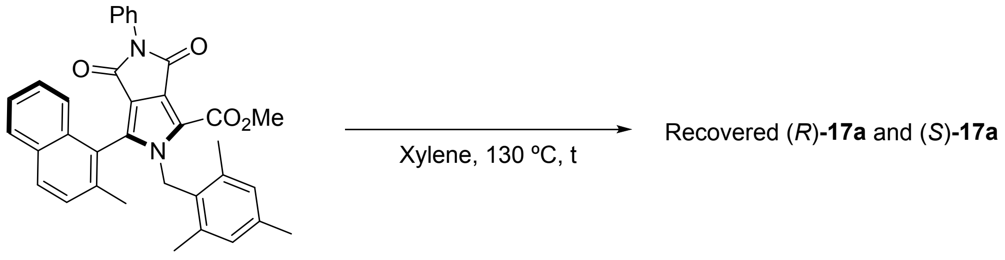

(*R*)-**17a**

**t (s)**	***ee* (%)**	**Ln(*ee*$_0$/*ee*$_1$)**
0	89	0
1800	87	0,02154226
4200	87	0,02903499
6000	86	0,03468002
7800	86	0,03679352
9600	85	0,04782812
12300	84	0,05502384
15600	84	0,06325003
17400	83	0,07111125
20100	82	0,07939863
22800	81	0,09283247
25200	81	0,09833112
27000	81	0,10274198

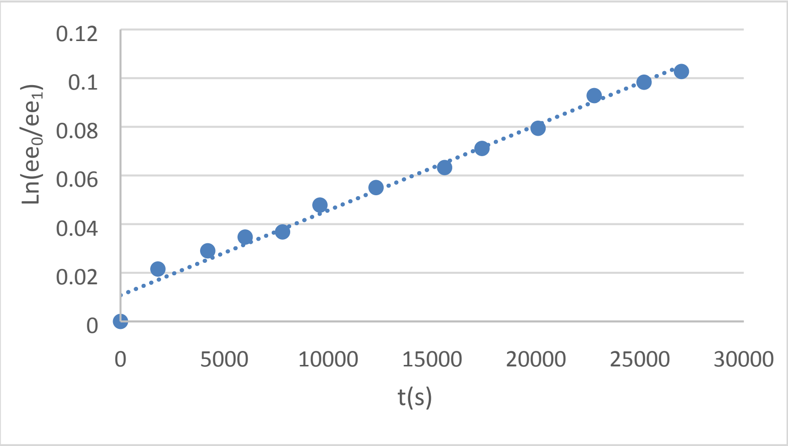

$k_{racemization}$ = 3.49 x 10^{-6}

$k_{enantiomerization}$ = 1.74 x 10^{-6}

$\Delta G^{\pm}$ = 33.574 kcal/mol

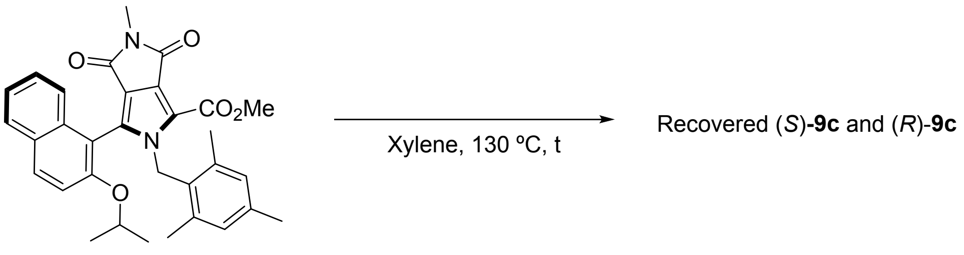

(*S*)-**9c**

t (s)	*ee* (%)	Ln(*ee*$_0$/*ee*$_1$)
0	87	0
900	86	0,006851
2700	86	0,01503196
4500	82	0,0542256
6300	81	0,06943632
10320	80	0,09005047
13500	78	0,10697101
18300	76	0,12929735
21480	74	0,1614377
24000	71	0,20590788
27000	69	0,22818497

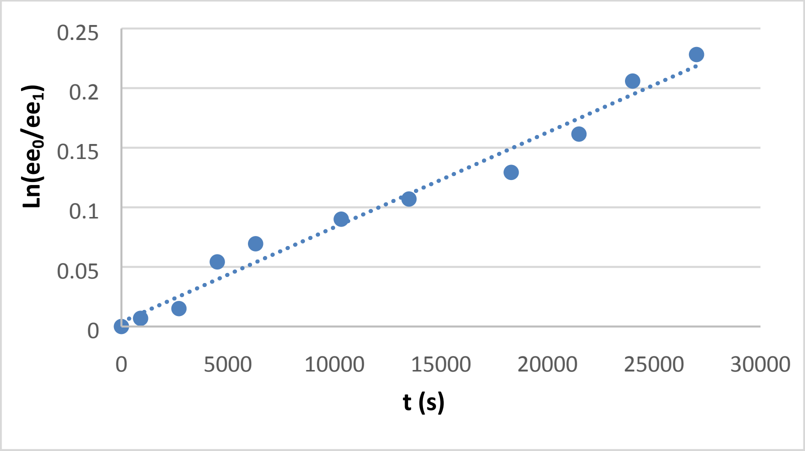

$k_{racemization}$ = 7.96 x 10^{-6}

$k_{enantiomerization}$ = 3.98 x 10^{-6}

ΔG± = 33.789 kcal/mol

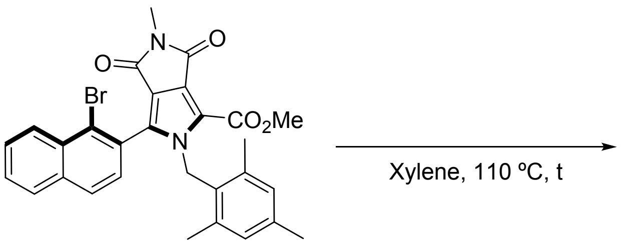

Recovered (*R*)-**13a** and (*S*)-**13a**

(*R*)-**13a**

t (s)	*ee* (%)	Ln(ee_0/ee_1)
0	85	0
1800	42	0,70308574
3600	21	1,41084571
5400	10	2,16899484
7200	4	2,93964709
9000	2	3,71053923
13200	0	5,35324832

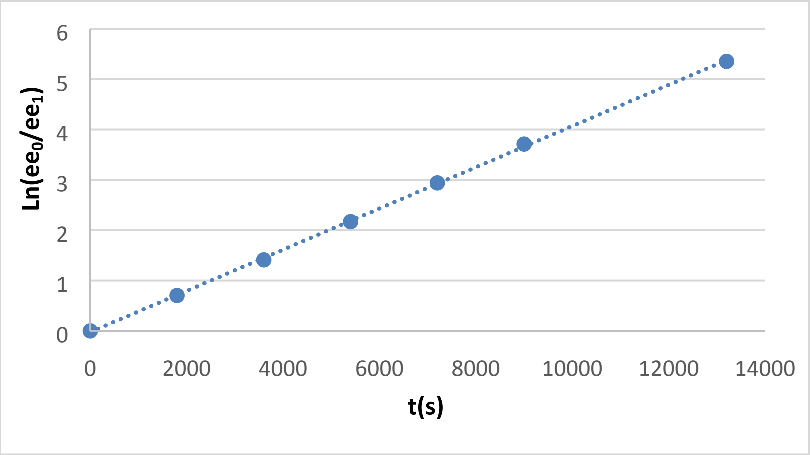

$k_{racemization} = 4.09 \times 10^{-4}$

$k_{enantiomerization} = 2.05 \times 10^{-4}$

$\Delta G^{\pm} = 29.074$ kcal/mol

8. Mechanistic proposal for the DDQ-mediated oxidation

Mechanistic proposal for the DDQ-mediated oxidation step

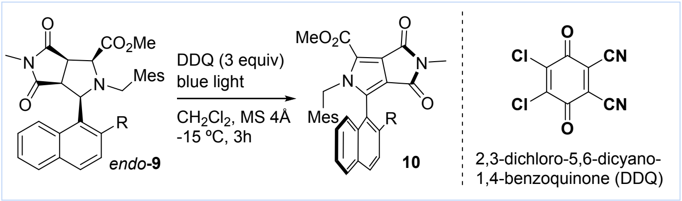

Initiates with a single electron transfer from the substrate to DDQ to form the radical cation of the substrate

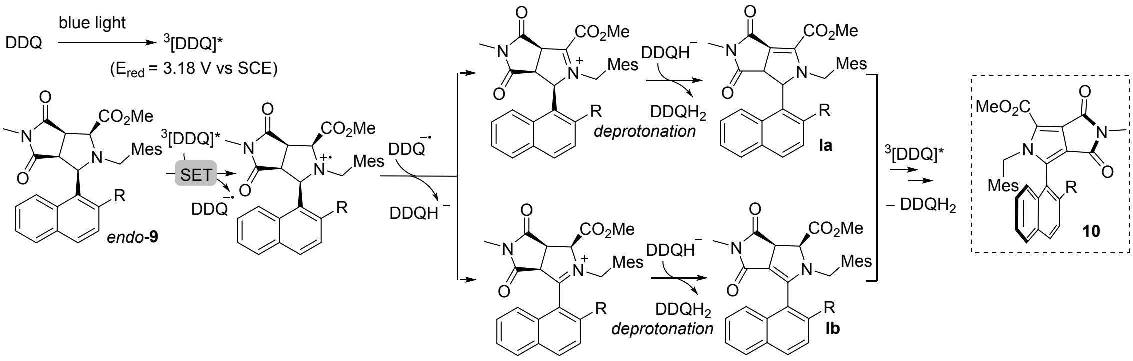

Different mechanistic pathways have been postulated in the literature for the DDQ-mediated oxidation depending on the substrates and reaction conditions. Under our reaction conditions, irradiating with blue light, the preferred pathway would be an electron transfer from the substrates to DDQ to form the radical cation of the substrate[8]. The one-electron oxidizing capacity of DDQ improves significantly by visible-light excitation from 0.51 V *vs* SCE to 3.18 V *vs* SCE by excitation to its triplet (^{3}DDQ*) (3.8 V *vs* SCE) by singlet-excitation (^{1}DDQ*)[9]. The radical cation is then transformed into iminium cation by a proton abstraction mediated by $[DDQ]^{\cdot-}$ and a single-electron-transfer step. Then, deprotonation mediated by $[DDQ]^-$ would generate intermediate **Ia**, or **Ib**, and $DDQH_2$. Finally, reiteration of the process would lead to the corresponding pyrrole derivative.

9. HPLC charts

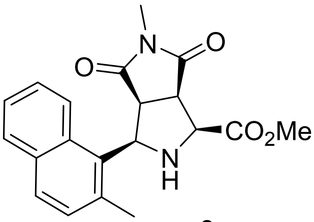

3a

(±)-3a

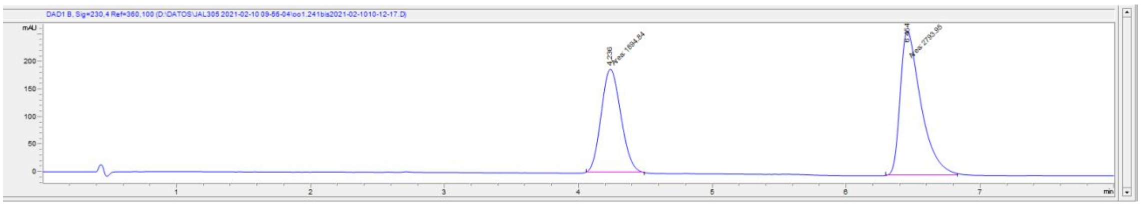

#	Time	Type	Area	Height	Width	Area%	Symmetry
1	4.236	MM	1894.8	186.5	0.1693	40.412	0.854
2	6.454	MM	2793.9	261	0.1784	59.588	0.513

(+)-3a; 87% *ee*

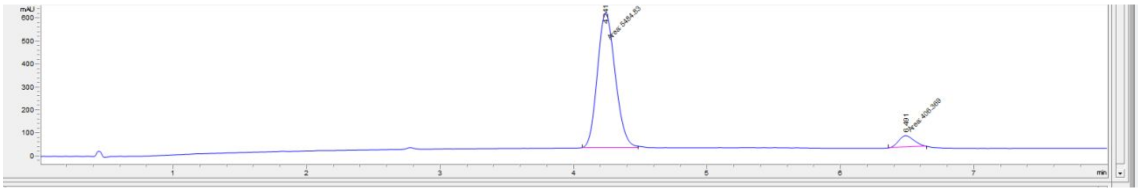

#	Time	Type	Area	Height	Width	Area%	Symmetry
1	4.241	MM	5484.8	587	0.1557	93.102	0.836
2	6.491	MM	406.4	50	0.1354	6.898	0.769

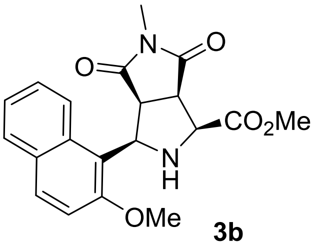

3b

(±)-3b

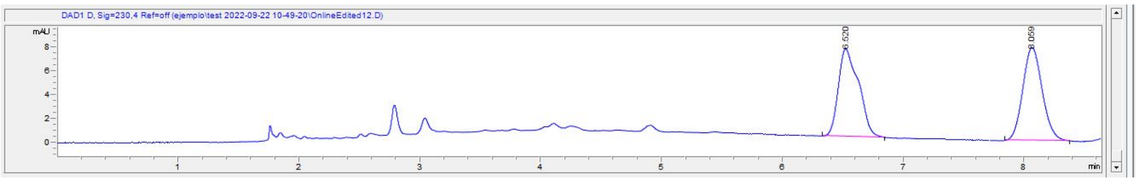

#	Time	Area	Height	Width	Area%	Symmetry
1	6.52	85.8	7.3	0.1519	49.621	0.557
2	8.059	87.1	7.8	0.1353	50.379	0.83

(+)-3b; >99% *ee*

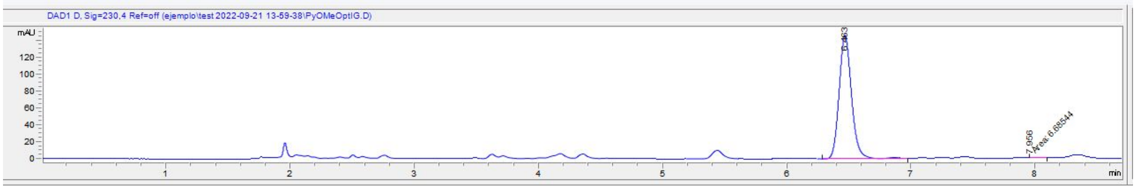

#	Time	Area	Height	Width	Area%	Symmetry
1	6.463	1029.3	147.7	0.106	99.355	0.843
2	7.956	6.7	8.4E-1	0.1329	0.645	1.36E-2

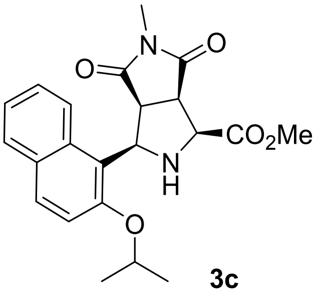

3c

(±)-3c

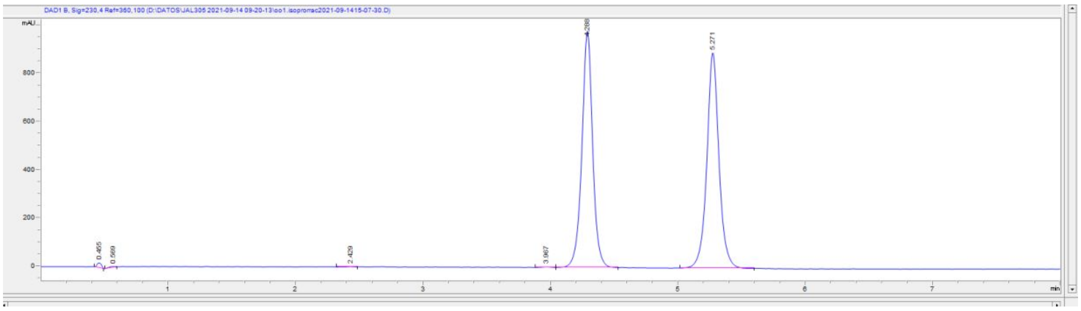

(+)-3c; 90% *ee*

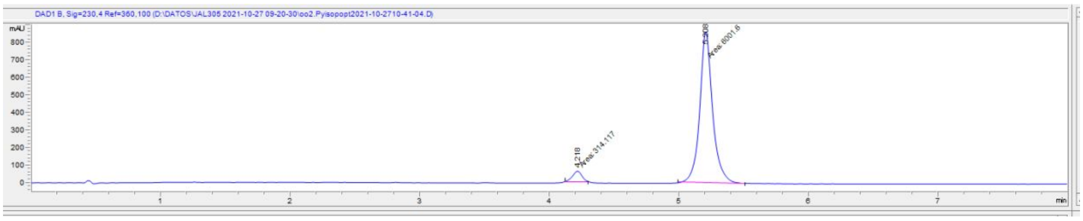

#	Time	Type	Area	Height	Width	Area%	Symmetry
1	4.218	MM	314.1	61.4	0.0852	4.974	1.11
2	5.208	MM	6001.6	864.3	0.1157	95.026	0.846

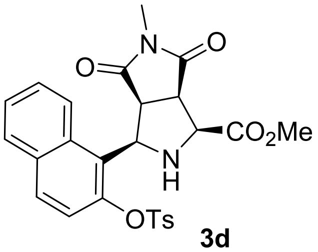

3d

(±)-3d

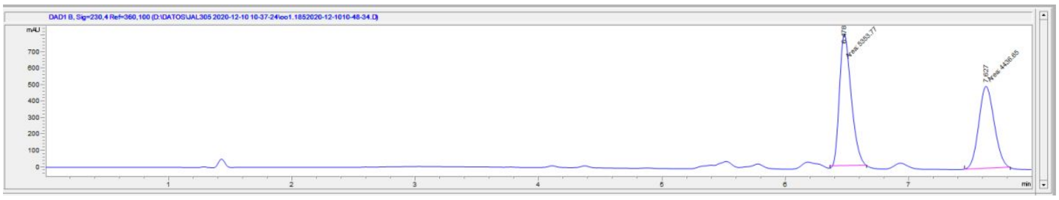

#	Time	Type	Area	Height	Width	Area%	Symmetry
1	6.478	MM	5353.8	809	0.1103	54.684	0.685
2	7.627	MM	4436.6	505.6	0.1462	45.316	0.868

(+)-3d; 97% *ee*

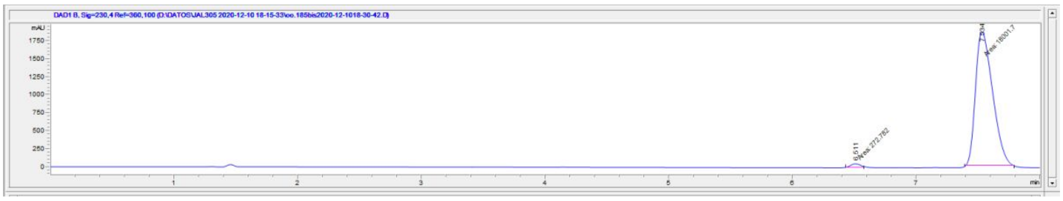

#	Time	Type	Area	Height	Width	Area%	Symmetry
1	6.511	MM	272.8	50.3	0.0904	1.493	0.919
2	7.534	MM	18001.7	1874.6	0.16	98.507	0.589

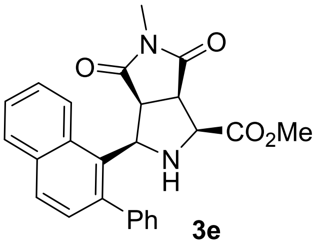

3e

(±)-3e

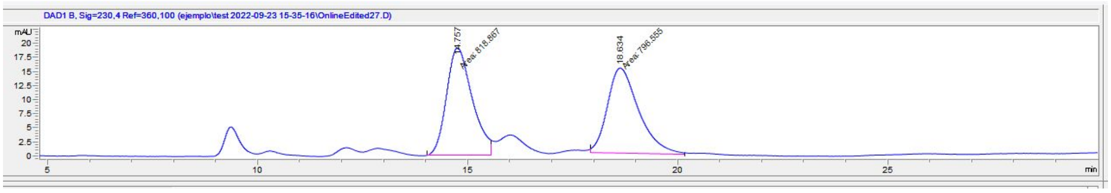

#	Time	Area	Height	Width	Area%	Symmetry
1	14.757	818.9	19.1	0.7148	50.691	0.741
2	18.634	796.6	15.2	0.8758	49.309	0.683

(+)-3e; 84% *ee*

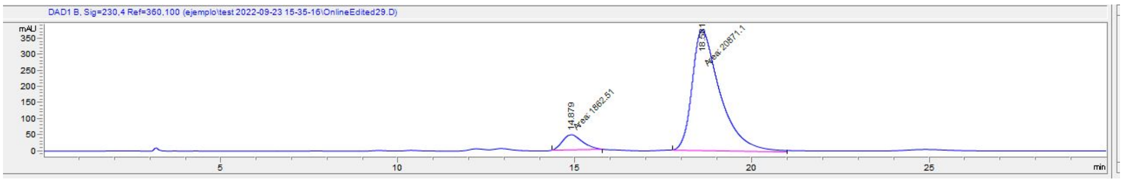

#	Time	Area	Height	Width	Area%	Symmetry
1	14.879	1862.5	47.4	0.6553	8.193	0.753
2	18.581	20871.1	376.5	0.9239	91.807	0.541

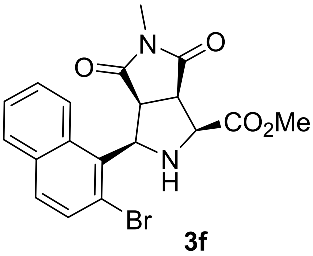

3f

(±)-3f

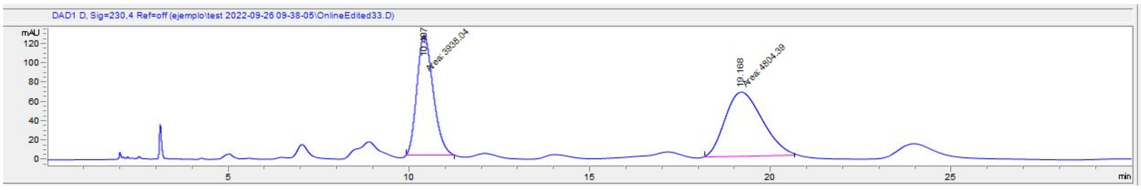

#	Time	Area	Height	Width	Area%	Symmetry
1	10.407	3938	125.2	0.524	45.045	0.769
2	19.168	4804.4	67	1.1954	54.955	0.714

(+)-3f; 89% *ee*

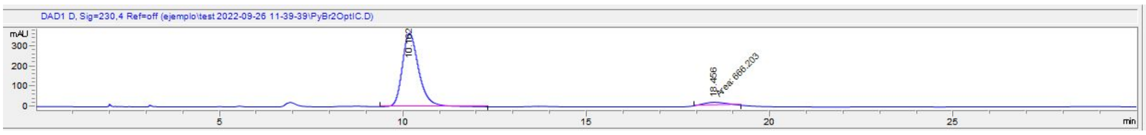

#	Time	Area	Height	Width	Area%	Symmetry
1	10.162	11487	366.6	0.4596	94.518	0.713
2	18.456	666.2	15.6	0.7127	5.482	0.844

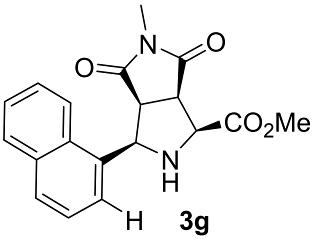

3g

(±)-3g

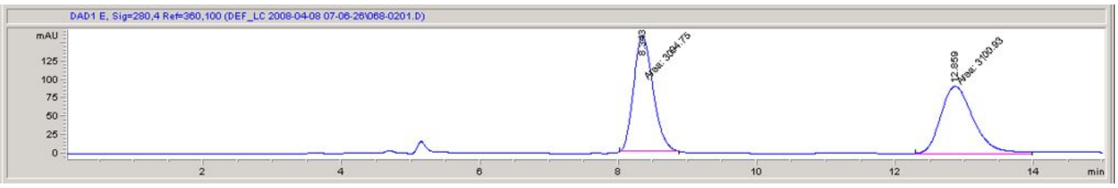

#	Time	Area	Height	Width	Area%	Symmetry
1	8.343	3094.7	157.9	0.3266	49.950	0.798
2	12.859	3100.9	92.4	0.5594	50.050	0.74

(+)-3g; 99% *ee*

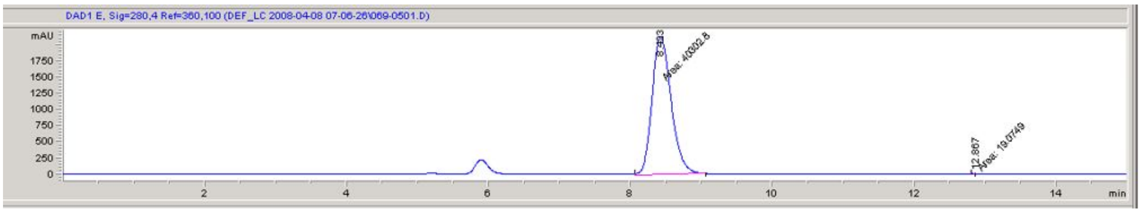

#	Time	Area	Height	Width	Area%	Symmetry
1	8.423	40302.8	2142.1	0.3136	99.953	0.786
2	12.867	19.1	12.5	0.0254	0.047	0

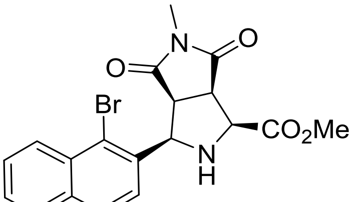

11a

(±)-11a

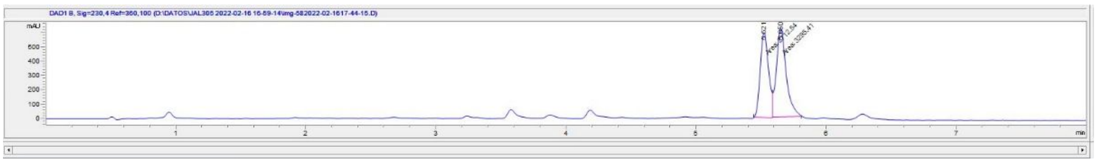

#	Time	Type	Area	Height	Width	Area%	Symmetry
1	5.521	MM	2712.5	598.5	0.0755	45.149	0.803
2	5.65	MM	3295.4	628.6	0.0874	54.851	0.743

(+)-11a; 99% *ee*

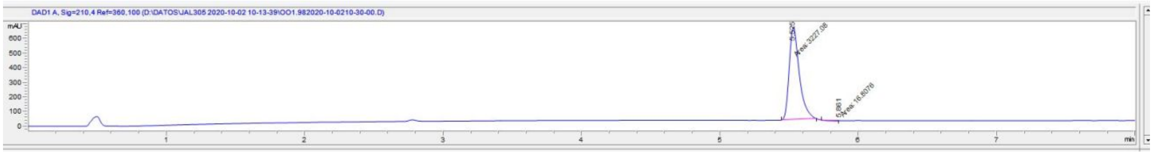

#	Time	Type	Area	Height	Width	Area%	Symmetry
1	5.525	MM	3227.1	636.2	0.0845	99.482	0.667
2	5.861	MM	16.8	3.6	0.0777	0.518	0

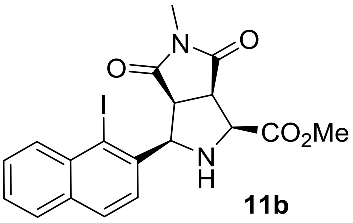

11b

(±)-11b

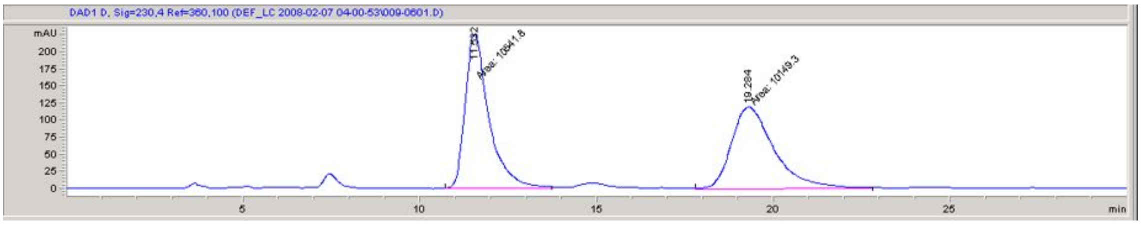

#	Time	Area	Height	Width	Area%	Symmetry
1	11.532	10541.8	225	0.7807	50.948	0.616
2	19.284	10149.3	119.9	1.4108	49.052	0.651

(+)-11b; 99% *ee*

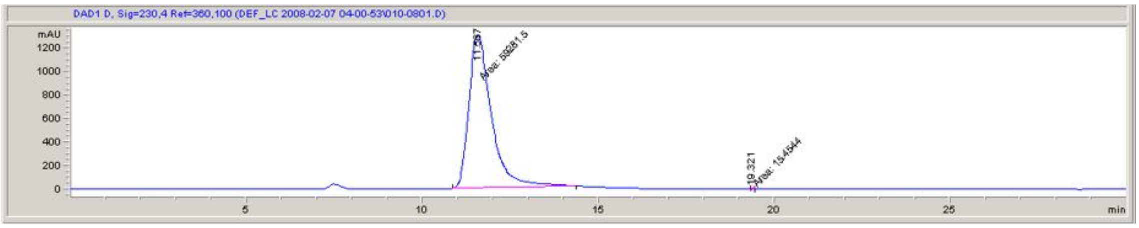

#	Time	Area	Height	Width	Area%	Symmetry
1	11.567	59281.5	1301	0.7594	99.974	0.611
2	19.321	15.5	15.1	0.017	0.026	0

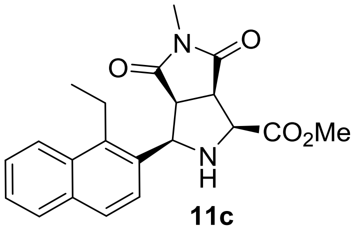

11c

(±)-11c

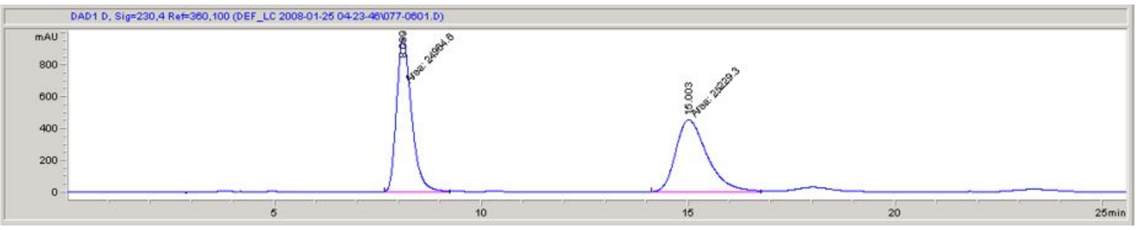

#	Time	Area	Height	Width	Area%	Symmetry
1	8.099	24964.6	968.9	0.4294	49.736	0.709
2	15.003	25229.3	461.4	0.9113	50.264	0.667

(+)-11c; 99% *ee*

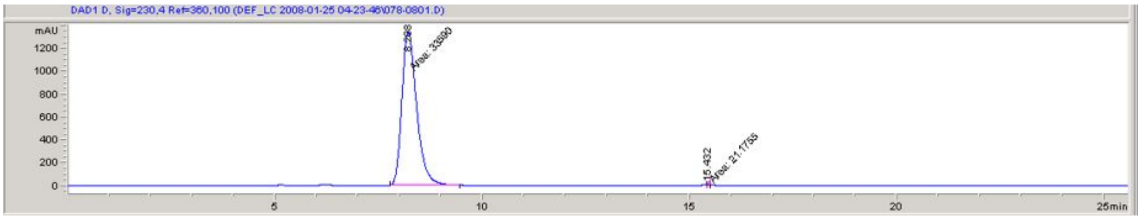

#	Time	Area	Height	Width	Area%	Symmetry
1	8.208	33590	1342.8	0.4169	99.937	0.705
2	15.432	21.2	33.6	0.0105	0.063	0

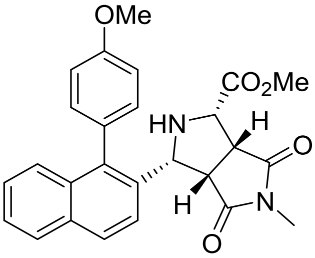

11d

(±)-11d

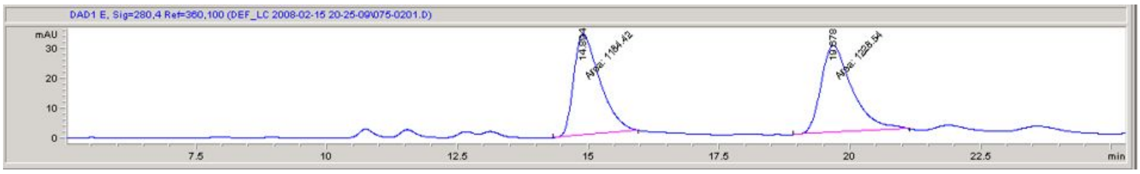

#	Time	Area	Height	Width	Area%	Symmetry
1	14.894	1184.4	34	0.5809	49.086	0.541
2	19.678	1228.5	29.3	0.6985	50.914	0.618

(+)-11d; 99% *ee*

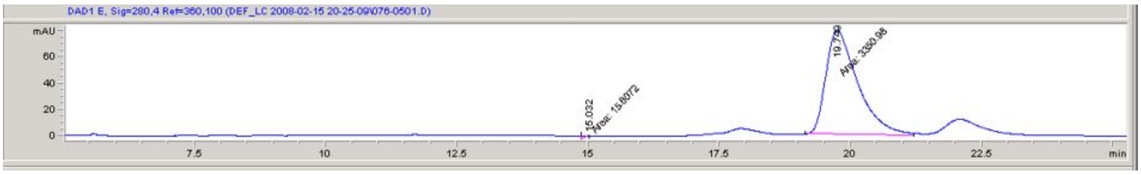

#	Time	Area	Height	Width	Area%	Symmetry
1	15.032	15.6	4.1E-1	0.4422	0.464	2.846
2	19.749	3351	79.2	0.7055	99.536	0.535

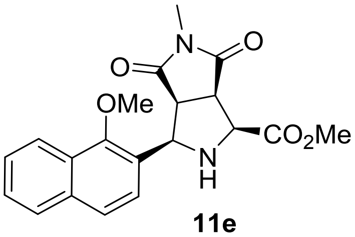

11e

(±)-11e

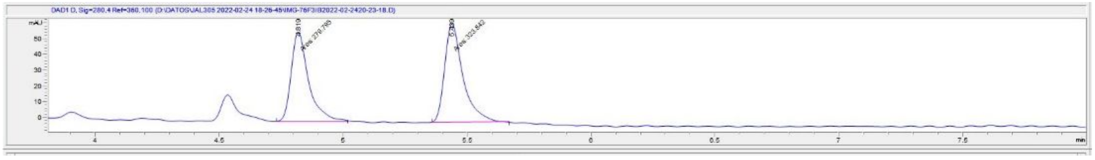

#	Time	Type	Area	Height	Width	Area%	Symmetry
1	4.819	MM	279.8	57.2	0.0816	46.352	0.653
2	5.439	MM	323.8	62.6	0.0862	53.648	0.637

(+)-11e; 95% *ee*

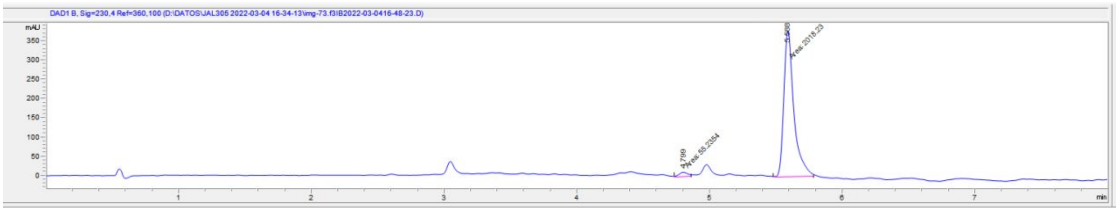

#	Time	Type	Area	Height	Width	Area%	Symmetry
1	4.799	MM	55.2	11.3	0.0814	2.664	0.84
2	5.588	MM	2018.2	382.4	0.088	97.336	0.654

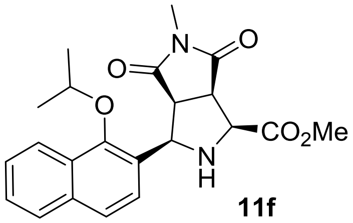

11f

(±)-11f

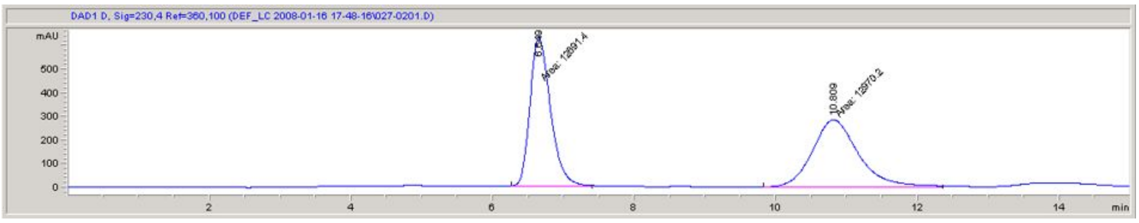

#	Time	Area	Height	Width	Area%	Symmetry
1	6.649	12691.4	630.1	0.3357	49.457	0.73
2	10.809	12970.2	284.8	0.7589	50.543	0.809

(+)-11f; 98% *ee*

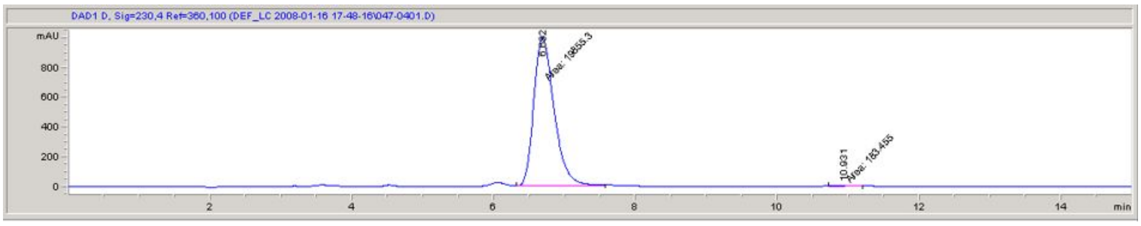

#	Time	Area	Height	Width	Area%	Symmetry
1	6.682	19855.3	998	0.3316	99.085	0.711
2	10.931	183.5	8.8	0.3083	0.915	1.625

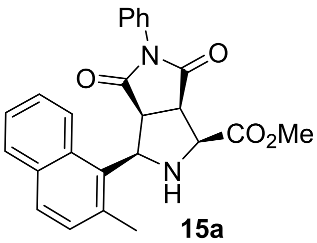

15a

(±)-15a

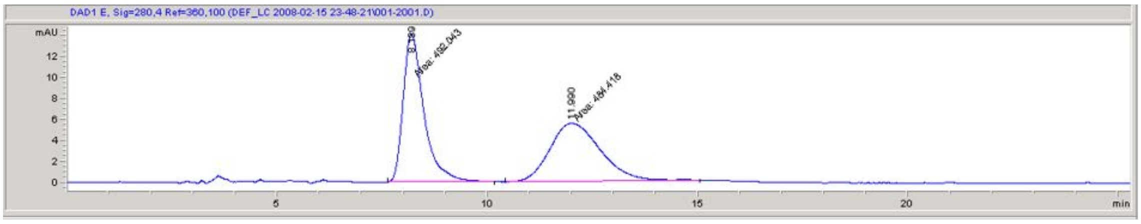

#	Time	Area	Height	Width	Area%	Symmetry
1	8.189	492	14.2	0.5783	50.390	0.64
2	11.99	484.4	5.6	1.4435	49.610	0.707

(+)-15a; 99% *ee*

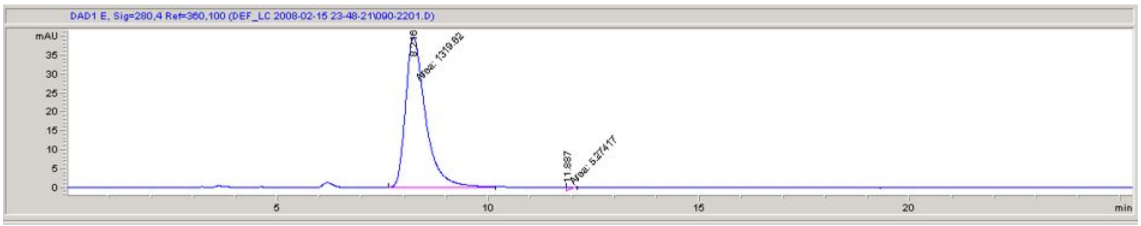

#	Time	Area	Height	Width	Area%	Symmetry
1	8.216	1319.6	39.7	0.5534	99.602	0.622
2	11.887	5.3	7.9E-1	0.085	0.398	0.183

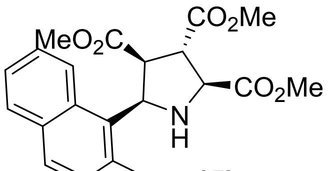

15b

(±)-15b

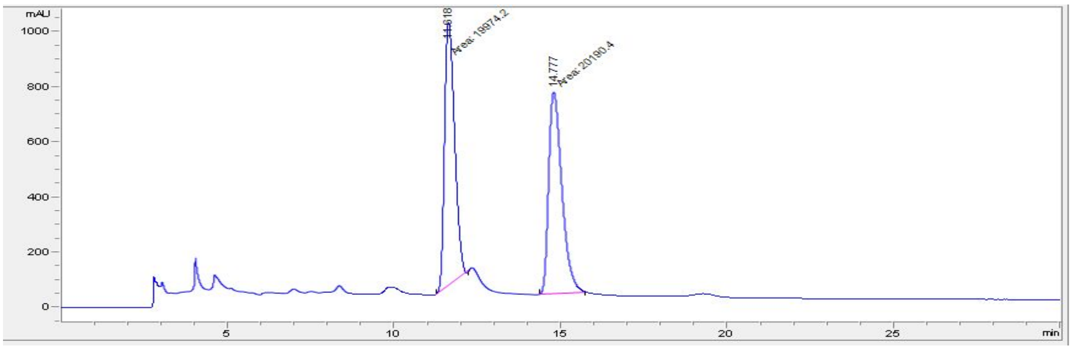

#	Time	Area	Height	Width	Area%	Symmetry
1	11.618	19974.2	951.1	0.35	49.731	0.639
2	14.777	20190.4	731.8	0.4598	50.269	0.599

(+)-15b; 88% *ee*

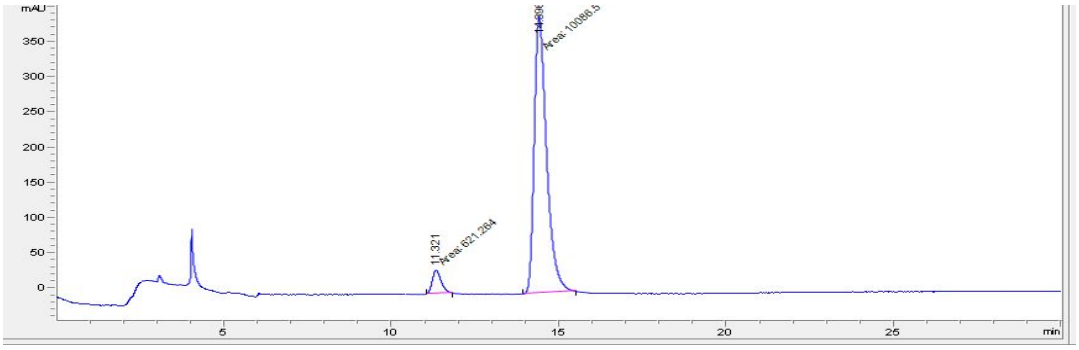

#	Time	Area	Height	Width	Area%	Symmetry
1	11.321	621.3	33.5	0.3094	5.802	0.757
2	14.396	10086.5	391.2	0.4297	94.198	0.633

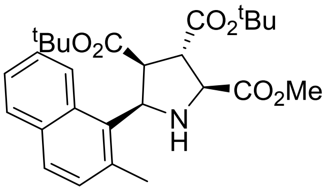

15c

(±)-15c

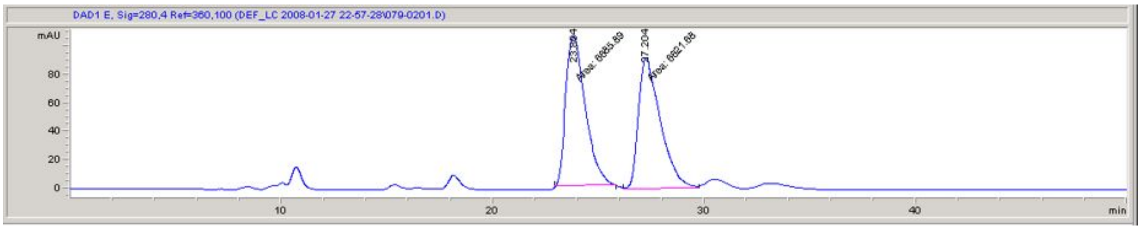

#	Time	Area	Height	Width	Area%	Symmetry
1	23.804	6685.9	104	1.0717	50.241	0.636
2	27.204	6621.7	91.8	1.2021	49.759	0.464

(+)-15c; 81% *ee*

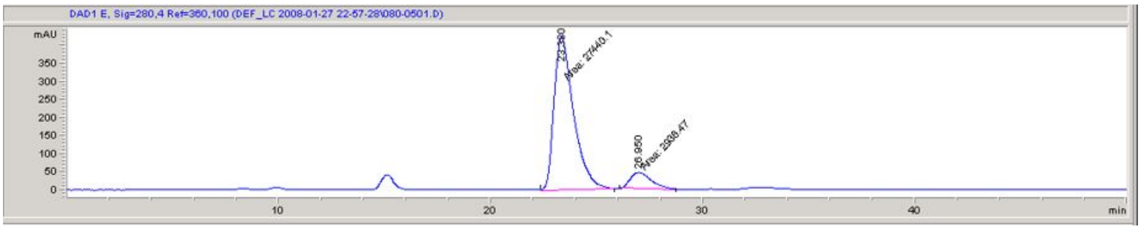

#	Time	Area	Height	Width	Area%	Symmetry
1	23.33	27440.1	426.7	1.0717	90.333	0.613
2	26.95	2936.5	43.3	1.1297	9.667	0.558

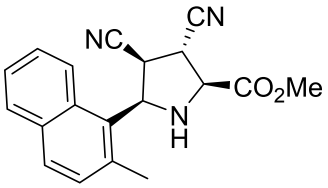

15d

(±)-15d

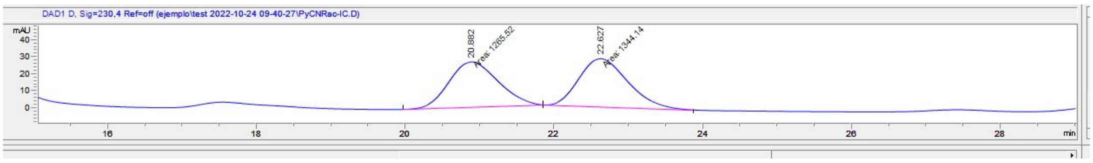

#	Time	Area	Height	Width	Area%	Symmetry
1	20.882	1265.5	27.1	0.779	48.494	0.801
2	22.627	1344.1	28.8	0.7792	51.506	0.757

(+)-15d; 98% *ee*

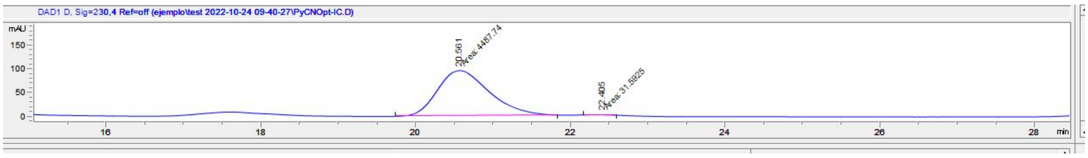

#	Time	Area	Height	Width	Area%	Symmetry
1	20.561	4487.7	95.8	0.7809	99.301	0.691
2	22.405	31.6	1.5	0.3548	0.699	0.78

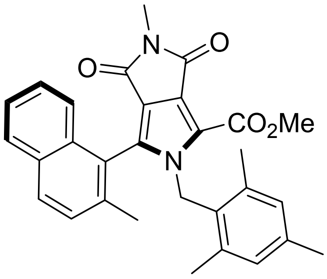

9a

(±)-9a

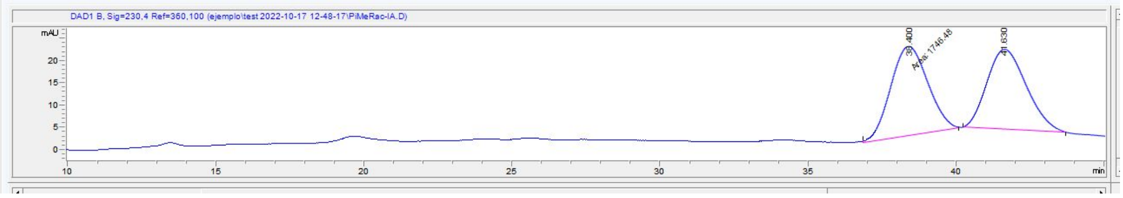

#	Time	Area	Height	Width	Area%	Symmetry
1	38.4	1746.5	20	1.455	51.397	0.995
2	41.63	1651.5	17.9	1.0792	48.603	0.81

(+)-9a; 84% *ee*

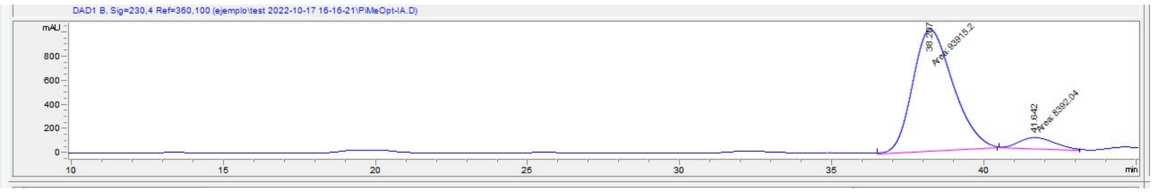

#	Time	Area	Height	Width	Area%	Symmetry
1	38.207	93915.2	1013	1.5452	91.797	0.76
2	41.642	8392	97.2	1.4388	8.203	0.743

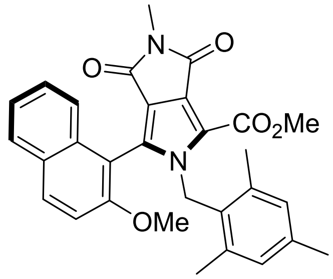

9b

(±)-9b

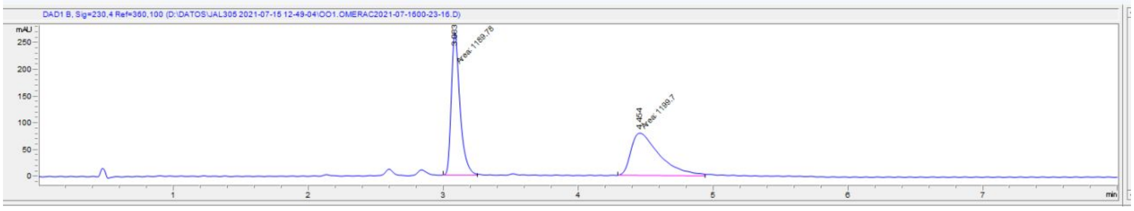

#	Time	Type	Area	Height	Width	Area%	Symmetry
1	3.083	MM	1189.8	267.8	0.0741	49.792	0.675
2	4.454	MM	1199.7	79.2	0.2523	50.208	0.417

(+)-9b; 92% *ee*

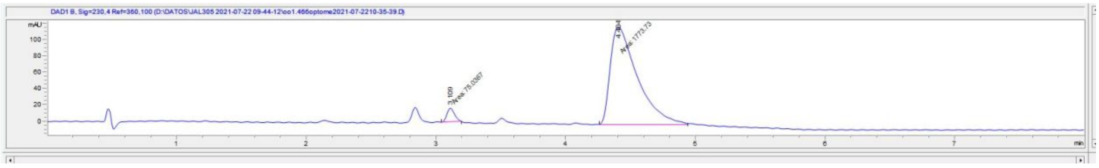

#	Time	Type	Area	Height	Width	Area%	Symmetry
1	3.109	MM	75	17.7	0.0705	4.059	0.797
2	4.404	MM	1773.7	120.7	0.2449	95.941	0.405

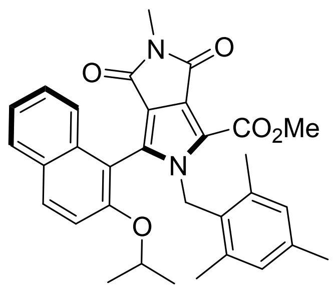

9c

(±)-9c

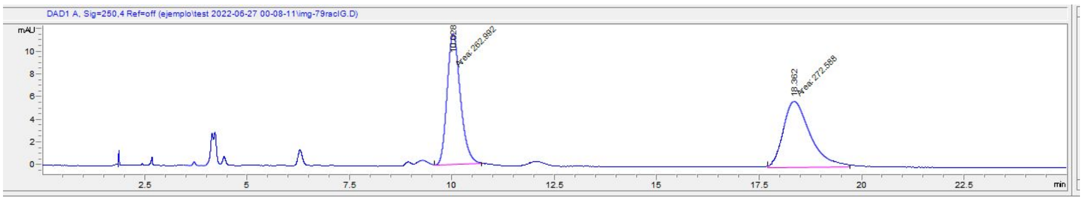

#	Time	Area	Height	Width	Area%	Symmetry
1	10.028	263	11.6	0.3765	49.104	0.706
2	18.362	272.6	5.9	0.7709	50.896	0.672

(-)-9c; 85% *ee*

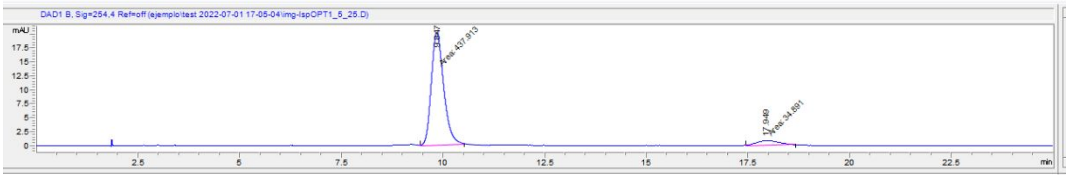

#	Time	Area	Height	Width	Area%	Symmetry
1	9.847	437.9	20.4	0.357	92.620	0.77
2	17.949	34.9	8.9E-1	0.6541	7.380	0.779

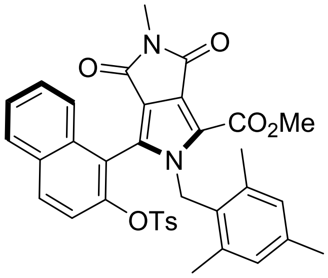

9d

(±)-9d

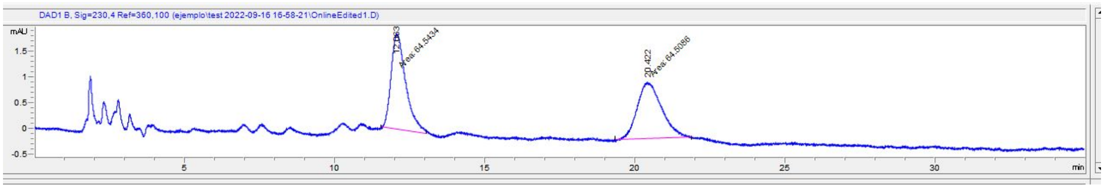

#	Time	Area	Height	Width	Area%	Symmetry
1	12.063	64.5	1.9	0.5714	50.013	0.579
2	20.422	64.5	1.1	0.9512	49.987	0.69

(-)-9d; 97% *ee*

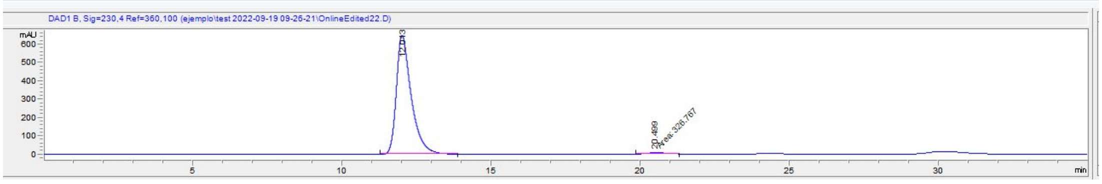

#	Time	Area	Height	Width	Area%	Symmetry
1	12.013	22367.2	643.5	0.49	98.560	0.585
2	20.499	326.8	7.2	0.7598	1.440	0.894

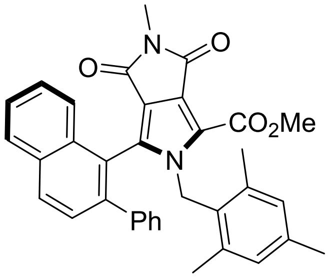

9e

(±)-9e

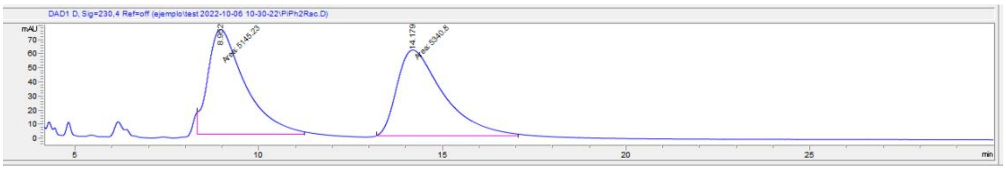

#	Time	Area	Height	Width	Area%	Symmetry
1	8.952	5145.2	73.8	1.1627	49.067	0.495
2	14.179	5340.8	60.5	1.471	50.933	0.481

(+)-9e; 70% *ee*

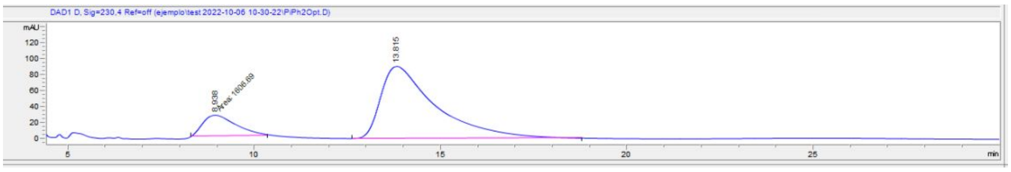

#	Time	Area	Height	Width	Area%	Symmetry
1	8.938	1606.7	26.1	1.0242	14.925	0.584
2	13.815	9158.7	89.5	1.2023	85.075	0.397

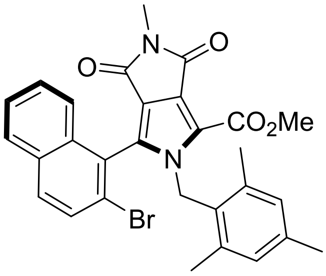

9f

(±)-9f

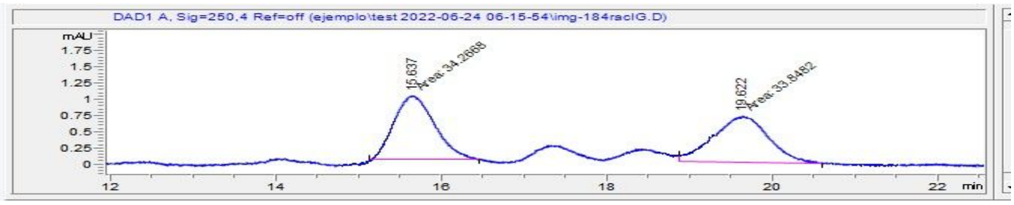

#	Time	Area	Height	Width	Area%	Symmetry
1	15.637	34.3	1E0	0.5725	50.307	0.788
2	19.622	33.8	7.3E-1	0.7704	49.693	1.054

(+)-9f; >99% *ee*

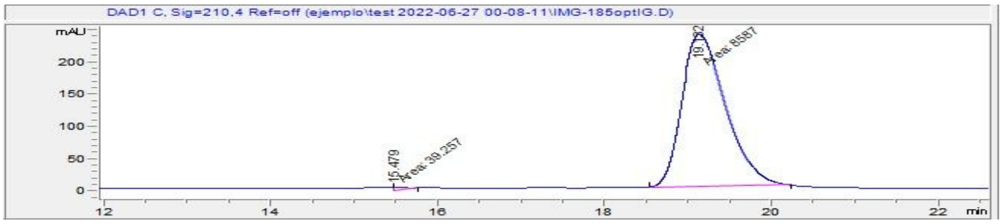

#	Time	Area	Height	Width	Area%	Symmetry
1	15.479	39.3	5.1	0.0906	0.455	0
2	19.132	8587	236	0.6064	99.545	0.675

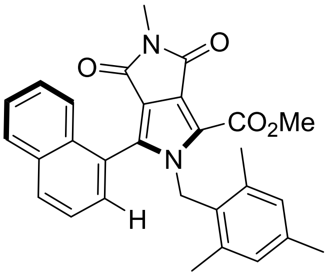

9g

(±)-9g

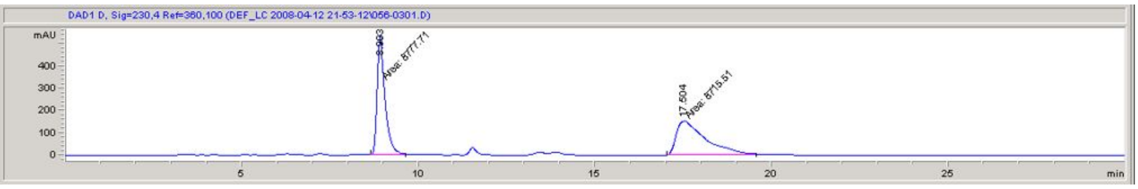

#	Time	Area	Height	Width	Area%	Symmetry
1	8.903	8777.7	540.1	0.2709	50.178	0.569
2	17.504	8715.5	150.5	0.9655	49.822	0.315

(±)-9g; 0% *ee*

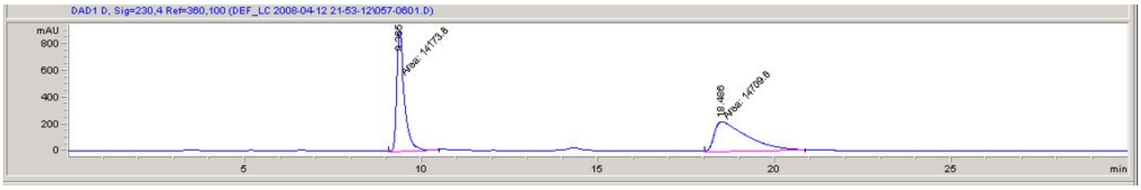

#	Time	Area	Height	Width	Area%	Symmetry
1	9.365	14173.8	907.2	0.2604	49.072	0.503
2	18.486	14709.6	223.4	1.0973	50.928	0.261

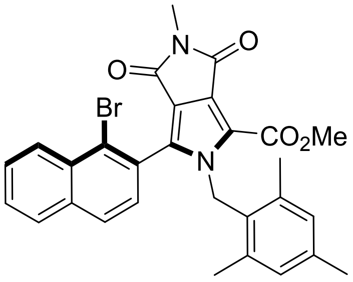

13a

(±)-13a

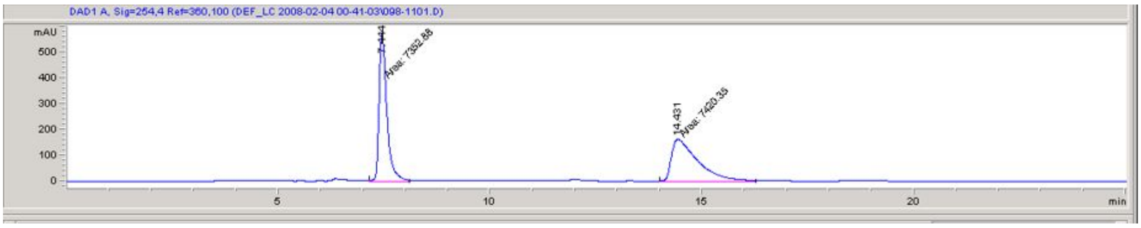

#	Time	Area	Height	Width	Area%	Symmetry
1	7.444	7352.9	574.7	0.2132	49.772	0.577
2	14.431	7420.3	165.7	0.7464	50.228	0.322

(+)-13a; 90% *ee*

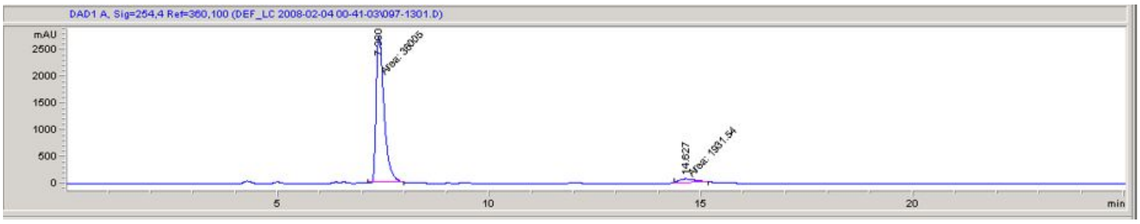

#	Time	Area	Height	Width	Area%	Symmetry
1	7.38	36005	2749.5	0.2183	94.909	0.516
2	14.627	1931.5	67.4	0.4778	5.091	0.604

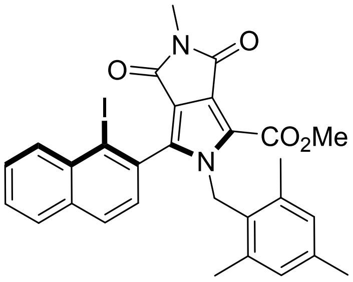

13b

(±)-13b

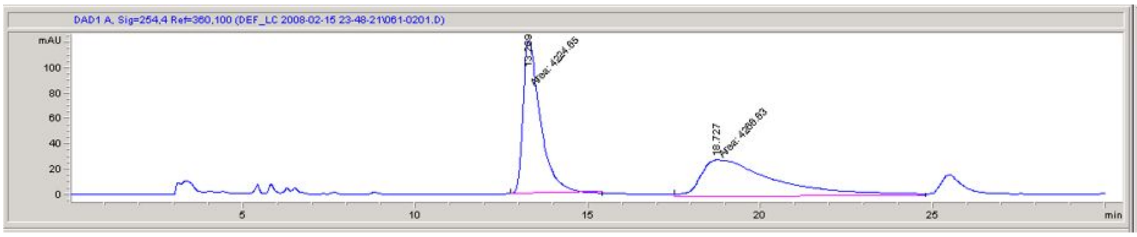

#	Time	Area	Height	Width	Area%	Symmetry
1	13.269	4224.6	119.9	0.5871	49.753	0.47
2	18.727	4266.6	28.7	2.4751	50.247	0.28

(+)-13b; 99% *ee*

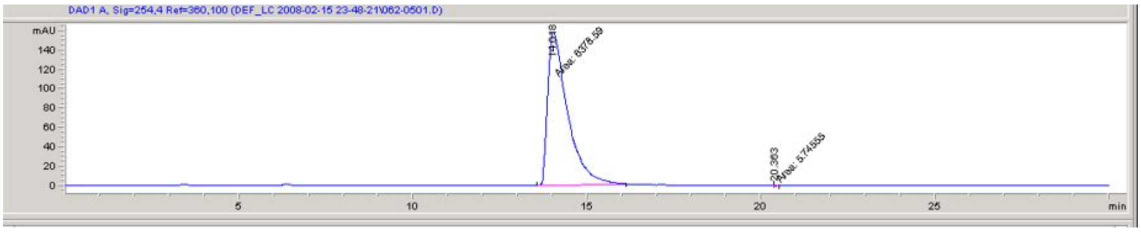

#	Time	Area	Height	Width	Area%	Symmetry
1	14.018	6378.6	159.4	0.6671	99.910	0.379
2	20.363	5.7	2.1	0.0447	0.090	0

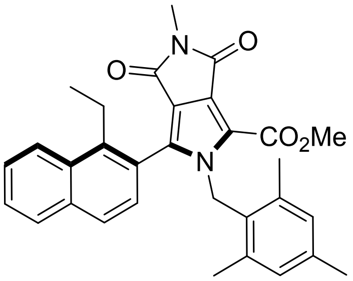

13c

(±)-13c

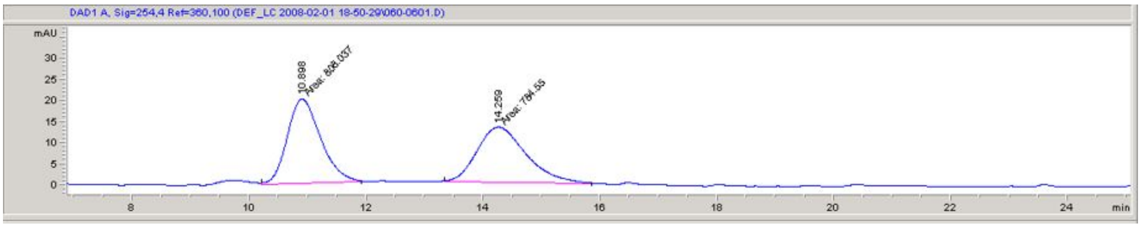

#	Time	Area	Height	Width	Area%	Symmetry
1	10.898	808	20.1	0.6694	50.737	0.768
2	14.259	784.6	13.2	0.9924	49.263	0.743

(+)-13c; 99% *ee*

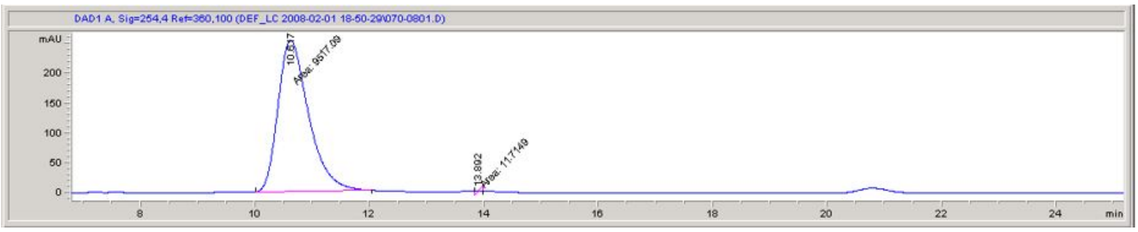

#	Time	Area	Height	Width	Area%	Symmetry
1	10.617	9517.1	254.2	0.624	99.877	0.666
2	13.892	11.7	1.4	0.1347	0.123	37.009

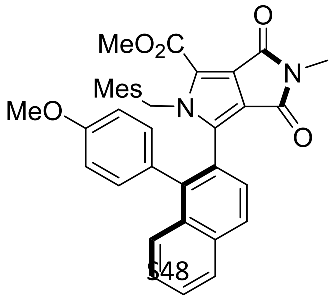

(±)-13d

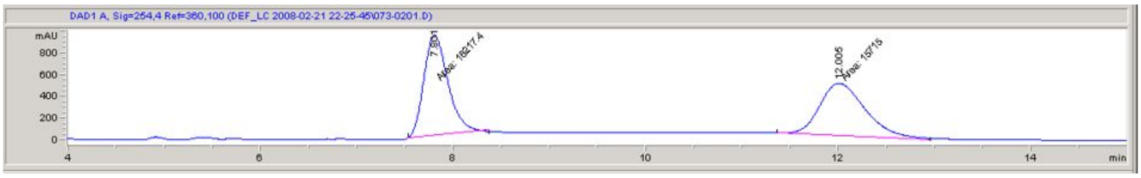

#	Time	Area	Height	Width	Area%	Symmetry
1	7.801	16217.4	928.2	0.2912	50.787	0.755
2	12.005	15715	478.3	0.5476	49.213	0.699

(±)-13c; 0% *ee*

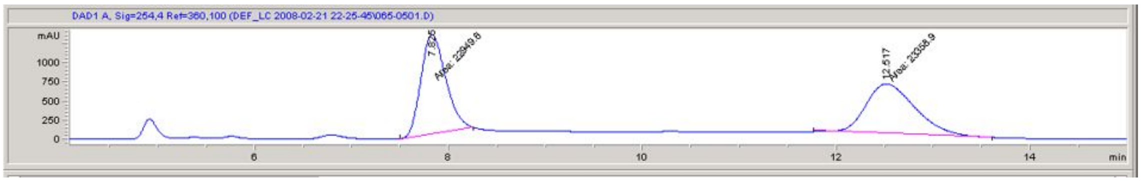

#	Time	Area	Height	Width	Area%	Symmetry
1	7.825	22949.6	1292.4	0.296	49.558	0.794
2	12.517	23358.9	652.4	0.5967	50.442	0.664

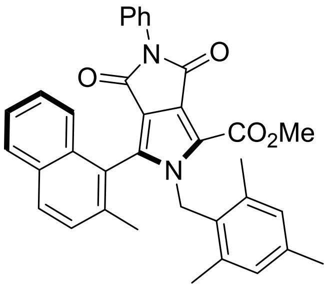

17a

(±)-17a

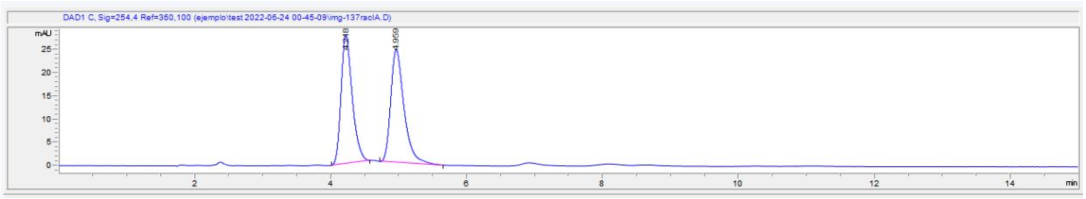

#	Time	Area	Height	Width	Area%	Symmetry
1	4.218	316.1	27.6	0.1632	48.826	0.709
2	4.959	331.3	24.3	0.1884	51.174	0.652

(+)-17a; 86% *ee*

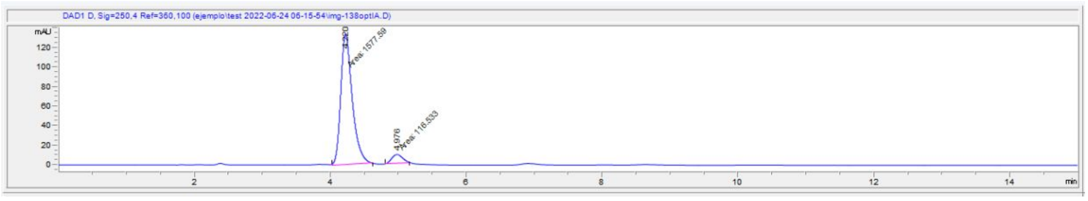

#	Time	Area	Height	Width	Area%	Symmetry
1	4.22	1577.6	134.1	0.196	93.121	0.692
2	4.976	116.5	9.8	0.1987	6.879	0.864

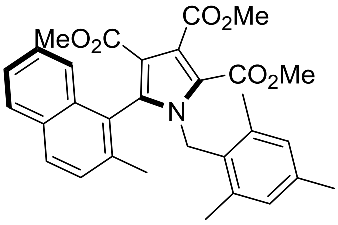

17b

(±)-17b

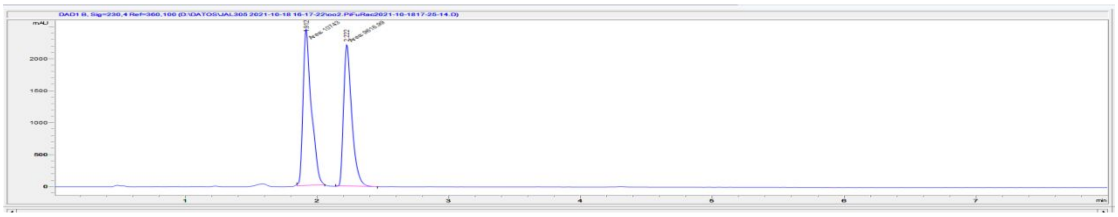

(-)-17b; 60% *ee*

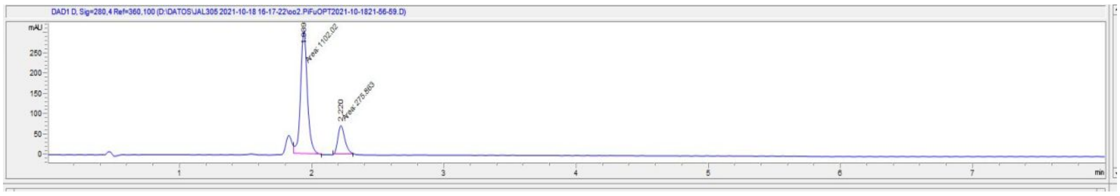

#	Time	Type	Area	Height	Width	Area%	Symmetry
1	1.939	MM	1102	308.6	0.0595	79.979	0.884
2	2.22	MM	275.9	72.8	0.0632	20.021	0.738

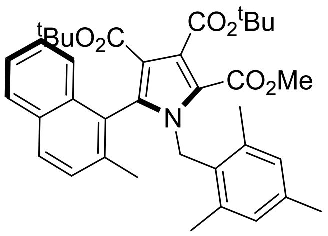

17c

(±)-17c

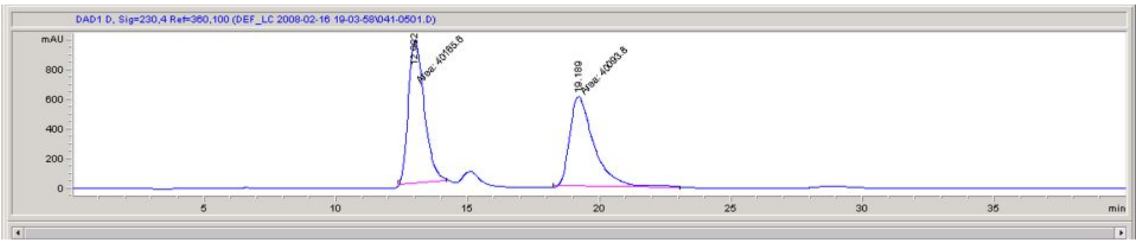

#	Time	Area	Height	Width	Area%	Symmetry
1	12.982	40165.6	960.2	0.6972	50.045	0.709
2	19.189	40093.8	606.2	1.1024	49.955	0.523

(-)-17c; 78% *ee*

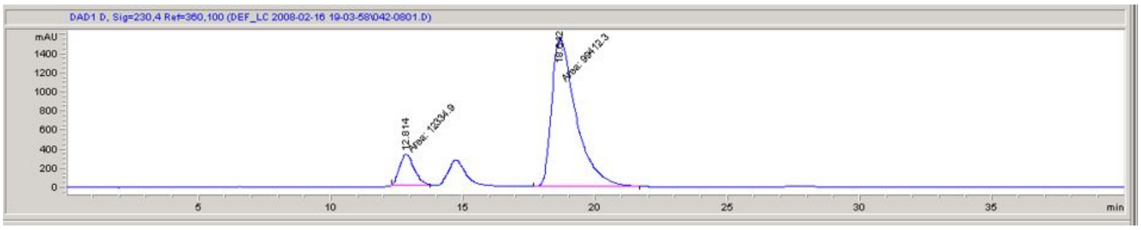

#	Time	Area	Height	Width	Area%	Symmetry
1	12.814	12334.9	321.1	0.6403	11.038	0.765
2	18.642	99412.3	1550.6	1.0686	88.962	0.504

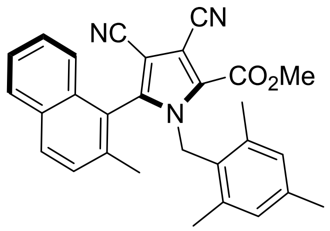

17d

(±)-17d

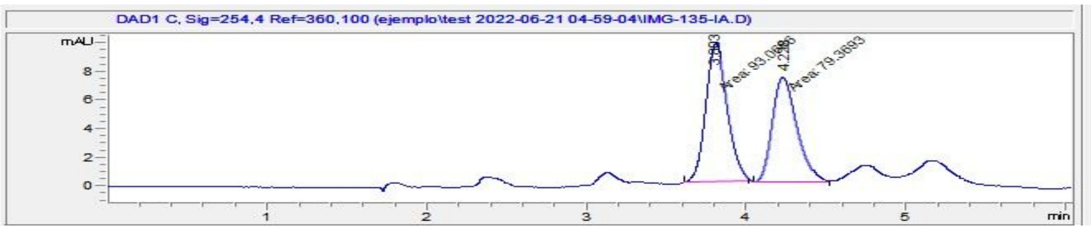

#	Time	Area	Height	Width	Area%	Symmetry
1	3.803	93.1	9.7	0.1595	53.972	0.803
2	4.228	79.4	7.4	0.179	46.028	0.742

(-)-17d; 99% *ee*

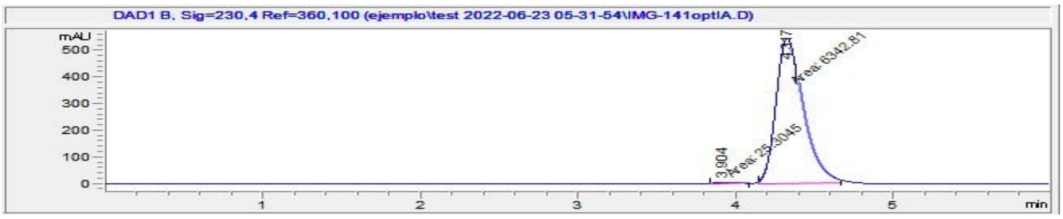

#	Time	Area	Height	Width	Area%	Symmetry
1	3.904	25.3	3.2	0.0961	0.397	1.106
2	4.317	6342.8	545.9	0.1936	99.603	0.679

10. NMR spectra collection

The chemical shifts of the solvents (used in this SI) signals observed for ^{1}H NMR and ^{13}C NMR spectra are listed in the following chart. The multiplicity is shown as 1 for singlet, 2 for doublet, etc.

Solvent	^{1}H NMR Chemical shift (ppm)	^{13}C NMR Chemical shift (ppm)
**Chloroform**	7.26 (1)	77.2 (3)

In the following table are the chemical shift of different solvents and grease in chloroform-d. The multiplicity is shown as bs for broad signal, s for singlet, q for quartet, t for triplet and m for multiplet.

Solvent	^{1}H NMR Chemical shift (ppm)	^{13}C NMR Chemical shift (ppm)
**Water**	1.56 (bs)	-
**Acetone**	2.17 (s)	207.0 and 30.9
**Ethyl acetate**	2.05 (s); 4.12 (q); 1.26 (t)	171.4, 60.5, 21.0 and 14.2
***n*-hexane**	0.88 (t); 1.26 (m)	31.6, 22.7 and 14.1
**Dichloromethane**	5.30 (s)	53.5
**H Grease**	0.84 – 0.87 (m); 1.27 (bs)	29.8

Methyl (*E*)-2-(((2-methylnaphthalen-1-yl)methylene)amino)acetate (1a)

^{1}H NMR ($CDCl_3$, 300 MHz)

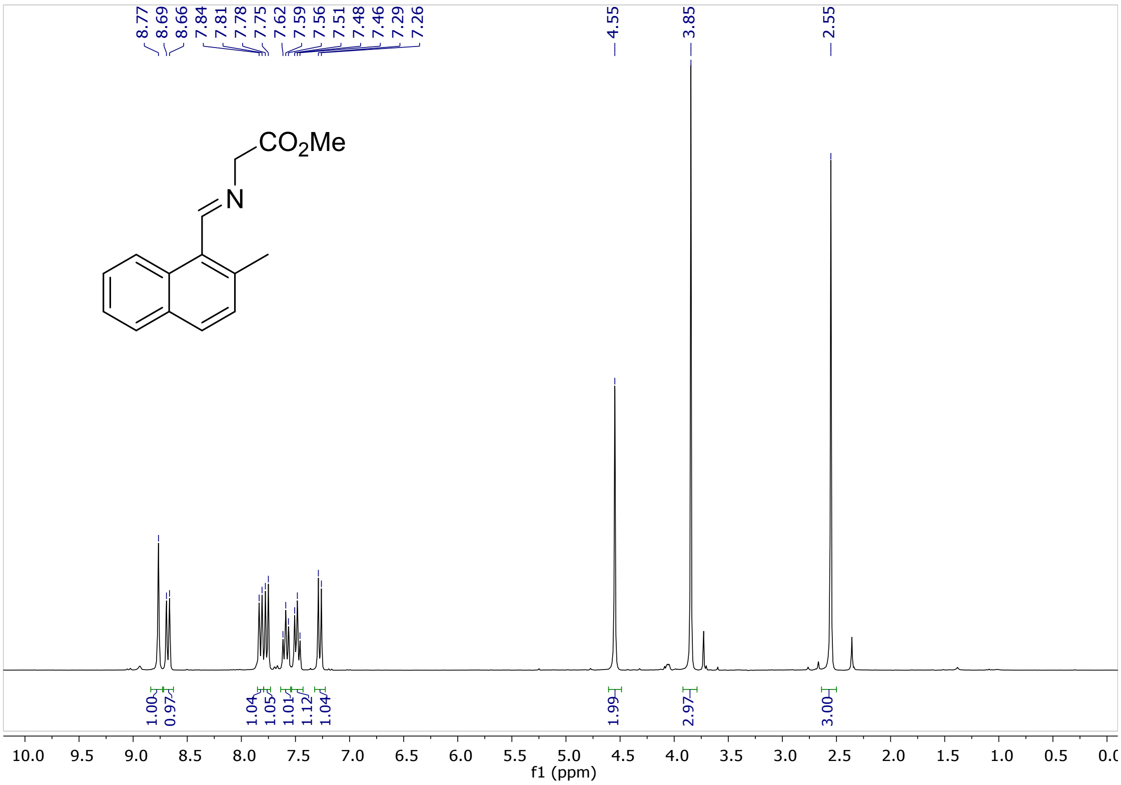

Methyl (*E*)-2-(((2-methoxynaphthalen-1-yl)methylene)amino)acetate (1b)

^{1}H NMR ($CDCl_3$, 300 MHz)

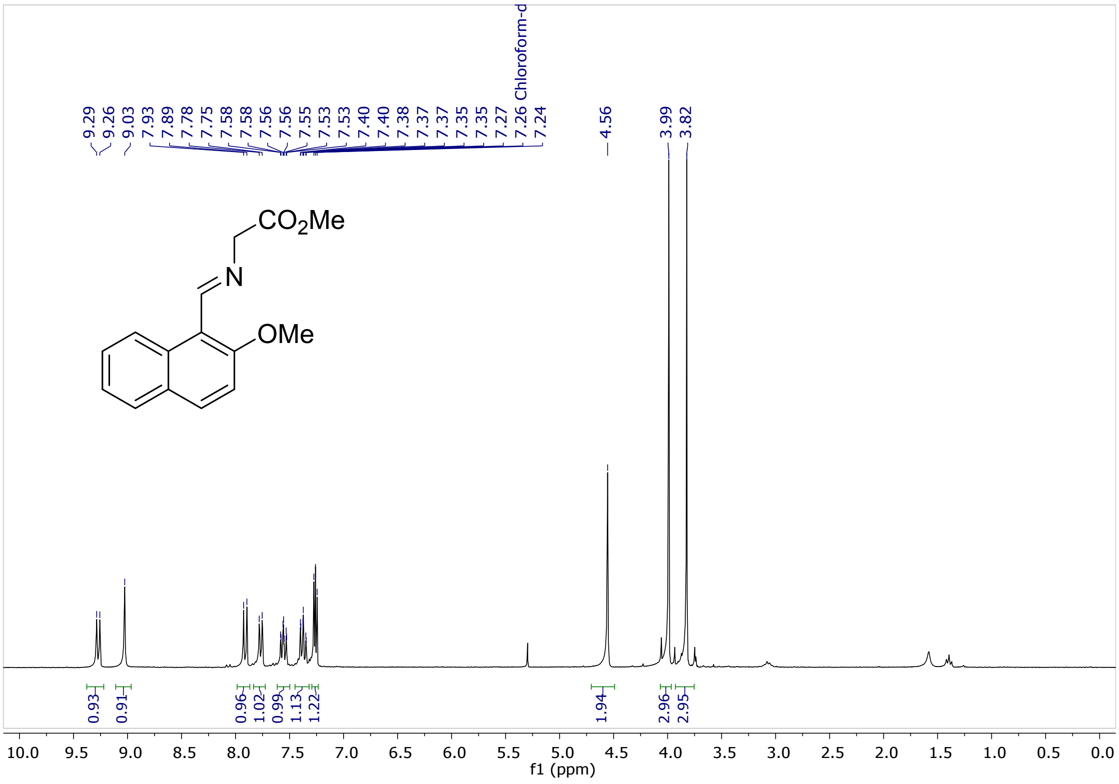

Methyl (*E*)-2-(((2-isopropoxynaphthalen-1-yl)methylene)amino)acetate (1c)

^{1}H NMR ($CDCl_3$, 300 MHz)

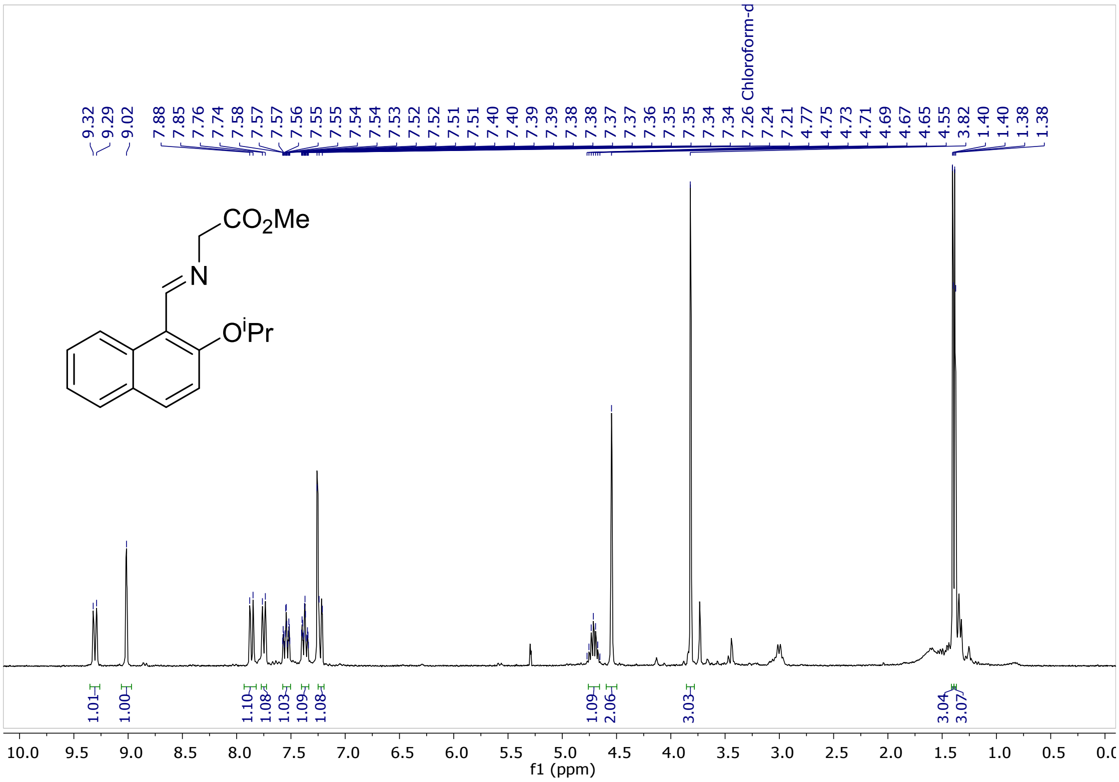

Methyl (*E*)-2-(((2-(tosyloxy)naphthalen-1-yl)methylene)amino)acetate (1d)

^{1}H NMR ($CDCl_3$, 300 MHz)

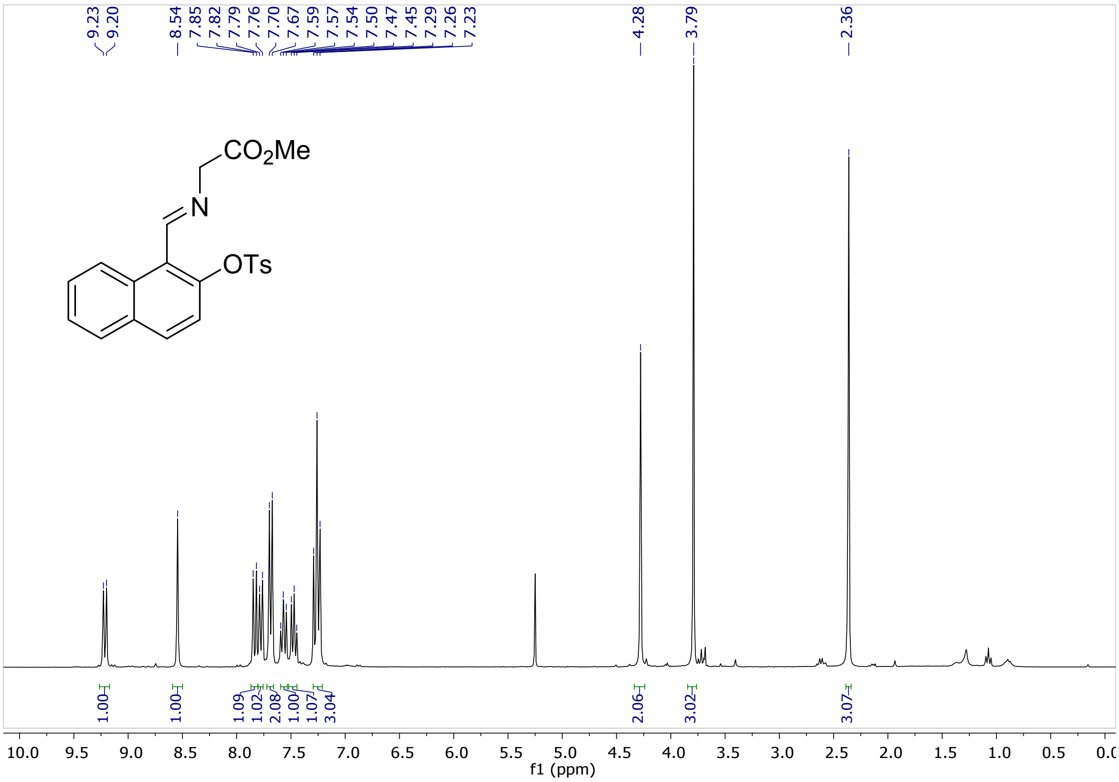

Methyl (*E*)-2-(((2-phenylnaphthalen-1-yl)methylene)amino)acetate (1e)

^{1}H NMR ($CDCl_3$, 300 MHz)

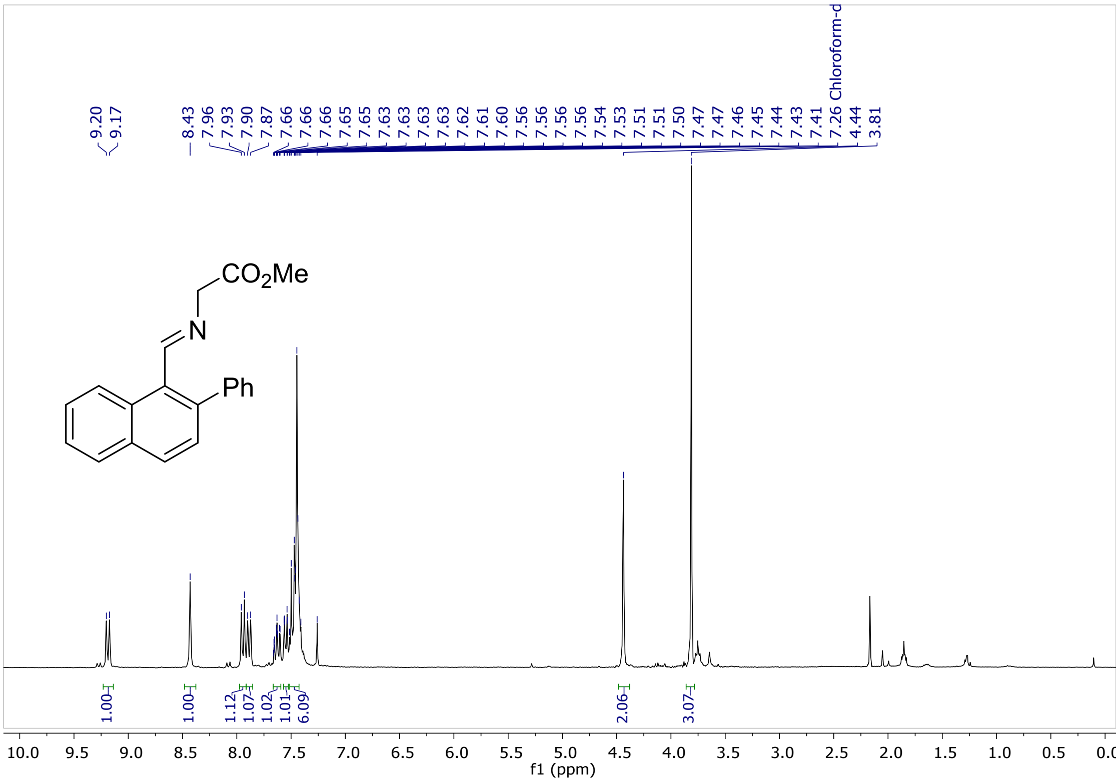

Methyl (*E*)-2-(((2-bromonaphthalen-1-yl)methylene)amino)acetate (1f)

^{1}H NMR ($CDCl_3$, 300 MHz)

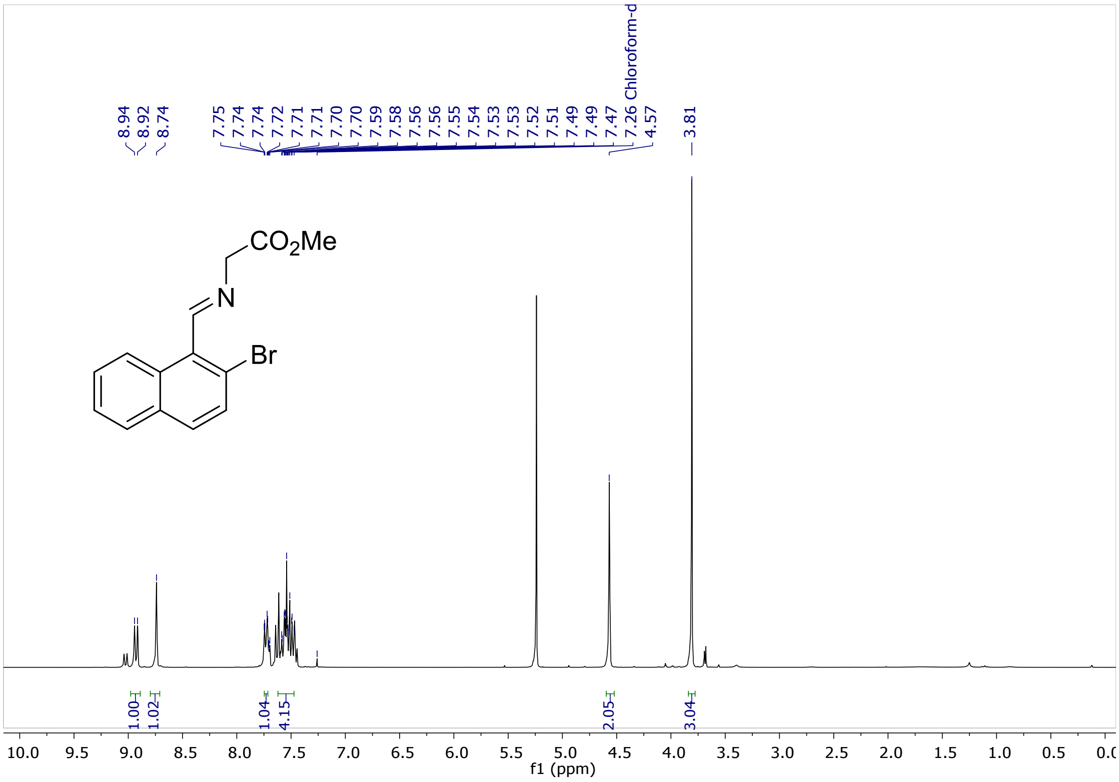

Methyl (*E*)-2-((naphthalen-1-ylmethylene)amino)acetate (1g)

^{1}H NMR ($CDCl_3$, 300 MHz)

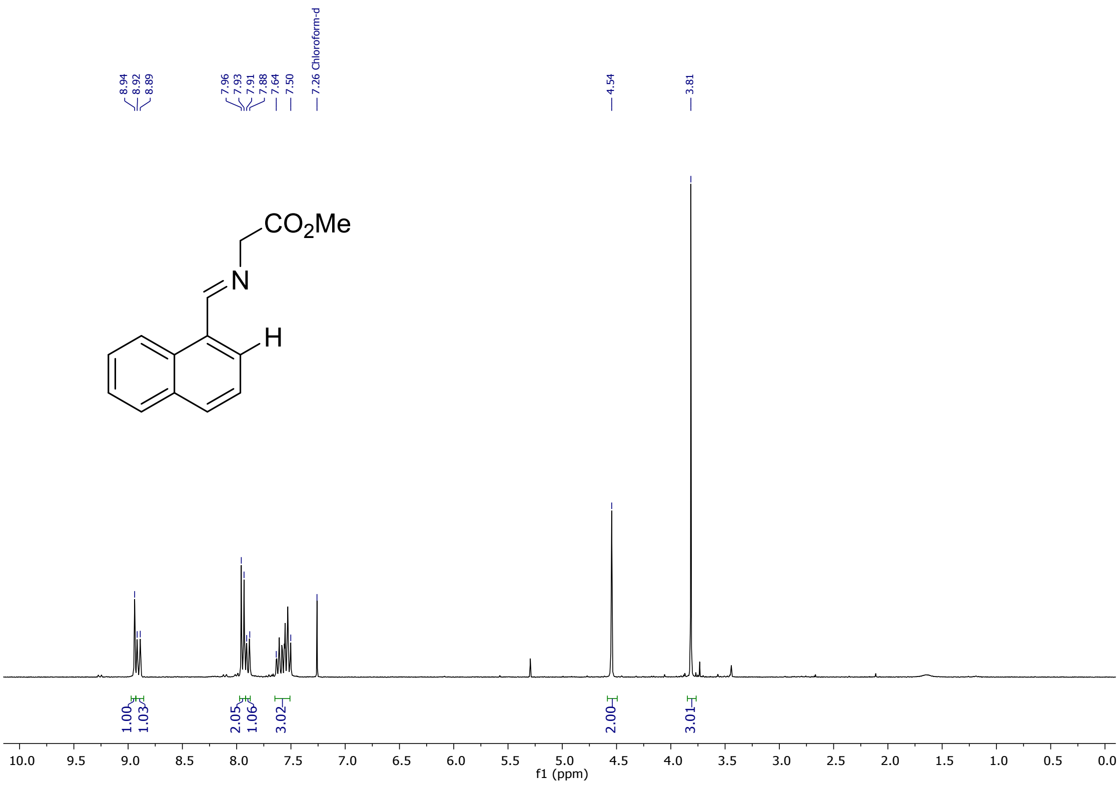

Methyl (*E*)-2-(((1-bromonaphthalen-2-yl)methylene)amino)acetate (10a)

^{1}H NMR ($CDCl_3$, 300 MHz)

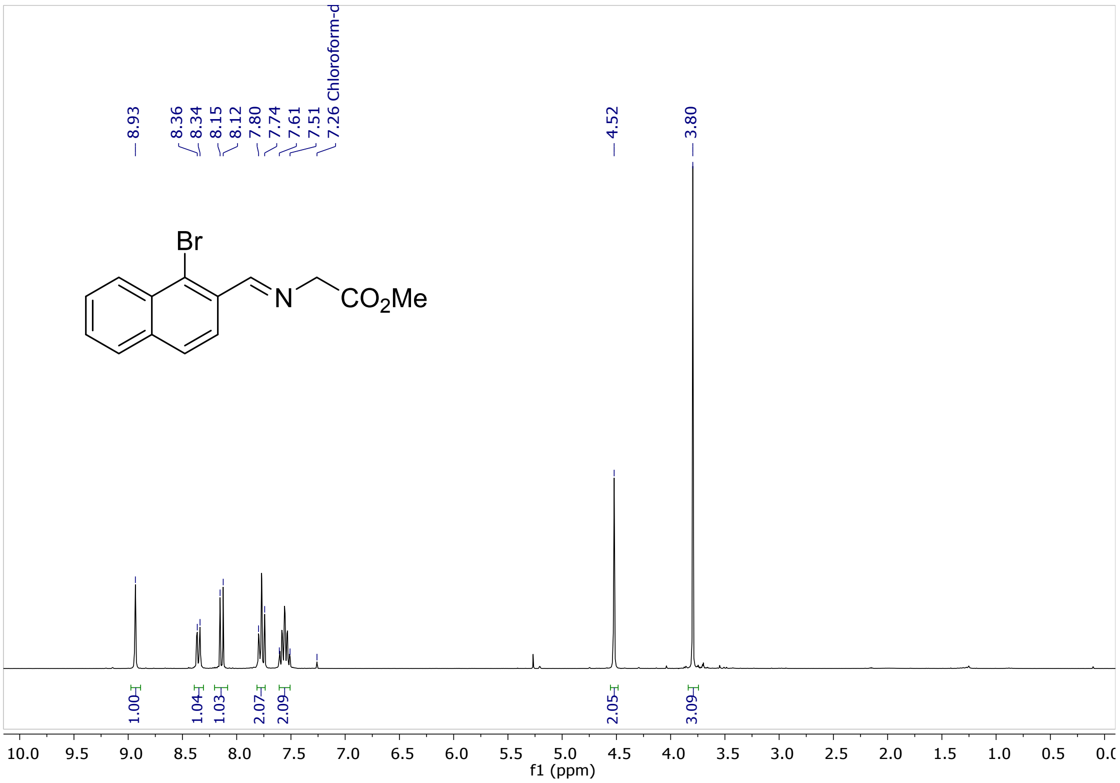

Methyl (*E*)-2-(((1-iodonaphthalen-2-yl)methylene)amino)acetate (10b)

^{1}H NMR ($CDCl_3$, 300 MHz)

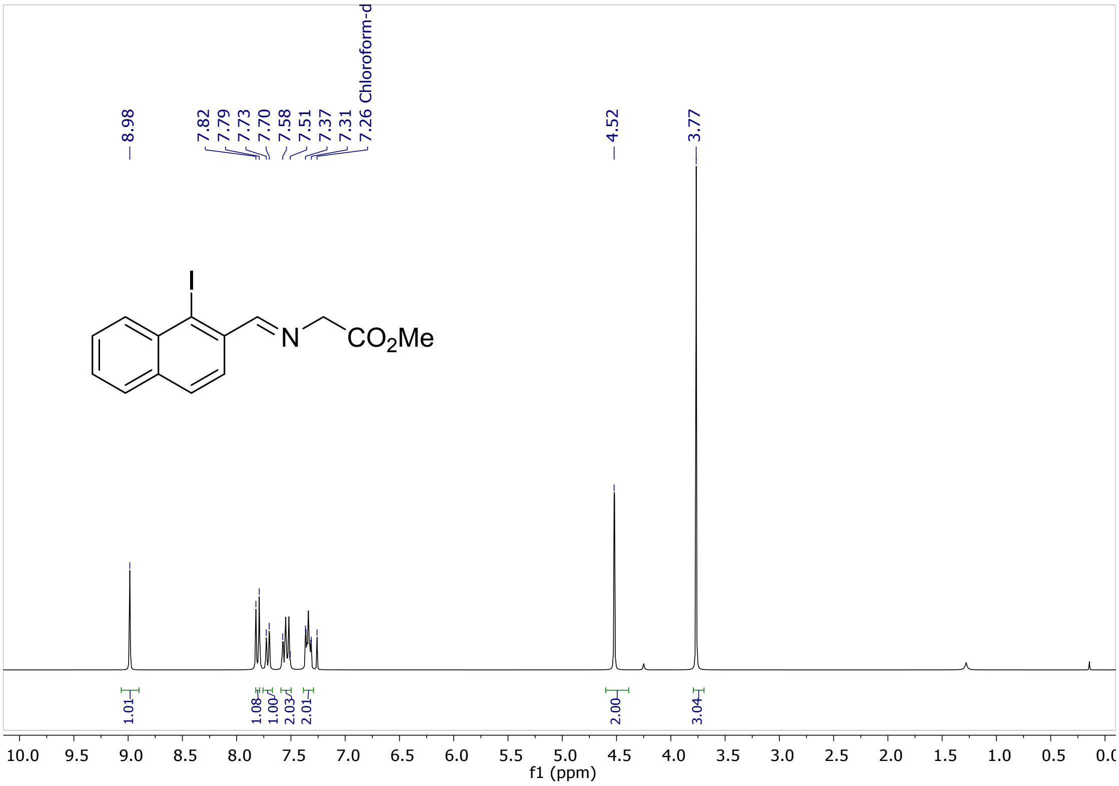

Methyl (*E*)-2-(((1-ethylnaphthalen-2-yl)methylene)amino)acetate (10c)

^{1}H NMR ($CDCl_3$, 300 MHz)

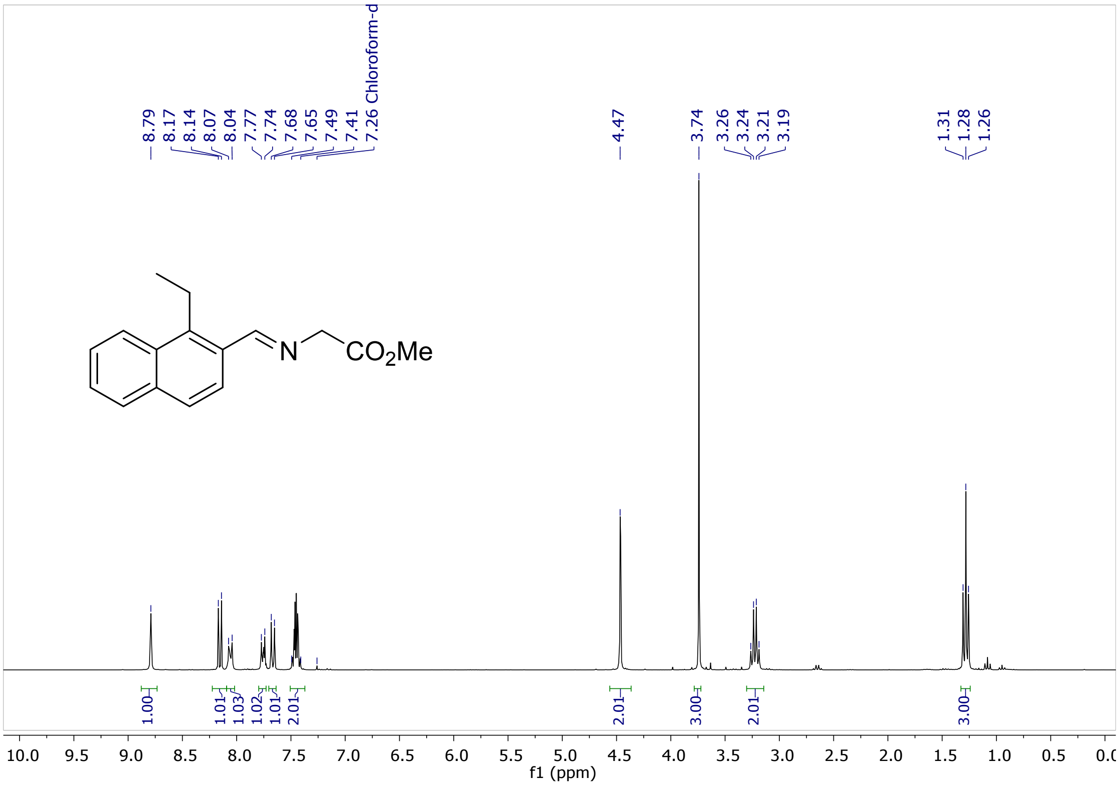

Methyl (*E*)-2-(((1-(4-methoxyphenyl)naphthalen-2-yl)methylene)amino)acetate (10d)

^{1}H NMR ($CDCl_3$, 300 MHz)

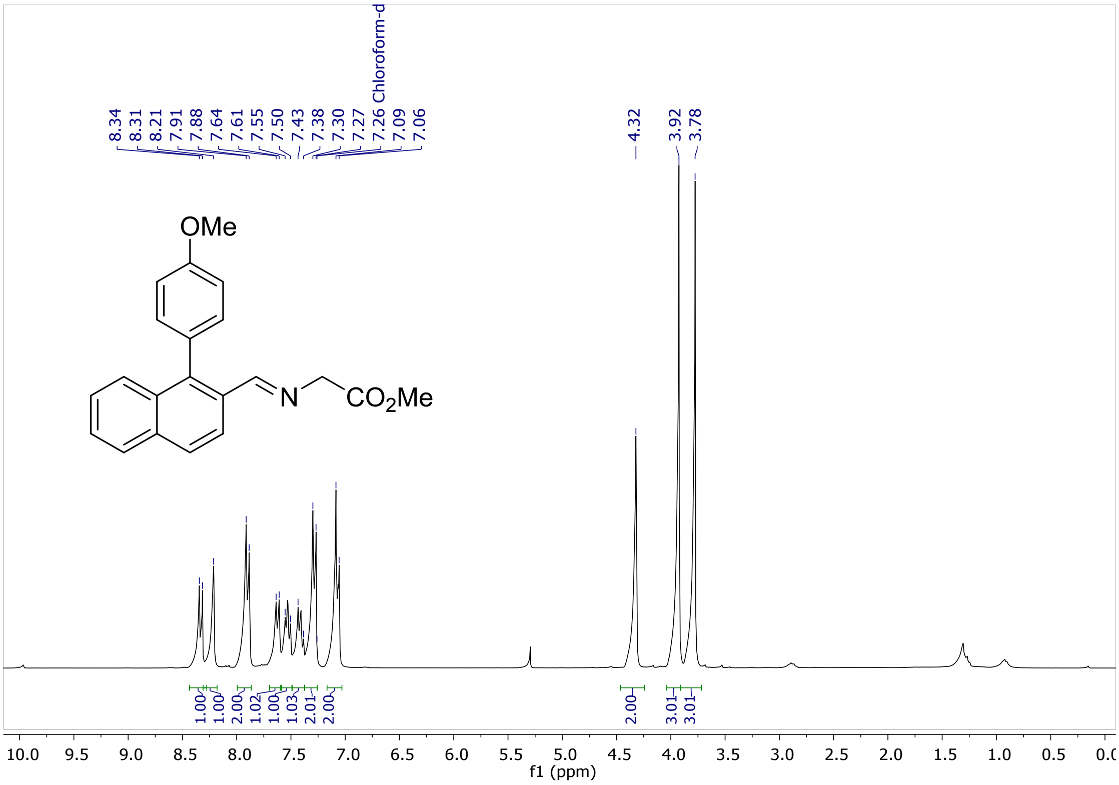

Methyl (*E*)-2-(((1-methoxynaphthalen-2-yl)methylene)amino)acetate (10e)

^{1}H NMR ($CDCl_3$, 300 MHz)

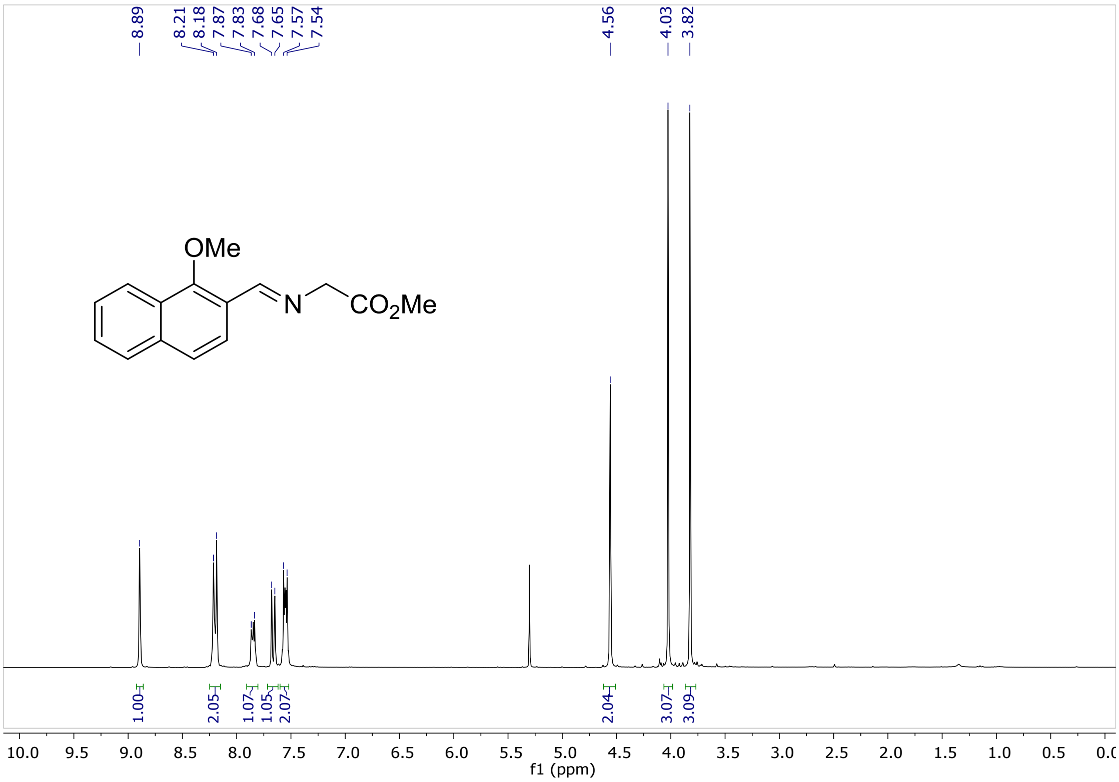

Methyl (*E*)-2-(((1-isopropoxynaphthalen-2-yl)methylene)amino)acetate (10f)

^{1}H NMR ($CDCl_3$, 300 MHz)

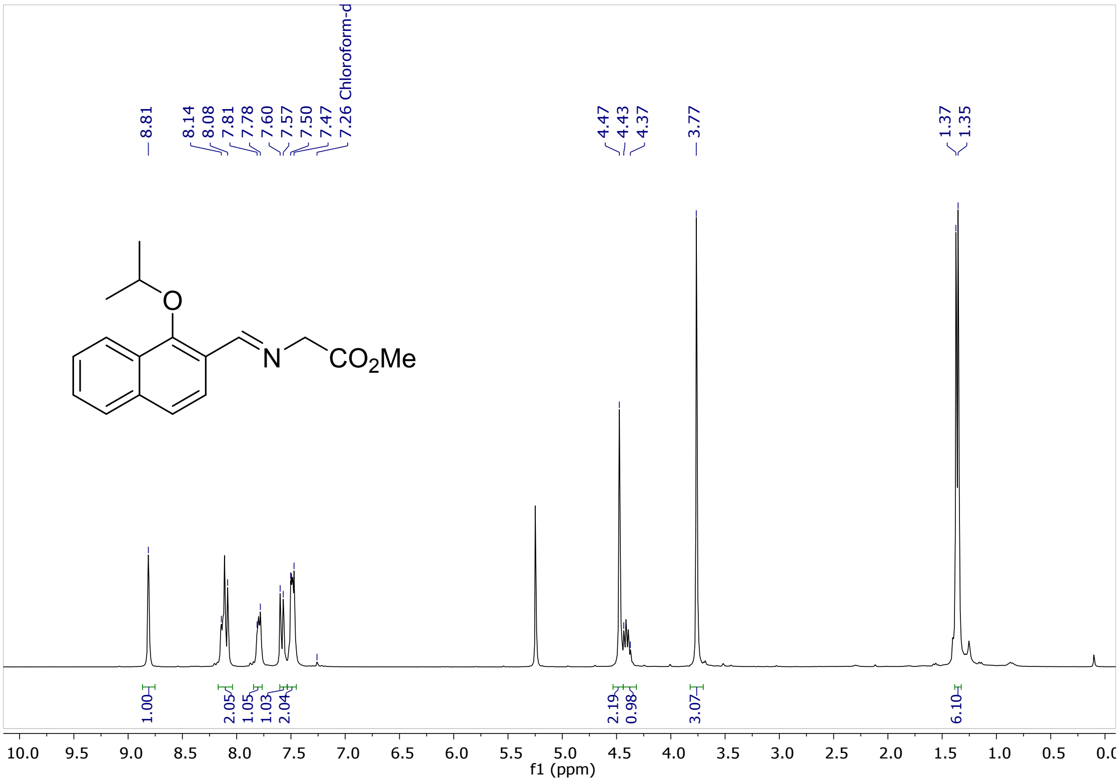

Methyl(1*S*,3*R*,3a*R*,6a*S*)-5-methyl-3-(2-methylnaphthalen-1-yl)-4,6-dioxooctahydropyrrolo[3,4-c]pyrrole-1-carboxylate (3a)

^{1}H NMR ($CDCl_3$, 300 MHz)

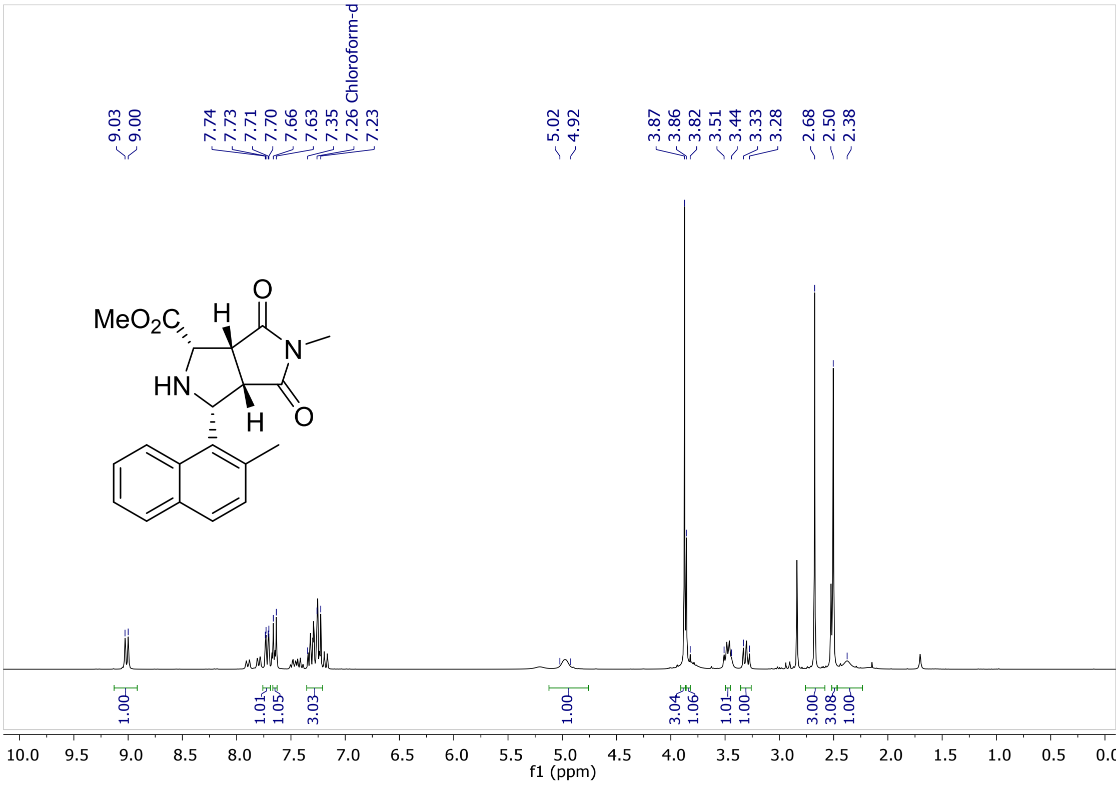

^{13}C NMR ($CDCl_3$, 75 MHz)

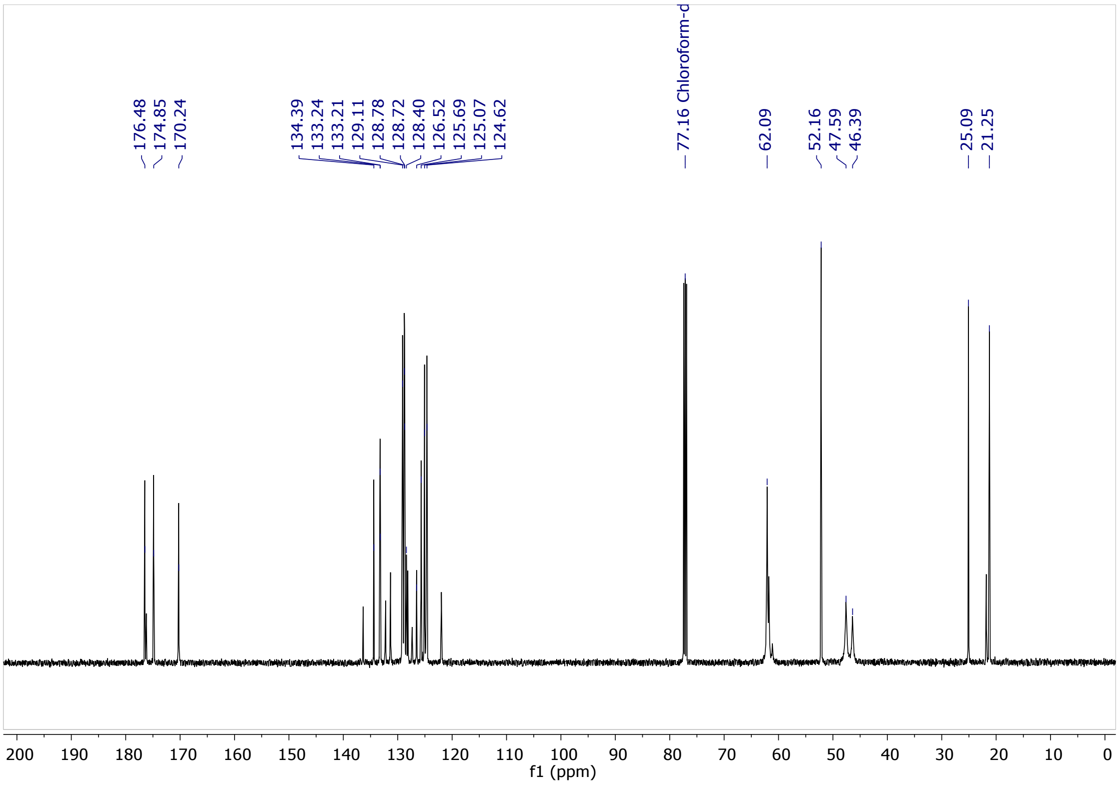

Methyl (1*S*,3*R*,3a*R*,6a*S*)-3-(2-methoxynaphthalen-1-yl)-5-methyl-4,6-dioxooctahydropyrrolo[3,4-c]pyrrole-1-carboxylate (3b)

^{1}H NMR ($CDCl_3$, 300 MHz)

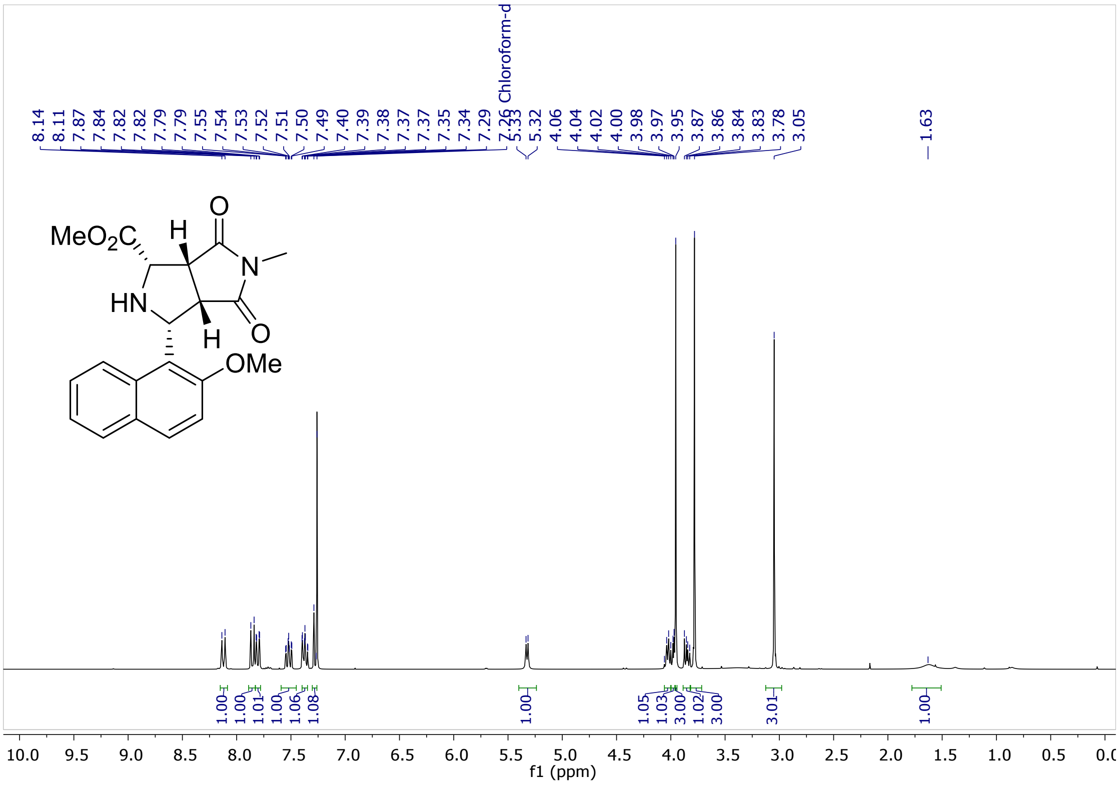

^{13}C NMR ($CDCl_3$, 75 MHz)

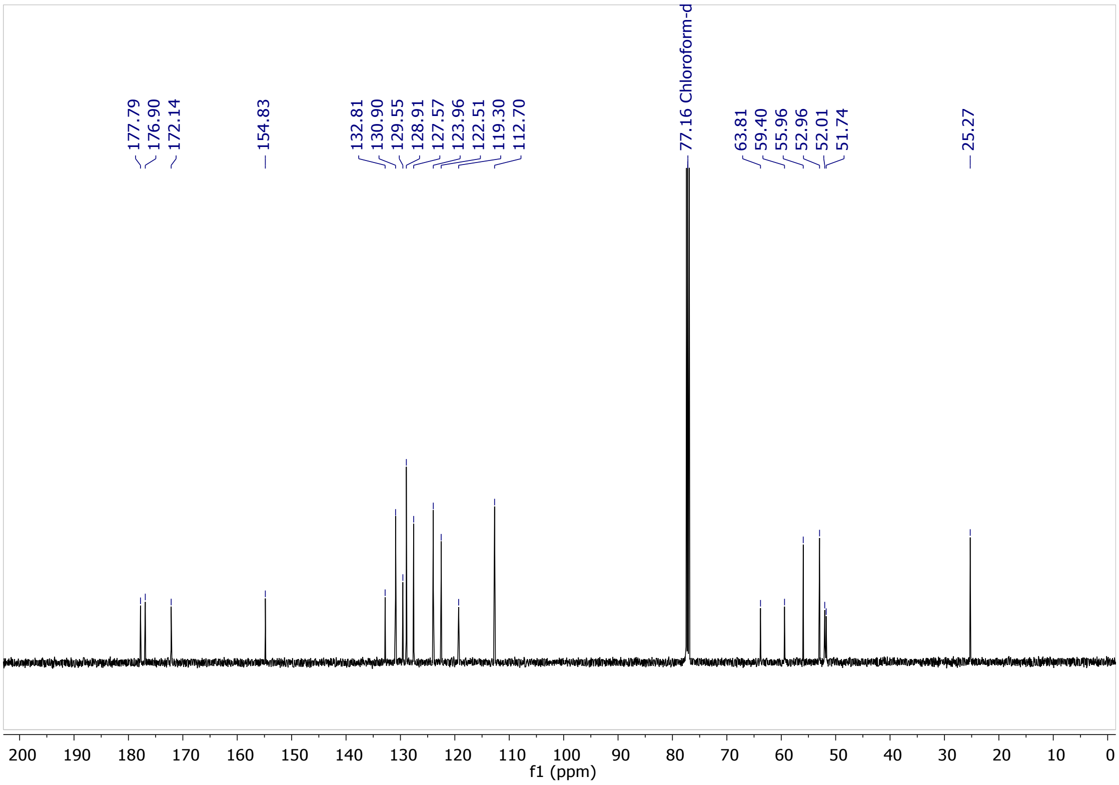

Methyl (1*S*,3*R*,3a*R*,6a*S*)-3-(2-isopropoxynaphthalen-1-yl)-5-methyl-4,6-dioxooctahydropyrrolo[3,4-c]pyrrole-1-carboxylate (3c)

^{1}H NMR ($CDCl_3$, 300 MHz)

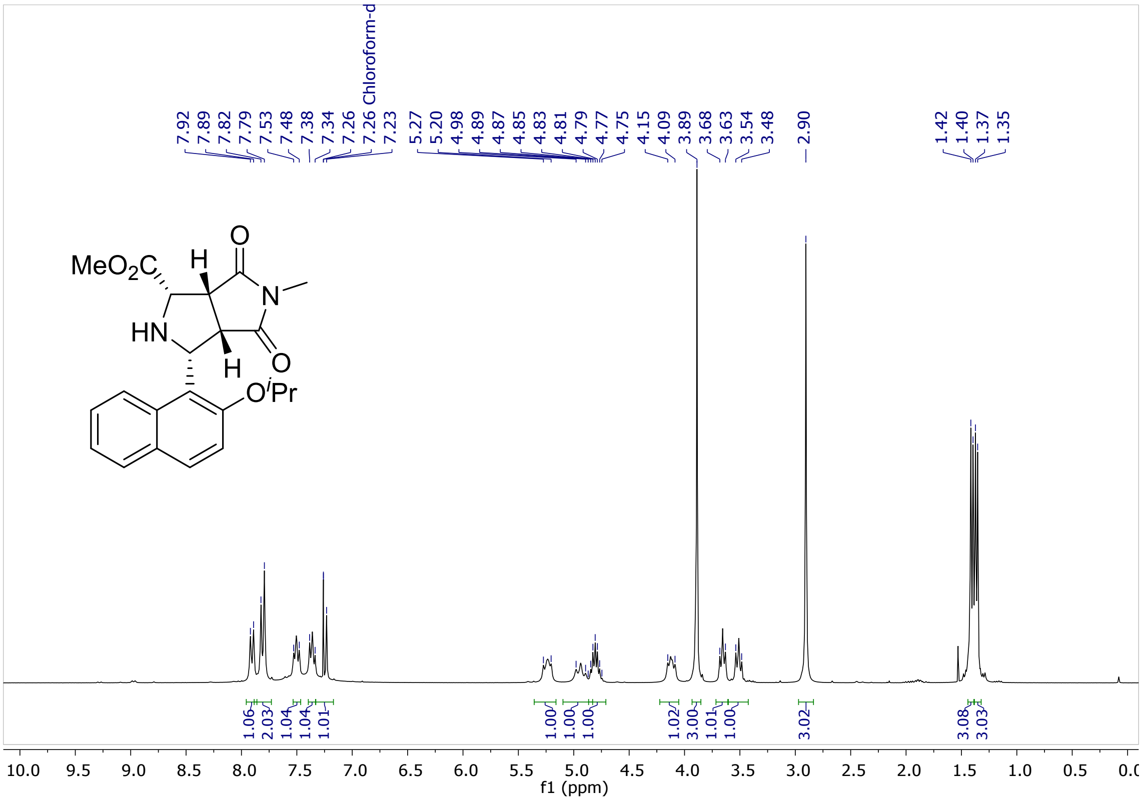

^{13}C NMR ($CDCl_3$, 75 MHz)

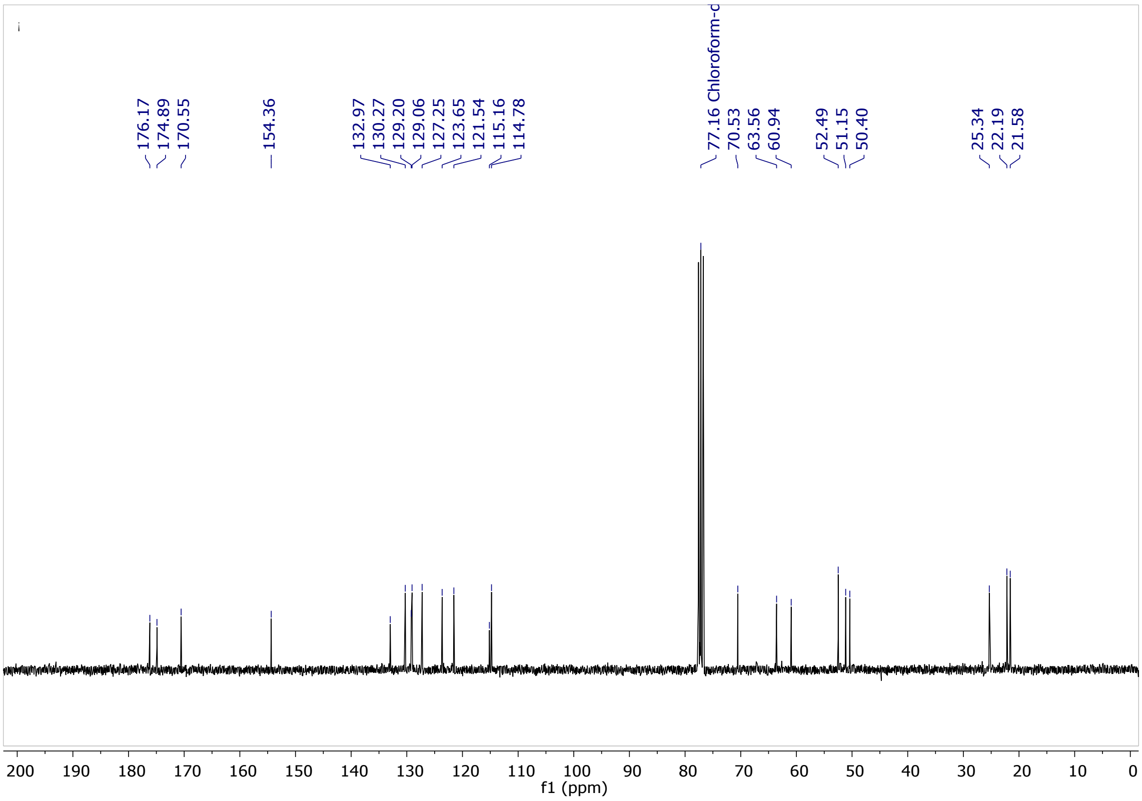

Methyl(1*S*,3*R*,3a*R*,6a*S*)-5-methyl-4,6-dioxo-3-(2-(tosyloxy)naphthalen-1-yl)octahydropyrrolo[3,4-c]pyrrole-1-carboxylate (3d)

^{1}H NMR ($CDCl_3$, 300 MHz)

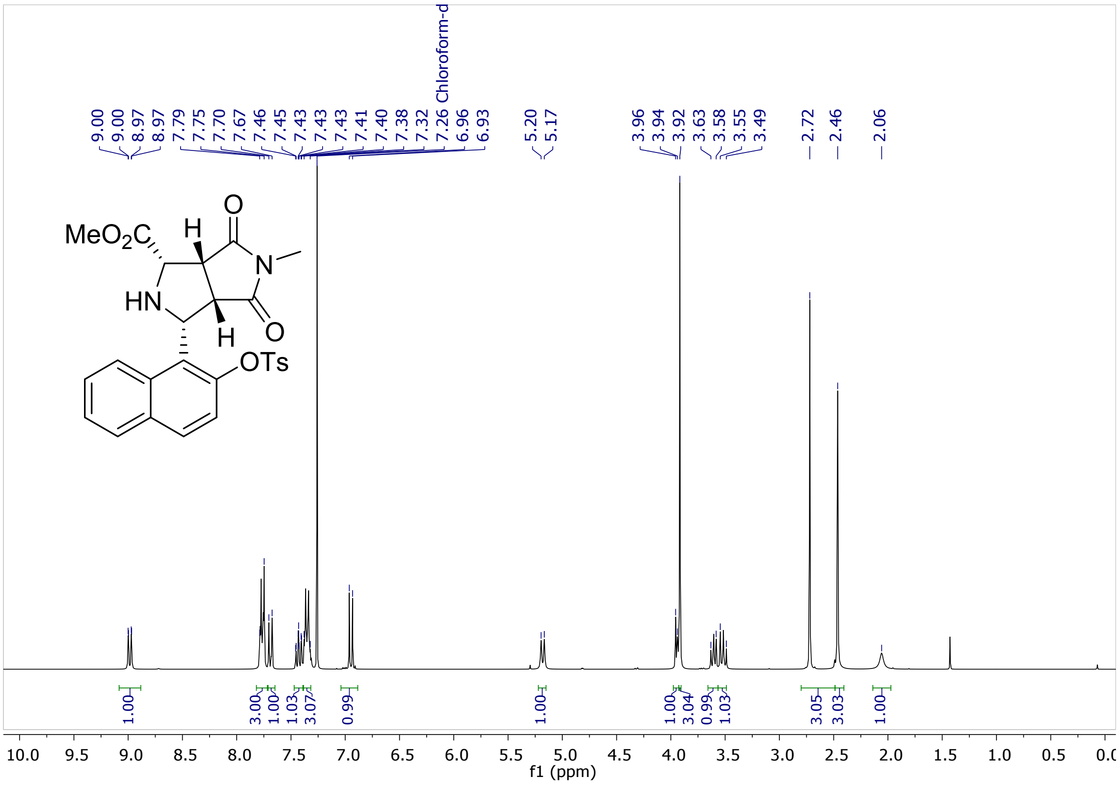

^{13}C NMR ($CDCl_3$, 126 MHz)

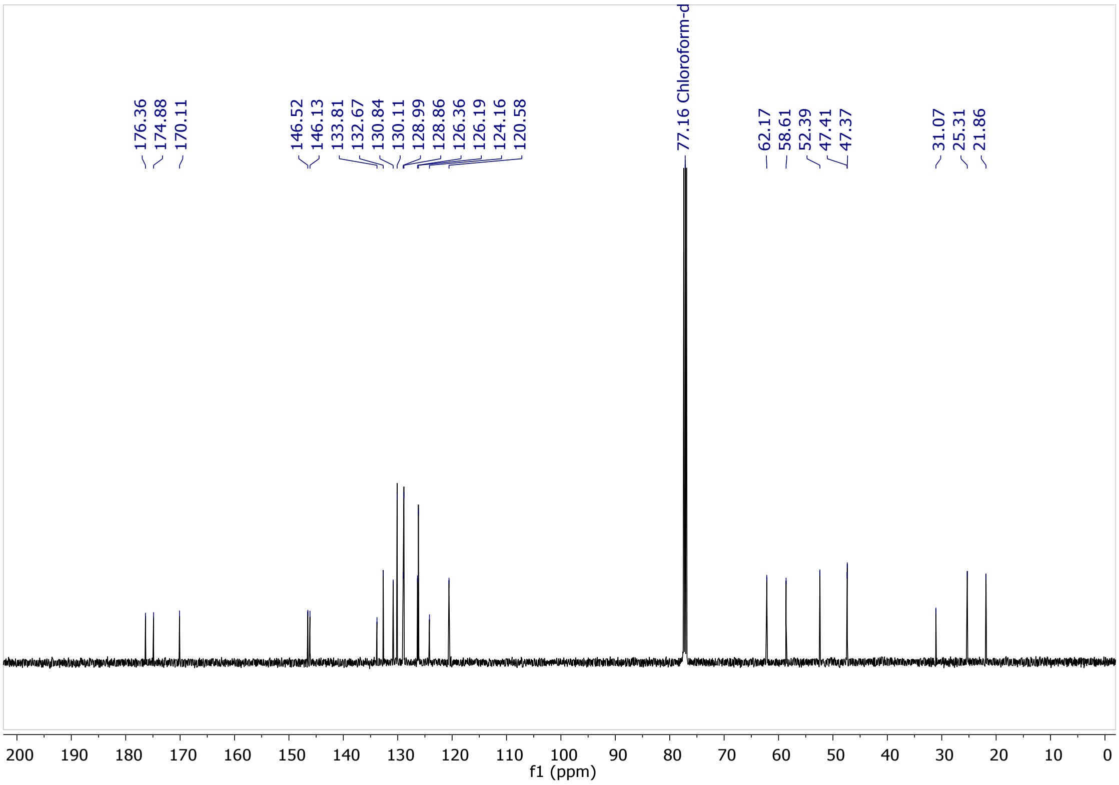

Methyl (1*S*,3*R*,3a*S*,6a*R*)-5-methyl-4,6-dioxo-3-(2-phenylnaphthalen-1-yl)octahydropyrrolo[3,4-c]pyrrole-1-carboxylate (3e)

^{1}H NMR ($CDCl_3$, 300 MHz)

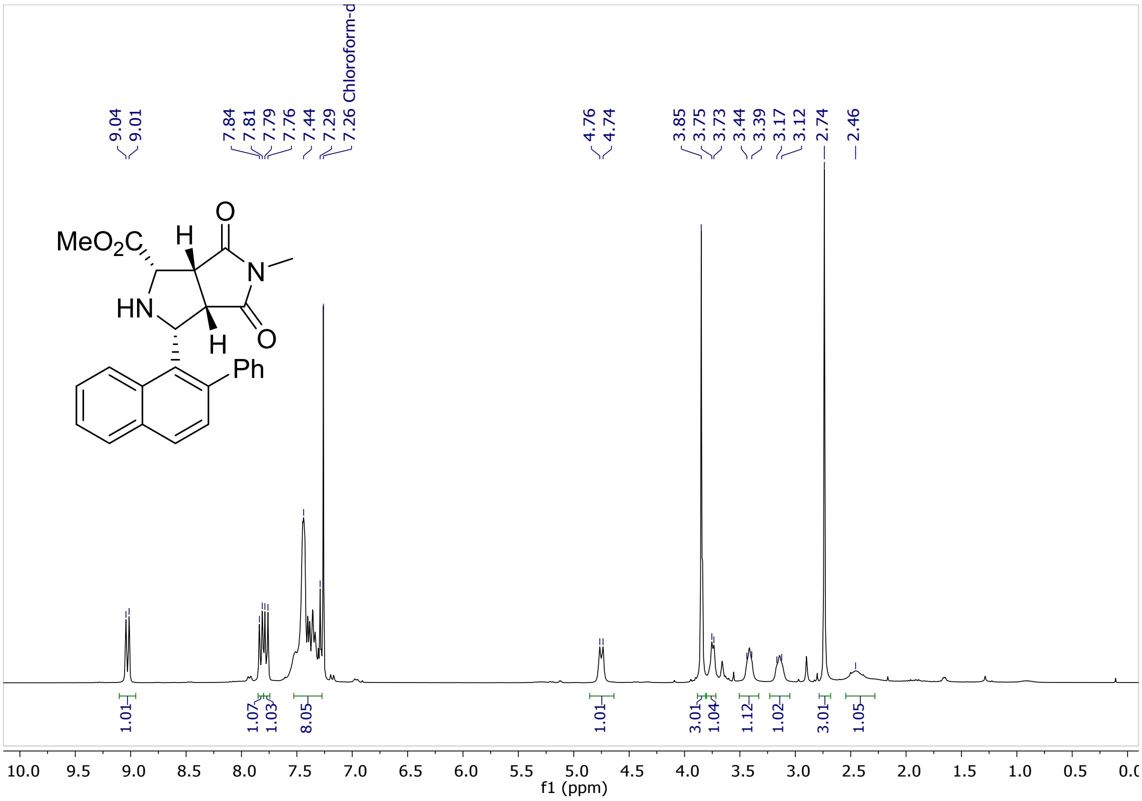

^{13}C NMR ($CDCl_3$, 75 MHz)

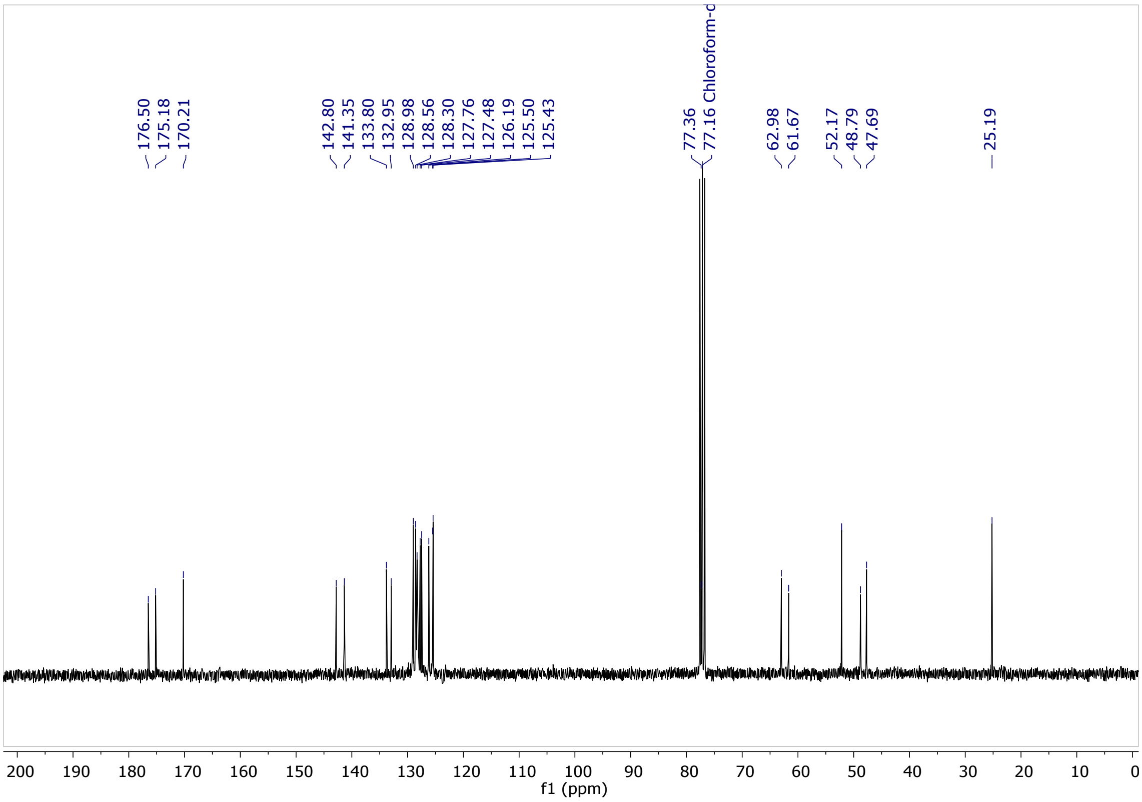

Methyl (1*S*,3*R*,3a*S*,6a*R*)-3-(2-bromonaphthalen-1-yl)-5-methyl-4,6-dioxooctahydropyrrolo[3,4-c]pyrrole-1-carboxylate (3f)

^{1}H NMR ($CDCl_3$, 300 MHz)

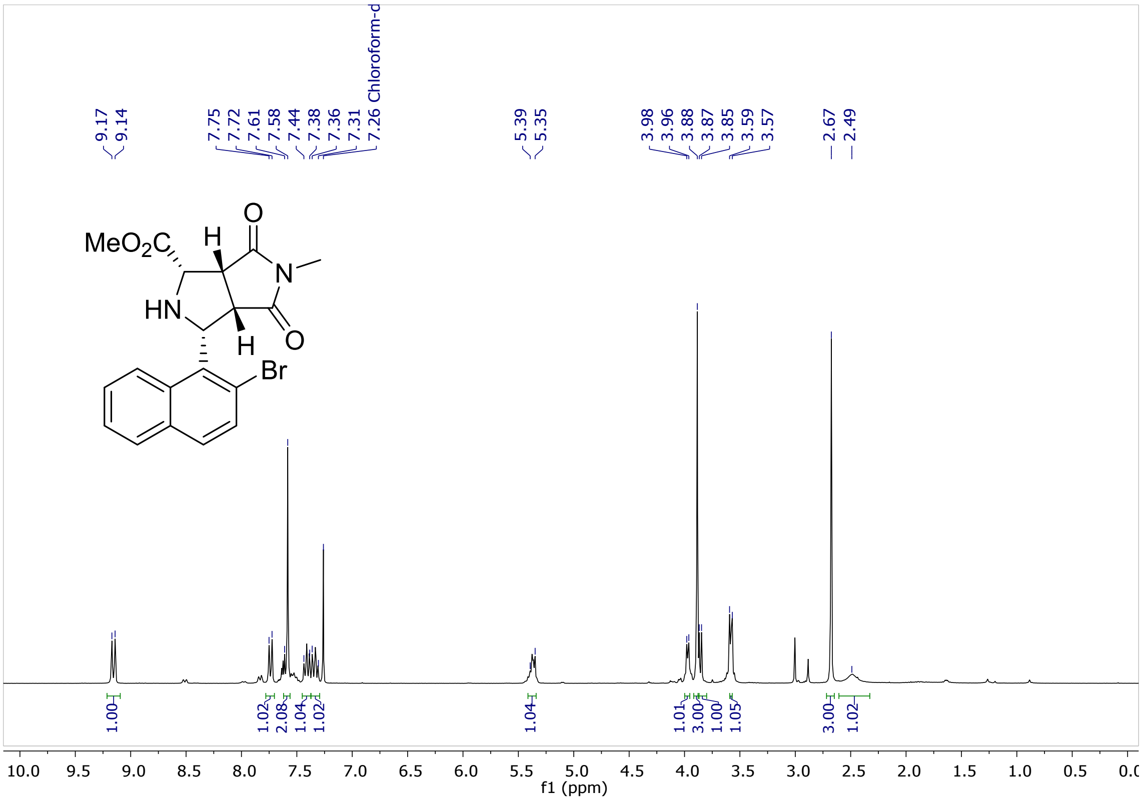

^{13}C NMR ($CDCl_3$, 75 MHz)

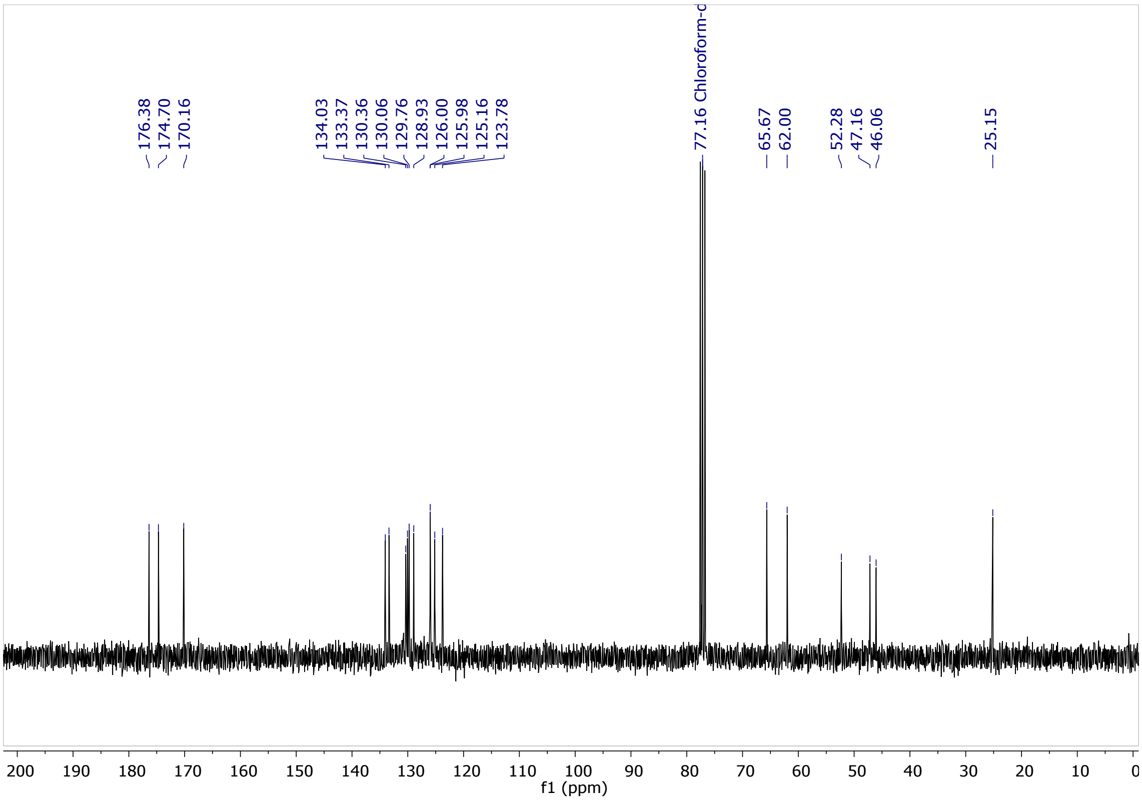

Methyl (1*S*,3*R*,3a*S*,6a*R*)-5-methyl-3-(naphthalen-1-yl)-4,6-dioxooctahydropyrrolo[3,4-c]pyrrole-1-carboxylate (3g)

^{1}H NMR ($CDCl_3$, 300 MHz)

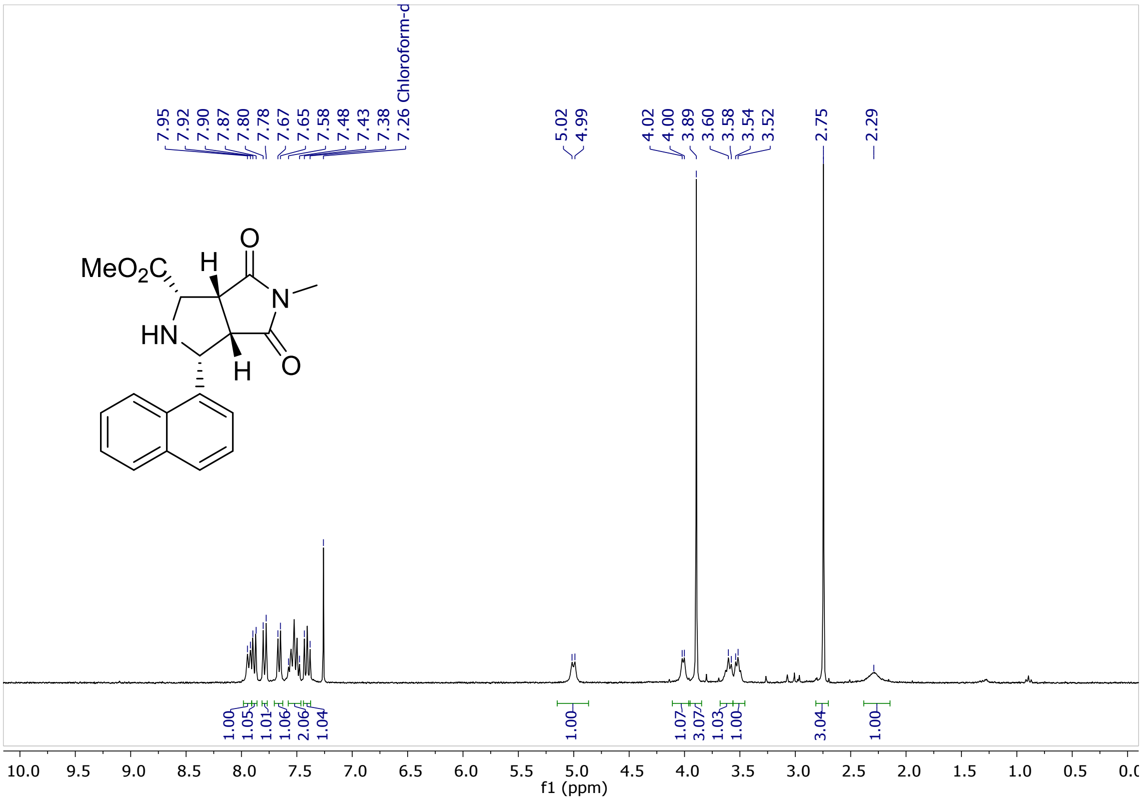

^{13}C NMR ($CDCl_3$, 75 MHz)

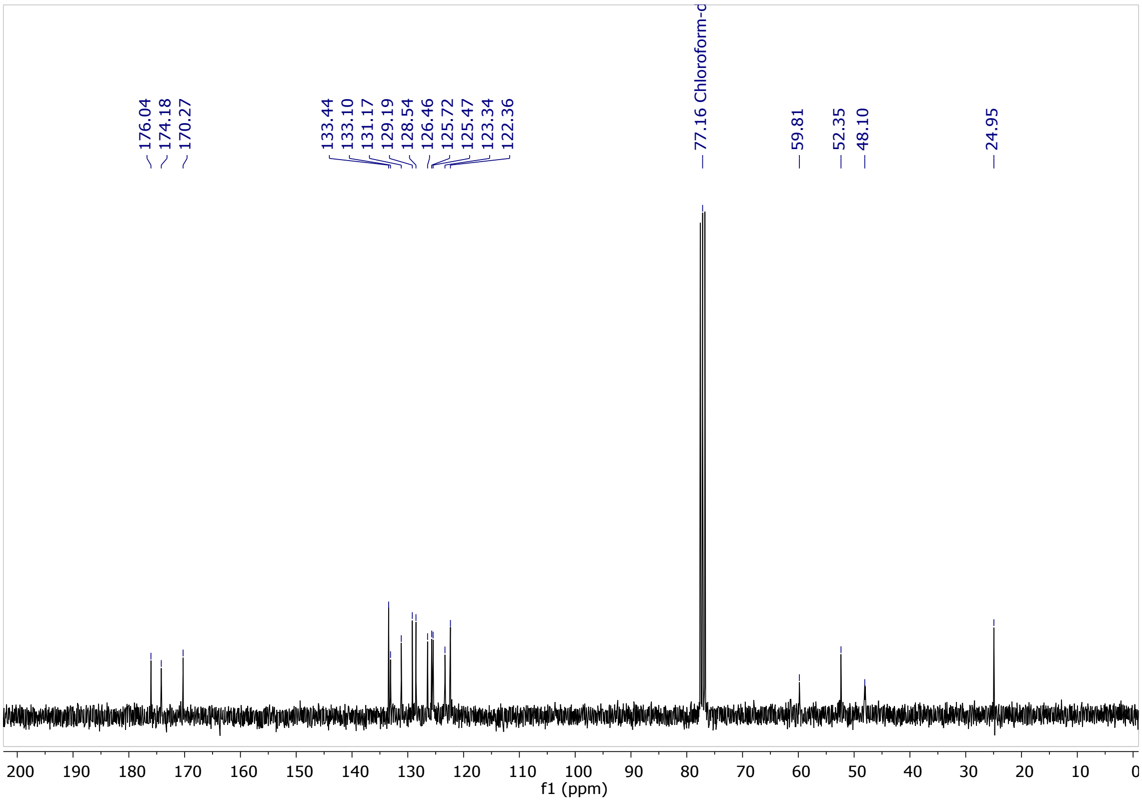

Methyl(1*S*,3*R*,3a*R*,6a*S*)-3-(1-bromonaphthalen-2-yl)-5-methyl-4,6-dioxooctahydropyrrolo[3,4-c]pyrrole-1-carboxylate (11a)

^{1}H NMR ($CDCl_3$, 300 MHz)

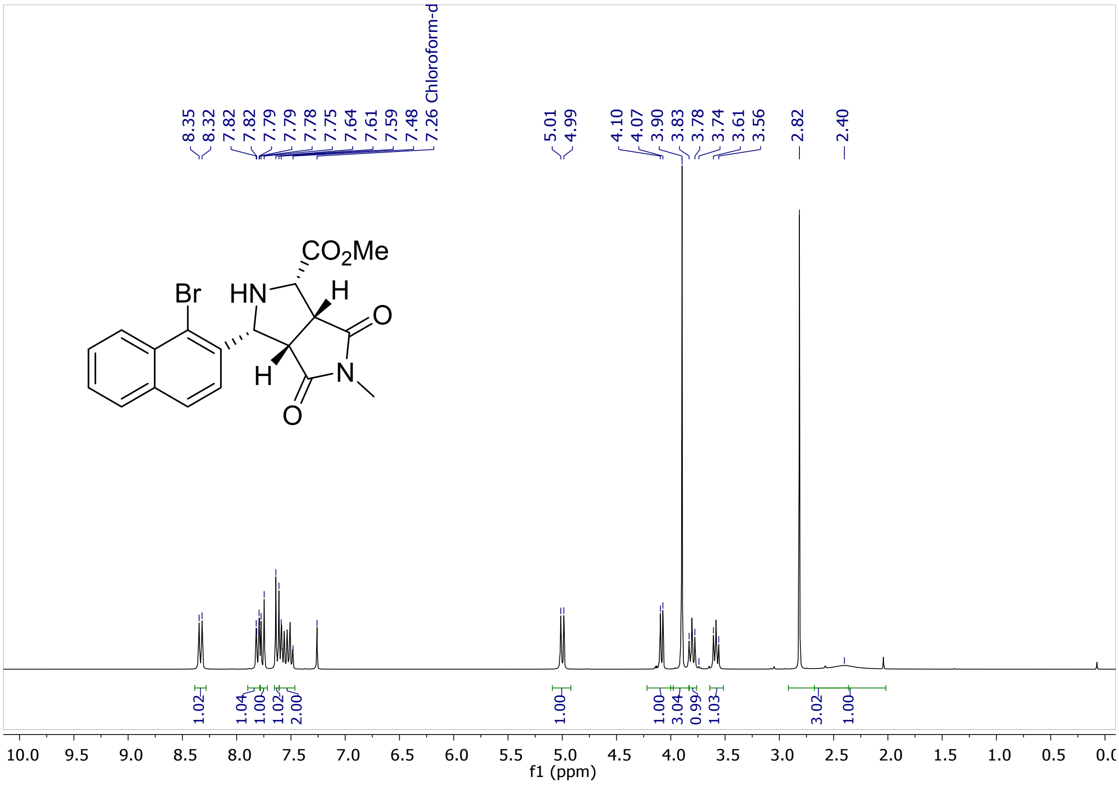

^{13}C NMR ($CDCl_3$, 75 MHz)

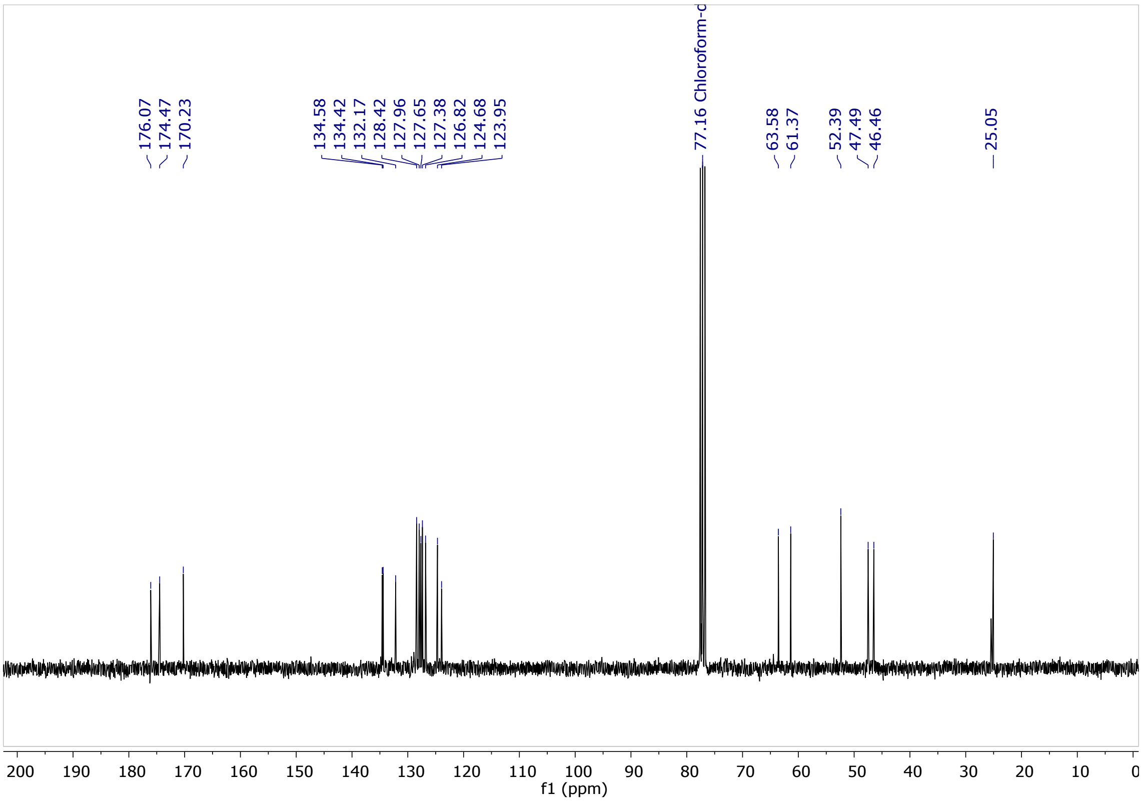

Methyl(1*S*,3*R*,3a*R*,6a*S*)-3-(1-iodonaphthalen-2-yl)-5-methyl-4,6-dioxooctahydropyrrolo[3,4-c]pyrrole-1-carboxylate (11b)

^{1}H NMR ($CDCl_3$, 300 MHz)

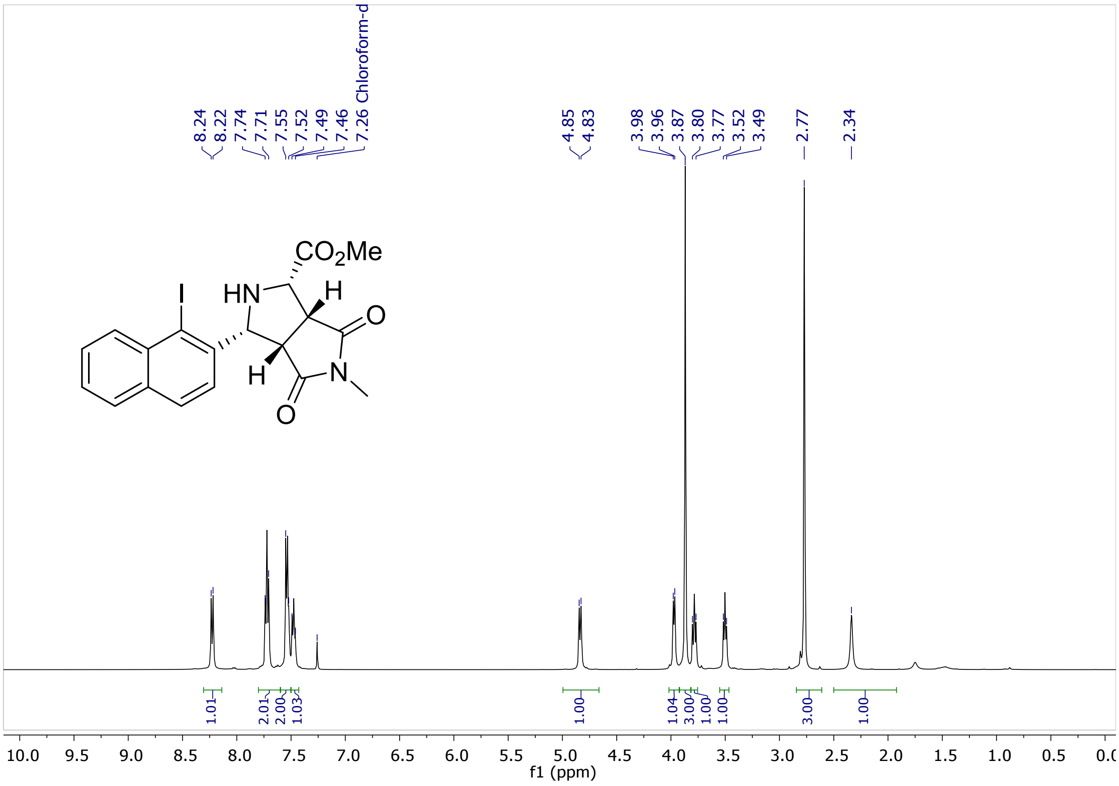

^{13}C NMR ($CDCl_3$, 75 MHz)

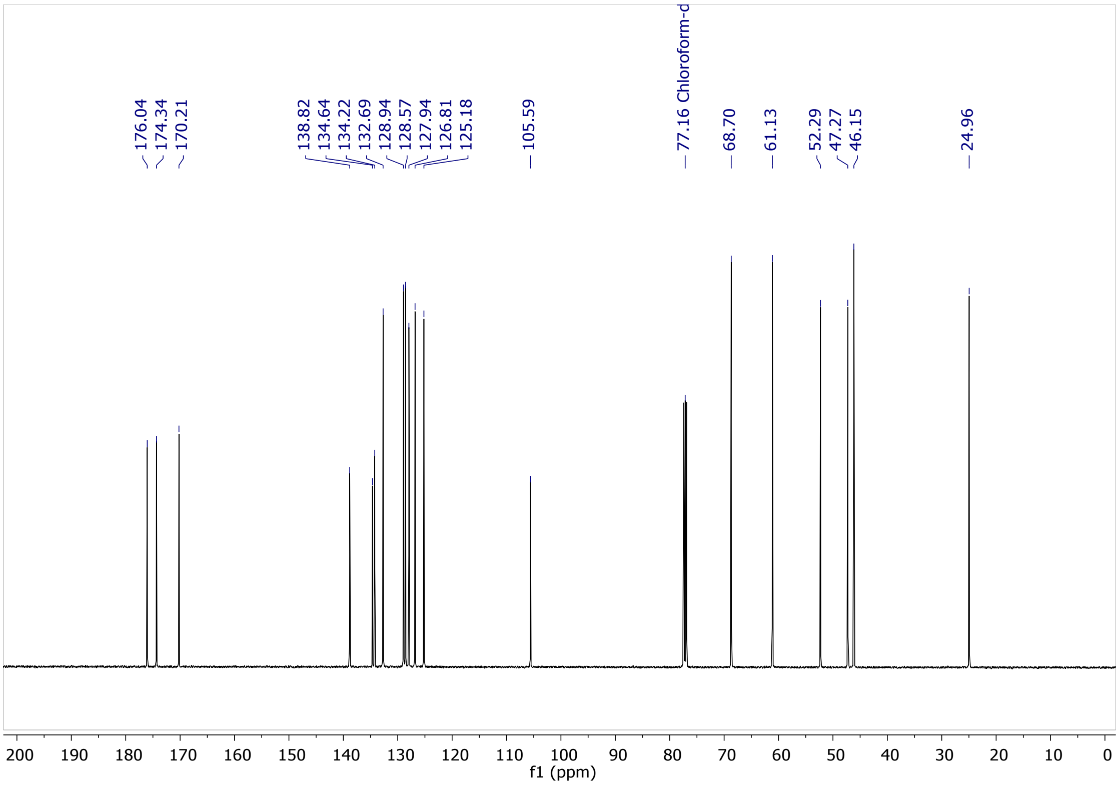

Methyl (1*S*,3*R*,3a*S*,6a*R*)-3-(1-ethylnaphthalen-2-yl)-5-methyl-4,6-dioxooctahydropyrrolo[3,4-c]pyrrole-1-carboxylate (11c)

^{1}H NMR ($CDCl_3$, 300 MHz)

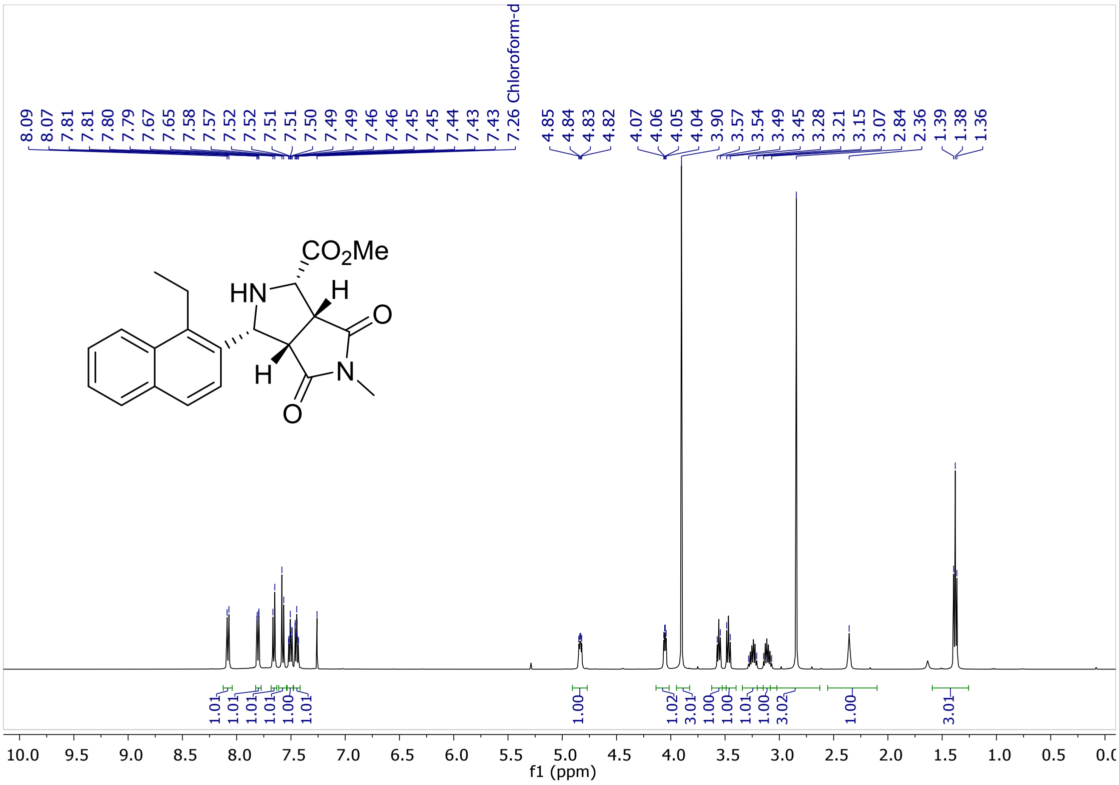

^{13}C NMR ($CDCl_3$, 75 MHz)

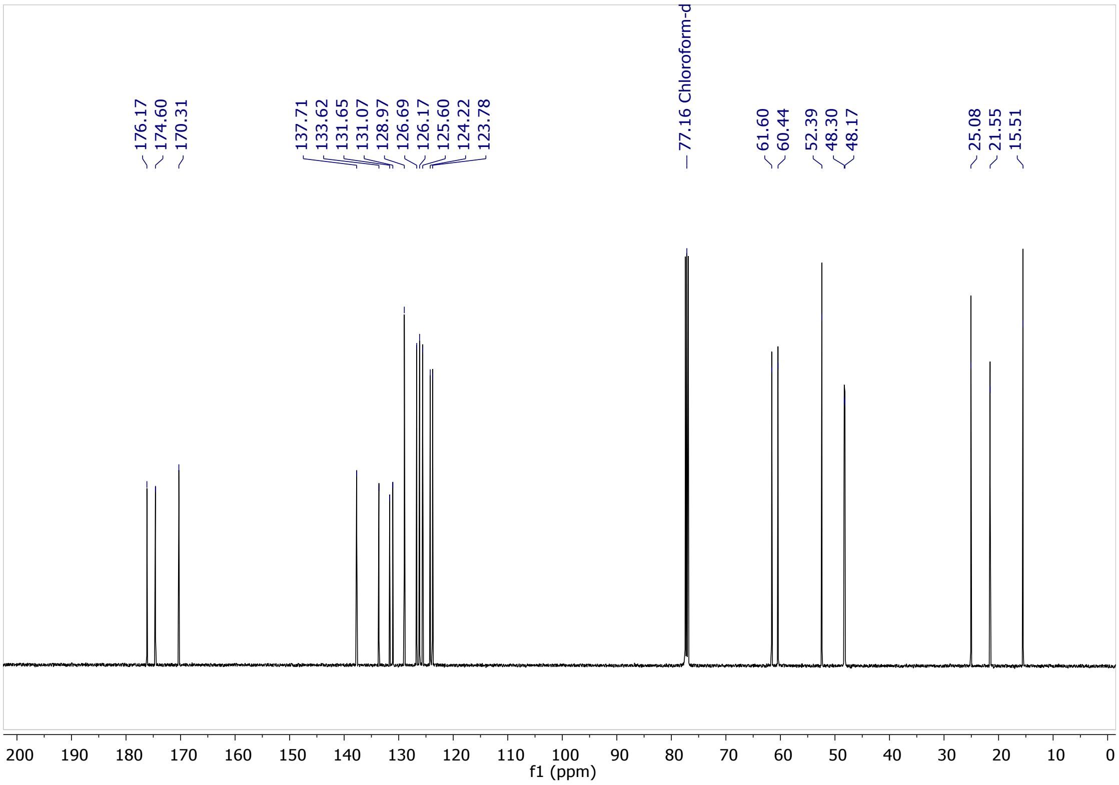

Methyl (1*S*,3*R*,3a*S*,6a*R*)-3-(1-(4-methoxyphenyl)naphthalen-2-yl)-5-methyl-4,6-dioxooctahydropyrrolo[3,4-c]pyrrole-1-carboxylate (11d)

^{1}H NMR ($CDCl_3$, 300 MHz)

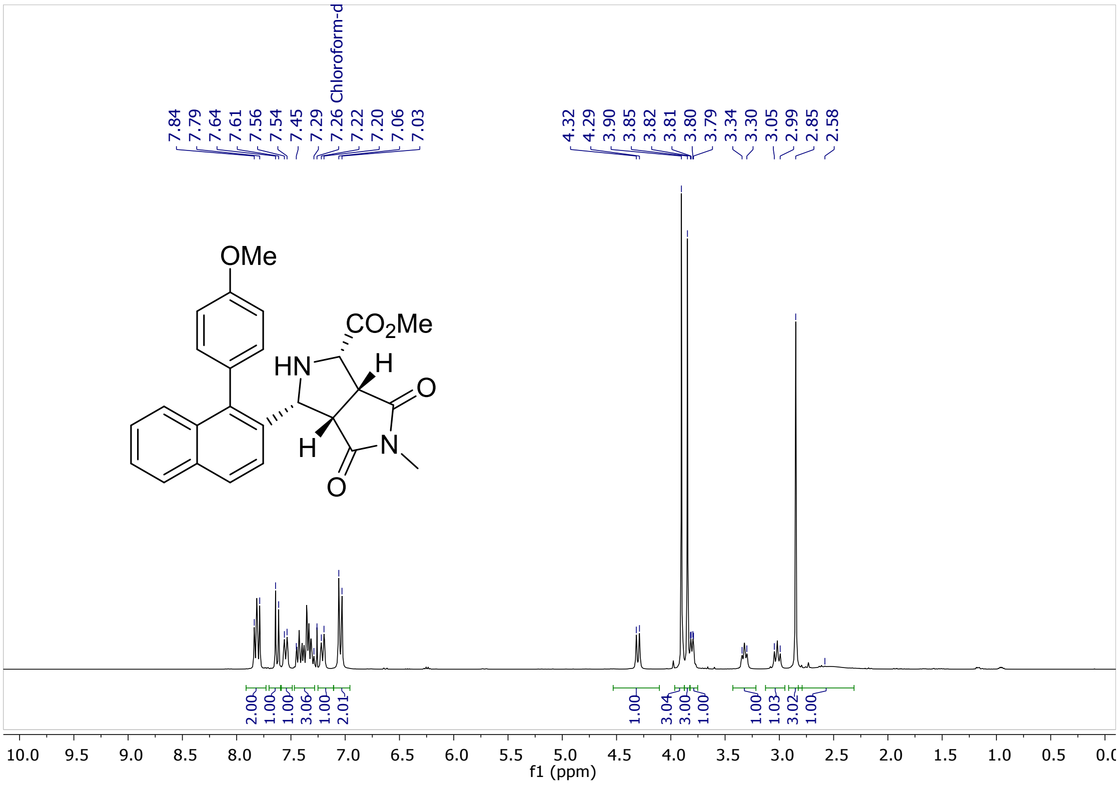

^{13}C NMR ($CDCl_3$, 126 MHz)

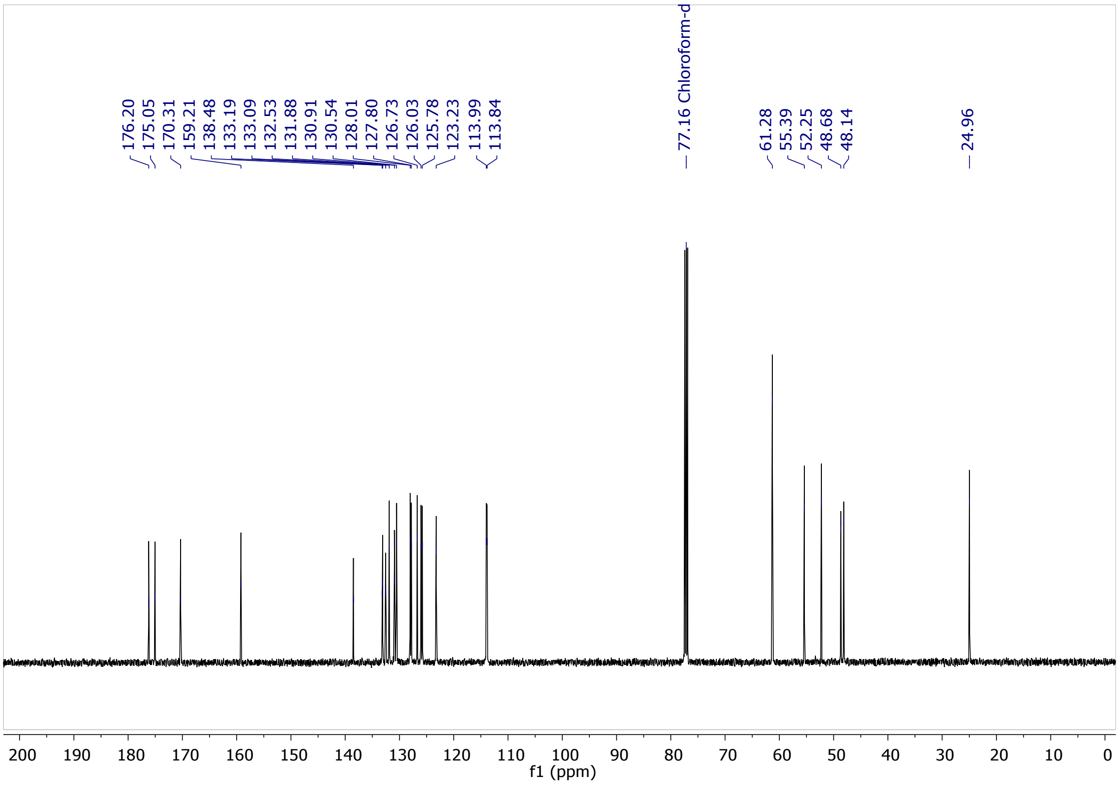

Methyl (1*S*,3*R*,3a*S*,6a*R*)-3-(1-methoxynaphthalen-2-yl)-5-methyl-4,6-dioxooctahydropyrrolo[3,4-c]pyrrole-1-carboxylate (11e)

^{1}H NMR ($CDCl_3$, 300 MHz)

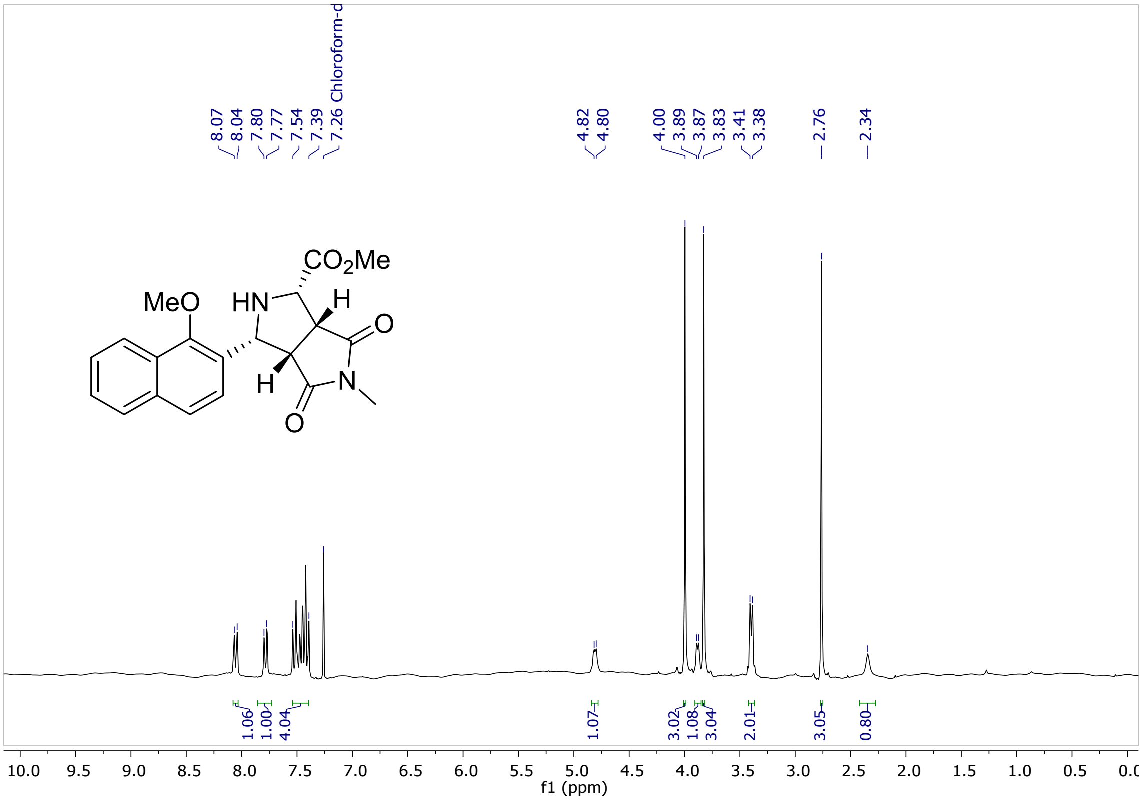

^{13}C NMR ($CDCl_3$, 75 MHz)

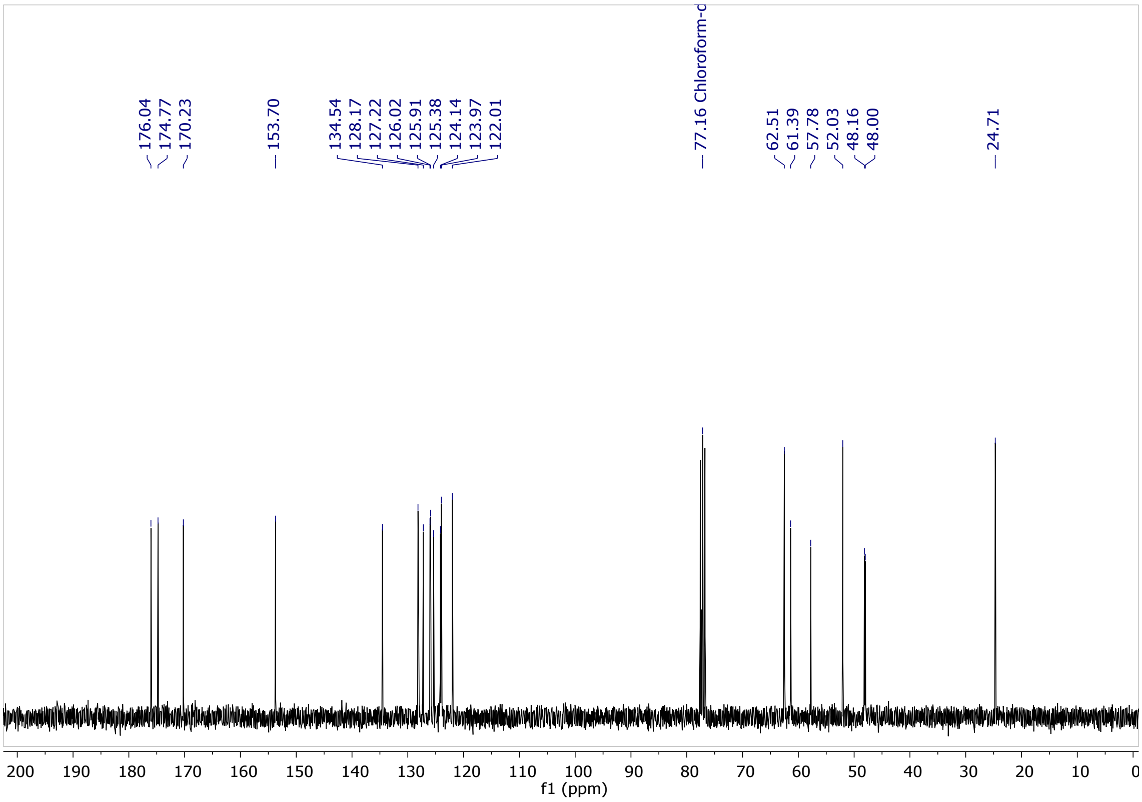

Methyl (1*S*,3*R*,3a*S*,6a*R*)-3-(1-isopropoxynaphthalen-2-yl)-5-methyl-4,6-dioxooctahydropyrrolo[3,4-c]pyrrole-1-carboxylate (11f)

^{1}H NMR ($CDCl_3$, 300 MHz)

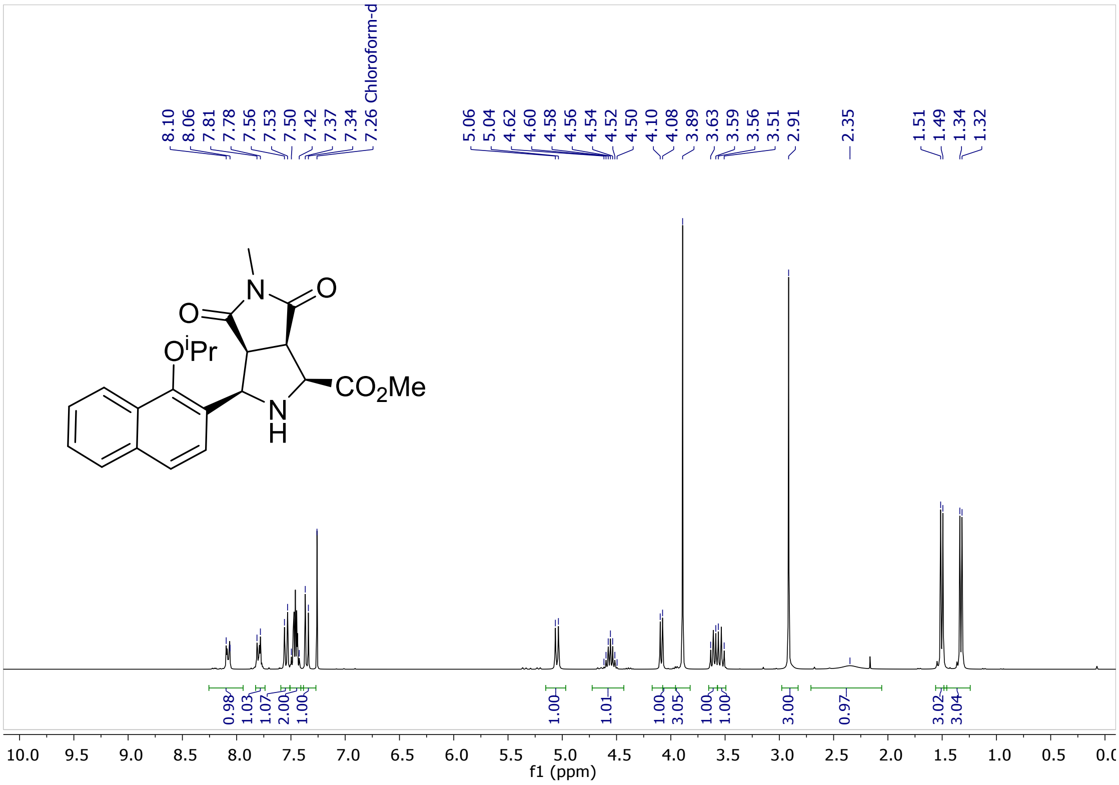

^{13}C NMR ($CDCl_3$, 126 MHz)

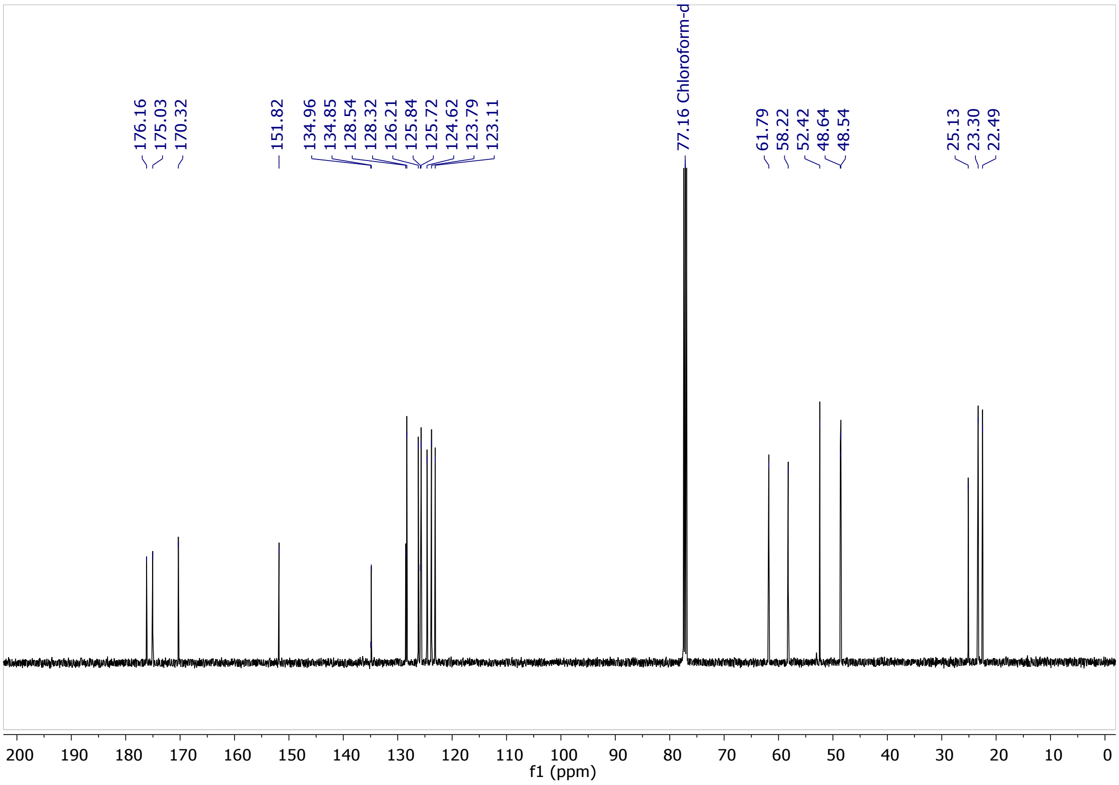

Methyl (1*S*,3*R*,3a*S*,6a*R*)-3-(2-methylnaphthalen-1-yl)-4,6-dioxo-5-phenyloctahydropyrrolo[3,4-c]pyrrole-1-carboxylate (15a)

^{1}H NMR ($CDCl_3$, 300 MHz)

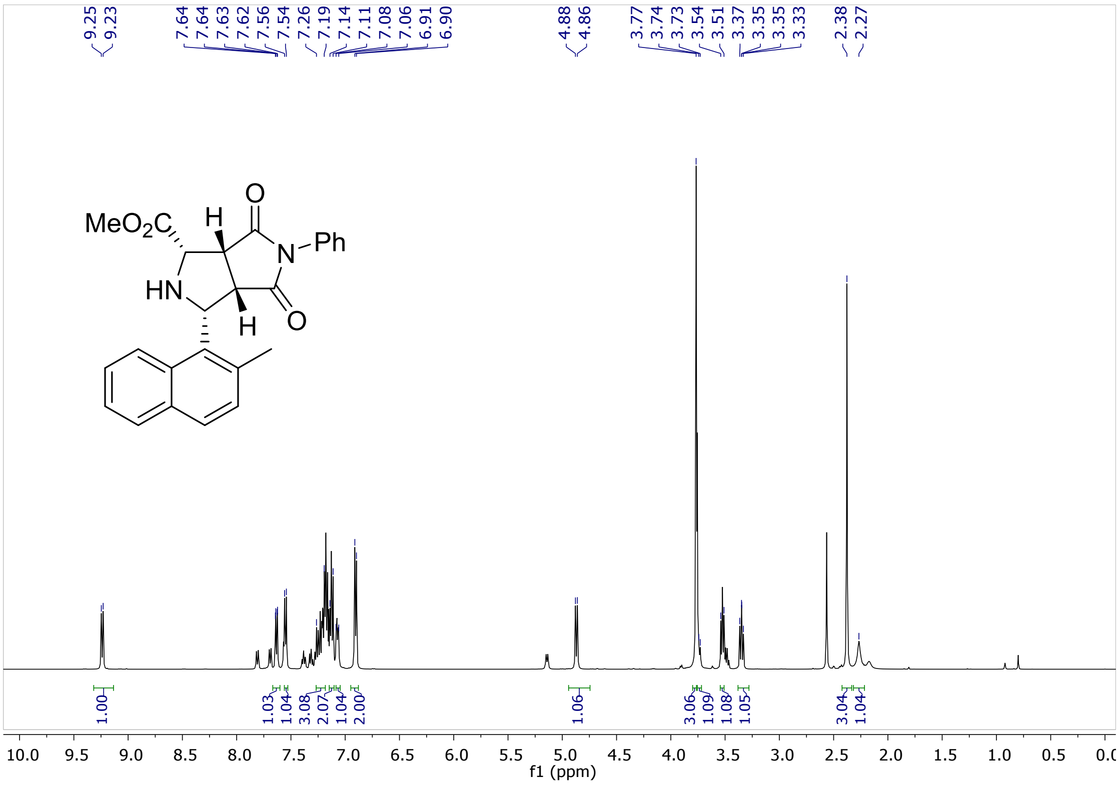

^{13}C NMR ($CDCl_3$, 75 MHz)

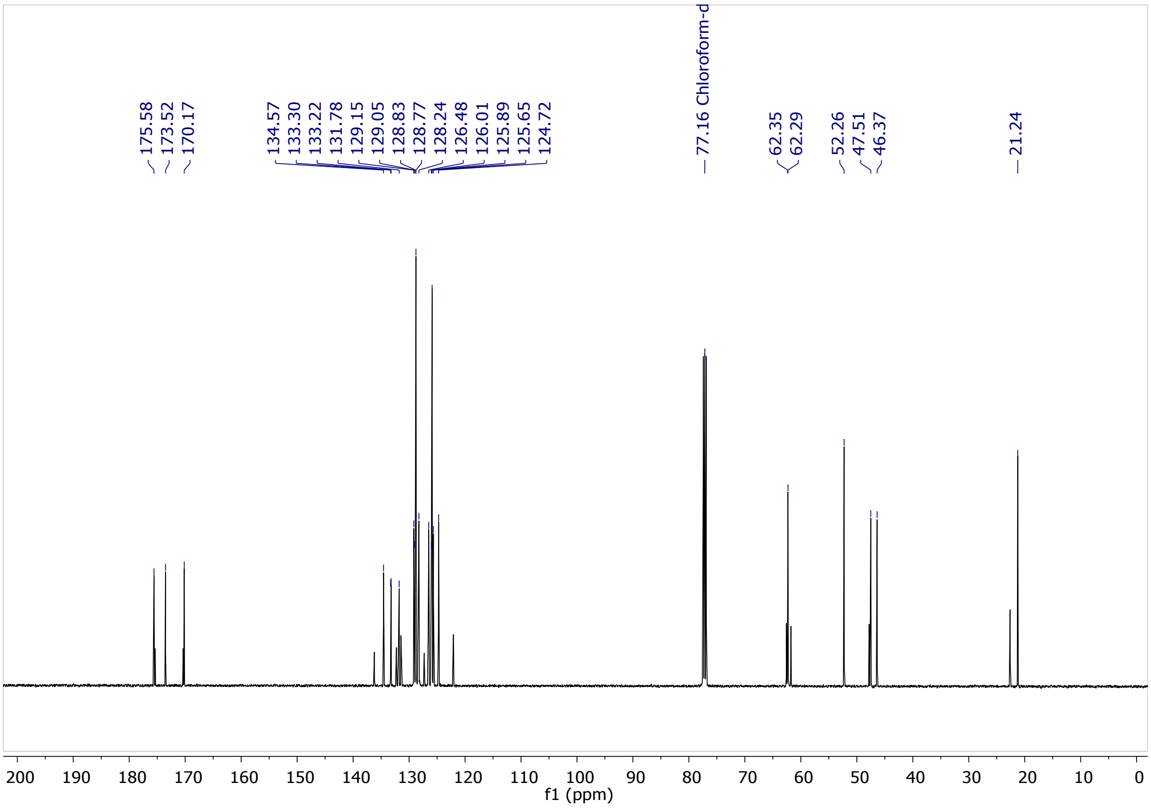

Trimethyl (2*S*,3*S*,4*S*,5*R*)-5-(2-methylnaphthalen-1-yl)pyrrolidine-2,3,4-tricarboxylate (15b)

^1H NMR ($CDCl_3$, 300 MHz)

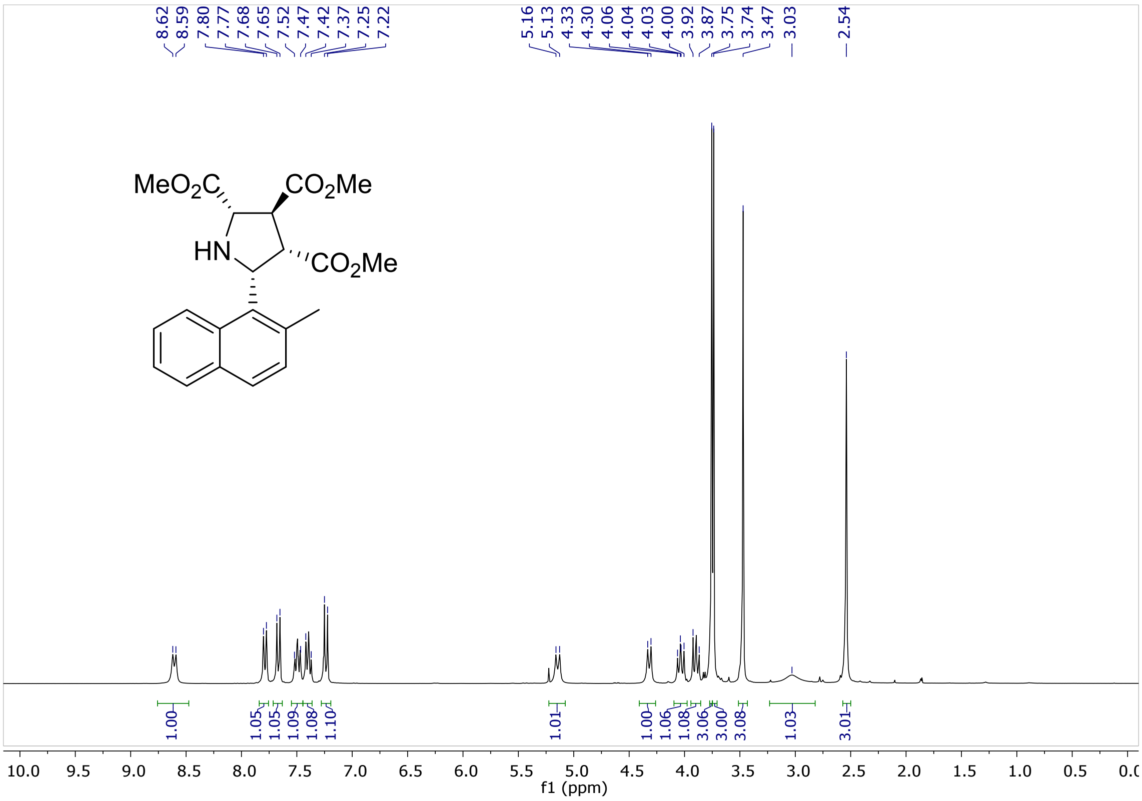

^{13}C NMR ($CDCl_3$, 75 MHz)

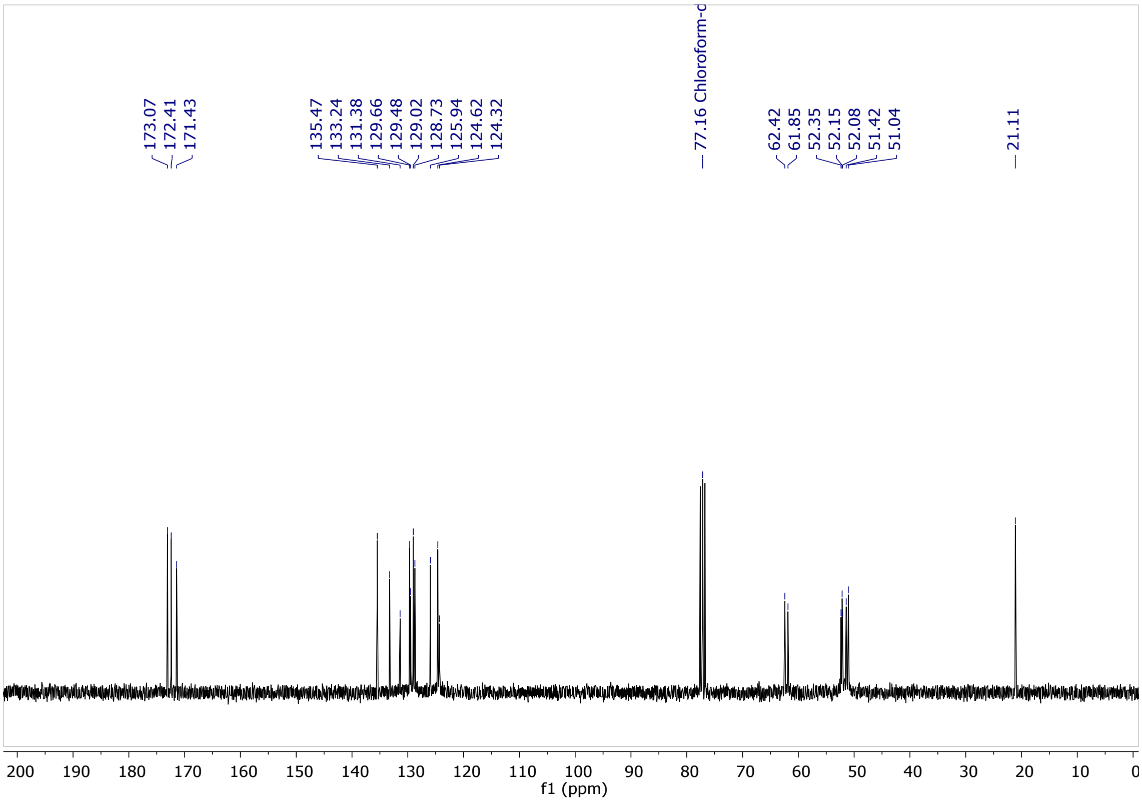

3,4-Di-tert-butyl 2-methyl (2*S*,3*S*,4*S*,5*R*)-5-(2-methylnaphthalen-1-yl)pyrrolidine-2,3,4-tricarboxylate (15c)

^{1}H NMR ($CDCl_3$, 300 MHz)

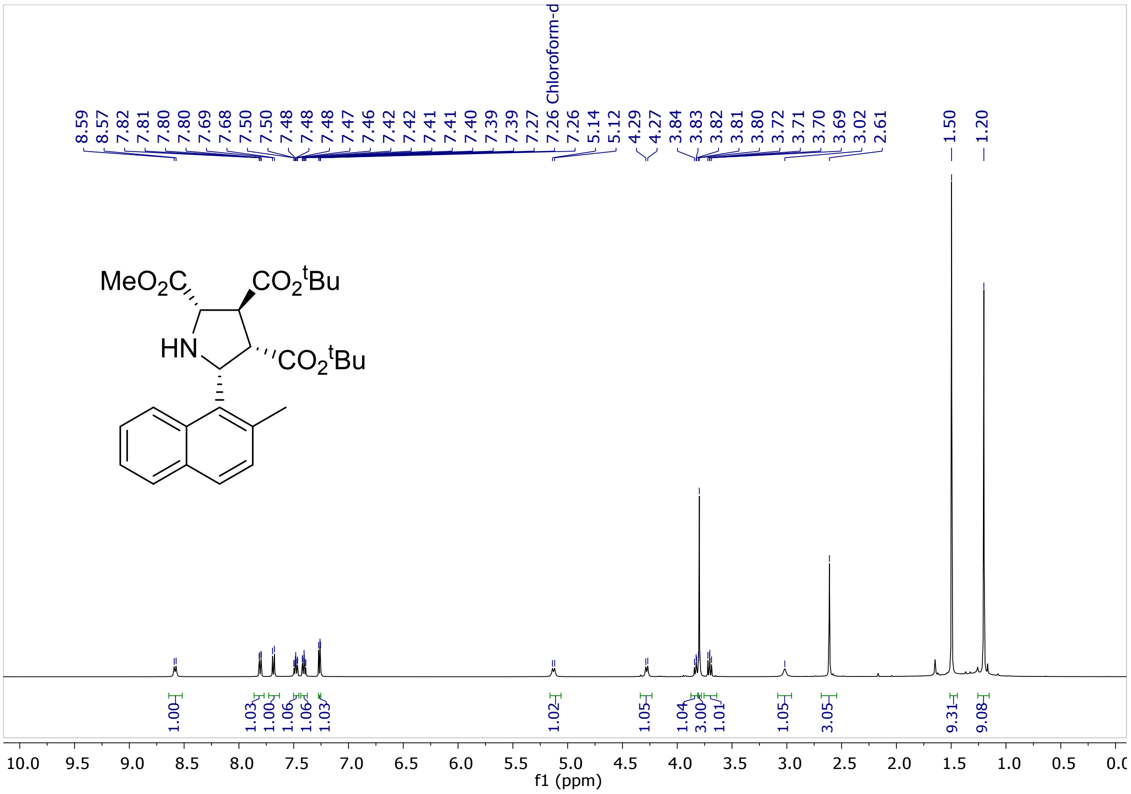

^{13}C NMR ($CDCl_3$, 75 MHz)

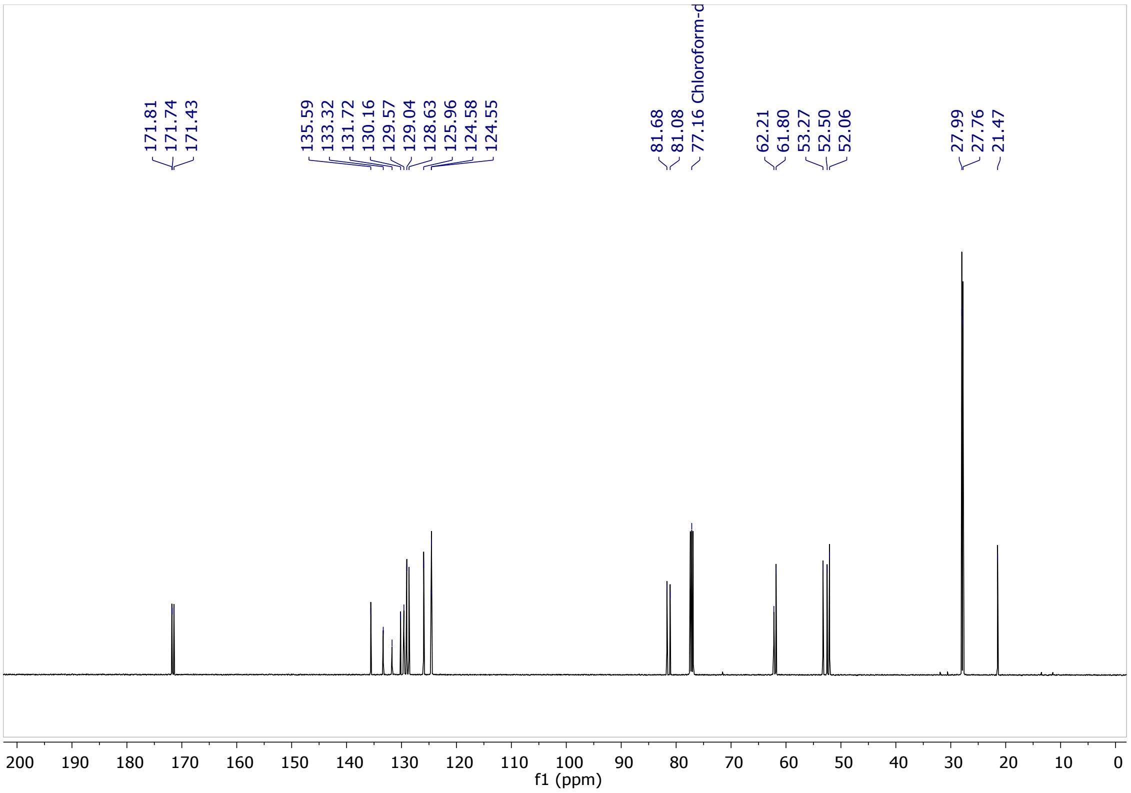

Methyl (2*S*,3*S*,4*S*,5*R*)-3,4-dicyano-5-(2-methylnaphthalen-1-yl)pyrrolidine-2-carboxylate (15d)

^1H NMR ($CDCl_3$, 300 MHz)

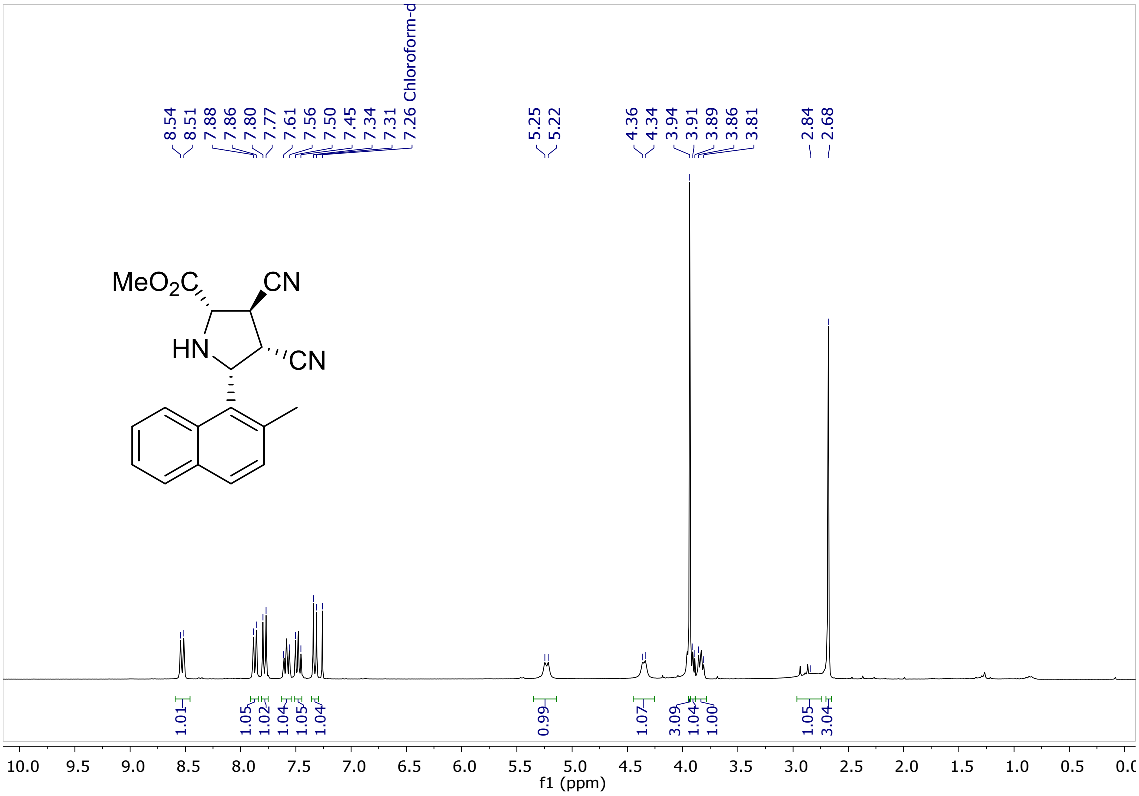

^{13}C NMR ($CDCl_3$, 75 MHz)

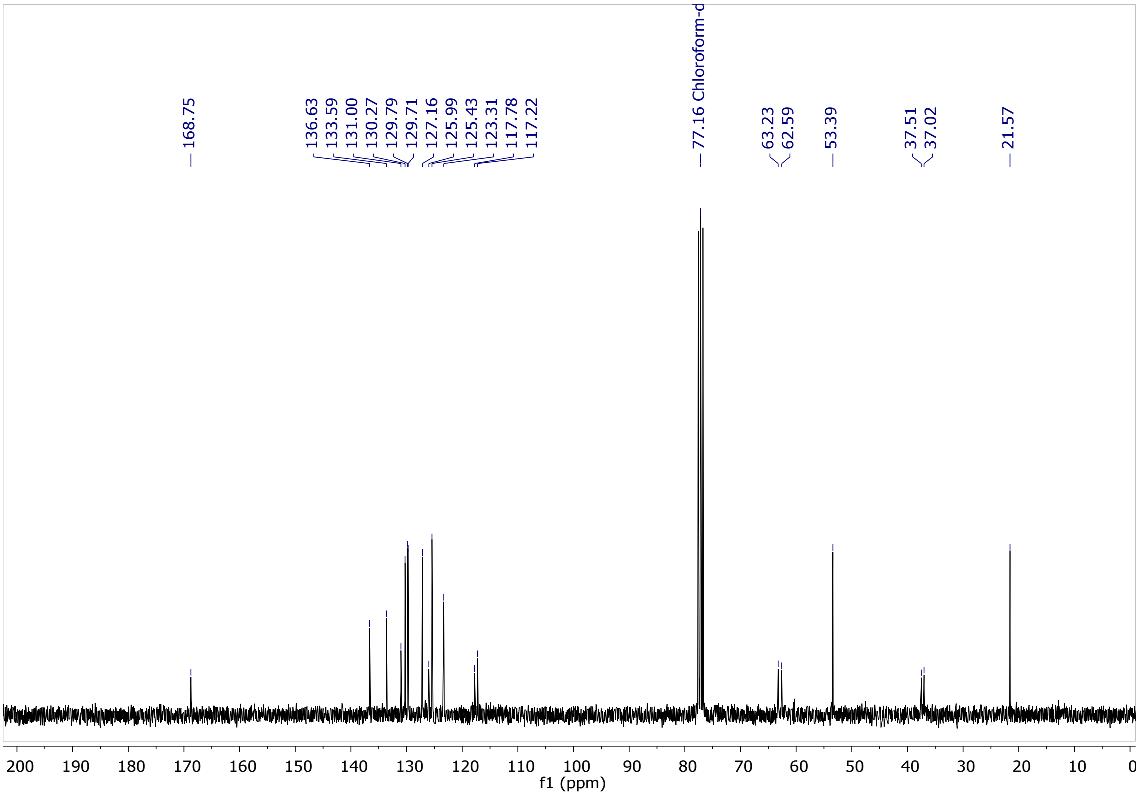

Methyl 5-methyl-3-(2-methylnaphthalen-1-yl)-4,6-dioxo-2-(2,4,6-trimethylbenzyl)-2,4,5,6-tetrahydropyrrolo[3,4-c]pyrrole-1-carboxylate (9a)

^1H NMR ($CDCl_3$, 300 MHz)

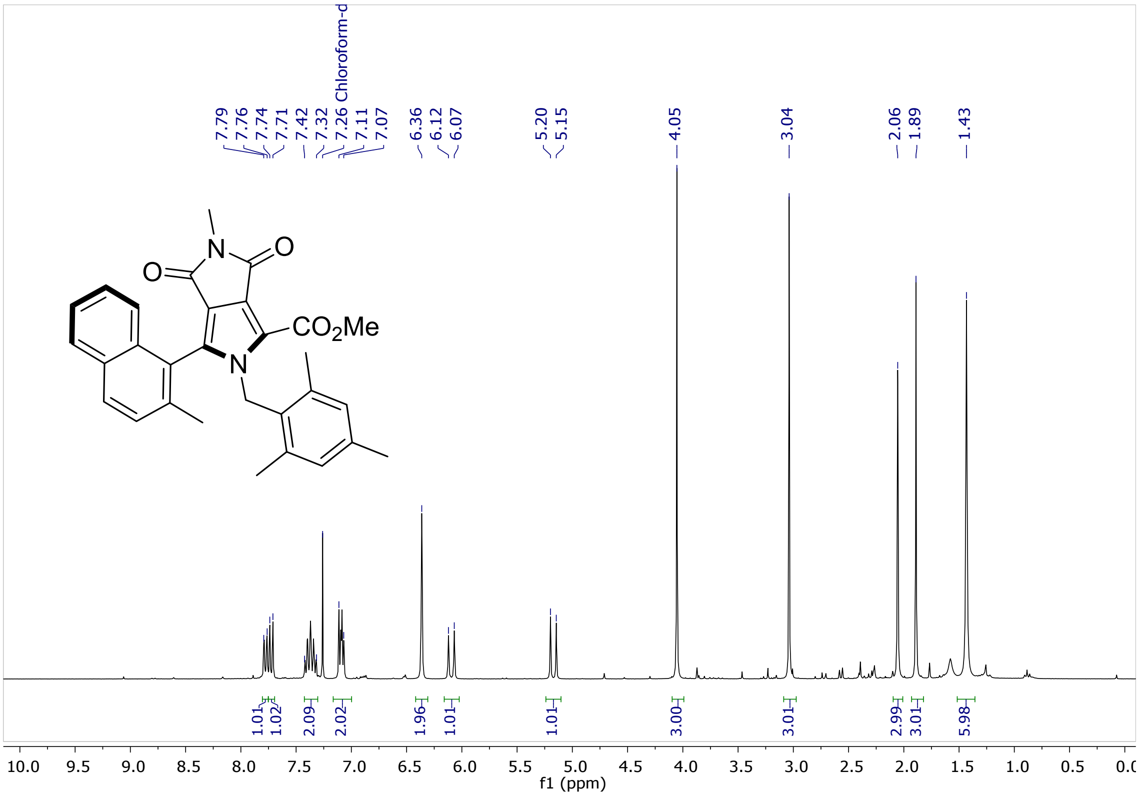

^{13}C NMR ($CDCl_3$, 75 MHz)

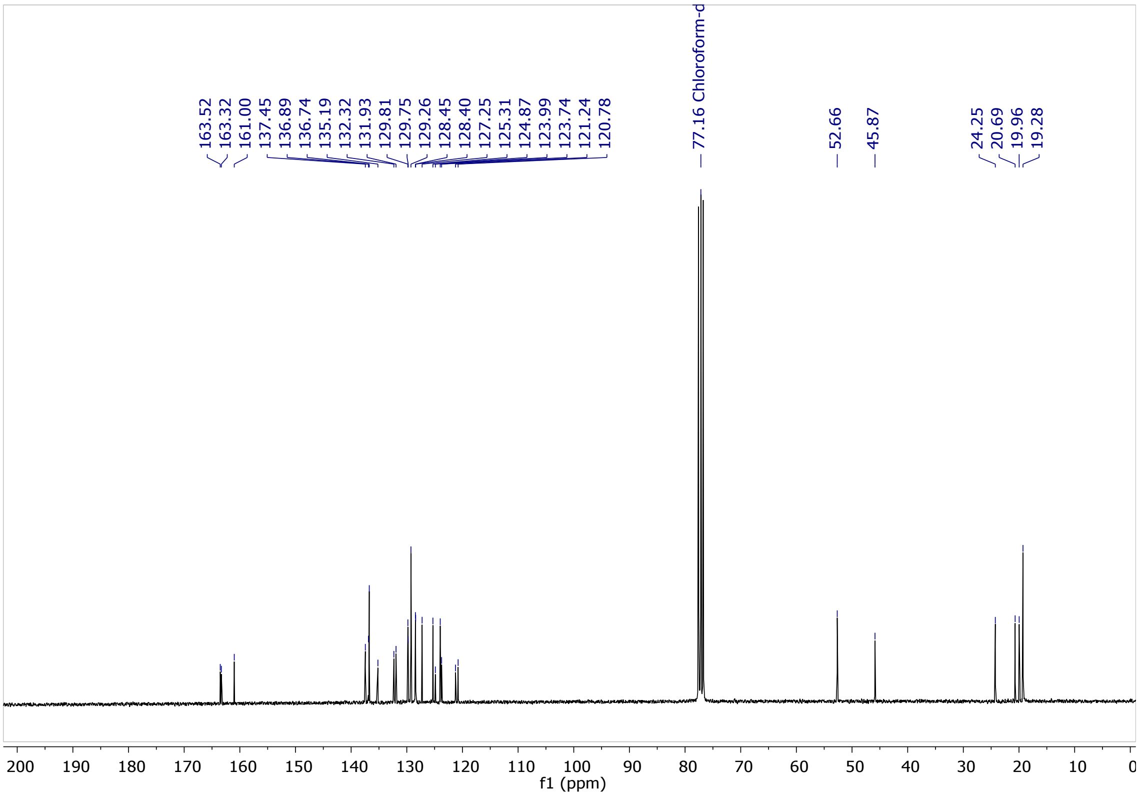

Methyl-3-(2-methoxynaphthalen-1-yl)-5-methyl-4,6-dioxo-2-(2,4,6-trimethylbenzyl)-2,4,5,6-tetrahydropyrrolo[3,4-c]pyrrole-1-carboxylate (9b)

^1H NMR ($CDCl_3$, 300 MHz)

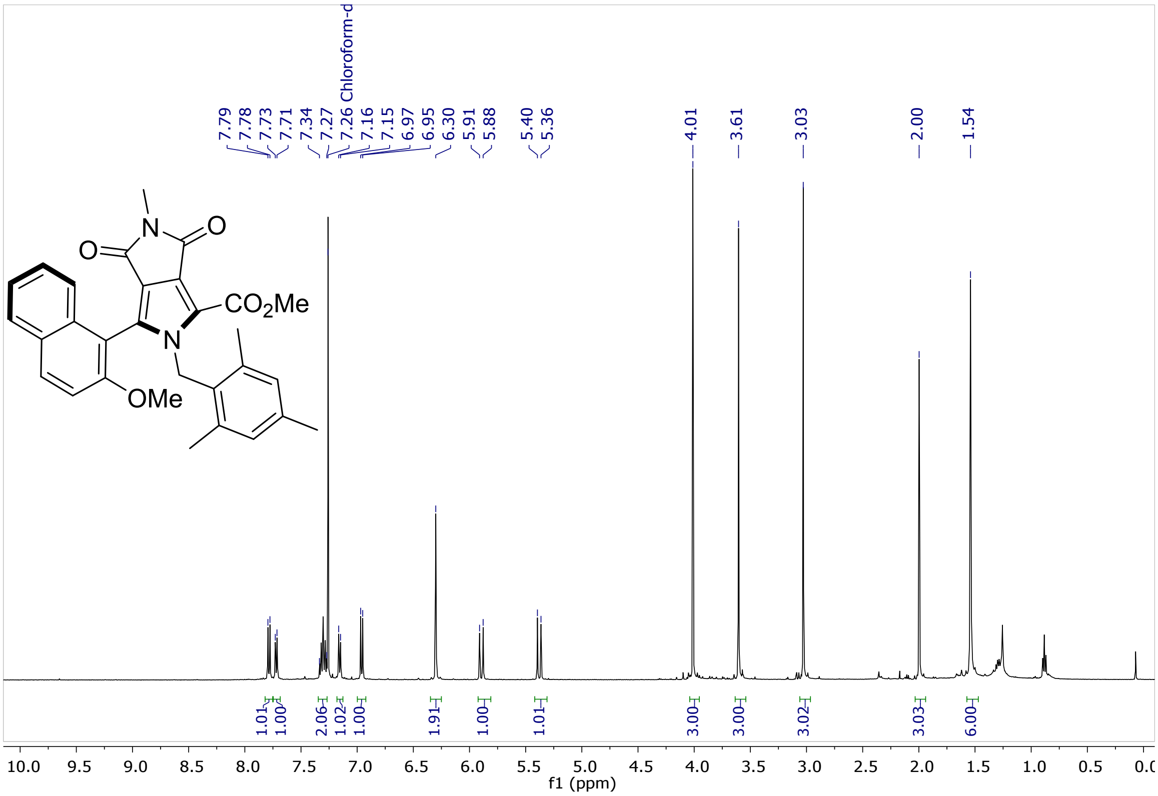

^{13}C NMR ($CDCl_3$, 75 MHz)

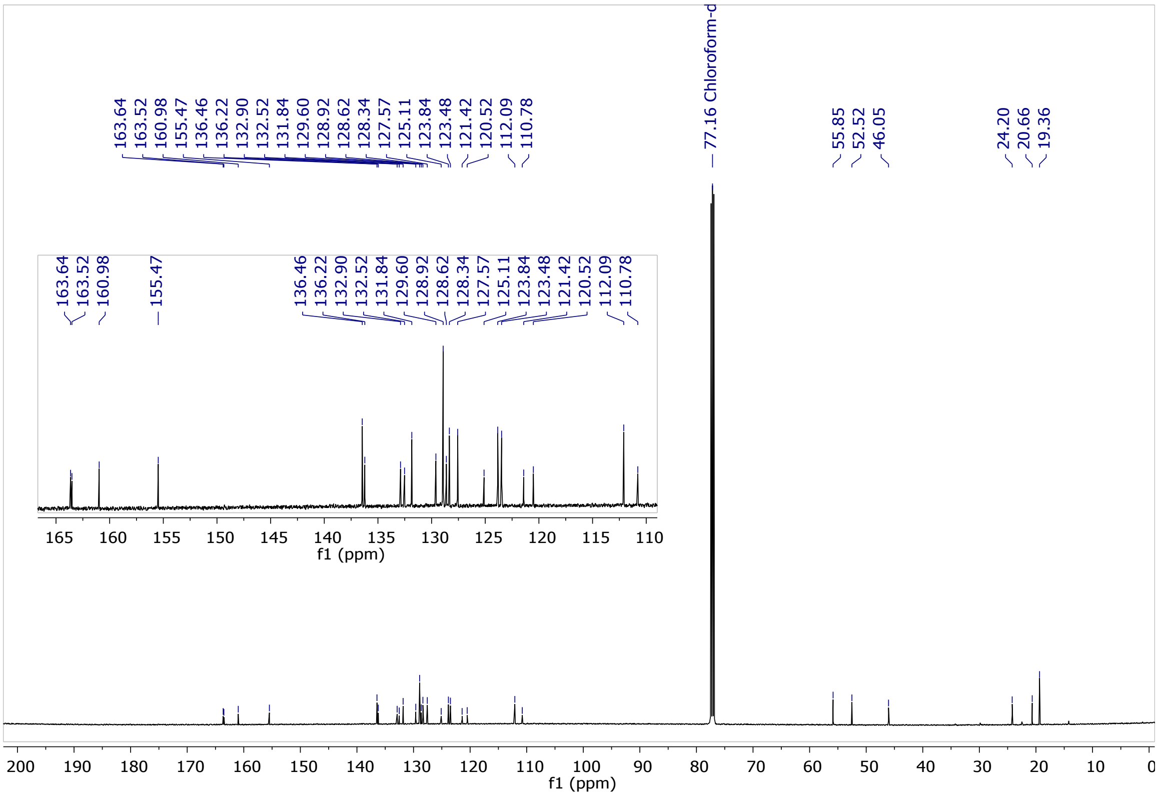

Methyl-3-(2-isopropoxynaphthalen-1-yl)-5-methyl-4,6-dioxo-2-(2,4,6-trimethylbenzyl)-2,4,5,6-tetrahydropyrrolo[3,4-c]pyrrole-1-carboxylate (9c)

^{1}H NMR ($CDCl_3$, 300 MHz)

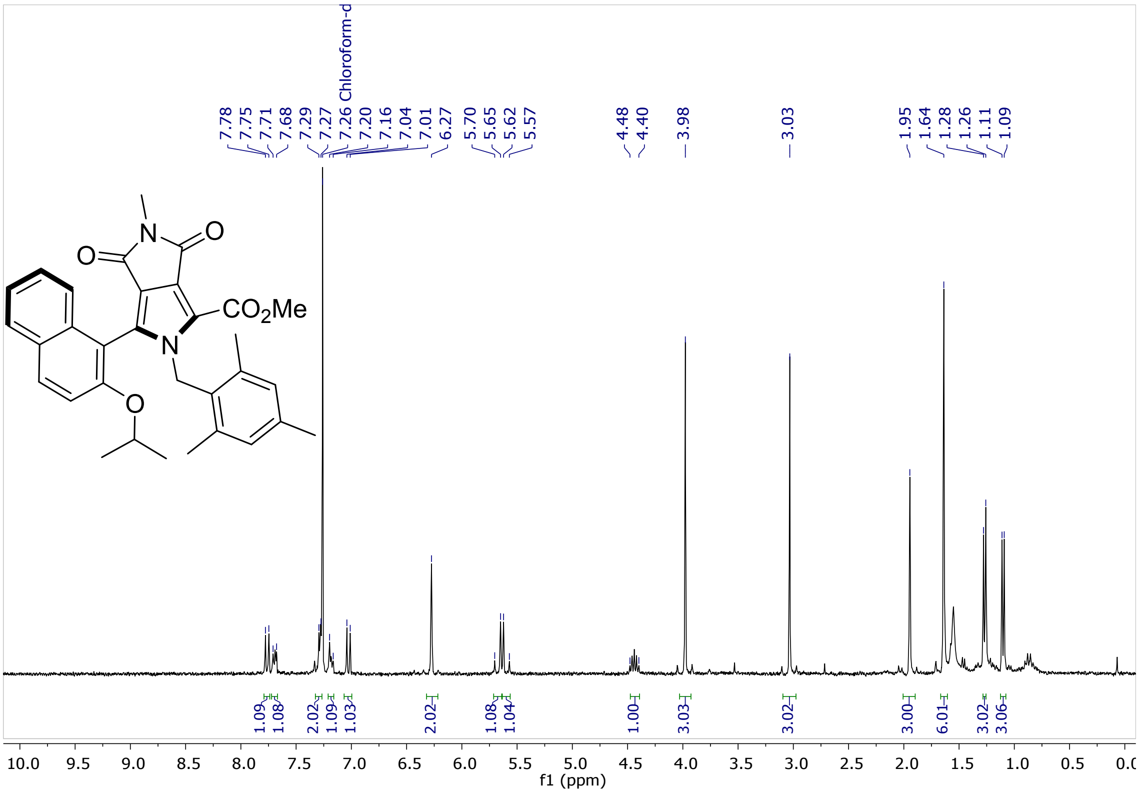

^{13}C NMR ($CDCl_3$, 75 MHz)

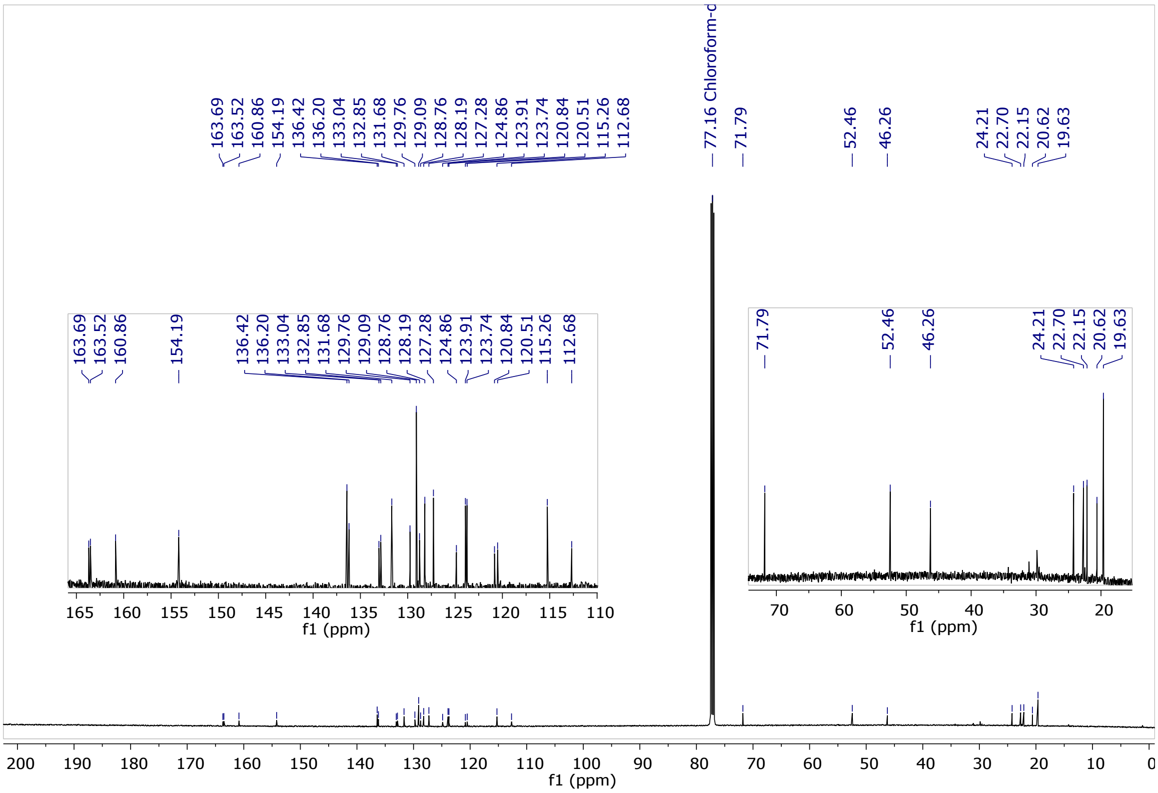

Methyl-5-methyl-4,6-dioxo-3-(2-(tosyloxy)naphthalen-1-yl)-2-(2,4,6-trimethylbenzyl)-2,4,5,6-tetrahydropyrrolo[3,4-c]pyrrole-1-carboxylate (9d)

^{1}H NMR ($CDCl_3$, 300 MHz)

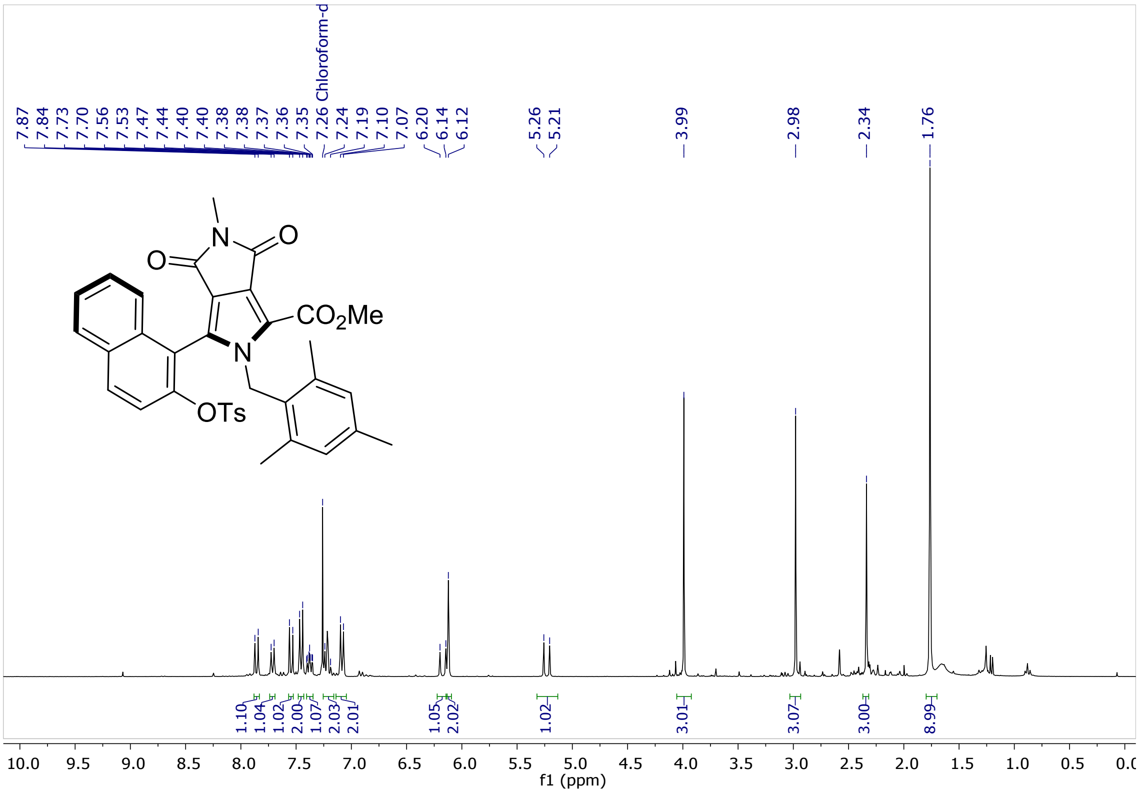

^{13}C NMR ($CDCl_3$, 75 MHz)

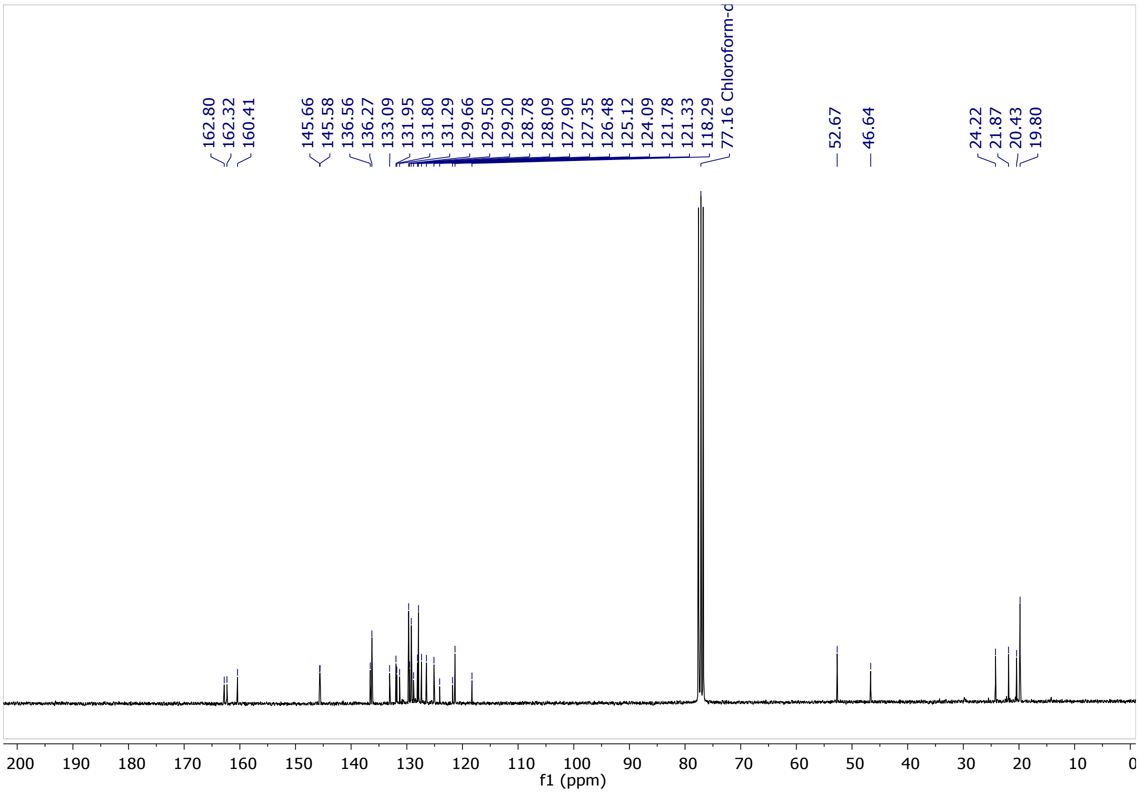

Methyl 5-methyl-4,6-dioxo-3-(2-phenylnaphthalen-1-yl)-2-(2,4,6-trimethylbenzyl)-2,4,5,6-tetrahydropyrrolo[3,4-c]pyrrole-1-carboxylate (9e)

^{1}H NMR ($CDCl_3$, 300 MHz)

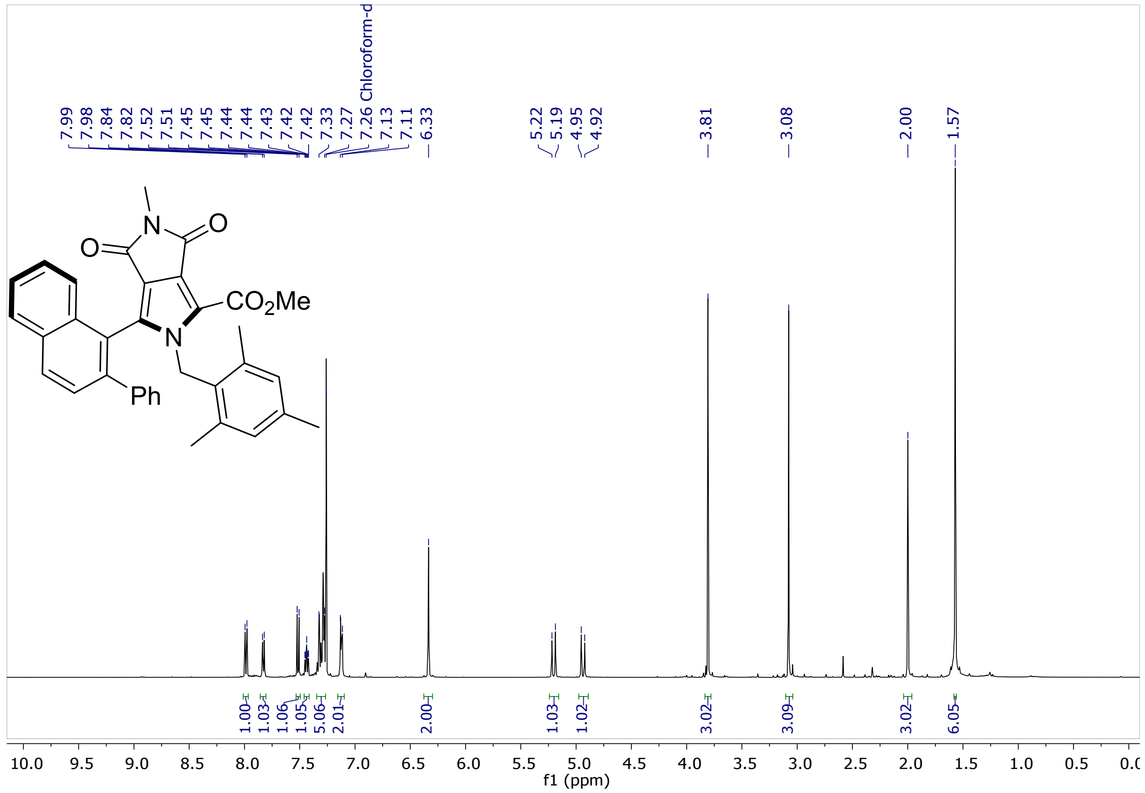

^{13}C NMR ($CDCl_3$, 75 MHz)

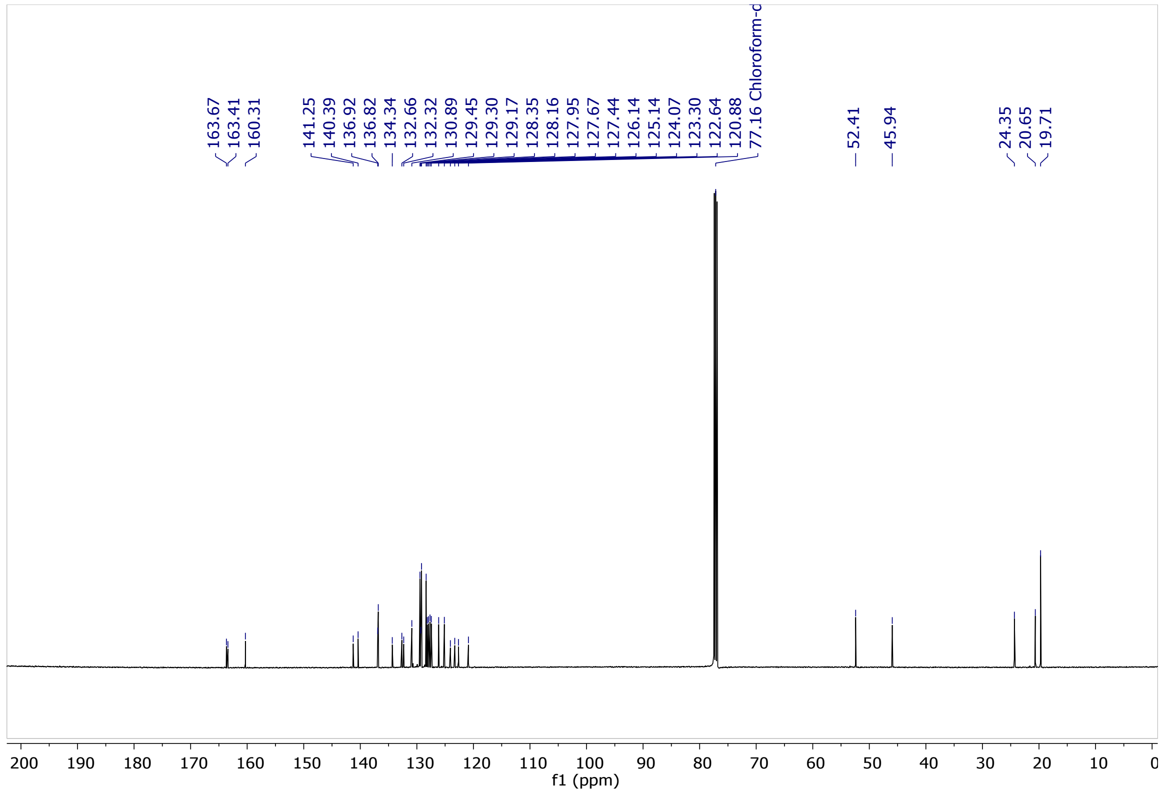

Methyl 3-(2-bromonaphthalen-1-yl)-5-methyl-4,6-dioxo-2-(2,4,6-trimethylbenzyl)-2,4,5,6-tetrahydropyrrolo[3,4-c]pyrrole-1-carboxylate (9f)

^{1}H NMR ($CDCl_3$, 300 MHz)

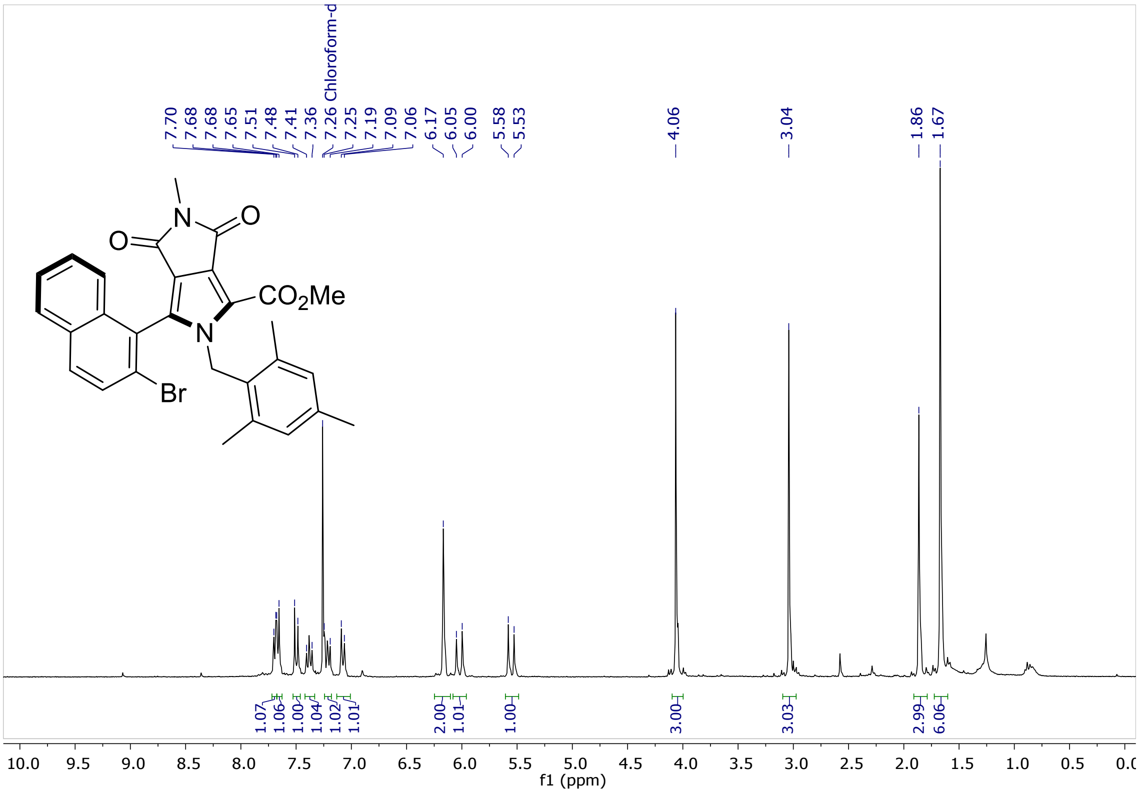

^{13}C NMR ($CDCl_3$, 75 MHz)

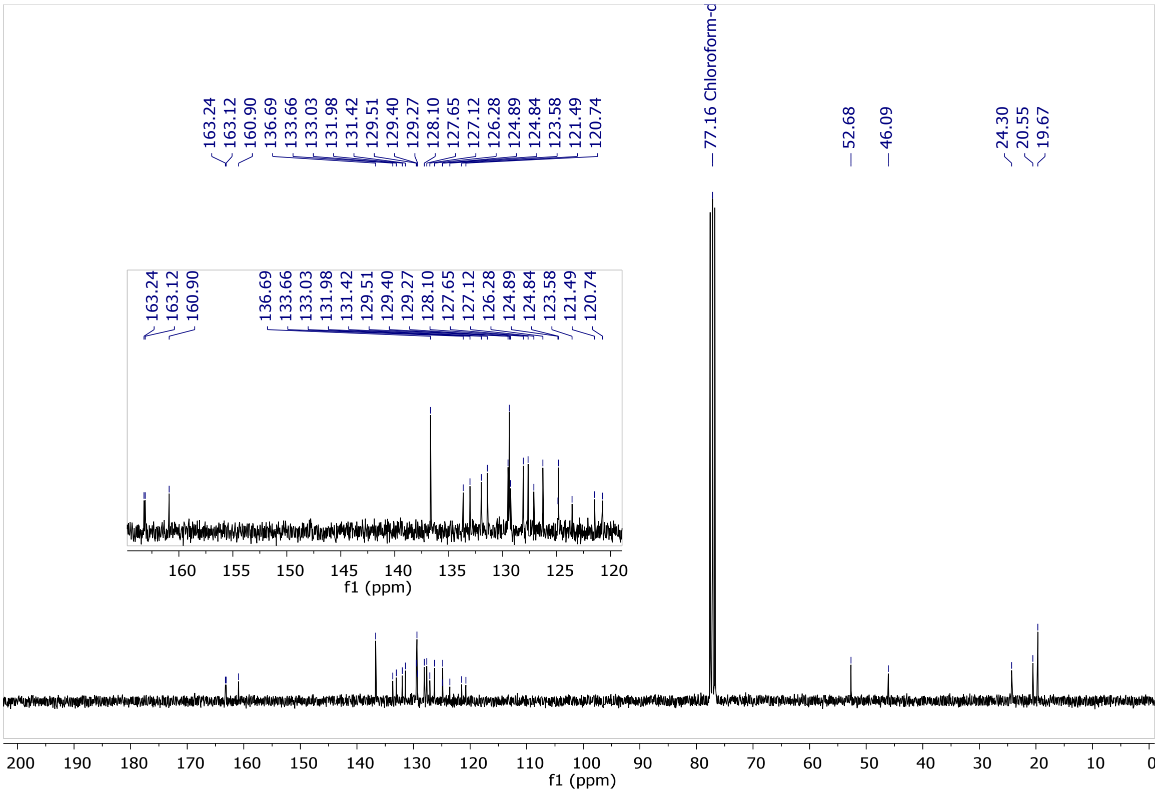

Methyl 5-methyl-3-(naphthalen-1-yl)-4,6-dioxo-2-(2,4,6-trimethylbenzyl)-2,4,5,6-tetrahydropyrrolo[3,4-c]pyrrole-1-carboxylate (9g)

^{1}H NMR ($CDCl_3$, 500 MHz)

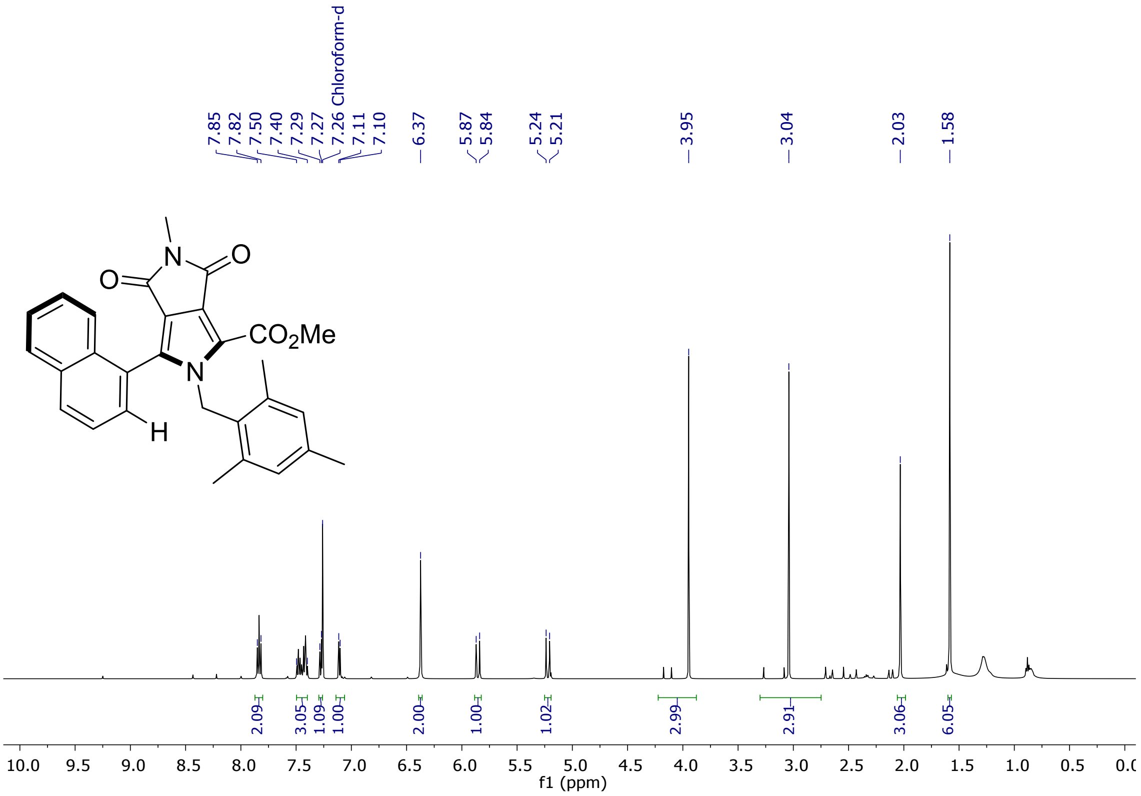

^{13}C NMR ($CDCl_3$, 126 MHz)

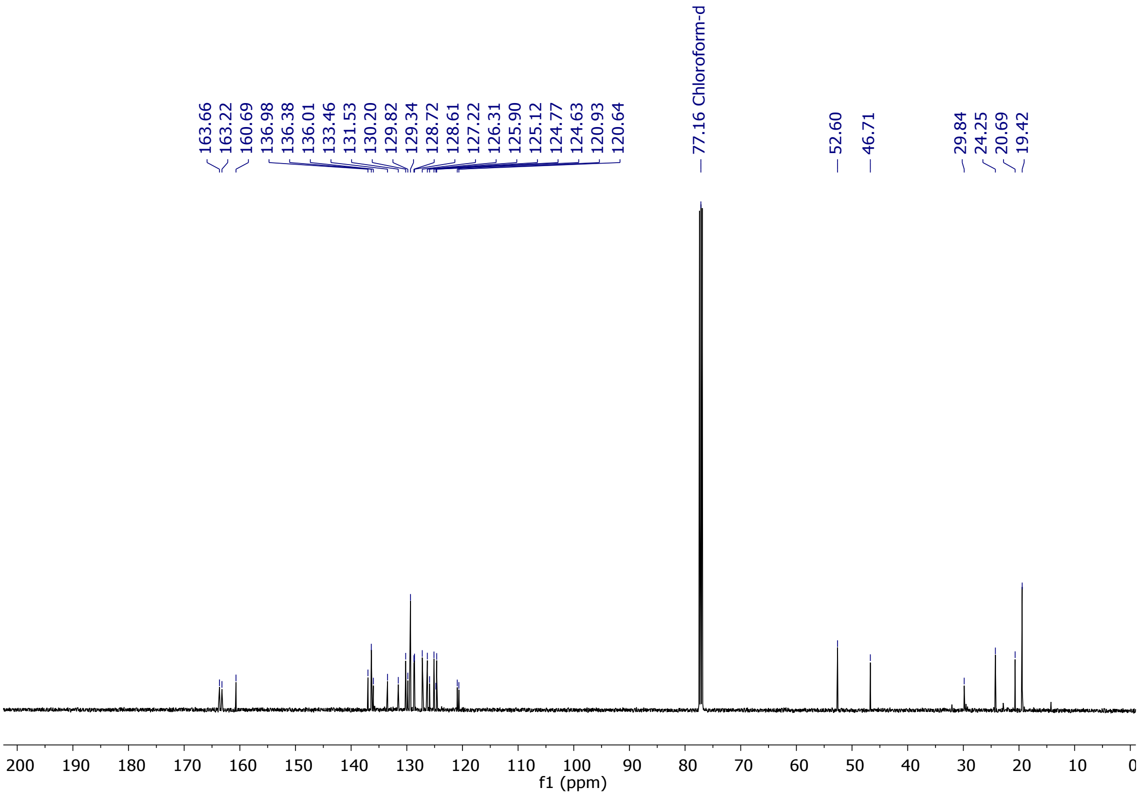

Methyl 3-(1-bromonaphthalen-2-yl)-5-methyl-4,6-dioxo-2-(2,4,6-trimethylbenzyl)-2,4,5,6-tetrahydropyrrolo[3,4-c]pyrrole-1-carboxylate (13a)

^1H NMR ($CDCl_3$, 300 MHz)

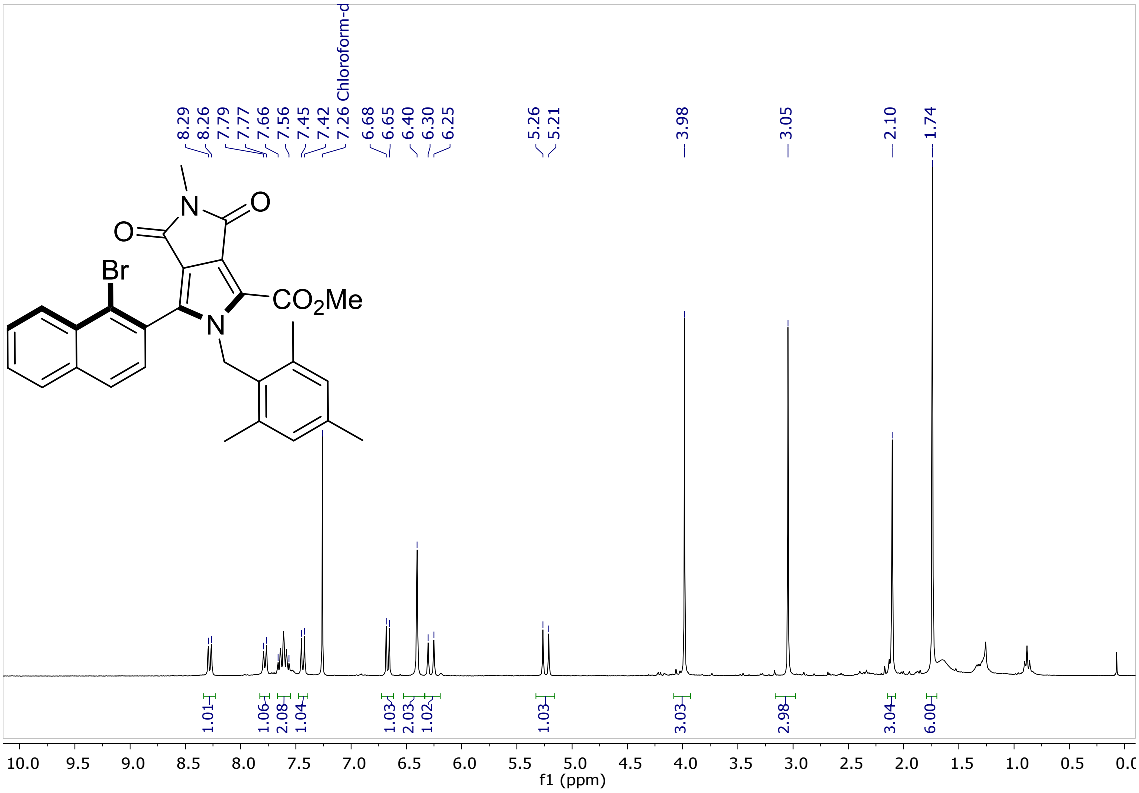

^{13}C NMR ($CDCl_3$, 75 MHz)

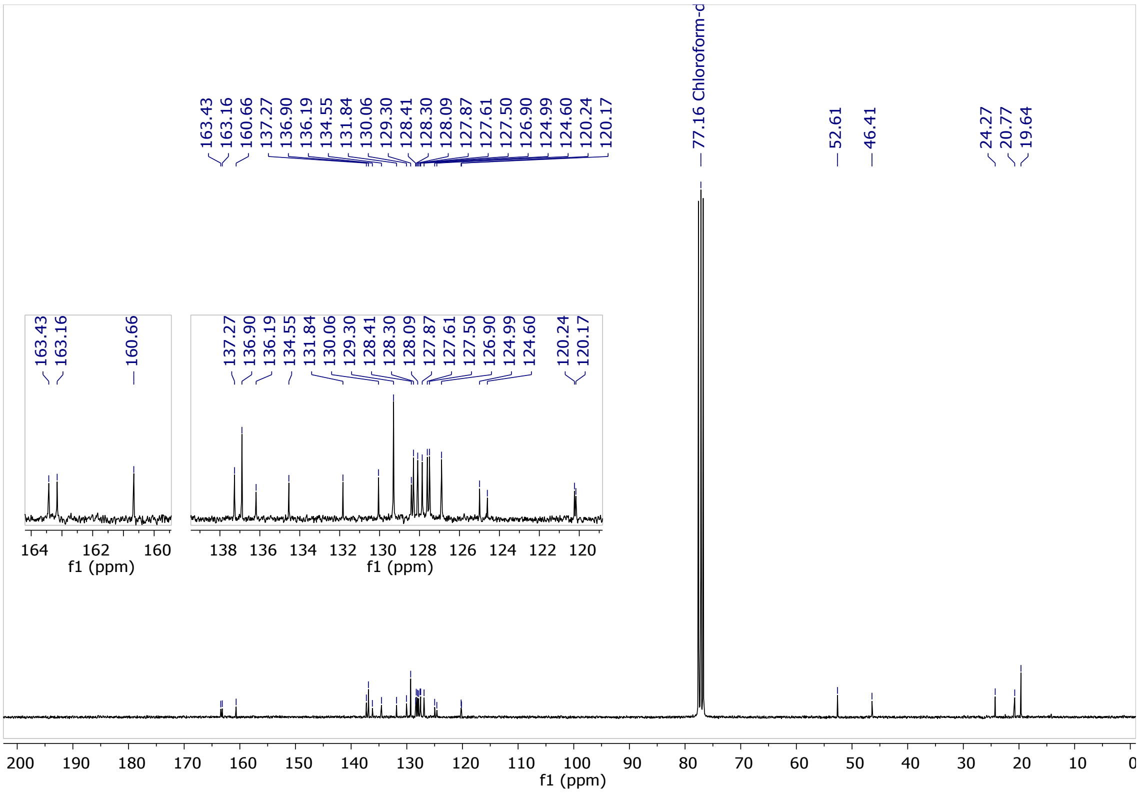

Methyl 3-(1-iodonaphthalen-2-yl)-5-methyl-4,6-dioxo-2-(2,4,6-trimethylbenzyl)-2,4,5,6-tetrahydropyrrolo[3,4-c]pyrrole-1-carboxylate (13b)

^{1}H NMR ($CDCl_3$, 300 MHz)

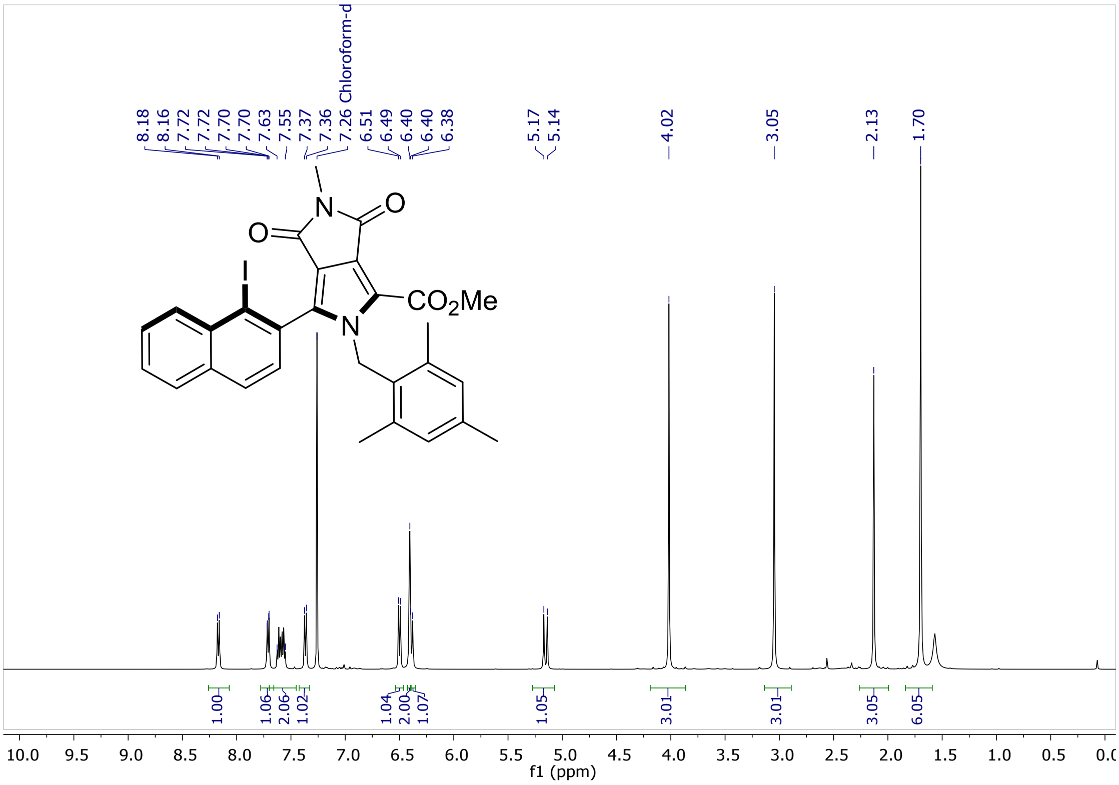

^{13}C NMR ($CDCl_3$, 126 MHz)

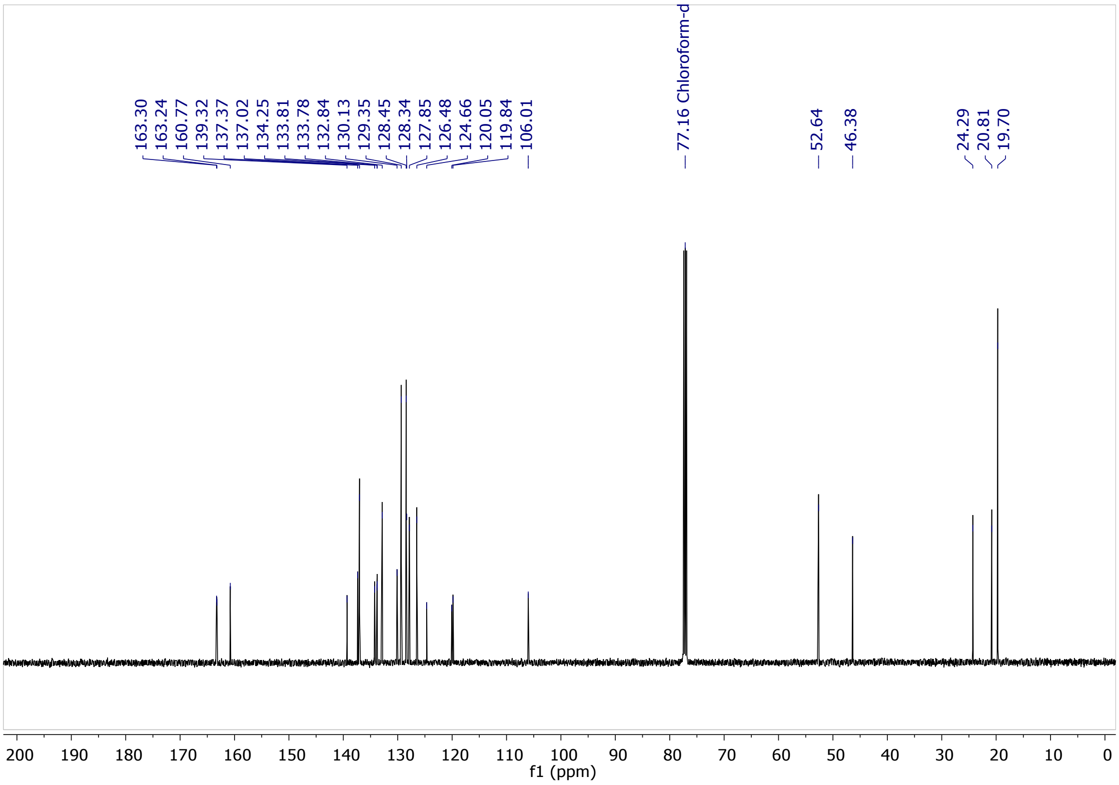

Methyl 3-(1-ethylnaphthalen-2-yl)-5-methyl-4,6-dioxo-2-(2,4,6-trimethylbenzyl)-2,4,5,6-tetrahydropyrrolo[3,4-c]pyrrole-1-carboxylate (13c)

^{1}H NMR ($CDCl_3$, 300 MHz)

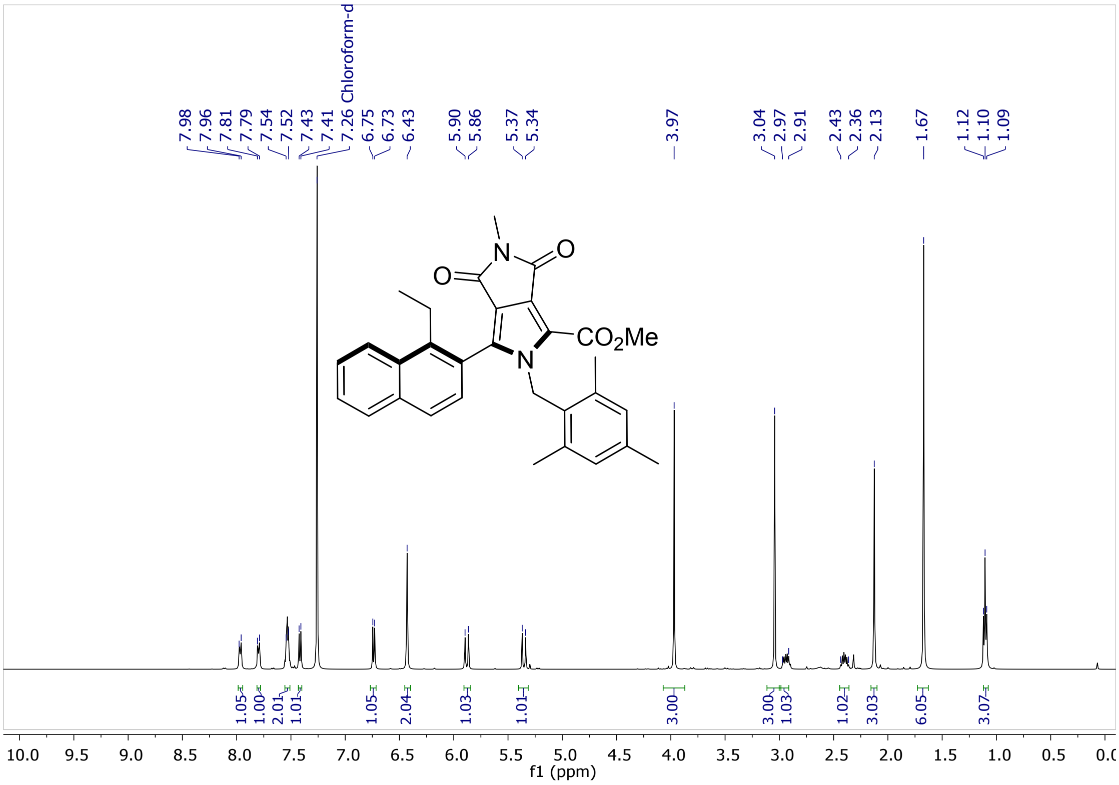

^{13}C NMR ($CDCl_3$, 126 MHz)

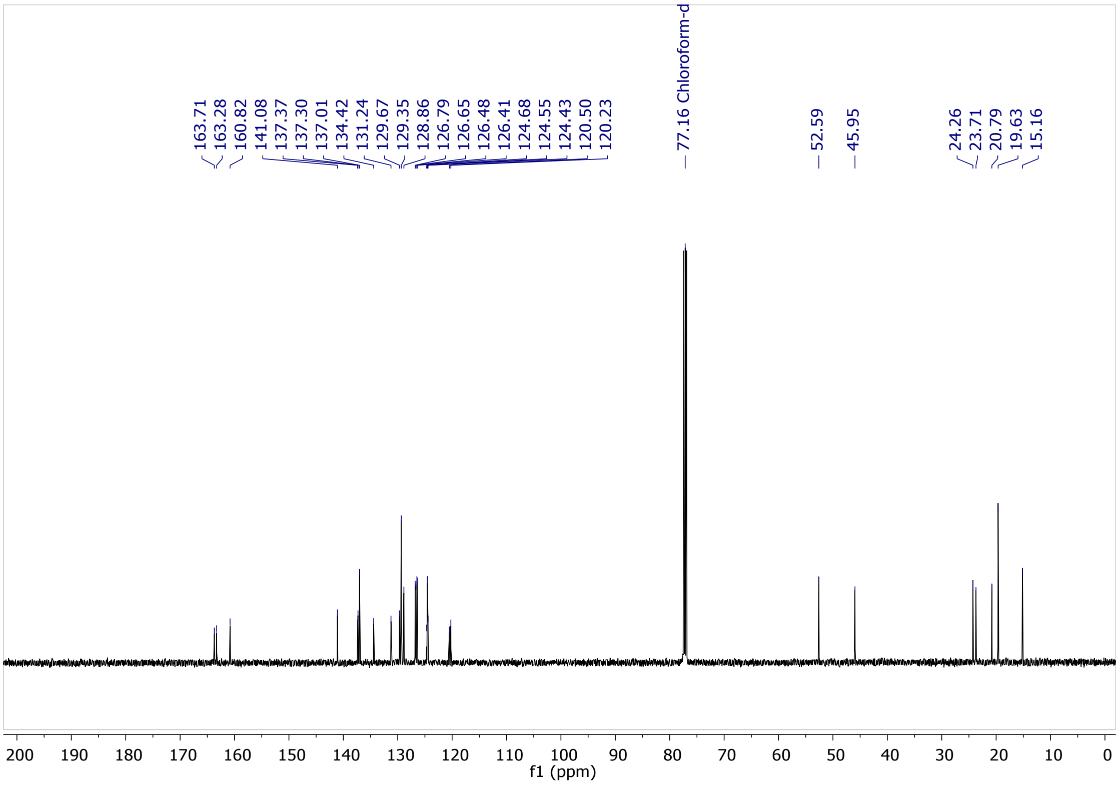

(±)-Methyl 3-(1-(4-methoxyphenyl)naphthalen-2-yl)-5-methyl-4,6-dioxo-2-(2,4,6-trimethylbenzyl)-2,4,5,6-tetrahydropyrrolo[3,4-c]pyrrole-1-carboxylate (13d)

^{1}H NMR ($CDCl_3$, 300 MHz)

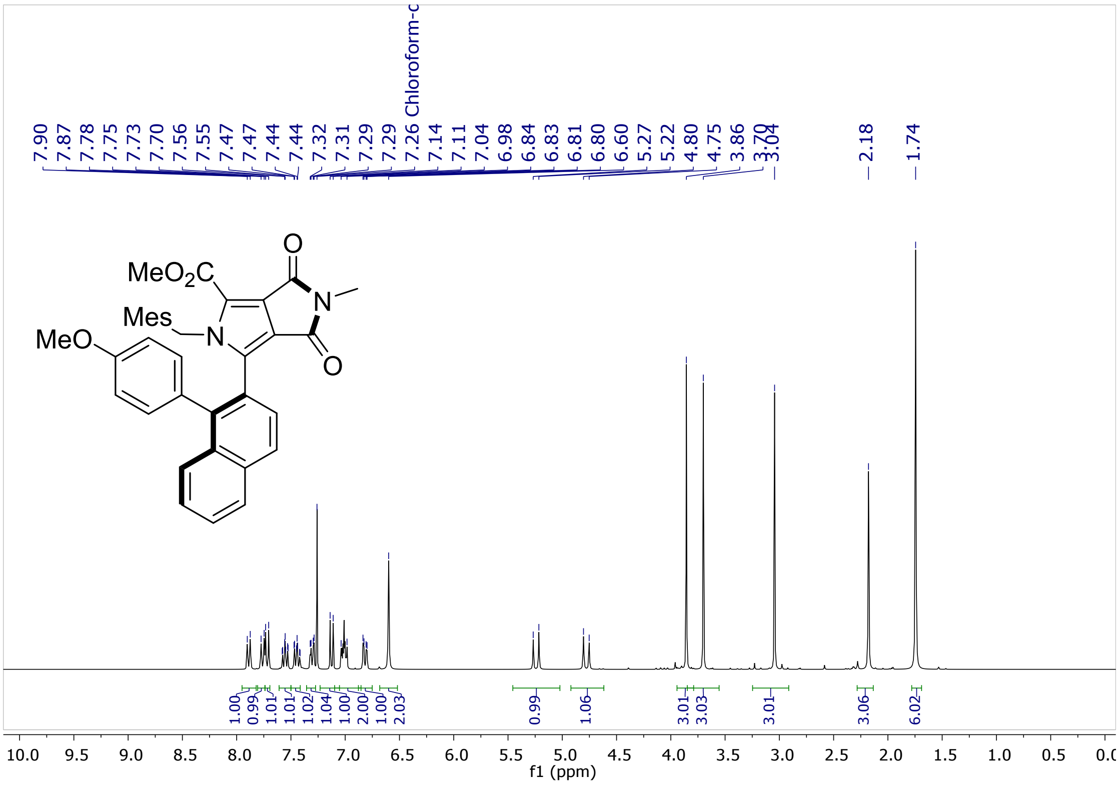

^{13}C NMR ($CDCl_3$, 126 MHz)

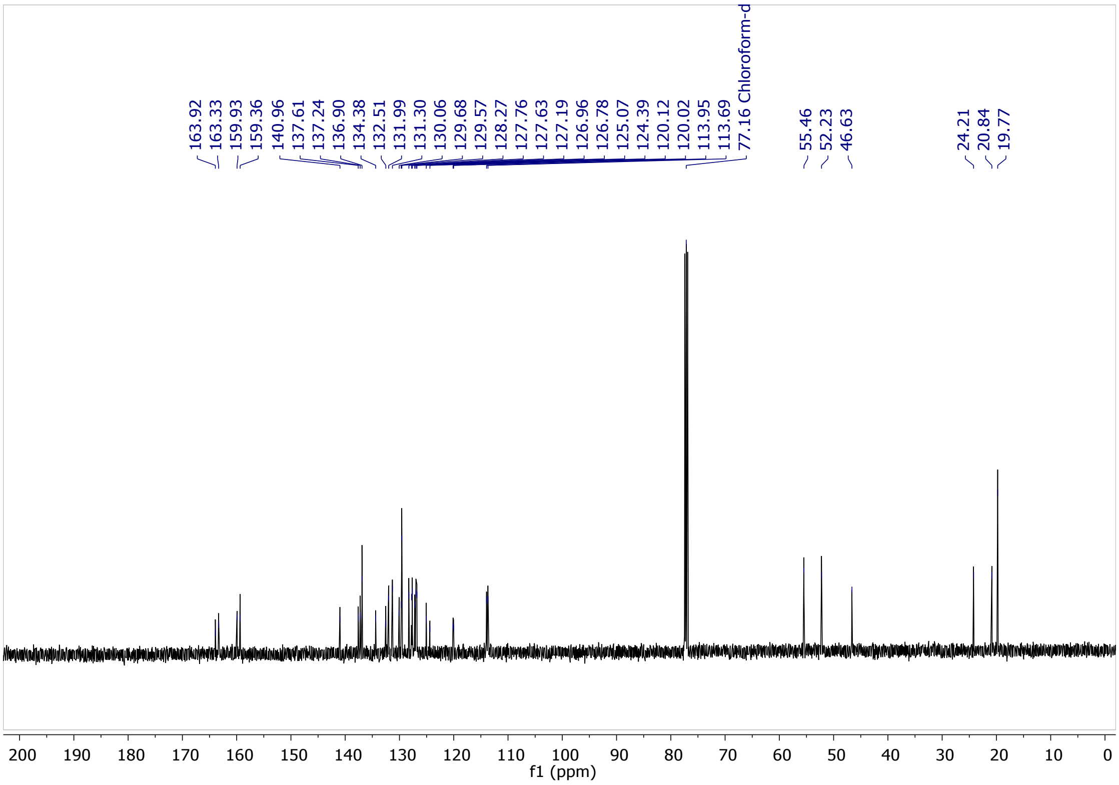

Methyl 3-(1-methoxynaphthalen-2-yl)-5-methyl-4,6-dioxo-2-(2,4,6-trimethylbenzyl)-2,4,5,6-tetrahydropyrrolo[3,4-c]pyrrole-1-carboxylate (13e)

^{1}H NMR ($CDCl_3$, 300 MHz)

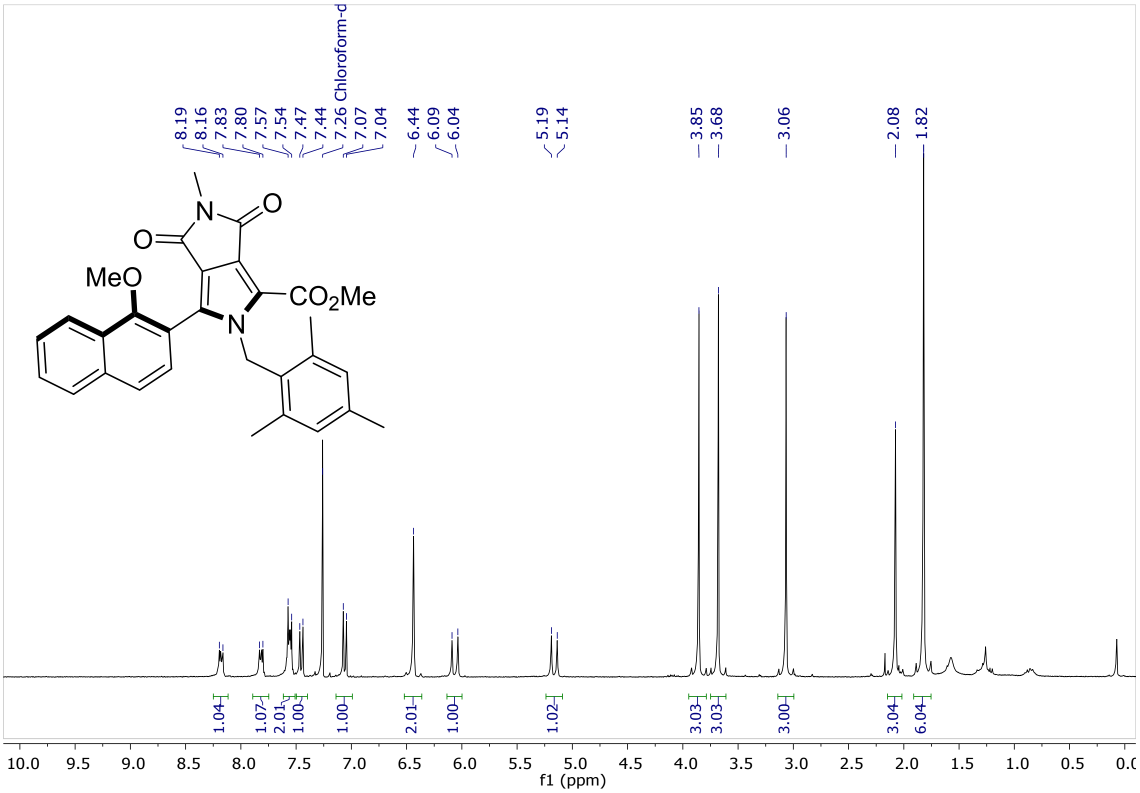

^{13}C NMR ($CDCl_3$, 75 MHz)

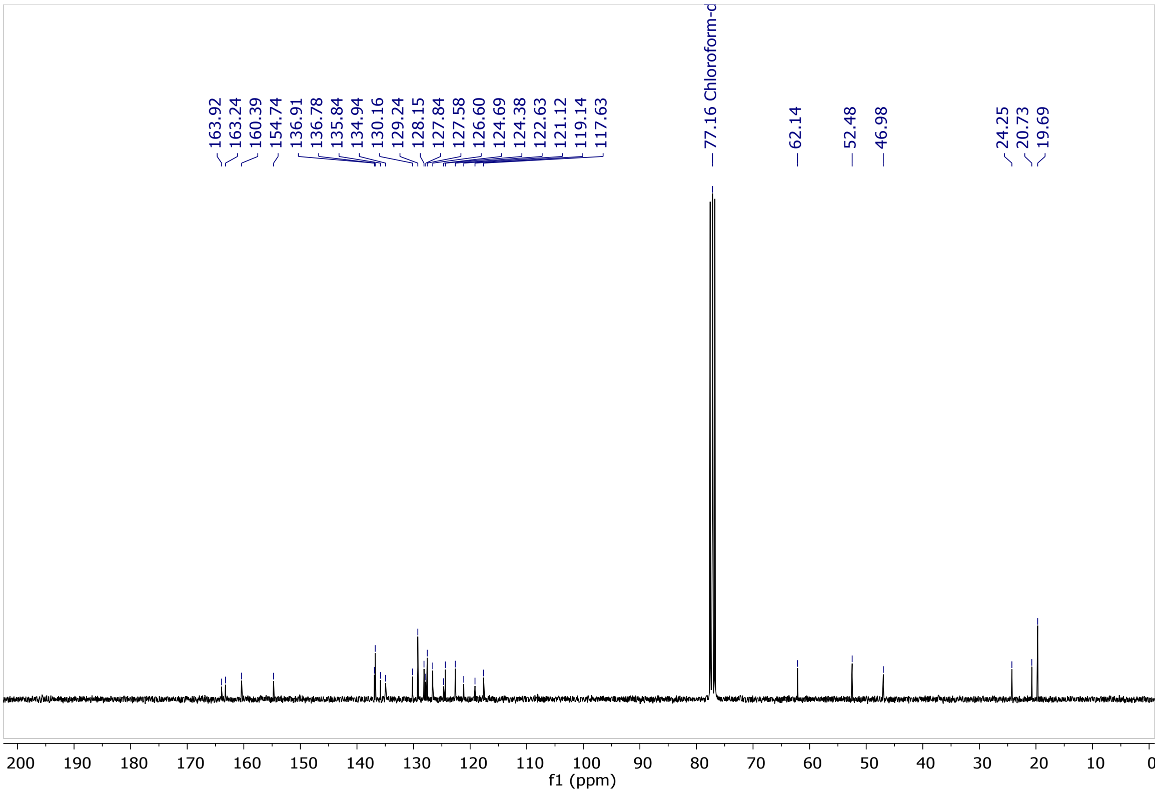

Methyl 3-(1-isopropoxynaphthalen-2-yl)-5-methyl-4,6-dioxo-2-(2,4,6-trimethylbenzyl)-2,4,5,6-tetrahydropyrrolo[3,4-c]pyrrole-1-carboxylate (13f)

^{1}H NMR ($CDCl_3$, 300 MHz)

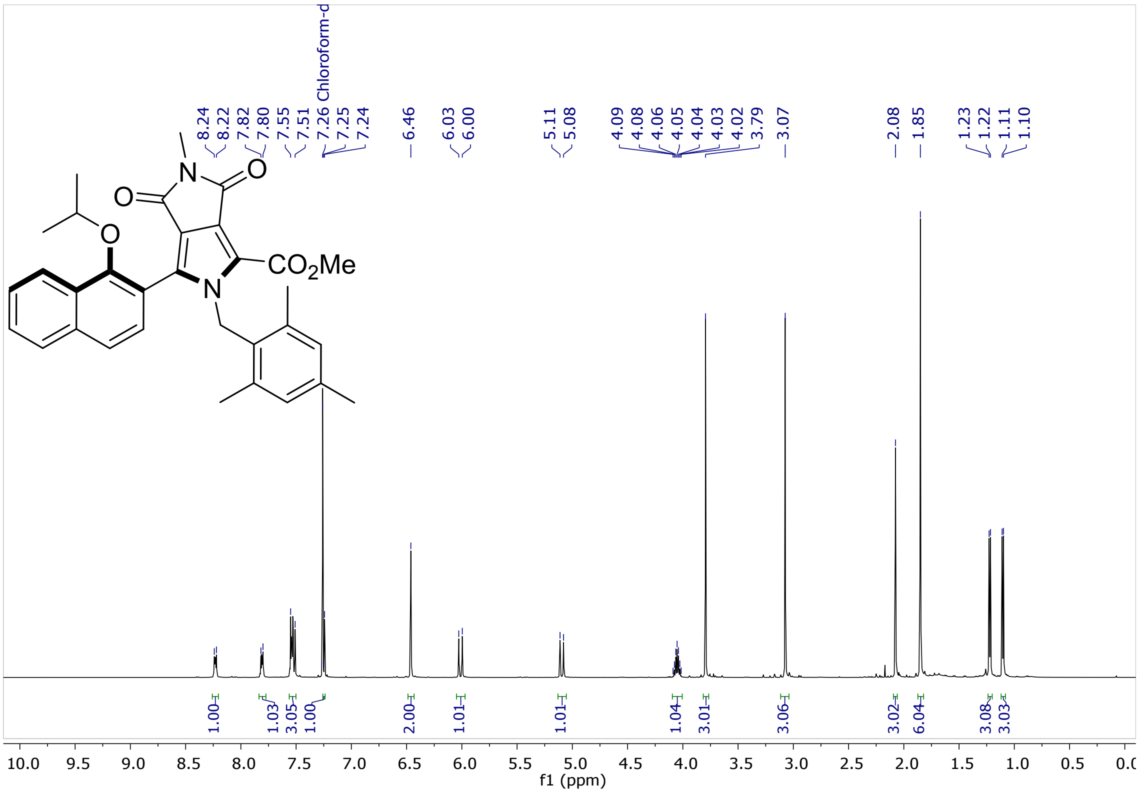

^{13}C NMR ($CDCl_3$, 75 MHz)

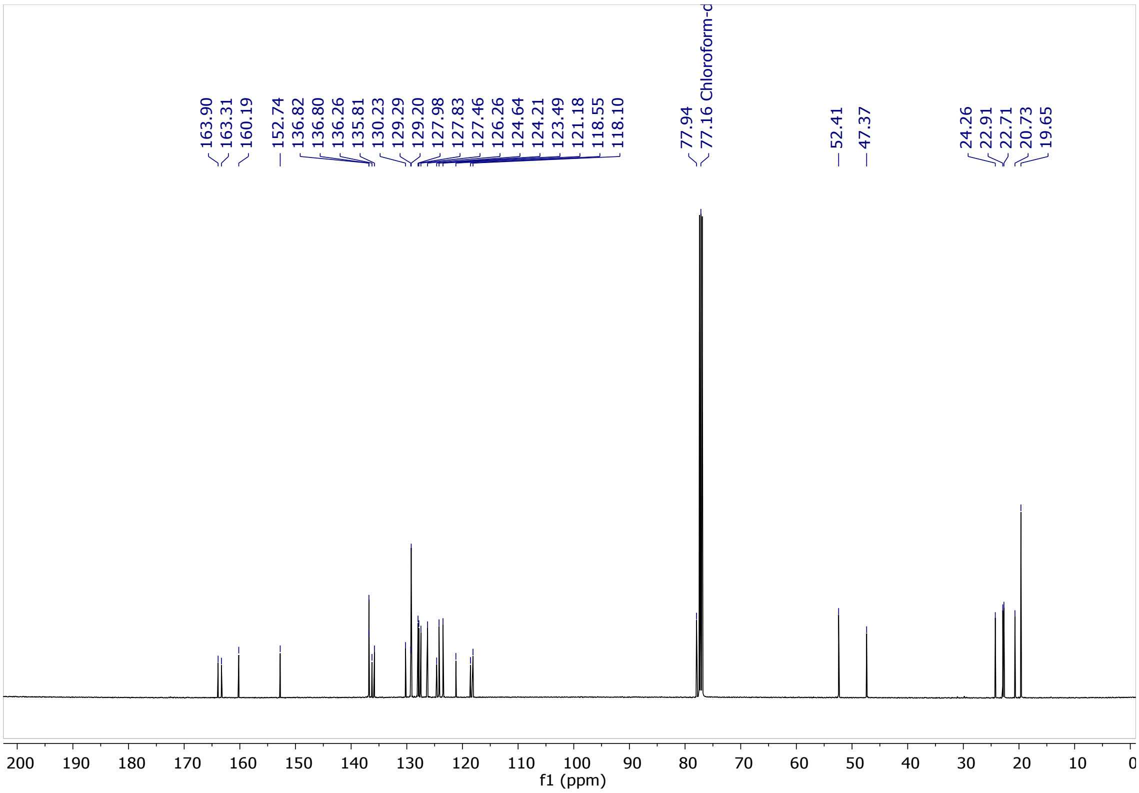

Methyl 3-(2-methylnaphthalen-1-yl)-4,6-dioxo-5-phenyl-2-(2,4,6-trimethylbenzyl)-2,4,5,6-tetrahydropyrrolo[3,4-c]pyrrole-1-carboxylate (17a)

^{1}H NMR ($CDCl_3$, 300 MHz)

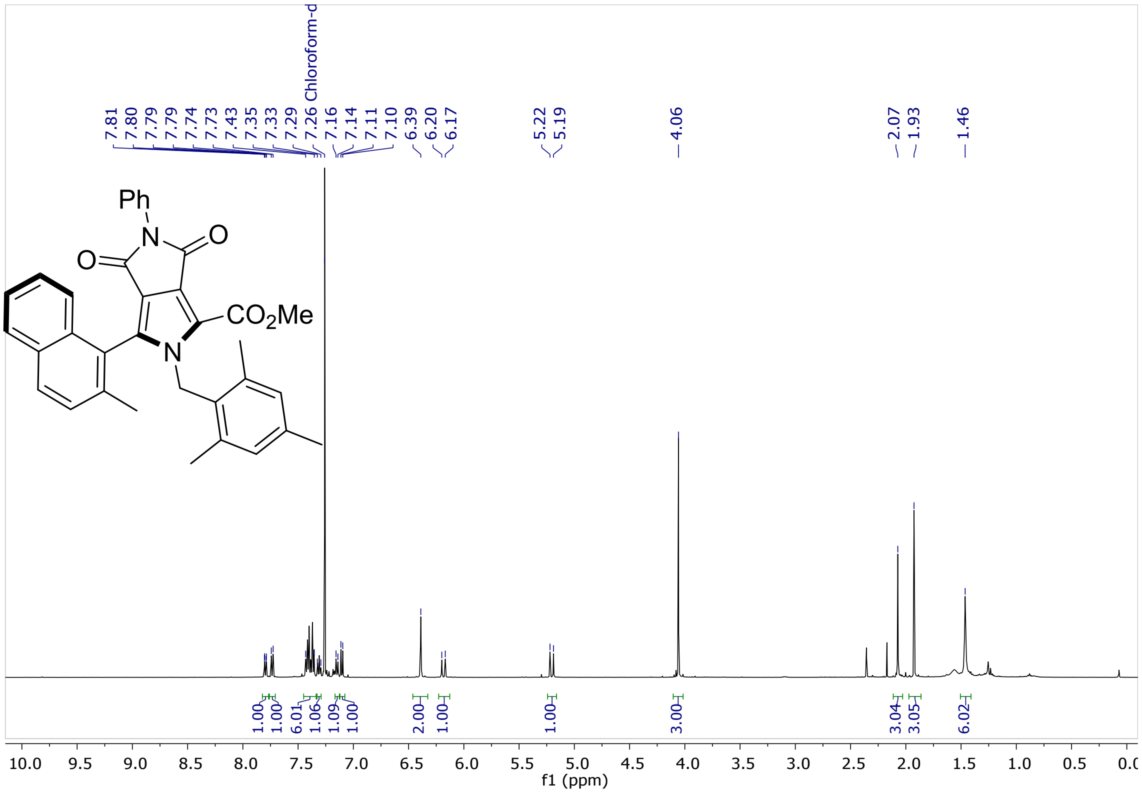

^{13}C NMR ($CDCl_3$, 75 MHz)

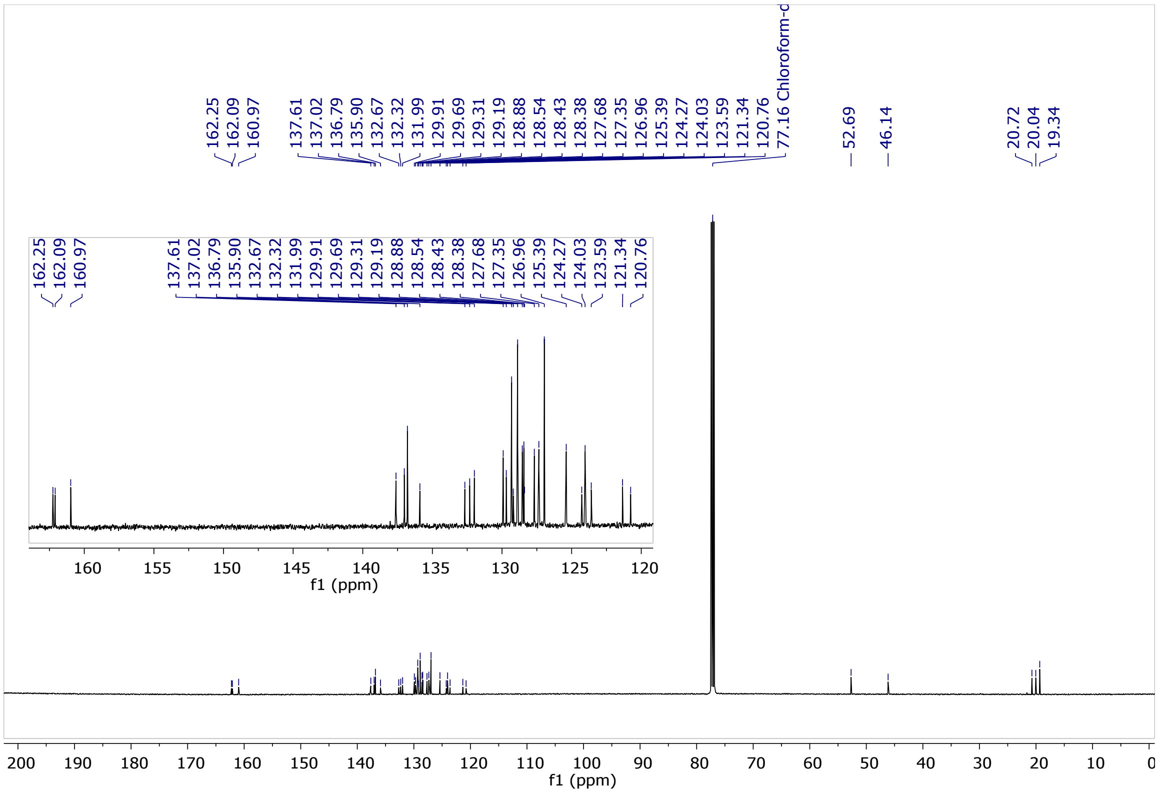

Trimethyl 5-(2-methylnaphthalen-1-yl)-1-(2,4,6-trimethylbenzyl)-1H-pyrrole-2,3,4-tricarboxylate (17b)

^{1}H NMR ($CDCl_3$, 300 MHz)

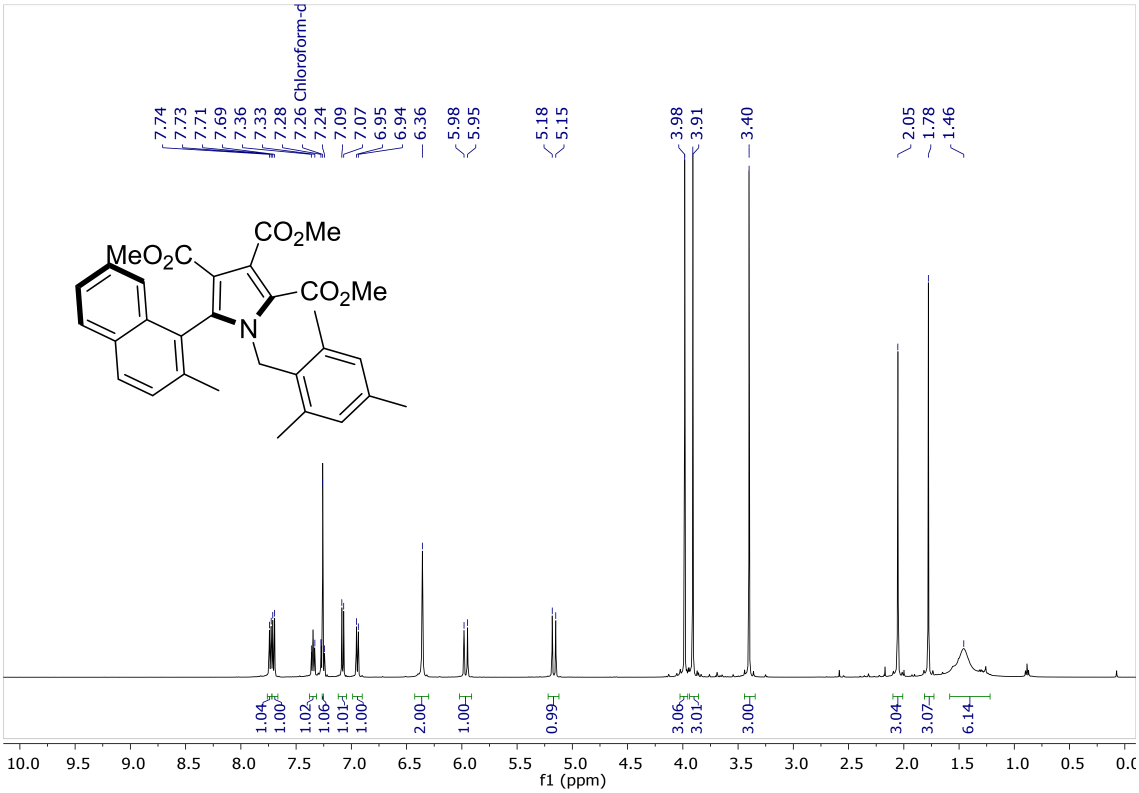

^{13}C NMR ($CDCl_3$, 75 MHz)

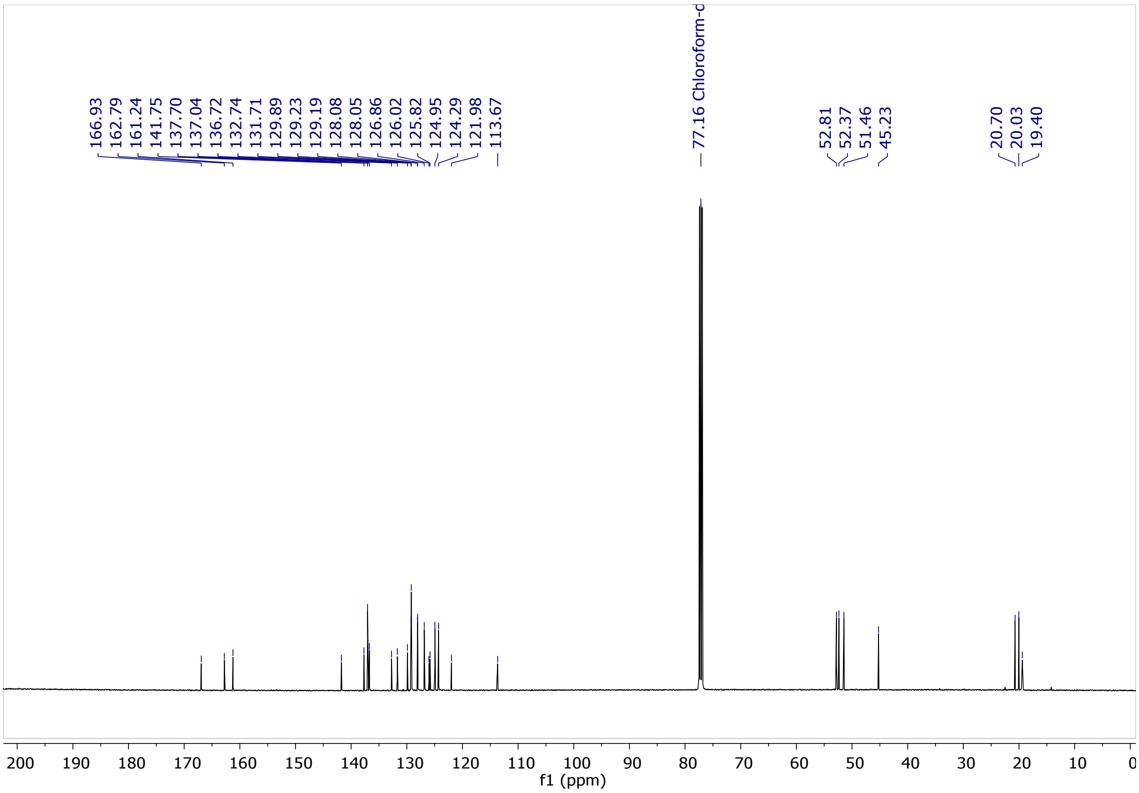

3,4-Di-tert-butyl 2-methyl 5-(2-methylnaphthalen-1-yl)-1-(2,4,6-trimethylbenzyl)-1H-pyrrole-2,3,4-tricarboxylate (17c)

^{1}H NMR ($CDCl_3$, 300 MHz)

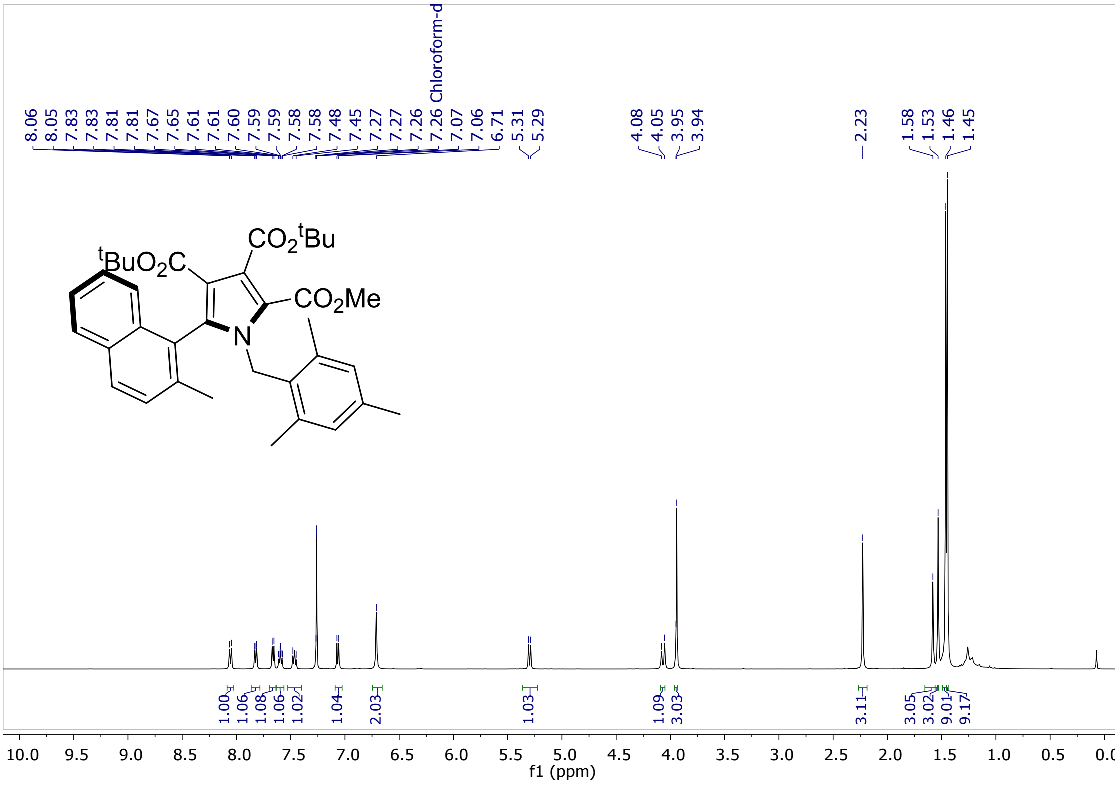

^{13}C NMR ($CDCl_3$, 75 MHz)

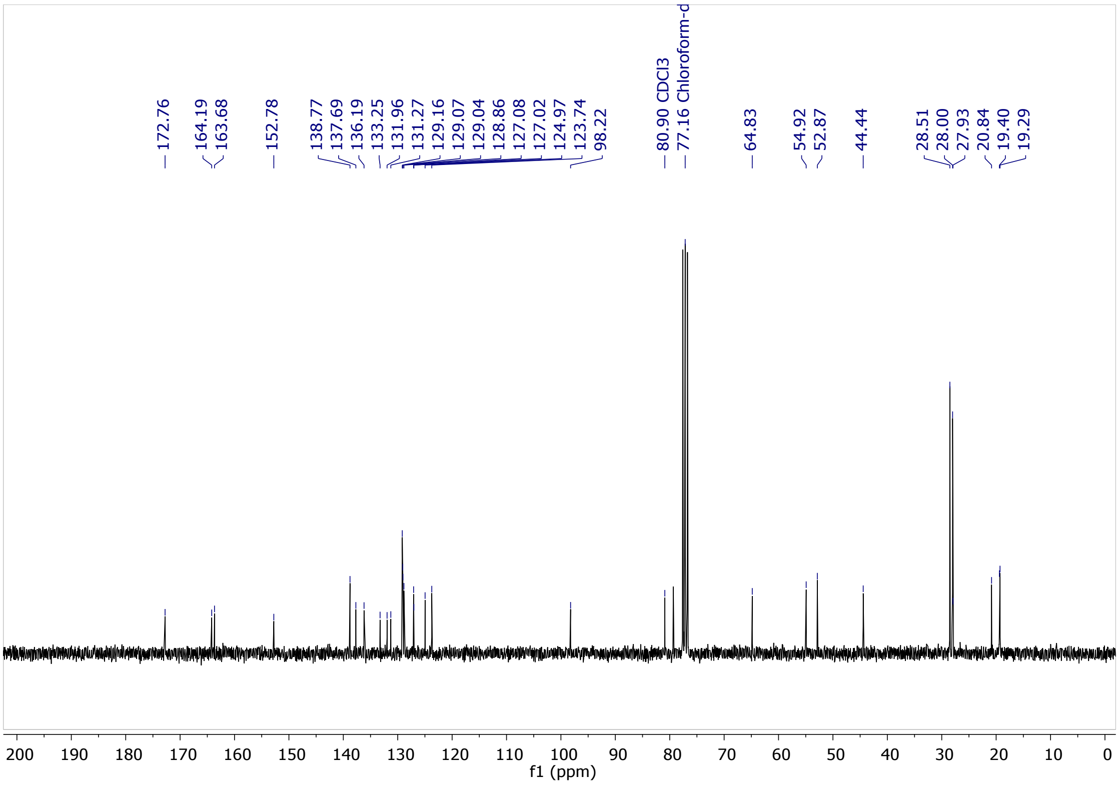

Methyl 3,4-dicyano-5-(2-methylnaphthalen-1-yl)-1-(2,4,6-trimethylbenzyl)-1H-pyrrole-2-carboxylate (17d)

^{1}H NMR ($CDCl_3$, 300 MHz)

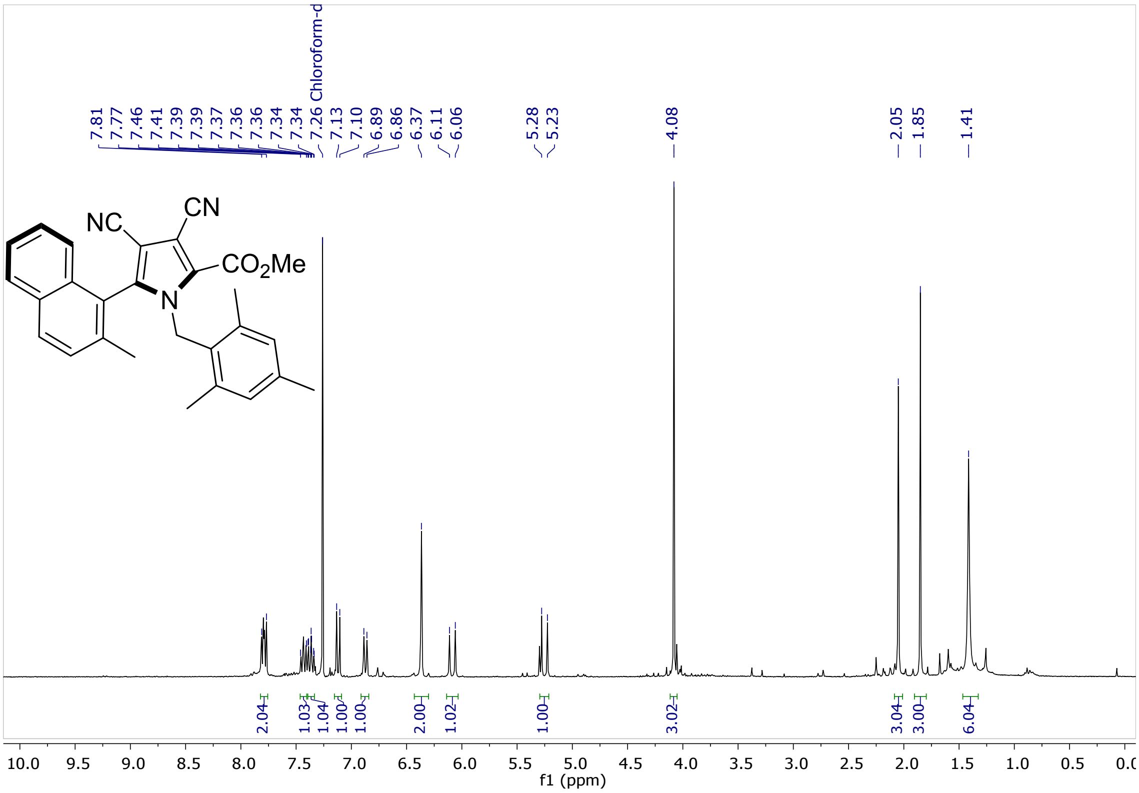

^{13}C NMR ($CDCl_3$, 75 MHz)

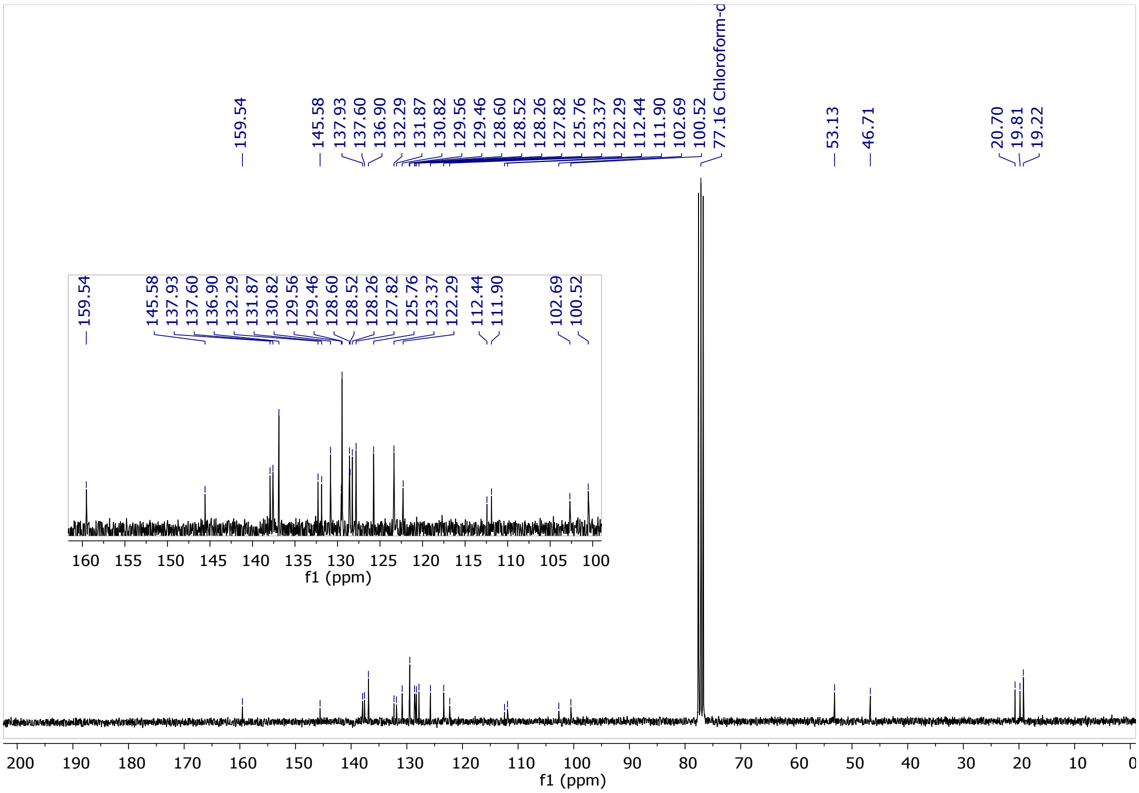

Methyl 2-benzyl-5-methyl-3-(2-methylnaphthalen-1-yl)-4,6-dioxo-2,4,5,6-tetrahydropyrrolo[3,4-c]pyrrole-1-carboxylate (6a)

^{1}H NMR ($CDCl_3$, 300 MHz)

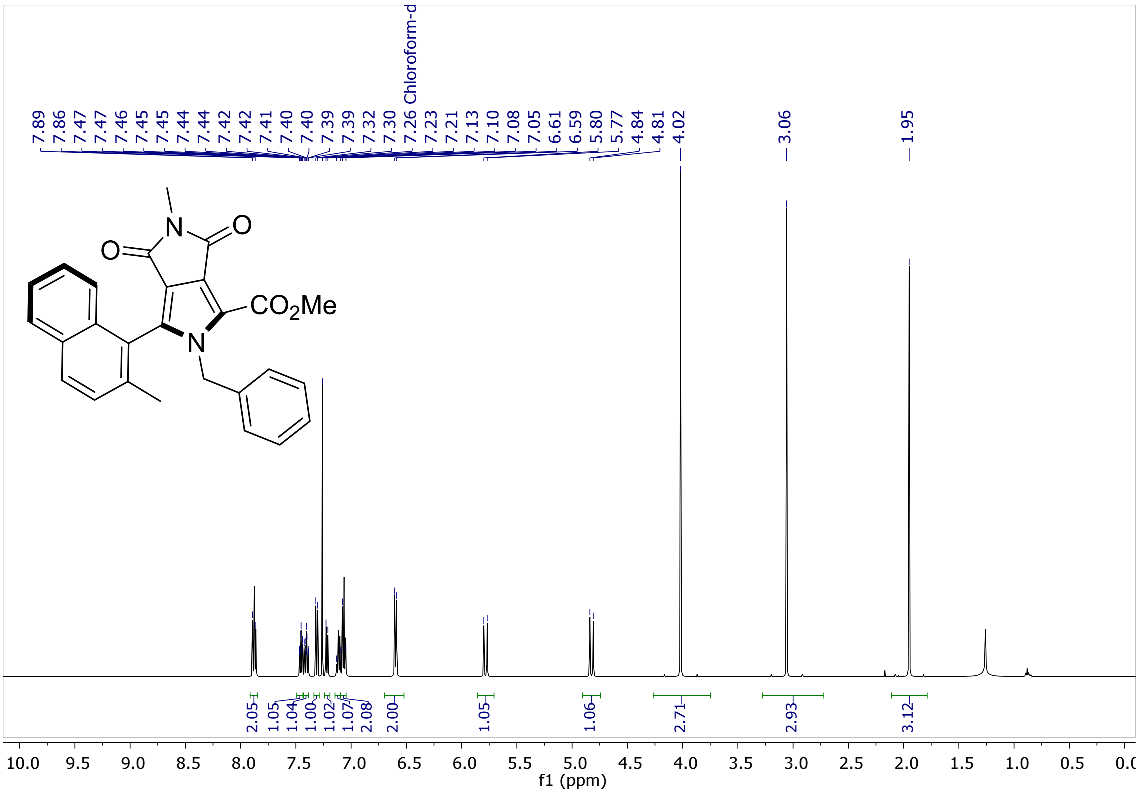

^{13}C NMR ($CDCl_3$, 75 MHz)

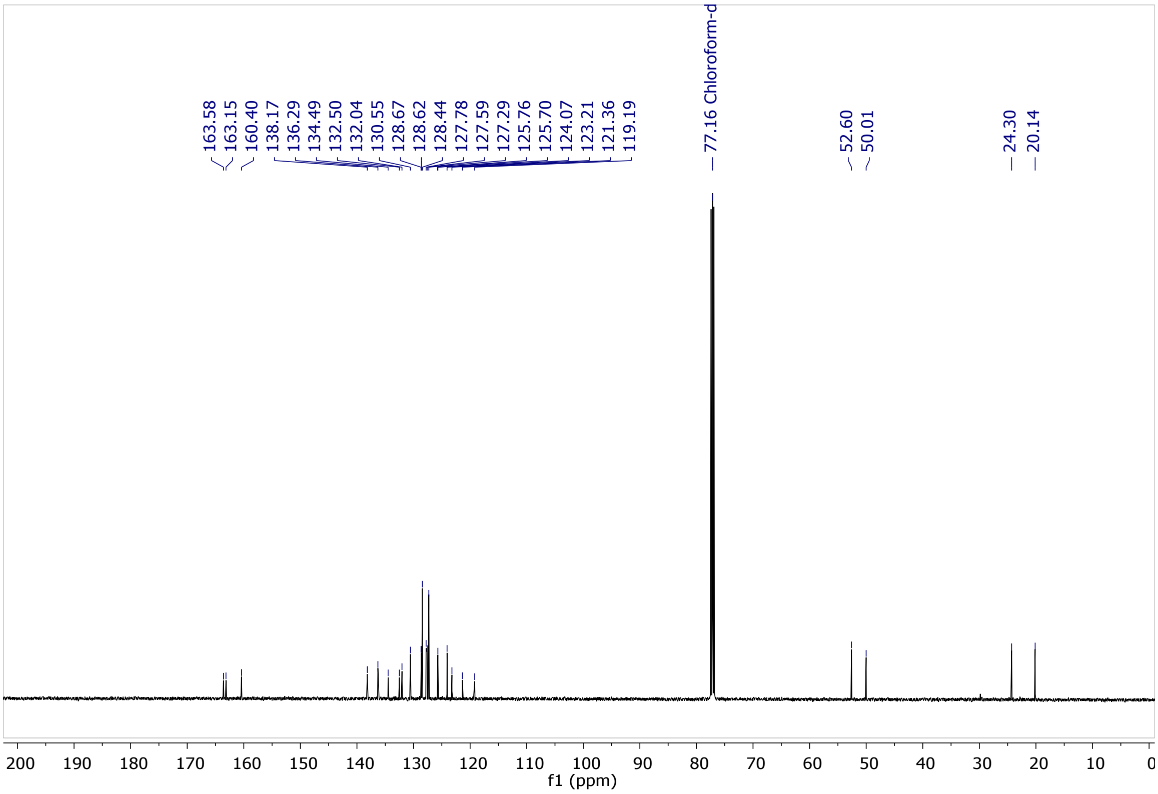

Methyl 2-benzyl-5-methyl-3-(2-methylnaphthalen-1-yl)-4,6-dioxo-2,4,5,6-tetrahydropyrrolo[3,4-c]pyrrole-1-carboxylate (6a)

^{1}H NMR ($CDCl_3$, 300 MHz)

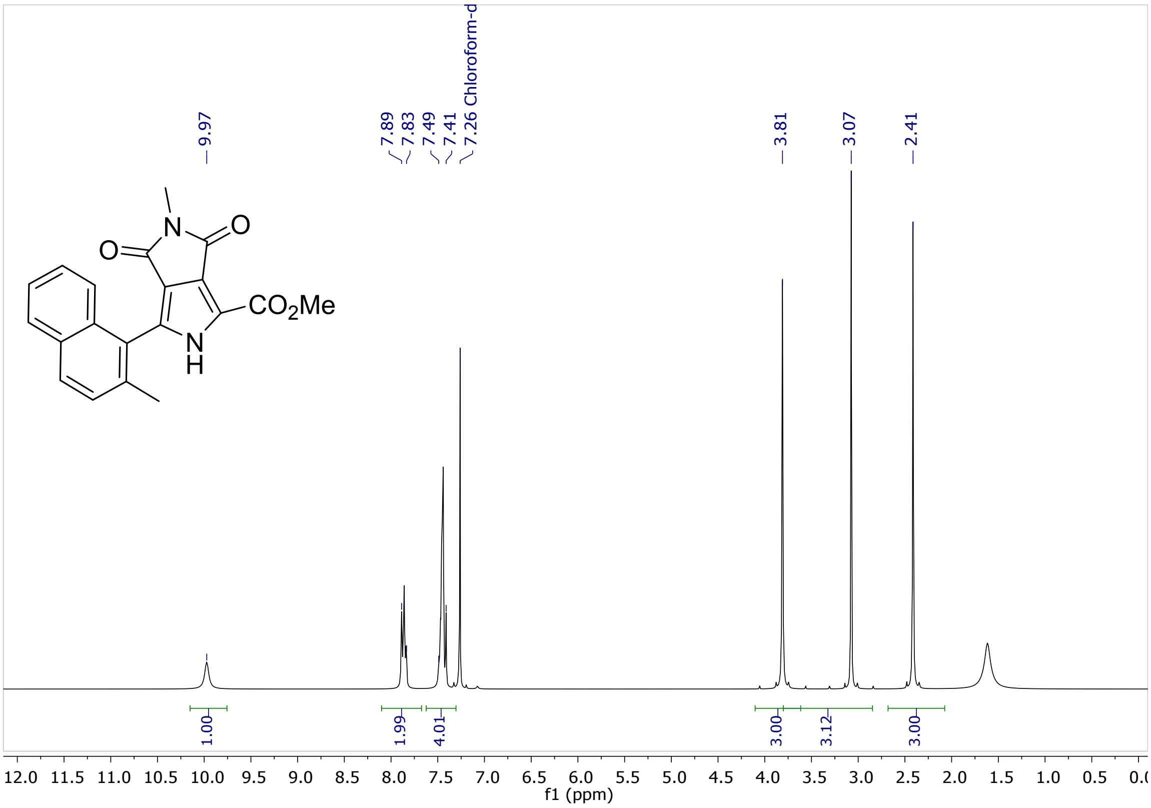

^{13}C NMR ($CDCl_3$, 75 MHz)

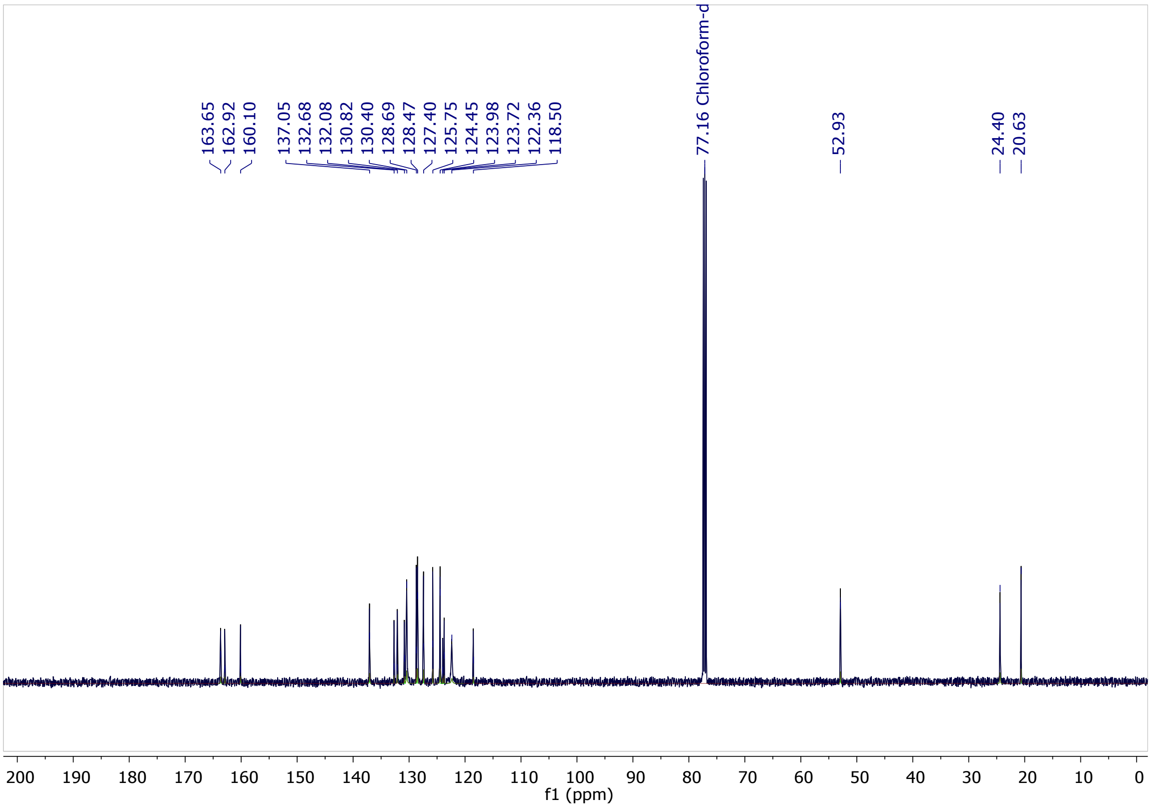

Methyl (1*S*,3*R*,3a*S*,6a*R*)-2-acetyl-5-methyl-3-(2-methylnaphthalen-1-yl)-4,6-dioxooctahydropyrrolo[3,4-c]pyrrole-1-carboxylate (8b)

^{1}H NMR ($CDCl_3$, 300 MHz)

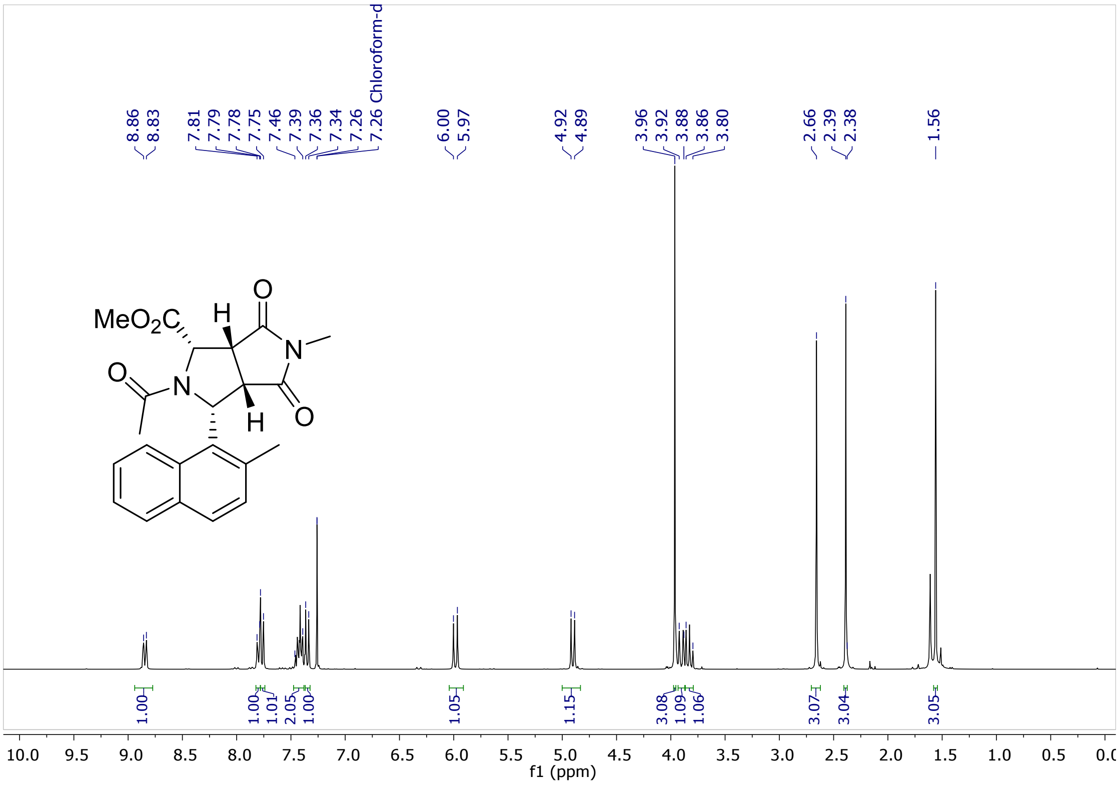

^{13}C NMR ($CDCl_3$, 75 MHz)

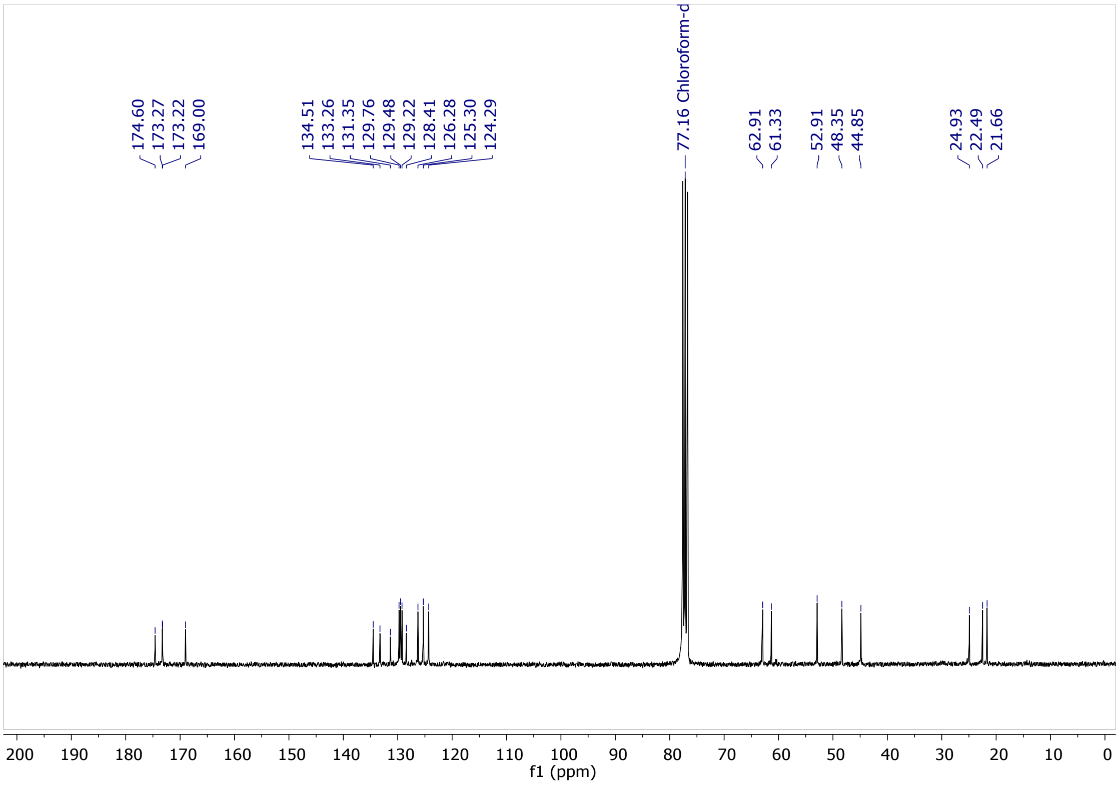

Methyl (1*S*,3*R*,3a*S*,6a*R*)-5-methyl-3-(2-methylnaphthalen-1-yl)-4,6-dioxo-2-tosyloctahydropyrrolo[3,4-c]pyrrole-1-carboxylate (8c)

^{1}H NMR ($CDCl_3$, 300 MHz)

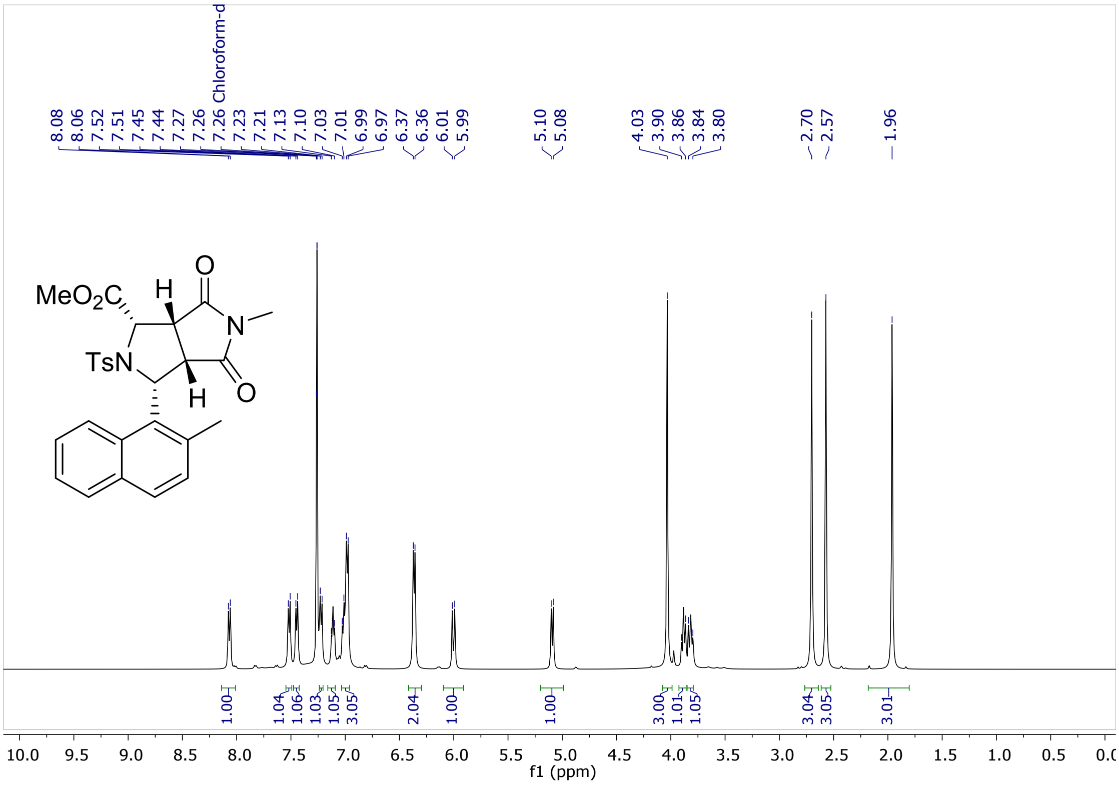

^{13}C NMR ($CDCl_3$, 75 MHz)

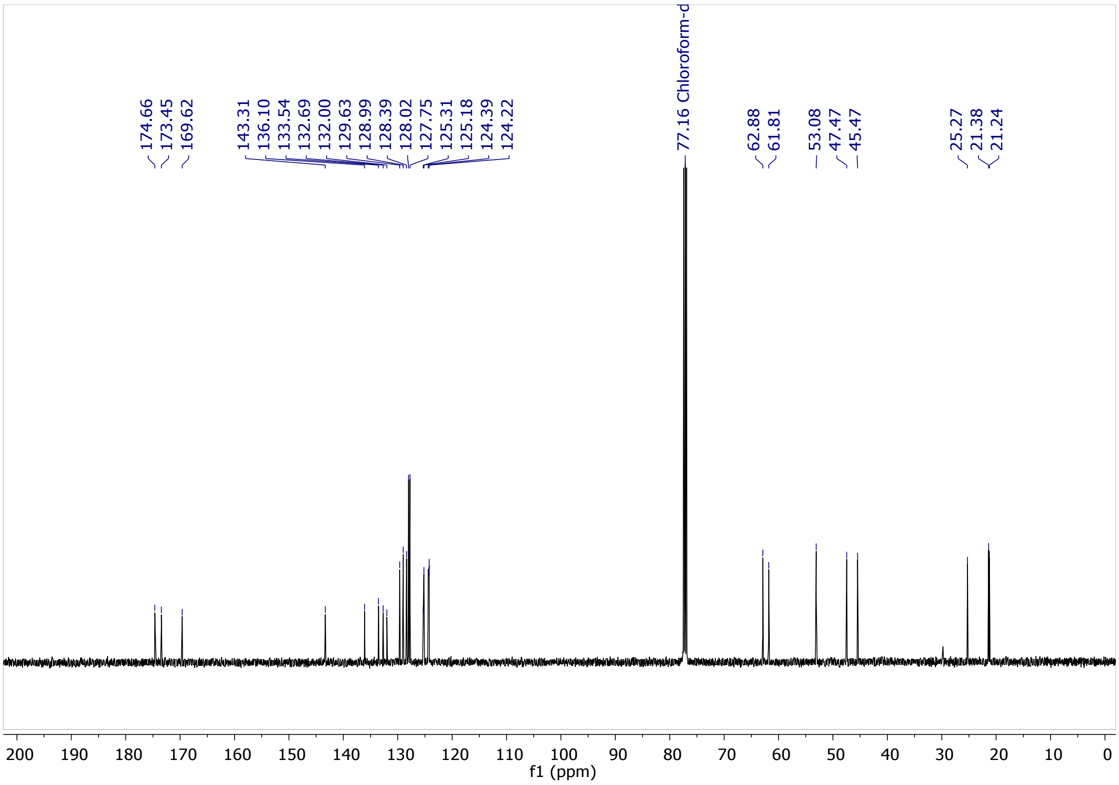

11. References

[1] Companys, S.; Peixoto, P. A.; Bosset, C.; Chassaing, S.; Miqueu, K.; Sotiropoulos, J. M.; Pouységu, L.; Quideau, S. Asymmetric Alkynylation of β-Ketoesters and Naphthols Promoted by New Chiral Biphenylic Iodanes. *Chem. Eur. J.* **2017**, *23*, 13309-13313.

[2] Murugesan, V.; Balakrishnan, V.; Rasappan, R. Nickel-catalyzed cross-coupling reaction of carbamates with silylmagnesium reagents. *Journal of Catalysis*. **2019**, *377*, 293-298.

[3] Lei, X.; Jalla, A.; Abou Shama, M. A.; Stafford, J. M.; Cao, B. Chromatography-Free and Eco-Friendly Synthesis of Aryl Tosylates and Mesylates. *Synth*. **2015**, *47*, 2578-2585.

[4] Feng, W.; Cheng, T.; Zhu, S. Construction of Partially Protected Nonsymmetrical Biaryldiols via Semipinacol Rearrangement of o-NQM Derived from Enynones. *Org. Lett.* **2021,** *23*, 71-75.

[5] Singha, R.; Dhara, S.; Ghosh, M.; Ray, J. K. Copper catalyzed room temperature lactonization of aromatic C–H bond: a novel and efficient approach for the synthesis of dibenzopyranones. *RSC Adv*. **2015**, *5*, 8801-8805.

[6] Sakai, H., Shinto, S., Araki, Y., Wada, T., Sakanoue, T., Takenobu, T. and Hasobe, T. Formation of One-Dimensional Helical Columns and Excimerlike Excited States by Racemic Quinoxaline-Fused [7]Carbohelicenes in the Crystal. *Chem. Eur. J.* **2014**, *20*, 10099-10109.

[7] Chen, H.; Jin, W.; Yu, S. Enantioselective Remote C(sp^3)–H Cyanation via Dual Photoredox and Copper Catalysis. *Org. Lett*. **2020,** *22*, 5910-5914.

[8] Jang, Y. H.; Youn, S. W. Metal-Free C–H Amination for Indole Synthesis. *Org. Lett.* **2014**, *16*, 3720-3723.

[9] Natarajan, P.; König, B. Excited-State 2,3-Dichloro-5,6-dicyano-1,4-benzoquinone (DDQ*) Initiated Organic Synthetic Transformations under Visible-Light Irradiation. *Eur. J. Org. Chem.* **2021**, 2145-2161.
